# Wnt5b/FZD1/LRP6 signaling drives renal fibrosis by triggering cytoplasmic stabilization and nuclear translocation of β-catenin under hypoxia

**DOI:** 10.1016/j.isci.2026.116129

**Published:** 2026-05-28

**Authors:** Zhibin Wu, Zheng Kuang, Jia Liu, Guanghui Dong, Lixia Liang, Shuang Ma, Fei Zou, Guanghai Wang

**Affiliations:** 1 Department of Occupational Health and Occupational Medicine, Guangdong Provincial Key Laboratory of Tropical Disease Research, School of Public Health, Southern Medical University, Guangzhou 510515, China; 2 Research Center for High Altitude Medicine, Qinghai University, Xining 810000, China; 3 Joint International Research Laboratory of Environment and Health, Ministry of Education, Guangdong Provincial Engineering Technology Research Center of Environmental Pollution and Health Risk Assessment, Department of Occupational and Environmental Health, School of Public Health, Sun Yat-sen University, Guangzhou 510080, China

**Keywords:** environmental medicine, molecular biology

## Abstract

Hypobaric hypoxia, a unique environmental feature of high-altitude areas, is associated with kidney disease, but the mechanism remains unclear. In this study, blood and urine samples from high-altitude volunteers are examined, revealing that high-altitude hypoxia causes kidney injury. Renal fibrosis developed in C57BL/6 mice following 28 days of hypobaric hypoxia, suggesting Wnt5b involvement in fibrogenesis under hypoxic conditions. *Wnt5b* knockdown ameliorates hypoxia-induced renal fibrosis *in vivo*. NRK-52E cells undergo partial epithelial-mesenchymal transition and produce more Wnt5b under hypoxic conditions. Wnt5b secreted by NRK-52E cells via exosomes activates canonical Wnt signaling in NRK-49F cells. This activation requires cooperation between FZD1 and LRP6 receptors, which trigger β-catenin cytoplasmic stabilization and nuclear translocation, driving fibroblast activation. These findings suggest that intercellular communication between renal epithelial and mesenchymal cells via the Wnt5b/β-catenin pathway promotes renal fibrosis development under high-altitude hypoxia. This study uncovers the mechanisms underlying renal fibrosis and proposes promising therapeutic targets for altitude-induced kidney disease.

## Highlights

• High-altitude hypoxia causes renal fibrosis
• Wnt5b induces fibroblast activation via the FZD1/LRP6-mediated β-catenin signaling pathway
• Exosomal Wnt5b mediates epithelial-mesenchymal crosstalk under hypoxia
• Targeting Wnt5b ameliorates hypoxia-induced renal fibrosis

## Graphical abstract

**Figure:**
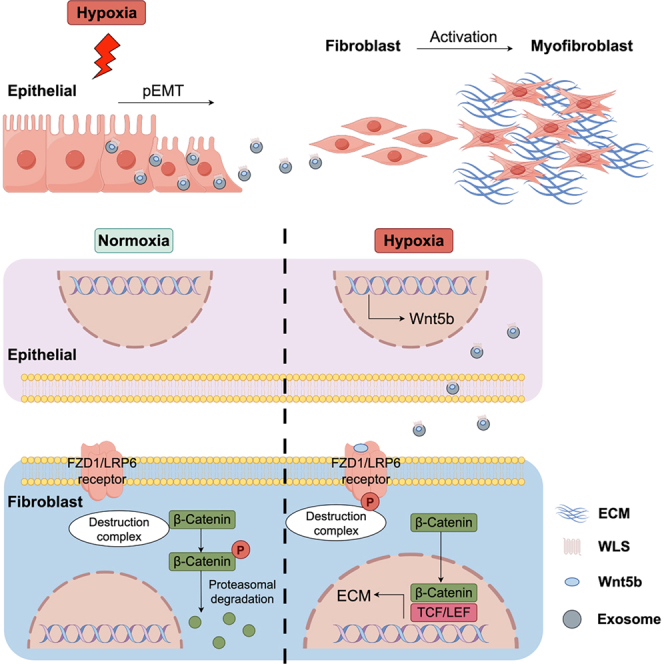

## Introduction

High-altitude regions, characterized by extreme climatic conditions such as low atmospheric pressure, pose significant health risks to human populations, including cognitive dysfunction, cardiovascular disorders, and renal impairment.^1^^,^^2^^,^^3^ The primary adverse effect of high altitude is hypobaric hypoxia resulting from reduced atmospheric pressure.^4^ As altitude increases, atmospheric pressure decreases, reducing the partial pressure of oxygen and the number of oxygen molecules per volume of inhaled air.^5^ Globally, over 100 million people live above 2,500 m.^6^ These residents are continuously exposed to harsh hypobaric hypoxic conditions that cause chronic health complications. While acute high-altitude illnesses are increasingly well characterized, chronic conditions, particularly renal disease, remain understudied.^7^^,^^8^^,^^9^^,^^10^ The detrimental impact of high-altitude hypoxia on renal function has been recognized only recently.^11^^,^^12^ Notably, the prevalence of chronic kidney disease (CKD) among Tibetan adults reaches 19%, which is substantially higher than that observed in sea-level populations.^13^ Although previous studies have demonstrated that chronic high-altitude hypoxia induces renal fibrosis in sheep, the precise molecular mechanisms underlying hypoxia-induced renal fibrosis require further elucidation.^14^

Renal fibrosis, characterized by excessive deposition of extracellular matrix (ECM), is a common hallmark of CKDs of various etiologies.^15^^,^^16^ Myofibroblasts are the primary effectors in fibrosis, promoting its progression through the production and secretion of ECM components.^17^ Renal tubular epithelial cells are the key drivers of fibrosis, acting through the process of partial epithelial-to-mesenchymal transition (pEMT).^18^ During pEMT, tubular epithelial cells partially lose epithelial markers and cell polarity while acquiring mesenchymal features.^19^ These transitioning cells remain attached to the basement membrane and drive renal fibrosis by stimulating fibroblast activation.^20^^,^^21^^,^^22^ Although the association between renal fibrosis and pathological hypoxia has been studied in other contexts, the impact of high-altitude hypoxia on renal fibrosis remains poorly characterized.^23^^,^^24^^,^^25^^,^^26^^,^^27^

The Wnt signaling pathway plays critical roles in organ development, tissue repair, remodeling, and inflammatory responses.^28^ While generally quiescent in adult tissues, Wnt signaling becomes activated following injury.^29^ Following synthesis and endoplasmic reticulum (ER) lipidation, Wnt proteins are transported and secreted via the Wnt ligand secretion (WLS) mediator.^30^ Evidence indicates that Wnt ligands can be transported extracellularly via exosomes or other mechanisms.^30^^,^^31^^,^^32^ The exosomes serve a dual function: they shield the Wnt ligands from degradation in the extracellular environment and facilitate their targeted delivery to recipient cells.^33^ Wnt ligands then bind to Frizzled (FZD) receptors and co-receptors (e.g., LRP5/6, ROR1/2, or RECK) on target cells, initiating canonical or non-canonical signaling pathways.^34^^,^^35^ The FZD receptors, such as FZD1, and the co-receptor LRP6 have been shown to cooperate in mediating canonical Wnt signaling pathways. Upon Wnt binding, the cytoplasmic domain of LRP6 is phosphorylated. This phosphorylation recruits Axin and facilitates disassembly of the β-catenin destruction complex. Consequently, β-catenin accumulates, translocates to the nucleus, and forms a transcriptional complex with TCF/LEF. This complex upregulates the expression of fibrotic mediators, including Snail1, PAI-1, and TRPC6, and promotes fibrosis.^28^^,^^36^^,^^37^ The non-canonical Wnt signaling pathways include Wnt/planar cell polarity (PCP) and the Wnt/Ca^2+^ signaling pathway, operating independently of β-catenin. They are initiated by Wnt ligands binding to specific FZD receptors and co-receptors (e.g., ROR1/2 and Glypican-4), which activate downstream effectors such as NFAT and c-Jun instead of β-catenin.^38^^,^^39^^,^^40^ Previous studies have revealed that tubular epithelial cell-derived Wnt proteins trigger β-catenin signaling and play a pivotal role in fibroblast activation through epithelial-mesenchymal crosstalk.^41^^,^^42^^,^^43^^,^^44^ These findings suggest that the canonical Wnt signaling pathway may contribute to high-altitude hypoxia-induced renal fibrosis. However, the specific Wnt ligands involved and the underlying mechanisms remain to be elucidated.

In this study, we investigated the pathological effects of high-altitude hypoxia on renal tissue from humans and mice while elucidating the molecular mechanisms involved. This work identifies Wnt5b as the dominant hypobaric hypoxia-upregulated Wnt ligand and establishes the Wnt5b/β-catenin axis as a crucial driver of high-altitude hypoxia-induced renal fibrosis.

## Results

### High-altitude hypoxia caused renal injury and fibrosis

**Figure 1:**
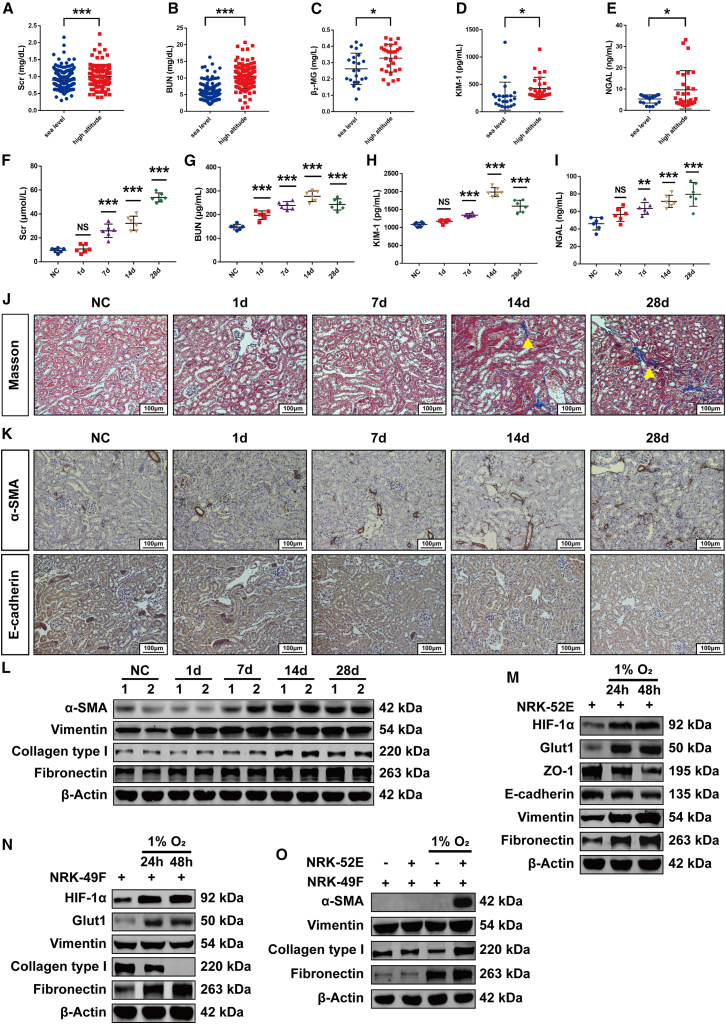
High-altitude hypoxia caused renal injury and fibrosis (A and B) Serum levels of Scr and BUN in volunteers from sea level (Guangzhou) and high altitude (Qinghai). (C–E) Urine levels of β_2_-MG, KIM-1, and NGAL in volunteers from sea level (Guangzhou) and high altitude (Qinghai). (F and G) Serum levels of Scr and BUN in mice from control and high-altitude hypoxia-exposed groups (*n* = 6). (H and I) Urine levels of KIM-1 and NGAL in mice from control and high-altitude hypoxia-exposed groups (*n* = 6). (J) Masson’s trichrome staining of kidney sections. Yellow arrow indicates collagen deposition (*n* = 6). Scale bars, 100 μm. (K) Immunohistochemistry of high-altitude hypoxia-induced changes of α-SMA and E-cadherin in mouse kidneys (*n* = 6). Scale bars, 100 μm. (L) Western blot analysis of high-altitude hypoxia-induced alterations of α-SMA, vimentin, collagen type Ⅰ, and fibronectin in mouse kidneys; samples 1 and 2 in each group represent biological replicates (*n* = 6). (M) Western blot analysis of hypoxia-induced alterations of hypoxia markers and EMT-related proteins in NRK-52E cells (*n* = 3). (N) Western blot analysis of hypoxia-induced alterations of hypoxia markers and fibroblast activation-related proteins in NRK-49F cells (*n* = 3). (O) Western blot analysis revealed the alterations of fibroblast activation-related proteins in NRK-49F cells when co-cultured with NRK-52E cells under hypoxic conditions (*n* = 3). Quantitative analyses of the data presented in (J)–(O) are provided in Figures S1A–S1D and S1I–S1K. Data were shown as mean ± SD. Student’s *t* test or one-way ANOVA was used to determine the significant differences. α = 0.05, ∗*p* < 0.05, ∗∗*p* < 0.01, ∗∗∗*p* < 0.001.

To assess the renal effects of high-altitude hypoxia, we recruited healthy volunteers from sea-level (Guangzhou, 43 m) and high-altitude (Qinghai, 4,058 m) regions, excluding individuals with hypertension or diabetes. Demographic and clinical characteristics are summarized in Table 1. While age, gender, BMI, and lifestyle factors were comparable between groups, high-altitude residents exhibited significantly elevated heart rates and decreased saturation of peripheral oxygen (SpO_2_) levels. Notably, serum creatinine (Scr) and blood urea nitrogen (BUN) levels, along with urinary biomarkers (β_2_-MG, KIM-1, and NGAL), were markedly increased in high-altitude volunteers (Figures 1A–1E).

**Table 1:** Demographic and clinical characteristics of the study subjects

Variable	Sea level (*n* = 296)	High altitude (*n* = 118)	*p*
**Continuous variable (mean ± SD)**			***t* test**

Age (years)	50.77 ± 14.27	53.73 ± 15.56	0.065
BMI	23.77 ± 13.60	25.06 ± 3.94	0.312
Heart rate (BPM)	70.84 ± 11.09	76.46 ± 11.62	<0.000
SpO_2_ (%)	96.78 ± 1.95	90.18 ± 5.10	<0.000

**Categorical variable (%)**			**chi-square**

Male	41.89%	42.37%	1.000
Smoking	18.92%	13.56%	0.250
Drinking	12.84%	11.02%	0.741

In parallel experiments, C57BL/6 mice exposed to simulated 5,000-m hypobaric hypoxia exhibited significantly elevated Scr and BUN levels, along with increased urinary KIM-1 and NGAL excretion (Figures 1F–1I). Masson’s trichrome staining revealed time-dependent accumulation of renal interstitial collagen (Figure 1J). Immunohistochemical analysis demonstrated increased α-SMA and decreased E-cadherin expression in hypoxic kidneys (Figure 1K), which was confirmed by western blot (Figure 1L) and qPCR (Figure S1E). These results collectively demonstrate that high-altitude hypoxia induces renal injury and promotes fibrotic progression *in vivo*.

### Under hypoxic conditions, renal tubular epithelial cells underwent pEMT, driving fibroblast activation

To investigate the cellular responses to hypoxia, we exposed NRK-52E (tubular epithelial) and NRK-49F (fibroblast) cells to 1% O_2_ conditions. Although the viability of both cell lines was inhibited by hypoxia when cultured in single-cell, co-culture with NRK-52E cells enhanced the viability of NRK-49F cells under hypoxic conditions (Figures S1F–S1H). Western blot analysis demonstrated time-dependent upregulation of vimentin and fibronectin, along with downregulation of ZO-1 and E-cadherin in hypoxic NRK-52E cells (Figure 1M). In contrast, NRK-49F cells exhibited limited responsiveness to hypoxia (Figure 1N). Notably, hypoxic co-culture with NRK-52E cells promoted activation of NRK-49F cells, as indicated by increased expression of α-SMA, collagen type I, and fibronectin (Figure 1O). These findings indicate that hypoxia induces pEMT in tubular epithelial cells, which subsequently activate fibroblasts through intercellular communication.

### Wnt5b/β-catenin was activated in hypobaric hypoxia-induced renal fibrosis

**Figure 2:**
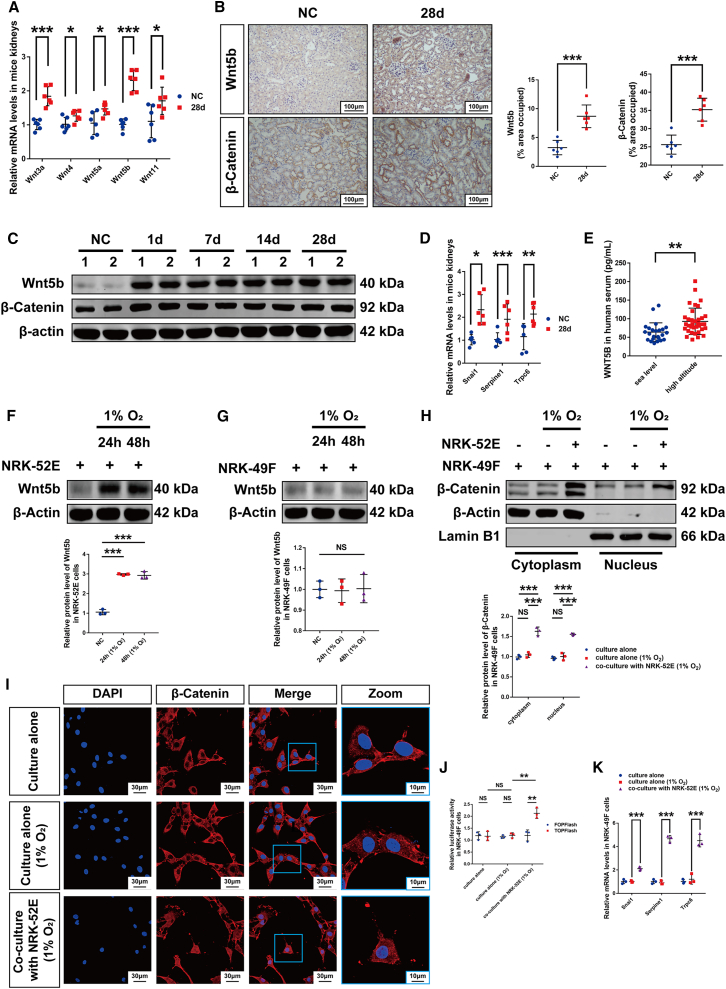
Wnt5b/β-catenin was activated in hypoxia-induced renal fibrosis (A) qPCR analysis showed increased mRNA levels of 5 Wnt ligands in mice kidneys (*n* = 6). (B) Immunohistochemistry of high-altitude hypoxia-induced changes of Wnt5b and β-catenin in mouse kidneys (*n* = 6). Scale bars, 100 μm. (C) Western blot analysis of high-altitude hypoxia-induced alterations of Wnt5b and β-catenin in mouse kidneys; samples 1 and 2 in each group represent biological replicates (*n* = 6). (D) qPCR analysis showed the high-altitude hypoxia-induced changes of mRNA levels of β-catenin target genes in mouse kidneys (*n* = 6). (E) Serum level of Wnt5b in volunteers from sea level (Guangzhou) and high altitude (Qinghai). (F and G) Western blot analysis of hypoxia-induced alterations of Wnt5b in NRK-52E and NRK-49F cells (*n* = 3). (H) Western blot analysis revealed the alteration of β-catenin in both the cytoplasm and nucleus of NRK-49F cells co-cultured with NRK-52E cells under hypoxic conditions (*n* = 3). (I) Immunofluorescence analysis showed the localization of β-catenin in NRK-49F cells co-cultured with NRK-52E cells under hypoxic conditions (*n* = 3). Scale bars: 30 μm (DAPI, β-catenin, merge); 10 μm (zoom). (J) Dual-luciferase reporter assay revealed the changes of β-catenin/TCF transcriptional activity in NRK-49F cells co-cultured with NRK-52E cells under hypoxic conditions (*n* = 3). (K) qPCR analysis showed the changes of mRNA levels of β-catenin target genes in NRK-49F cells co-cultured with NRK52E cells under hypoxic conditions (*n* = 3). Quantitative analyses of the data presented in (C) are provided in Figure S2B. Data were shown as mean ± SD. Student’s *t* test or one-way ANOVA was used to determine the significant differences. α = 0.05, ∗*p* < 0.05, ∗∗*p* < 0.01, ∗∗∗*p* < 0.001.

To elucidate the molecular mechanisms of renal fibrosis induced by high-altitude hypoxia, we investigated the role of Wnt/β-catenin signaling. Using qPCR, we analyzed the alteration of all 19 Wnt ligands in kidneys of mice exposed to simulated 5,000-m hypobaric hypoxia. Among the 19 Wnt ligands analyzed, five (*Wnt3a*, *Wnt4*, *Wnt5a*, *Wnt5b*, and *Wnt11*) were significantly upregulated, with *Wnt5b* showing the most pronounced increase (Figure 2A); eight remained stable; two were significantly downregulated (Figure S2A); and four were undetectable by qPCR (Ct values > 35 cycles). This distinct expression profile strongly suggests that Wnt5b plays a key role in hypoxia-induced renal pathology. Immunohistochemistry demonstrated elevated Wnt5b and β-catenin expression in hypoxic kidneys (Figure 2B), which was confirmed by western blot (Figure 2C). Hypobaric hypoxia also upregulated β-catenin target genes (*Snai1*, *Serpine1*, and *Trpc6*) in mouse kidneys after 28 days of exposure (Figure 2D). Consistently, high-altitude residents exhibited significantly higher serum Wnt5b levels than sea-level volunteers (Figure 2E).

*In vitro* studies revealed that hypoxia induced Wnt5b upregulation in NRK-52E cells (Figure 2F) but not in NRK-49F cells (Figure 2G). Moreover, the expression levels of Wnt3a, Wnt4, Wnt5a, and Wnt11 remained weak in NRK-52E cells under hypoxic conditions (Figure S2C). Co-culture experiments demonstrated upregulated expression and nuclear translocation of β-catenin in NRK-49F cells under hypoxic conditions (Figures 2H and 2I) and increased levels of active β-catenin (Figure S2D). Furthermore, β-catenin/TCF transcriptional activity was significantly enhanced (Figure 2J), concomitant with upregulation of its downstream target genes (Figure 2K). These findings demonstrate activation of the Wnt5b/β-catenin pathway in renal fibrosis induced by high-altitude hypoxia.

### Wnt5b activated fibroblasts mainly through the β-catenin signaling pathway

**Figure 3:**
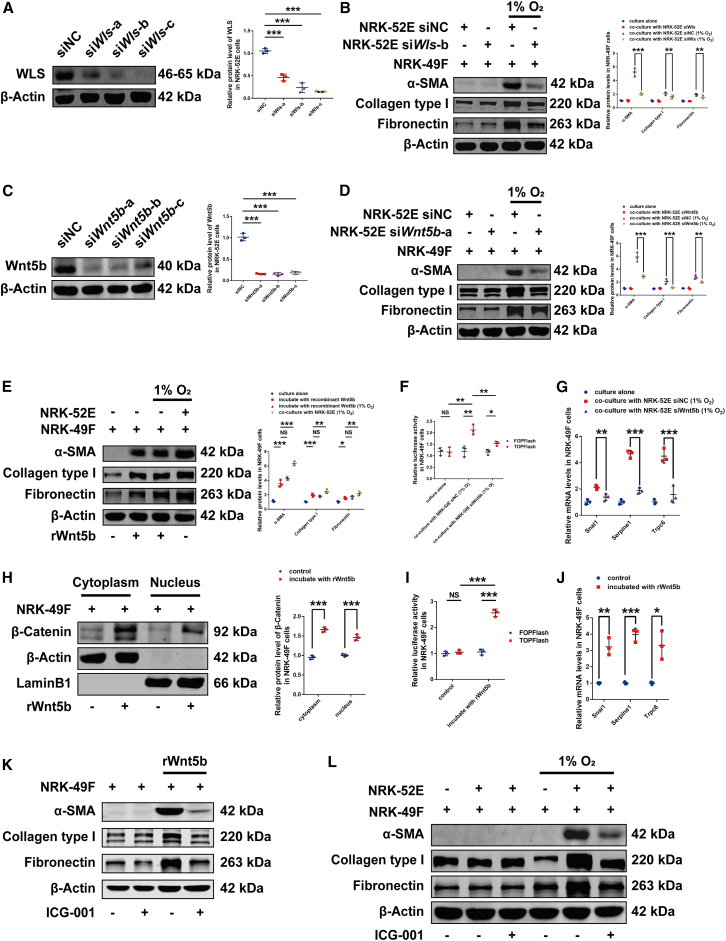
The role of Wnt5b/β-catenin signaling pathway in high-altitude hypoxia-induced renal fibrosis (A) Western blot analysis revealed the silencing effect of siRNA on *Wls* expression in NRK-52E cells. (B) Western blot analysis revealed the alterations of fibroblast activation-related proteins in NRK-49F cells when co-cultured with NRK-52E cells in which *Wls* was silenced by siRNA, under hypoxic conditions. (C) Western blot analysis revealed the silencing effect of siRNA on *Wnt5b* expression in NRK-52E cells. (D) Western blot analysis revealed the alterations of fibroblast activation-related proteins in NRK-49F cells when co-cultured with NRK-52E cells in which *Wnt5b* was silenced by siRNA, under hypoxic conditions. (E) Western blot analysis revealed the alterations of fibroblast activation-related proteins in NRK-49F cells induced by 10 ng/mL recombinant Wnt5b. (F) Dual-luciferase reporter assay revealed the changes of β-catenin/TCF transcriptional activity in NRK-49F cells when co-cultured with NRK-52E cells in which *Wnt5b* was silenced by siRNA, under hypoxic conditions. (G) qPCR analysis showed the changes of mRNA levels of β-catenin target genes in NRK-49F cells when co-cultured with NRK-52E cells in which *Wnt5b* was silenced by siRNA, under hypoxic conditions. (H) Western blot analysis revealed the alteration of β-catenin in both the cytoplasm and nucleus of NRK-49F cells induced by 10 ng/mL recombinant Wnt5b protein. (I) Dual-luciferase reporter assay revealed the changes of β-catenin/TCF transcriptional activity in NRK-49F cells induced by 10 ng/mL recombinant Wnt5b protein. (J) qPCR analysis showed the changes of mRNA levels of β-catenin target genes in NRK-49F cells induced by 10 ng/mL recombinant Wnt5b protein. (K) Western blot analysis revealed the effect of 10 μM ICG-001 on Wnt5b-induced elevation of fibroblast activation-related proteins in NRK-49F cells. (L) Western blot analysis revealed the effect of 10 μM ICG-001 on the elevation of fibroblast activation-related proteins in NRK-49F cells when co-cultured with NRK-52E cells under hypoxic conditions. Quantitative analyses of the data presented in (K) and (L) are provided in Figures S3H and S3I. A working concentration of 20 μmol/L was applied for the siRNA transfection. Data were shown as mean ± SD. Student’s *t* test or one-way ANOVA was used to determine the significant differences. *n* = 3, α = 0.05, ∗*p* < 0.05, ∗∗*p* < 0.01, ∗∗∗*p* < 0.001.

Because WLS is indispensable for the intracellular transport and secretion of Wnt proteins, we silenced *Wls* in NRK-52E cells using distinct small interfering RNA (siRNA) to investigate the role of Wnt5b signaling in fibroblast activation (Figure 3A; Figure S3A). Co-culture experiments showed that *Wls* knockdown in NRK-52E cells inhibited hypoxia-induced fibroblast activation in NRK-49F cells (Figure 3B; Figure S3B). Similarly, *Wnt5b* knockdown in NRK-52E cells (Figure 3C; Figure S3C) attenuated fibroblast activation, resembling the effects of *Wls* silencing (Figure 3D; Figure S3D). Recombinant Wnt5b protein (10 ng/mL) induced fibroblast activation after 48-h treatment under normoxic conditions (Figure 3E), suggesting that Wnt5b may contribute to fibroblast activation.

Under hypoxic conditions, *Wnt5b* knockdown in NRK-52E cells suppressed β-catenin/TCF transcriptional activity and its downstream genes in co-cultured NRK-49F cells (Figures 3F and 3G). Treatment of NRK-49F cells with 10 ng/mL recombinant Wnt5b protein for 48 h induced cytoplasmic accumulation and nuclear translocation of β-catenin (Figure 3H), along with increased levels of active β-catenin (Figure S3E), resulting in enhanced β-catenin/TCF-dependent transcriptional activity and upregulation of downstream target genes (Figures 3I and 3J; Figure S3F). ICG-001 (10 μM), a selective β-catenin inhibitor, inhibited fibroblast activation triggered by either recombinant Wnt5b protein or hypoxic co-culture with NRK-52E cells (Figures 3K–3L).

**Figure 4:**
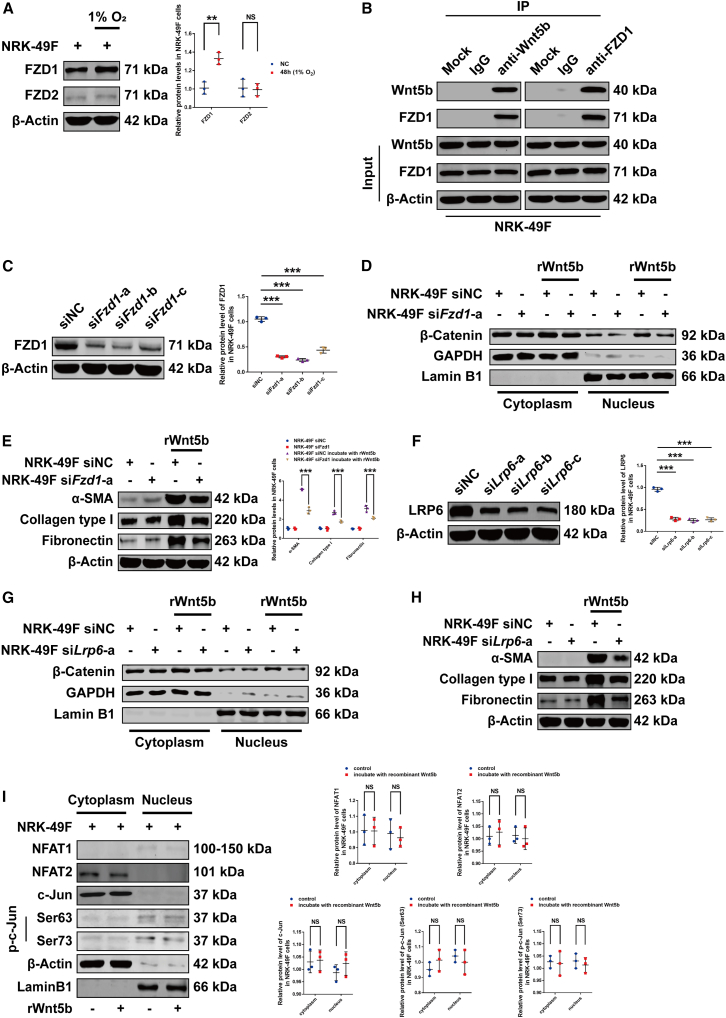
Wnt5b triggered high-altitude hypoxia-induced renal fibrosis through the canonical rather than non-canonical pathway (A) Western blot analysis revealed hypoxia-induced alterations in FZD1 and FZD2 proteins in NRK-49F cells. (B) CoIP followed by western blot analysis showed that recombinant Wnt5b co-precipitated with FZD1 in NRK-49F cells. (C) Western blot analysis revealed the silencing effect of siRNA on *Fzd1* expression in NRK-49F cells. (D) Western blot analysis revealed that *Fzd1* knockdown significantly decreased β-catenin levels in both the cytoplasm and nucleus of Wnt5b-treated NRK-49F cells. (E) Western blot analysis revealed that *Fzd1* knockdown significantly altered the expression of fibroblast activation-related proteins in Wnt5b-treated NRK-49F cells. (F) Western blot analysis revealed the silencing effect of siRNA on *Lrp6* expression in NRK-49F cells. (G) Western blot analysis revealed that *Lrp6* knockdown significantly decreased β-catenin levels in both the cytoplasm and nucleus of Wnt5b-treated NRK-49F cells. (H) Western blot analysis revealed that *Lrp6* knockdown significantly altered the expression of fibroblast activation-related proteins in Wnt5b-treated NRK-49F cells. (I) Western blot analysis revealed no alterations of NFAT1, NFAT2, c-Jun, and p-c-Jun (Ser63 and Ser73) in both the cytoplasm and nucleus of NRK-49F cells induced by 10 ng/mL recombinant Wnt5b protein. Quantitative analyses of the data presented in (D), (G), and (H) are provided in Figures S4C, S4F, and S4G. A working concentration of 20 μmol/L was applied for the siRNA transfection. Data were shown as mean ± SD. Student’s *t* test or one-way ANOVA was used to determine the significant differences. *n* = 3, α = 0.05, ∗*p* < 0.05, ∗∗*p* < 0.01, ∗∗∗*p* < 0.001.

Given the critical role of FZD receptors in Wnt signaling, we first profiled the mRNA levels of ten *Fzd*s in NRK-49F cells under hypoxia by qPCR (Figure S4A). We found that *Fzd1* and *Fzd7* are upregulated by 2.42-fold and 1.37-fold, respectively; *Fzd2*, *Fzd4*, *Fzd5*, and *Fzd6* are downregulated; *Fzd3* remains unchanged; while other subtypes are undetected (Ct values > 35 cycles) (Figure S4A). Subsequent western blot analysis confirmed a significant hypoxic upregulation of FZD1 at the protein level, contrasting with weak FZD2 expression (Figure 4A). This specific induction suggested FZD1 as the dominant receptor for Wnt5b-mediated β-catenin activation. Supporting this, co-immunoprecipitation (coIP) assays confirmed a direct interaction between recombinant Wnt5b and endogenous FZD1 (Figure 4B). To definitively establish its functional role, we performed siRNA-mediated knockdown of *Fzd1* in NRK-49F cells and assessed its impact on Wnt5b-driven signaling (Figure 4C; Figure S4B). Knockdown of *Fzd1* in NRK-49F cells significantly attenuated the Wnt5b-induced increase in β-catenin levels and its nuclear translocation (Figure 4D), thereby suppressing fibroblast activation (Figure 4E; Figure S4D). To determine the contributions of the canonical β-catenin pathway or non-canonical pathways to Wnt5b-induced NRK-49F cell activation, we knocked down *Lrp6* expression with siRNA (Figure 4F; Figure S4E), as LRP6 activation is exclusively required for Wnt-induced β-catenin stabilization. *Lrp6* knockdown in NRK-49F cells significantly reduced Wnt5b-induced β-catenin accumulation and nuclear translocation (Figure 4G), consequently inhibiting fibroblast activation (Figure 4H; Figure S4G). In contrast, 10 ng/mL recombinant Wnt5b protein treatment for 48 h did not increase the expression or nuclear localization of key non-canonical Wnt signaling mediators (c-Jun, NFAT1, and NFAT2) in NRK-49F cells (Figure 4I). Together, these results indicate that under hypoxic conditions, Wnt5b secreted by tubular epithelial cells signals through FZD1 and LRP6 receptors to activate fibroblasts, primarily via the canonical β-catenin pathway.

### Wnt5b deficiency ameliorated hypobaric hypoxia-induced renal fibrosis in mice

**Figure 5:**
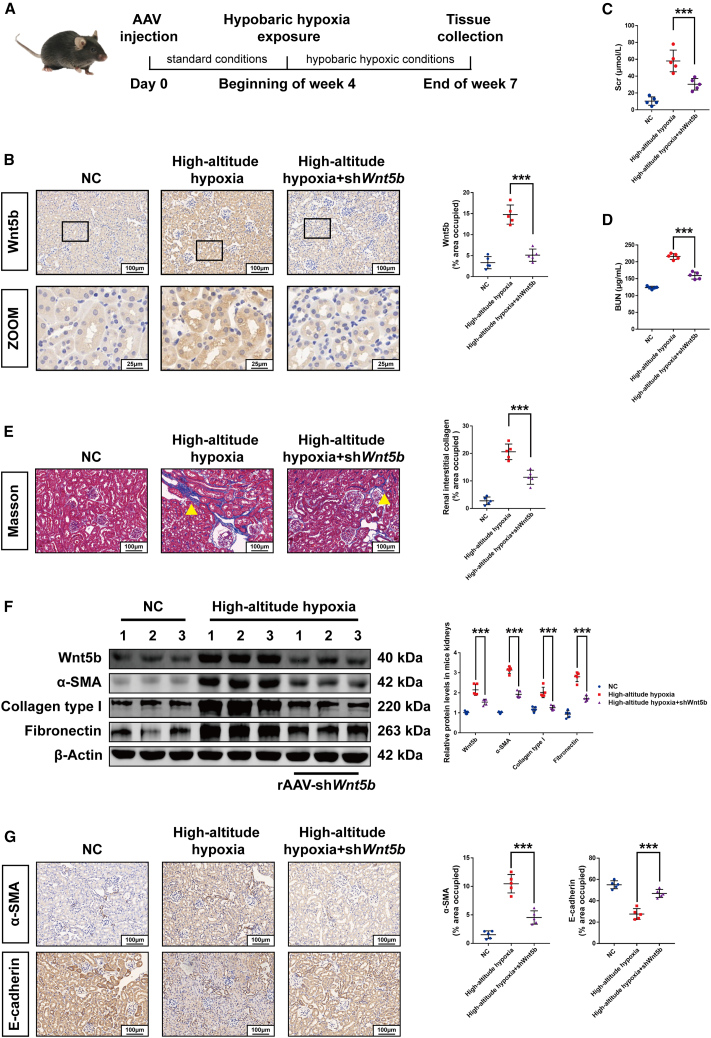
Wnt5b deficiency ameliorated high-altitude hypoxia-induced renal fibrosis in mice (A) Timeline of the animal experiment. (B) Immunohistochemistry analysis showed the expression of Wnt5b protein in proximal tubules. Scale bars: 100 (Wnt5b) and 25 μm (zoom). (C and D) Serum levels of Scr and BUN in mice. (E) Masson’s trichrome staining of kidney sections. Yellow arrow indicates collagen deposition. Scale bars, 100 μm. (F) Western blot analysis demonstrated alterations of Wnt5b, α-SMA, collagen type Ⅰ, and fibronectin in mice kidneys; samples 1, 2, and 3 in each group represent biological replicates. (G) Immunohistochemistry analysis showed changes of α-SMA and E-cadherin in mouse kidneys. Scale bars, 100 μm. Data were shown as mean ± SD. One-way ANOVA was used to determine the significant differences. *n* = 5, α = 0.05, ∗*p* < 0.05, ∗∗*p* < 0.01, ∗∗∗*p* < 0.001.

To elucidate the role of Wnt5b upregulation in hypobaric hypoxia-induced renal fibrosis, we administered rAAV-sh*Wnt5b* via tail vein injection (Figure 5A). The successful knockdown of *Wnt5b* in mouse kidneys was verified by immunohistochemistry analysis, which also confirmed its predominant expression in renal tubular epithelial cells (Figure 5B). *Wnt5b* silencing attenuated the hypobaric hypoxia-induced elevation of Scr and BUN levels (Figures 5C and 5D) and reduced renal interstitial collagen accumulation (Figure 5E). Western blot and immunohistochemistry analysis showed decreased expression of fibrotic markers (α-SMA, collagen type I, and fibronectin) (Figures 5F and 5G), demonstrating that *Wnt5b* knockdown confers protection against hypoxia-induced renal fibrosis *in vivo*.

### Wnt5b was secreted via exosomes

**Figure 6:**
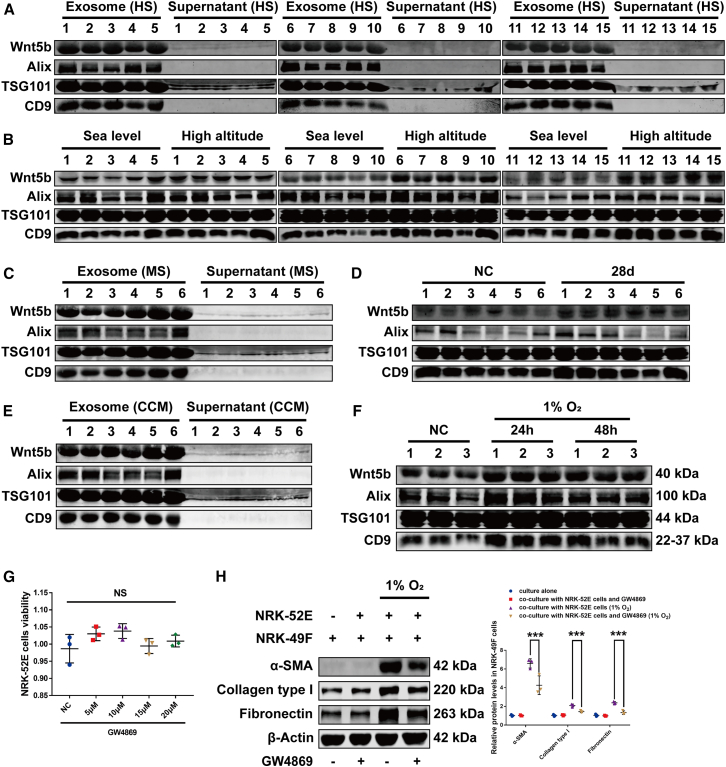
Hypoxia-induced Wnt5b was transported extracellularly via exosomes (A) Wnt5b distribution in human serum (*n* = 15). (B) Serum exosomal Wnt5b levels in volunteers from sea level (Guangzhou) vs. high altitude (Qinghai) (*n* = 15). (C) Wnt5b distribution in mouse serum (*n* = 6). (D) Serum exosomal Wnt5b in mice from control vs. high-altitude hypoxia-exposed groups (*n* = 6). (E) Wnt5b in exosome vs. exosome-free medium of cell culture (*n* = 6). (F) Exosome-borne Wnt5b in control vs. hypoxia-exposed cell cultures (*n* = 3). (G) CCK-8 assay showed the viability of NRK-52E cells treated with different concentrations of GW4869 (*n* = 3). (H) Western blot analysis revealed the effect of 20 μM GW4869 on the elevation of fibroblast activation-related proteins in NRK-49F cells when co-cultured with NRK-52E cells under hypoxic conditions (*n* = 3). HS represents human serum, MS represents mouse serum, and CCM represents cell culture medium. Data were shown as mean ± SD. One-way ANOVA was used to determine the significant differences. α = 0.05, ∗*p* < 0.05, ∗∗*p* < 0.01, ∗∗∗*p* < 0.001.

To investigate whether Wnt5b is secreted via exosomes, we isolated exosomes from serum of humans, mice, and cell cultures, using Alix, TSG101, and CD9 as markers. Wnt5b was exclusively detected in exosomes from human serum (Figure 6A), with significantly higher levels in high-altitude volunteers compared to sea-level controls (Figure 6B). Similarly, Wnt5b in mouse serum was exosome carried (Figure 6C) and increased following hypobaric hypoxia exposure (Figure 6D). Hypoxic NRK-52E cells also secreted Wnt5b via exosomes (Figures 6E and 6F). GW4869 (a classic exosome inhibitor) treatment for 48 h did not significantly affect NRK-52E cell viability (Figure 6G). However, inhibition of exosome release by 20 μM GW4869 in NRK-52E cells attenuated hypoxia-induced activation of co-cultured NRK-49F cells (Figure 6H). This effect indicates that hypoxia-induced renal fibrosis is driven by Wnt5b secreted via exosomes from tubular epithelial cells.

## Discussion

Pathological hypoxia has been considered a major driver for renal fibrosis, but the relationship between high-altitude hypoxia and renal fibrosis has not been clarified. In this study, we found that high-altitude hypoxia can induce renal fibrosis and elucidated the role of Wnt5b signaling in this process. Here, we observed elevated Scr and BUN levels, along with increased urinary biomarkers (KIM-1, NGAL, and β_2_-MG) in high-altitude residents, indicating that high-altitude hypoxia causes kidney injury. These findings confirm prior reports of higher CKD prevalence in Tibetan populations.^13^ Since kidney injury is the major driver of CKD, hypobaric hypoxia-induced renal injury may contribute to the development of CKD in high-altitude areas. To investigate the possibility and mechanisms of renal fibrosis induced by high-altitude hypoxia, we exposed C57BL/6 mice to simulated hypobaric hypoxia at an altitude of 5,000 m. The animal model exhibited characteristic renal pathology, featuring prominent tissue hypoxia and progressive fibrosis. Further investigations revealed that the crosstalk between renal tubular epithelial cells and fibroblasts played pivotal roles in hypoxia-induced renal fibrosis. We found that, under hypoxic conditions, renal tubular epithelial cells underwent pEMT and secreted bioactive molecules that activated fibroblasts, resulting in increased production of ECM components. Our findings consolidate the recent consensus that epithelial cell dysfunctions are the primary driver of renal fibrosis.^45^

Since increasing evidence indicates that Wnts play crucial roles in renal fibrosis, we further examined the involvement of Wnts in hypoxia-induced renal fibrosis.^41^^,^^46^ Of the nineteen Wnt family members analyzed, five were upregulated in mouse kidneys following hypobaric hypoxia exposure. *Wnt5b* exhibited the most pronounced increase (2.28-fold), suggesting that it may play a leading role in the hypoxic response, while the other four upregulated ligands (*Wnt3a*, *Wnt4*, *Wnt5a*, and *Wnt11*) may have ancillary or synergistic functions. This strong upregulation makes Wnt5b a prime candidate for functional studies. Immunohistochemistry revealed that Wnt5b is predominantly localized in renal epithelial cells. *In vitro* studies further revealed that hypoxia selectively upregulates Wnt5b in renal tubular epithelial cells, with no response observed in fibroblasts. In renal tubular epithelial cells under hypoxic conditions, only Wnt5b was significantly upregulated, while the alterations of Wnt3a, Wnt4, Wnt5a, and Wnt11 were all insignificant. This finding further solidifies the predominant role of Wnt5b in our model. Though Wnt5b expression remained stable during renal fibrosis progression in unilateral ureteral obstruction models,^47^ we observed significant Wnt5b upregulation in multiple hypoxic settings, including mouse kidney tissues, serum (from mice and humans), and hypoxia-treated tubular epithelial cells. Considering the stress-specific expression profile of the Wnt family, this discrepancy in Wnt5b expression may be due to the difference of stimuli employed in the experiments. In other words, Wnt5b upregulation might be specific to hypobaric hypoxia. We further knocked down *Wnt5b in vivo* using a recombinant adeno-associated virus carrying short hairpin RNA targeting *Wnt5b* (rAAV-sh*Wnt5b*), which is designed to exert an effect exclusively in renal tubular epithelial cells. The *in vivo* experiments demonstrated that tubular-specific *Wnt5b* deficiency attenuates hypobaric hypoxia-induced renal fibrosis in mice. In the co-culture system, tubular epithelial cell-derived Wnt5b activates fibroblasts under hypoxic conditions. Of note, despite having been associated with multiple diseases including osteoarthritis, osteoporosis, obesity, type 2 diabetes mellitus, neuropathology, and cancers,^48^ Wnt5b has not been implicated in renal fibrosis until this study.

Wnt ligands trigger cellular responses through either β-catenin-dependent canonical pathway or β-catenin-independent non-canonical pathways (PCP/Ca^2+^). Herein, we demonstrate that Wnt5b selectively activates the canonical pathway in fibroblasts, evidenced by β-catenin stabilization and nuclear translocation, while sparing key non-canonical effectors (c-Jun and NFAT1/NFAT2). The critical role of β-catenin was further confirmed by pharmacological inhibition with ICG001 (a classic β-catenin inhibitor^49^^,^^50^^,^^51^), which nearly abrogated Wnt5b-induced NRK-49F cell activation, particularly under hypoxic conditions. Mechanistically, β-catenin activation by Wnt5b in NRK-49F cells depends on receptors FZD1 and LRP6. Hypoxia exposure upregulated the mRNA expression of two *Fzd* receptors in fibroblasts. Of these, *Fzd1* exhibited the most pronounced increase (2.42-fold), implicating a potential leading role. This finding was corroborated by a significant upregulation of FZD1 protein under hypoxia. Moreover, we demonstrated a direct interaction between Wnt5b and FZD1, collectively pointing to the predominant function of FZD1 in this context. *Fzd1* knockdown substantially attenuates Wnt5b-dependent β-catenin stabilization and nuclear translocation, effectively blocking fibroblast activation. These results corroborate previous reports identifying FZD1 as an essential component of the canonical Wnt signaling pathway.^52^^,^^53^^,^^54^ *Lrp6* knockdown similarly attenuates Wnt5b-triggered β-catenin signaling and subsequent fibroblast activation. This demonstrates the selectivity of Wnt5b signaling, as LRP6 is specifically required for β-catenin activation but dispensable for non-canonical Wnt pathways. Together, our findings reveal that Wnt5b drives high-altitude hypoxia-induced renal fibrosis primarily through canonical Wnt/β-catenin pathway activation, with negligible involvement of non-canonical Wnt signaling.

Regarding the secretion mechanism of Wnt5b, our study assessed Wnt5b levels in both exosomal fractions and exosome-free medium across multiple biological samples. We demonstrated that Wnt5b is primarily secreted via exosomes. Inhibition of exosome biogenesis and release using GW4869 (a neutral sphingomyelinase/nSMase inhibitor^55^^,^^56^) prevented pEMT-induced fibroblast activation, confirming the exosome-dependent secretion pathway for Wnt5b. Building on prior structural characterization of Wnt secretion mechanisms, including the cryoelectron microscopy (cryo-EM) structure of the Wnt8-Wntless complex,^30^ we propose that Wnt5b is localized to exosome membranes. However, the exact biophysical mechanisms governing Wnt5b binding to its receptor and subsequent activation need further investigation.

Despite our findings confirming a significant role for Wnt5b in hypoxia-induced renal fibrosis, its inhibition did not fully suppress the fibrotic process. This incomplete suppression could be partially attributed to an insufficient siRNA-mediated knockdown, which may have allowed residual function of Wnt5b. Besides, additional signaling pathways might be involved in our model. Accumulating evidence suggests that hypoxia-inducible factors (HIFs) play essential roles in hypoxia-induced renal fibrosis by regulating a wide range of target genes, including transforming growth factor β (TGF-β).^57^^,^^58^ Previous findings have revealed that HIF-1α is capable of augmenting the expression of Wnt ligands, such as Wnt7a and Wnt11.^59^^,^^60^^,^^61^ Our study found that HIF-1α is induced by hypoxia, suggesting that it may be involved in Wnt5b upregulation under hypobaric hypoxic conditions. TGF-β is a well-established driver of renal fibrosis.^62^ Previous studies have indicated complex and context-dependent crosstalk between Wnt/β-catenin and TGF-β/Smad signaling.^63^ Future studies are required to elucidate the precise mechanism by which this interplay drives hypobaric hypoxia-induced renal fibrosis.

Our animal and cellular models consistently demonstrated that hypoxic conditions upregulate Wnt5b expression, which subsequently drives renal fibrosis through a series of downstream signaling events. Correspondingly, elevated levels of renal injury biomarkers were detected in the serum and urine of high-altitude residents, accompanied by increased circulating Wnt5b. These clinical findings lend supportive evidence to the translational relevance of our proposed mechanism, suggesting that a similar pathological process occurs in humans under chronic hypoxic exposure. Future longitudinal studies incorporating direct histological assessment (such as renal biopsy) or advanced imaging techniques are warranted to confirm the progression of renal fibrosis in this population.

Our study elucidates the critical role of exosome-derived Wnt5b in high-altitude hypoxia-induced renal fibrosis, mediated through activation of the β-catenin signaling pathway. This finding not only advances our understanding of the pathogenesis of high-altitude nephropathy but also implies a potentially conserved mechanism underlying various fibrotic diseases. For example, canonical Wnt signaling has been shown to promote glycolysis in hepatic stellate cells and contribute to liver fibrosis^64^; similarly, in idiopathic pulmonary fibrosis, while TGF-β is considered the central driver, its downstream signaling extensively intersects with the β-catenin pathway.^65^^,^^66^^,^^67^ These observations suggest that although the initial triggers of fibrosis, such as viral infection, toxins, or hypoxia, may differ across organs, they likely converge on common terminal pathways, such as β-catenin activation, which promotes myofibroblast differentiation and ECM deposition. This convergence is mediated by multiple ligand-receptor systems, including TGF-β, Wnt, and JNK signaling.^68^^,^^69^^,^^70^^,^^71^ Specifically, our work reveals a mechanism by which hypoxia induces cells to secrete Wnt5b via exosomes, facilitating intercellular communication and amplifying fibrotic responses. Targeting this axis, for instance by inhibiting exosome release or disrupting the Wnt5b/β-catenin interaction, may hold therapeutic promise not only for high-altitude nephropathy but also for other fibrosis-related conditions. Thus, our findings provide a conceptual framework and potential targets for the development of broad-spectrum anti-fibrotic strategies.

In conclusion, Wnt5b plays a crucial role in renal fibrosis under high-altitude hypoxia. Tubular epithelial cell-derived Wnt5b mediates intercellular communication to activate fibroblasts via the β-catenin signaling pathway. Our study may help elucidate the mechanisms of hypobaric hypoxia-induced renal fibrosis and inform the development of preventive and therapeutic strategies.

### Limitations of the study

While our study provides insights into hypobaric hypoxia-induced renal fibrosis, several limitations warrant consideration. First, the fibrosis status was not assessed in humans at high altitude due to the unavailability of appropriate non-invasive evaluation methods. Second, our study focused on tubular epithelial-fibroblast crosstalk and did not investigate potential Wnt5b origins from other cell types under hypoxic conditions. Third, a limitation of this study is the use of only male mice, which prevents the assessment of potential sex-specific effects given estrogen’s regulatory role in Wnt5b expression. Fourth, though other Wnt ligands were not assessed in our study due to their limited expression changes, their potential contributions to hypobaric hypoxia-induced renal fibrosis cannot be excluded, and their potential ancillary roles warrant future investigation. Finally, animals were exposed to hypobaric hypoxia for up to 4 weeks, which may not fully replicate the chronic pathological process of renal fibrosis in high-altitude residents. Further epidemiological and experimental studies are needed to elucidate the precise mechanisms by which Wnt5b/β-catenin signaling mediates hypobaric hypoxia-induced renal fibrosis and CKD.

## Resource availability

### Lead contact

Requests for further information should be directed to and will be fulfilled by the lead contact, Dr. Guanghai Wang (ghwang@smu.edu.cn).

### Materials availability

This study did not generate new unique reagents.

### Data and code availability

• All data reported in this paper will be shared by the lead contact upon request.
• This paper does not generate new code.
• Any additional information required to reanalyze the data reported in this paper is available from the lead contact upon request.

## Acknowledgments

This study was supported by the 10.13039/501100001809National Natural Science Foundation of China (grant nos. 82130054 and 82271911).

## Author contributions

Z.W., writing – original draft, visualization, and methodology; Z.K., writing – original draft, visualization, and methodology; J.L., investigation; G.D., investigation; L.L., investigation; S.M., supervision and methodology; F.Z., supervision, funding acquisition, and conceptualization; G.W., writing – review and editing, supervision, methodology, funding acquisition, and conceptualization.

## Declaration of interests

The authors declare no competing interests.

## Declaration of generative AI and AI-assisted technologies in the writing process

During the preparation of this article, the authors used DeepSeek to improve the readability of some texts. After using this tool, the authors reviewed and edited the content as needed and took full responsibility for the content of the publication.

## STAR★Methods

### Key resources table

**Table:** 

REAGENT or RESOURCE	SOURCE	IDENTIFIER
**Antibodies**		

anti-HIF-1α	Abcam	ab179483; RRID: AB_2732807
anti-Glut1	Cell Signaling Technology	12939S; RRID: AB_2687899
anti-E-cadherin	Cell Signaling Technology	14472S; RRID: AB_2728770
anti-ZO-1	Proteintech	21773-1-AP; RRID：AB_10733242
anti-α-SMA	Cell Signaling Technology	19245S; RRID：AB_2734735
anti-Vimentin	Proteintech	10366-1-AP; RRID：AB_2273020
anti-Collagen type Ⅰ	Abcam	ab270993; RRID: AB_2927551
anti-Fibronectin	Abcam	ab45688; RRID: AB_732380
anti-Alix	Proteintech	67715-1-Ig; RRID: AB_2882905
anti-TSG101	Proteintech	67381-1-Ig; RRID：AB_2882628
anti-CD9	Cell Signaling Technology	98327S; RRID：AB_3351665
anti-WLS	Proteintech	17950-1-AP; RRID：AB_2034928
anti-Wnt5b	Santa Cruz	sc-376249; RRID：AB_11010021
anti-Wnt5b	Bioss	bs-8010R
anti-Wnt3a	Bioss	bs-1700R; RRID: AB_10856383
anti-Wnt4	Bioss	bs-6134R; RRID：AB_11100292
anti-Wnt5a	Bioss	bs-1948R; RRID：AB_10856979
anti-Wnt11	Bioss	bs-42347R
anti-β-Catenin	Cell Signaling Technology	8480S; RRID：AB_11127855
anti-active β-Catenin	Cell Signaling Technology	13537S; RRID：AB_2798251
anti-FZD1	Affinity Biosciences	DF4882; RRID：AB_2837174
anti-FZD1	Santa Cruz	sc-398082; RRID：AB_2725749
anti-FZD2	Affinity Biosciences	AF5282; RRID：AB_2837768
anti-LRP6	Affinity Biosciences	DF2995; RRID：AB_2840974
anti-*c*-Jun	Affinity Biosciences	AF6090; RRID：AB_2834984
anti-p-*c*-Jun (Ser63)	Affinity Biosciences	AF3089; RRID: AB_2834526
anti-p-*c*-Jun (Ser73)	Affinity Biosciences	AF3095; RRID：AB_2834532
anti-NFAT1	Affinity Biosciences	DF7189; RRID：AB_2839141
anti-NFAT2	Affinity Biosciences	DF6446; RRID：AB_2838409
anti-β-Actin	Proteintech	66009-1-Ig; RRID: AB_2687938
anti-GAPDH	Proteintech	60004-1-Ig; RRID: AB_2687938
anti-Lamin B1	Proteintech	12987-1-AP; RRID：AB_2136290
anti-TCF4	Proteintech	13838-1-AP; RRID: AB_2199812

**Bacterial and virus strains**		

Recombinant adeno-associated virus type 9 carrying short hairpin RNA targeting *Wnt5b* (rAAV9-sh*Wnt5b*) and its negative controls (rAAV9-shNC)	Tsingke Biotech (Beijing, China)	

**Biological samples**		

SPF male C57BL/6 mice	Southern Medical University Laboratory Animal Center	
Blood samples	Qinghai University and Sun Yat-sen University	
Urine specimens	Qinghai University and Sun Yat-sen University	

**Chemicals, peptides, and recombinant proteins**		

Recombinant Wnt5b protein	CUSABIO (China)	CSB-YP026139MO
ICG-001	Selleck Chemicals LLC	S2662
GW4869	Selleck Chemicals LLC	S7609

**Critical commercial assays**		

Creatinine detection kit	Biosharp	BL890B
Urea Nitrogen detection kit	Boxbio	AKNM002M
Human β_2_-MG ELISA kit	Elabscience	E-EL-H2188c
Human KIM-1 ELISA kit	Proteintech	KE00136
Human NGAL ELISA kit	Proteintech	KE00144
Mouse NGAL ELISA kit	Proteintech	KE10045
Human Wnt5b ELISA kit	MEIMIAN	MM-62581H1

**Experimental models: Cell lines**		

The rat kidney tubule epithelial cell line (NRK-52E)	KeyGen BioTech (Nanjing, China)	KGG1101-1
The rat kidney fibroblast cell line (NRK-49F)	Cell Resource Center, Institute of Basic Medical Sciences, CAMS/PUMC (Beijing, China)	3101RATSCSP5213

**Software and algorithms**		

SPSS 23.0	IBM	
GraphPad Prism 6	GraphPad Software	
Adobe Illustrator 2023	Adobe	

### Experimental model and study participant details

#### Human participants

Baseline data (Table 1), serum, and urine samples were collected from native high-altitude and native sea-level volunteers, with those having a history of hypertension and diabetes excluded. One hundred eighteen volunteers (≥18 years) from Qinghai, China (high altitude, average altitude 4058 m; 50 males and 68 females), and two hundred ninety-six volunteers (≥18 years) from Guangzhou, China (sea level, average altitude 43 m; 124 males and 172 females) were interviewed and tested for β_2_-microglobulin (β_2_-MG), human kidney injury molecule 1 (KIM-1), and neutrophil gelatinase-associated lipocalin (NGAL) in urine, as well as serum creatinine (Scr) and blood urea nitrogen (BUN). Body mass index (BMI), heart rate (beats per minute, BPM), and saturation of peripheral oxygen (SpO_2_) were measured.

All experiments involving human were conducted according to the ethical policies and procedures approved by the Institutional Review Board of Qinghai University (Approval no. PJ202401-26). Informed consent was obtained from all the participants involved in the study. All research procedures were performed in accordance with the ethical principles of the Declaration of Helsinki.

#### Animals

SPF male C57BL/6 mice wild-type mice (all aged 10 weeks) were obtained from the Southern Medical University Laboratory Animal Center. The mice were housed in an SPF environment under a 12/12-h light/dark cycle. A hypobaric chamber (Yuyan Instruments Co., Ltd., Shanghai, China) was utilized to simulate hypobaric hypoxia at an altitude of 5,000 m. In the treatment groups, six mice per group were exposed to hypobaric hypoxia for 1, 7, 14, and 28 days, respectively. In the control group, six mice were maintained at an altitude of 50 m. Samples, including serum, urine, and kidneys, were collected from the mice for subsequent experiments.

Male mice were used in this study to minimize variability associated with the estrous cycle and fluctuating estrogen levels, thereby allowing a more consistent assessment of hypoxia-induced Wnt5b expression and its role in promoting renal fibrosis. The potential influence of sex hormones on Wnt5b regulation was not addressed in the current investigation.

All experiments involving animals were conducted according to the ethical policies and procedures approved by the Southern Medical University Animal Care and Use Committee (Approval no. SMUL2022285).

#### Cell lines

The rat kidney tubule epithelial cell line (NRK-52E) was purchased from KeyGen BioTech, Nanjing, China (KGG1101-1). The rat kidney fibroblast cell line (NRK-49F) was purchased from Cell Resource Center, Institute of Basic Medical Sciences, CAMS/PUMC, Beijing, China (3101RATSCSP5213).

NRK-52E and NRK-49F were cultured in DMEM (Gibco, USA) media with 10% fetal bovine serum (ExCell Bio, China). In the normoxia groups, cells were incubated at 37 °C in a humidified atmosphere with 5% CO_2_ and 21% O_2_. In the hypoxia groups, cells were incubated at 37 °C in a humidified atmosphere with 5% CO_2_ and 1% O_2_.

In co-culture experiments, NRK-52E cells on transwell inserts with 95% confluence were placed into six-well plates with NRK-49F cells at the bottom (Corning, USA).

All cell lines were obtained from commercial suppliers and were authenticated by short tandem repeat (STR) profiling prior to distribution. Prior to experiments, all cell lines were confirmed to be mycoplasma-free by PCR assay (PB180525; Procell, China).

### Method details

#### Intrarenal adeno-associated virus (AAV) delivery

Recombinant adeno-associated virus type 9 carrying short hairpin RNA targeting *Wnt5b* (rAAV9-sh*Wnt5b*) and its negative controls (rAAV9-shNC) were designed and purchased from Tsingke Biotech (Beijing, China). The Ksp-cadherin promoter was used in the construct to control the transcription of shRNA, thereby achieving targeted knockdown in renal tubular epithelial cells. The titers of rAAV9-sh*Wnt5b* and rAAV9-shNC were approximately 10^13^ viral genomes/mL. A total of 5×10^11^ viral genomes of rAAV9-sh*Wnt5b* or rAAV9-shNC were delivered to the kidneys via tail vein injection.

Mice received tail vein injections of either rAAV9-sh*Wnt5b* or rAAV9-shNC and were then housed under standard conditions for three weeks to ensure adequate AAV transduction and *Wnt5b* knockdown. Subsequently, beginning in the fourth week, the mice were subjected to a four-week period of hypobaric hypoxia to simulate high-altitude conditions. Finally, at the end of seventh week, the animals were euthanized, and kidney tissue, serum, and urine were collected for subsequent analysis.

#### Immunohistochemistry

Kidney tissues underwent fixation in 4% paraformaldehyde, were paraffin-embedded, and sliced into 4 μm-thick sections. Following a 2-h heat treatment at 65°C, the paraffin-embedded tissue sections were sequentially immersed in 100% toluene, and ethanol solutions of decreasing concentrations (100%, 95%, 85%, and 75%) to remove the paraffin. The sections were then heated in a 0.5M EDTA solution (pH 8.0) using a microwave at 100°C for 20 min. This was followed by a 10-min incubation with 3% H_2_O_2_ at room temperature and overnight incubation with antibodies diluted in 3% BSA in PBS at 4°C. The antibodies employed included anti-α-SMA (19245S; Cell Signaling Technology, USA), anti-E-cadherin (14472S; Cell Signaling Technology, USA), anti-Wnt5b (sc-376249; Santa Cruz, USA), and anti-β-Catenin (8480S; Cell Signaling Technology, USA). Subsequently, the sections were incubated with corresponding secondary antibodies for an hour, treated with DAB staining solution for 3 min, and counterstained with hematoxylin for 30 s. Ultimately, microscopic images of the stained sections were acquired using a Nikon microscope (Tokyo, Japan).

#### Assessment of renal injury

The levels of Scr and BUN were determined by detection kits (Biosharp, China; Boxbio, China), according to the manufacturer’s instructions. The levels of β_2_-MG, KIM-1, NGAL and Wnt5b were measured by using specific enzyme-linked immunosorbent assay (ELISA) kits (Elabscience, China; Proteintech, China; Proteintech, China; MEIMIAN, China) according to the manufacturer’s instructions.

#### RNA interference

Small interfering RNA (siRNA) targeting *Wls*, *Wnt5b*, *Fzd1* and *Lrp6* were synthesized by IGE Biotechnology (Guangzhou, China), with the sequences provided in Table S1. For cell transfection, NRK-52E cells were seeded in six-well plates and grown to 50% confluence. Transfections were then performed using Lipofectamine 3000 (Invitrogen, USA) according to the manufacturer’s protocol. The final concentration of siRNA in each well was adjusted to 20 μmol/L, with 5 μg of Lipofectamine 3000 reagent added per well.

#### CCK-8 assay

Cells were seeded in a 96-well plate at a density of 5 × 10^3^ cells/well and incubated overnight. In the normoxia groups, cells were incubated at 37 °C in a humidified atmosphere with 5% CO_2_ and 21% O_2_. In the hypoxia groups, cells were incubated at 37 °C in a humidified atmosphere with 5% CO_2_ and 1% O_2_. Following 24 h or 48 h incubation, CCK-8 dye (Dojindo Laboratories, Japan) was introduced to each well. The 96-well plates were then placed in a cell culture incubator for 2 h. Absorbance at 450 nm was measured using a microplate reader (Thermo Fisher Scientific, USA). Cell viability was expressed as the relative quantity of viable cells in the treatment group compared to the control.

#### Chromatin immunoprecipitation (ChIP)

Cells were cross-linked with 1% formaldehyde for 10 min at room temperature, and the reaction was quenched with glycine (at a final concentration of 125 mM). Cells were then harvested, lysed, and the chromatin was sheared by ultrasonication to an average fragment size of 200–500 bp.

An aliquot of sheared chromatin was saved as Input DNA (processed in parallel through de-crosslinking and purification) and used as an internal reference to normalize for variations in starting material and PCR efficiency. The remaining sheared chromatin was incubated overnight at 4 °C with protein A/G magnetic beads pre-coated with either 4.8 μg of a specific antibody against TCF4 (13838-1-AP; Proteintech, China) or an equivalent amount of species-matched control IgG (rabbit) as a negative control for background estimation.

Following immunoprecipitation, the beads were washed extensively with low-salt, high-salt, and LiCl wash buffers. Cross-links were reversed by incubation with 200 mM NaCl at 65 °C overnight. After reverse cross-linking, immunoprecipitated DNA was purified using the Pierce Magnetic ChIP Kit (26157; Thermo Fisher Scientific, USA).

To assess enrichment, quantitative real-time PCR (qPCR) was performed with primers specific for target loci (*Serpine1*, *Mmp7*) and for a non-specific genomic region—the inter-translated region between the first and second coding exons of the *Gapdh* gene, which lacks known protein binding sites. This region was included to evaluate background levels; in our experiments, its Ct value was consistently >35, indicating negligible enrichment.

All qPCR primers (for *Serpine1*, *Mmp7*, and the *Gapdh* inter-translated region) were synthesized by IGE Biotechnology (Guangzhou, China), and their sequences are listed in Table S3. Enrichment values were calculated relative to the Input DNA fraction (set as 100%) to determine fold enrichment in the IgG and anti-TCF4 groups, thereby reflecting specific binding rather than general chromatin accessibility or amplification bias.

#### Quantitative real-time PCR

Cell and kidney mRNA extraction was performed using the RNAiso Plus kit (Takara, Japan), followed by cDNA synthesis with the PrimeScript RT Reagent Kit (Takara, Japan). qPCR was conducted using the Hieff qPCR SYBR Green Master Mix (Yeasen, China). Specific primers for each gene were synthesized by IGE Biotechnology (Guangzhou, China), and the sequences are detailed in Table S2.

#### Co-immunoprecipitation (Co-IP)

Protein-protein interactions were analyzed by co-immunoprecipitation (Co-IP) under non-denaturing conditions. Briefly, cells were lysed in a mild IP lysis buffer supplemented with protease inhibitors. After centrifugation to obtain clarified cell lysates, the lysates were divided into three groups: (1) the experimental group, incubated with a mouse monoclonal antibody against Wnt5b (sc-376249; Santa Cruz, USA) or FZD1 (sc-398082; Santa Cruz, USA); (2) the negative control group, incubated with normal mouse IgG (B900620; Proteintech, China); and (3) the input control group. All groups were rotated overnight at 4°C to facilitate antibody-antigen binding. Protein A/G Plus Agarose beads were then added to each group and incubated for an additional 4 h at 4°C to capture the antibody-protein complexes. The beads were collected by centrifugation, washed extensively with ice-cold lysis buffer to remove non-specifically bound proteins, and the bound complexes were eluted by boiling in 2× SDS loading buffer.

For Western blot analysis, the input lysate, immunoprecipitated samples, and IgG control samples were separated by SDS-PAGE and transferred to PVDF membranes. To avoid cross-reactivity from the IP antibodies, the membranes were probed with species-matched secondary antibodies distinct from those used in IP: specifically, Wnt5b was detected using a rabbit polyclonal antibody (bs-8010R; Bioss, China), and FZD1 was detected using another rabbit polyclonal antibody (DF4882; Affinity Biosciences, USA). Signal development was performed using horseradish peroxidase (HRP)-conjugated goat anti-rabbit IgG (secondary antibody). This design ensured that any bands observed in the IP samples (but absent in the mouse IgG control) represented specific interactions between the target proteins.

#### Western blot

We evaluated the following indices through Western blot analysis: the hypoxic markers HIF-1α and Glut1; the epithelial cell markers E-cadherin and ZO-1; the mesenchymal cell markers α-SMA, Vimentin, Collagen type Ⅰ, and Fibronectin; the exosomes markers Alix, TSG101 and CD9; Wnt/β-Catenin signaling pathway-associated proteins WLS, Wnt5b, β-Catenin, non-phospho (active) β-Catenin (Ser33/37/Thr41), FZD1 and LRP6; and the loading controls β-Actin, GAPDH and Lamin B1. Protein samples were separated using 8% or 10% SDS-polyacrylamide gel electrophoresis and subsequently transferred to a PVDF membrane (Millipore, USA) via the electro-transfer method. The membranes were then blocked for 2 h at room temperature with 5% skimmed milk powder in TBS, and then incubated overnight at 4 °C with appropriate primary antibodies. Primary antibodies used were described in the Supplemental Methods S1. After washing three times with TBST, the membranes were incubated with appropriate secondary antibodies for 2 h at room temperature. Finally, the protein levels were normalized by the loading controls. For the exosomal Western blot analysis, however, quantitative analysis was not performed due to the absence of validated exosomal reference proteins. Instead, equal protein loading was applied across lanes to ensure comparability.

Uncropped images of all immunoblots are provided in Data S1.

#### Immunofluorescence staining

Following the treatment, the cells were immobilized using 4% paraformaldehyde at ambient temperature for 15 min. Subsequently, cell membrane permeability was enhanced by incubating the cells in 100% methanol at room temperature for 10 min. To prevent non-specific binding, the cells were then blocked with a solution of 3% BSA in PBS for 0.5 h. Subsequently, the cells were incubated with anti-β-Catenin (8480S; Cell Signaling Technology, USA) at 4°C overnight. Then, the cells were incubated with Alexa Fluor 555 goat anti-rabbit IgG (H + L) (Cell Signaling Technology, USA) at room temperature for 2 h. Nuclei were stained with DAPI at room temperature for 5 min. Finally, images were captured using an Olympus FV1000 Confocal Laser Scanning Microscope (Tokyo, Japan).

#### Dual-luciferase assay

The transcriptional activity of β-Catenin-mediated TCF/LEF was assessed using the TOPFlash reporter plasmid (D2501; Beyotime Biotechnology, China), with its mutant control FOPFlash (D2503; Beyotime Biotechnology, China). The pRL-TK vector was co-transfected as an internal control for normalization. Cells were seeded in 96-well plates and transfected at 60% confluence. Luciferase activity was measured using the Renilla-Firefly Luciferase Dual Assay Kit (RG088S; Beyotime Biotechnology, China) according to the manufacturer’s instructions. Relative luciferase activity was normalized to Renilla luciferase activity and expressed as fold-change relative to control groups.

#### Isolation of exosomes

Exosomes were isolated from cell culture media using three centrifugation steps at 4°C: (1) centrifugation at 500 × g for 15 min to eliminate cells, (2) centrifugation at 10,000 × g for 30 min to remove cell debris, and (3) ultracentrifugation at 110,000 × g for 70 min to pellet the exosomes. The pellet was then resuspended in PBS and subjected to centrifugation at 110,000 × g for an additional 70 min to remove soluble and secreted proteins.

Exosomes were isolated from serum using ExoQuick Exosome Precipitation Solution (EXOQ5A-1; SBI System Biosciences, USA) according to the manufacturer’s specified protocol.

### Quantification and statistical analysis

Data were analyzed with SPSS 23.0 (IBM, USA) and are expressed as mean ± standard deviation (SD). Statistical significance between two groups or among more than two groups was assessed using Student’s *t* test or one-way ANOVA followed by Tukey’s HSD test, respectively. A significance level of α = 0.05 was applied. The *n* values in this study represent biological replicates. For each graph, the exact *n* value, the specific statistical test applied, and the precise *p* value are all detailed in the corresponding figure legends. Graphs were created using GraphPad Prism 6 (GraphPad Software, USA).

## Supplemental information

Document S1. Figures S1–S4, Tables S1 and S3, Data S1, and Methods S1

Table S2. Primers used for qPCR in this study
