# Supplementary material for: Wnt5b/FZD1/LRP6 signaling drives renal fibrosis by triggering cytoplasmic stabilization and nuclear translocation of β-catenin under hypoxia
Source: iScience. 2026 May 28;29(6):116129. doi: 10.1016/j.isci.2026.116129 (PMC13233589; doi:10.1016/j.isci.2026.116129)
Supplement: Document S1. Figures S1–S4, Tables S1 and S3, Data S1, and Methods S1 [file mmc1.pdf]

iScience, Volume *29*

Supplemental information

Wnt5b/FZD1/LRP6 signaling drives renal fibrosis by triggering cytoplasmic stabilization and nuclear translocation of β-catenin under hypoxia

Zhibin Wu, Zheng Kuang, Jia Liu, Guanghui Dong, Lixia Liang, Shuang Ma, Fei Zou, and Guanghai Wang

Figure S1

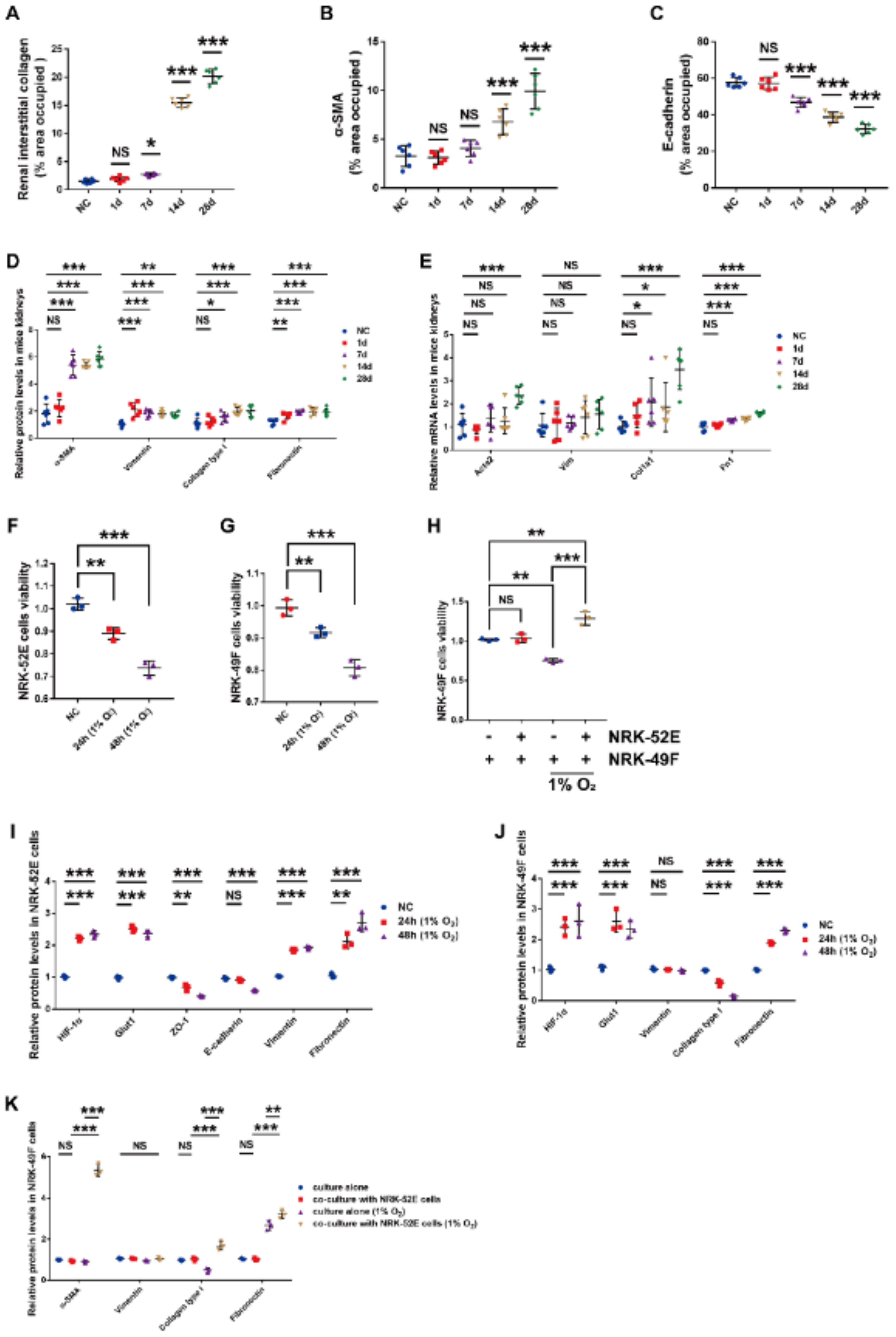

Figure S1. High-altitude hypoxia caused renal fibrosis. (A) Masson's trichrome staining of kidney sections (n=6). (B-C) Immunohistochemistry of high-altitude hypoxia-induced changes of α-SMA and E-cadherin in mice kidneys (n=6). (D) Western blot analysis of high-altitude hypoxia-induced alterations of α-SMA, Vimentin, Collagen type I and Fibronectin in mice kidneys (n=6). (E) qPCR analysis of high-altitude hypoxia-induced changes of *Acta2*, *Vim*, *Col1a1* and *Fn1* in mice kidneys (n=6). (F-H) CCK-8 assay showed the viability of NRK-52E and NRK-49F cells under hypoxic conditions (n=3). (I) Western blot analysis of hypoxia-induced alterations of hypoxia markers and EMT-related proteins in NRK-52E cells (n=3). (J) Western blot analysis of hypoxia-induced alterations of hypoxia markers and fibroblast activation-related proteins in NRK-49F cells (n=3). (K) Western blot analysis revealed the alterations of fibroblast activation-related proteins in NRK-49F cells when co-cultured with NRK-52E cells under hypoxic

conditions (n=3). Data were shown as mean ± SD. One-way ANOVA was used to determine the significant differences. $\alpha=0.05$, *$P<0.05$, **$P<0.01$, ***$P<0.001$.

Figure S2

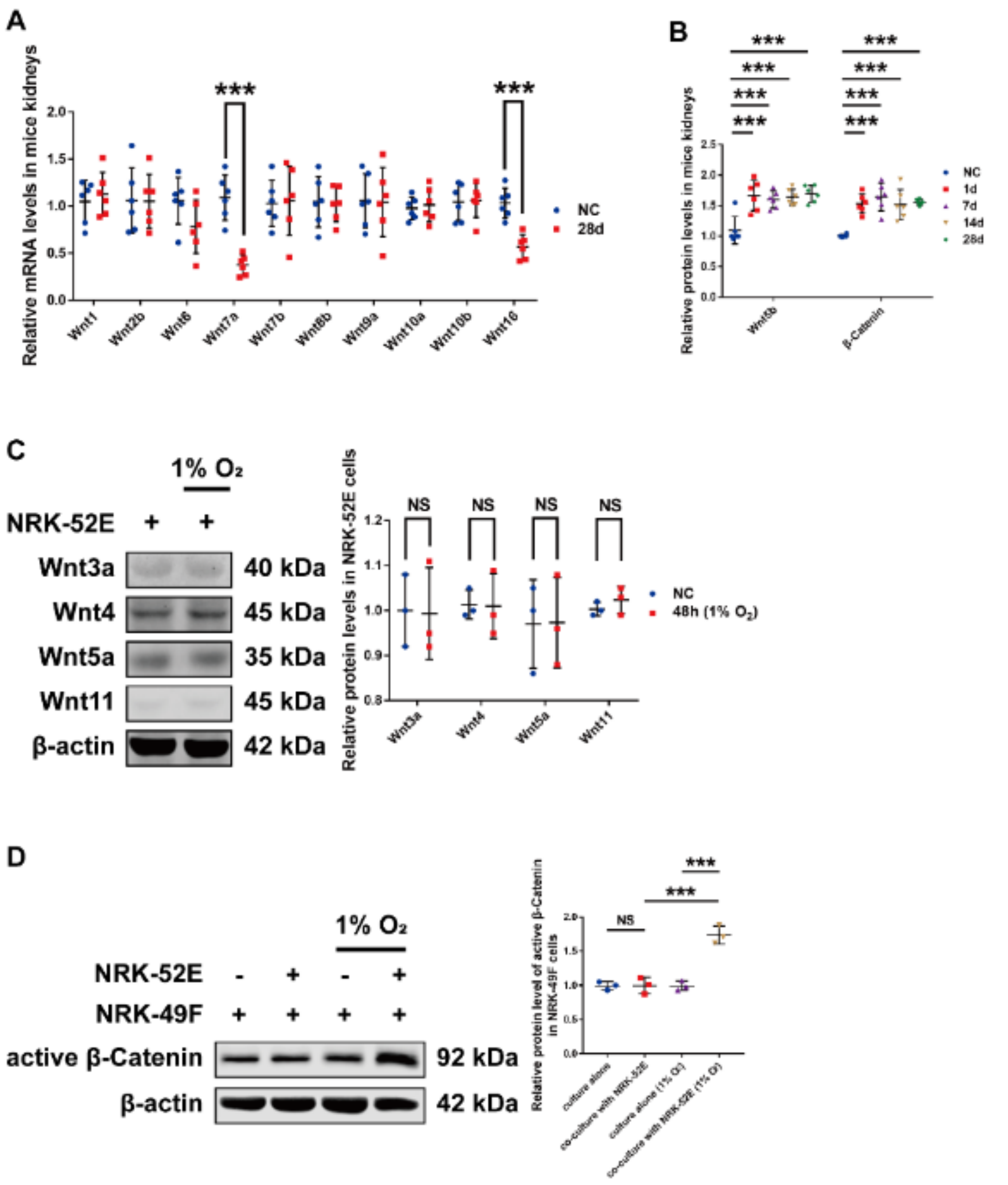

Figure S2. Wnt5b/β-Catenin was activated in hypoxia-induced renal fibrosis. (A) qPCR analysis showed mRNA levels of 10 Wnt ligands in mice kidneys (n=6). (B) Western blot analysis of high-altitude hypoxia-induced alterations of Wnt5b and β-Catenin in mice kidneys (n=6). (C) Western blot analysis of the expression of Wnt3a, Wnt4, Wnt5a, and Wnt11 in NRK-52E cells under hypoxic conditions (n=3). (D) Western blot analysis revealed the alterations of active β-Catenin in NRK-49F cells when co-cultured with NRK-52E cells under hypoxic conditions (n=3). Student's t-test or one-way ANOVA was used to determine the significant differences. α=0.05, $^{*}P<0.05$, $^{**}P<0.01$, $^{***}P<0.001$.

Figure S3

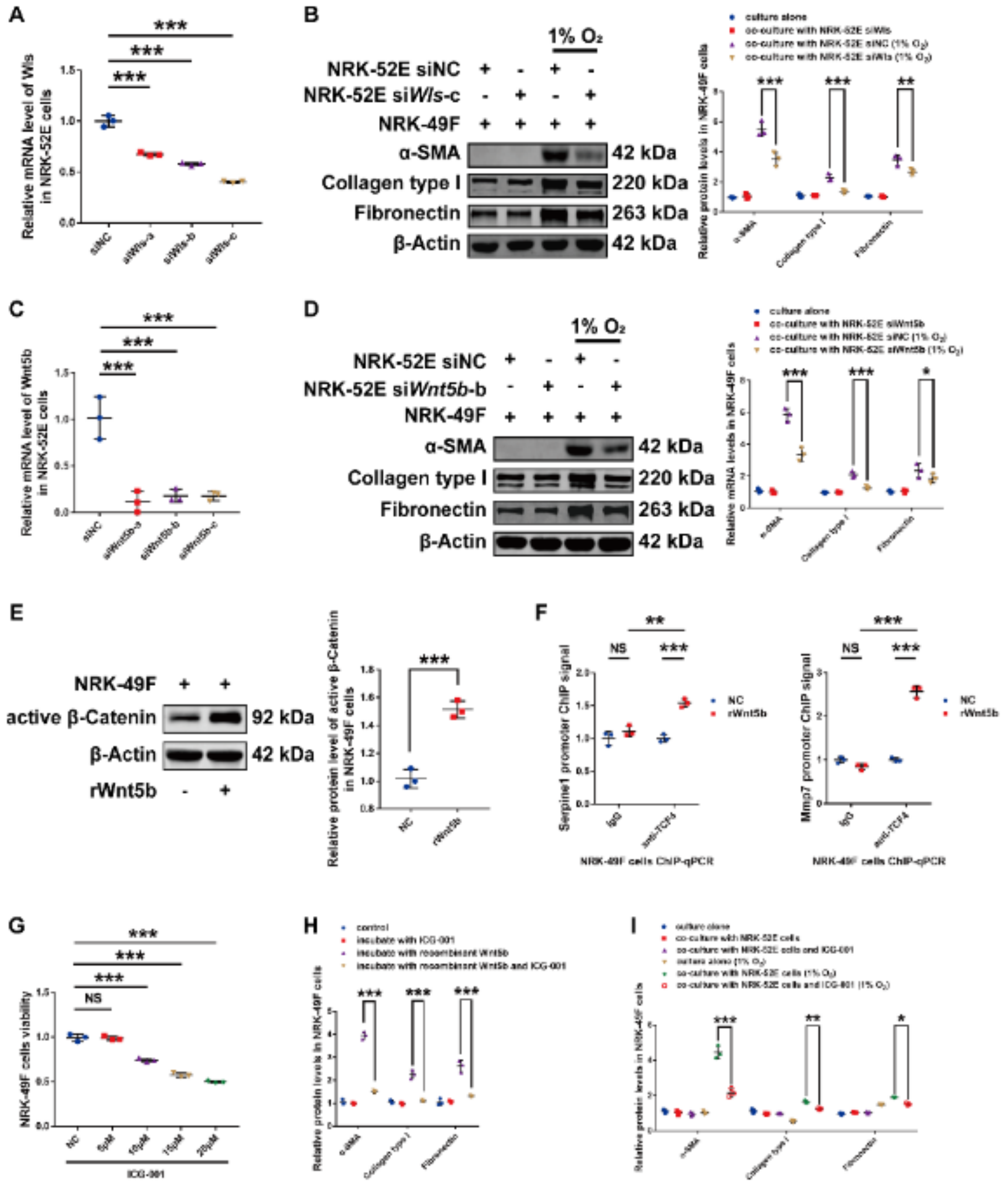

Figure S3. Wnt5b/β-Catenin promoted high-altitude hypoxia-induced renal fibrosis. (A) qPCR analysis showed the silencing effect of siRNAs on *Wls* expression in NRK-52E cells. (B) Western blot analysis revealed the alterations of fibroblast activation-related proteins in NRK-49F cells when co-cultured with NRK-52E cells in which *Wls* was silenced by siRNA, under hypoxic conditions. (C) qPCR analysis showed the silencing effect of siRNAs on *Wnt5b* expression in NRK-52E cells. (D) Western blot analysis revealed the alterations of fibroblast activation-related proteins in NRK-49F cells when co-cultured with NRK-52E cells in which *Wnt5b* was silenced by siRNA, under hypoxic conditions. (E) Western blot analysis revealed the alterations of active β-Catenin in NRK-49F cells induced by recombinant Wnt5b. (F) ChIP-qPCR showed the enrichment of TCF4 binding on the *Serpine1* and *Mmp7* promoters in NRK-49F cells following treatment with 10ng/mL recombinant Wnt5b protein. (G) CCK-8 assay showed the viability of NRK-49F cells treated with different concentrations of ICG-001. (H) Western blot analysis revealed the effect of 10μM ICG-001 on Wnt5b-induced elevation of fibroblast activation-related proteins in NRK-49F cells. (I) Western blot analysis revealed the effect of 10μM ICG-001 on the elevation of fibroblast activation-related proteins in NRK-49F cells when co-cultured with NRK-52E cells under hypoxic conditions. A working concentration of 20μmol/L was applied for the siRNA transfection. Data were shown as mean ± SD. One-way ANOVA was used to determine the significant differences. n=3, α=0.05, *P<0.05, **P<0.01, ***P<0.001.

Figure S4

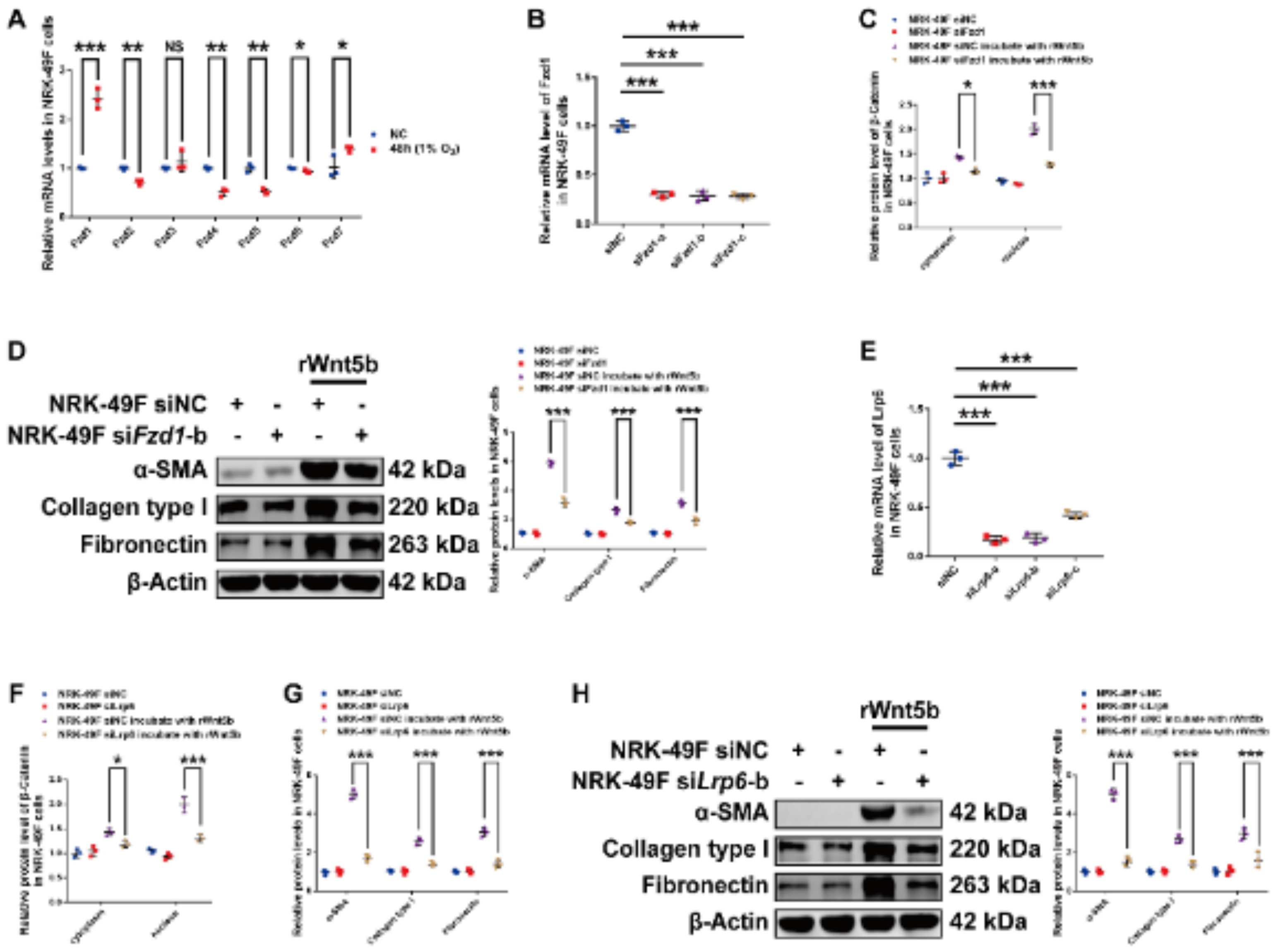

Figure S4. Wnt5b activated β-catenin via FZD1/LRP6, leading to fibroblast activation. (A) qPCR analysis showed alterations in *Fzd*s levels in NRK-49F cells under hypoxic conditions. (B) qPCR analysis showed the silencing effect of siRNAs on *Fzd1* expression in NRK-49F cells. (C) Western blot analysis revealed *Fzd1* knockdown significantly altered β-Catenin levels in both cytoplasm and nucleus of Wnt5b-treated NRK-49F cells. (D) Western blot analysis revealed *Fzd1* knockdown significantly altered the expression of fibroblast activation-related proteins in Wnt5b-treated NRK-49F cells. (E) qPCR analysis showed the silencing effect of siRNAs on *Lrp6* expression in NRK-49F cells. (F) Western blot analysis revealed *Lrp6* knockdown significantly altered β-Catenin levels in both cytoplasm and nucleus of Wnt5b-treated NRK-49F cells. (G-H) Western blot analysis revealed *Lrp6* knockdown significantly altered the expression of fibroblast activation-related proteins in Wnt5b-treated NRK-49F cells. A working concentration of 20μmol/L was applied for the siRNA transfection. Data were shown as mean ± SD. One-way ANOVA was used to determine the significant differences. n=3, α=0.05, *P<0.05, **P<0.01, ***P<0.001.

Table S1

Table S1. siRNA sequences used in this study

**Name**	**Sequence 5' to 3'**	
	**sense strand**	**antisense strand**
siNC	UUCUCCGAACGUGUCACGUdTdT	ACGUGACACGUUCGGAGAAdTdT
si*Wls*-a	GCUGUUUAUCCUGCAGAUAdTdT	UAUCUGCAGGAUAAACAGCdTdT
si*Wls*-b	CCUUCCUUUCAUGGAAAUUdTdT	AAUUUCCAUGAAAGGAAGGdTdT
si*Wls*-c	CCAGCAUCUUCAUCAUUAUdTdT	AUAAUGAUGAAGAUGCUGGdTdT
si*Wnt5b*-a	GGAGGACCUGGUCUACGUAGAdTdT	UCUACGUAGACCAGGUCCUCCdTdT
si*Wnt5b*-b	GGCUGAUGUCUCCUGCAAAUGdTdT	CAUUUGCAGGAGACAUCAGCCdTdT
si*Wnt5b*-c	GCGGCUAUGACCGCUUCAAGAdTdT	UCUUGAAGCGGUCAUAGCCGCdTdT
si*Fzd1*-a	GCAGCAGUACAACGGCGAAtt	UUCGCCGUUGUACUGCUGCtt

Continued Table S1. siRNA sequences used in this study

si*Fzd1*-b	GCACGGACAUCGCGUACAAtt	UUGUACGCGAUGUCCGUGCtt
si*Fzd1*-c	GCGAGGCACUCAUGAACAAtt	UUGUUCAUGAGUGCCUCGCtt
si*Lrp6*-a	GGUUCAAGUCGCUUUGUUAtt	UAACAAAGCGACUUGAACCtt
si*Lrp6*-b	CGACAUUGUCCUACAGUUAtt	UAACUGUAGGACAAUGUCGtt
si*Lrp6*-c	GUCGCUUUGUUAUAAUAAAtt	UUUAUUAUAACAAAGCGACtt

Table S3

Table S3. Primers used for ChIP-qPCR in this study

**Gene**	**Primer Sequence 5' to 3'**	
	**Forward**	**Reverse**
Serpine1-Rat	CTTTTCTAGTCCAAGCCCTC	TTAAGAGGACCAGGCTGTGT
Mmp7-Rat	TCAGACAGGAGGCTGTGAGA	GGAGCACTGCTGGTGTTTCA
Gapdh inter-translated region-Rat	TGGATAGGGTGGCCGAAGTA	AAAGGCGGAGTTACAAGGGG

Data S1. Uncropped blots for all figures in this study.

Full unedited blot for Figure 1L

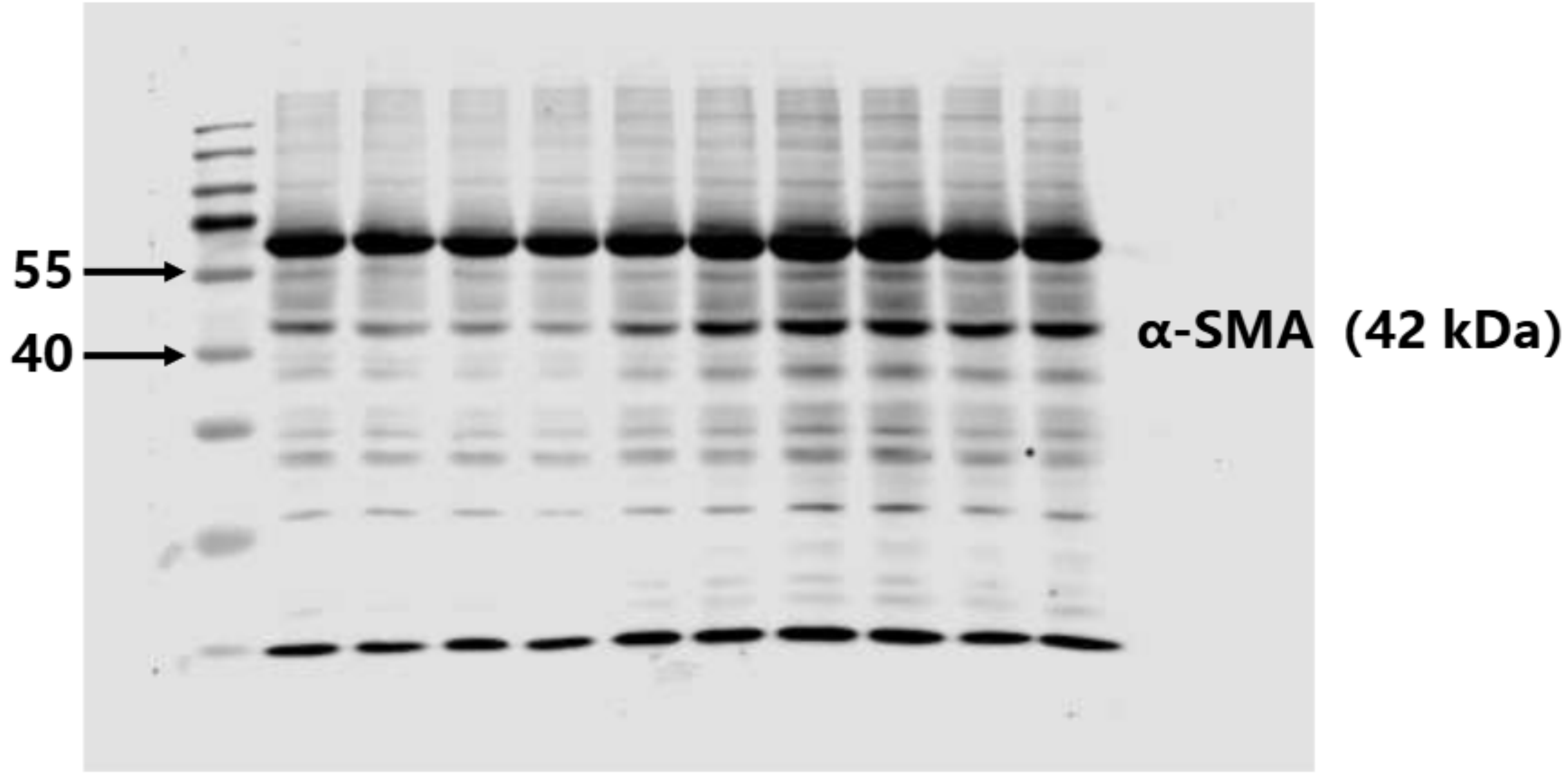

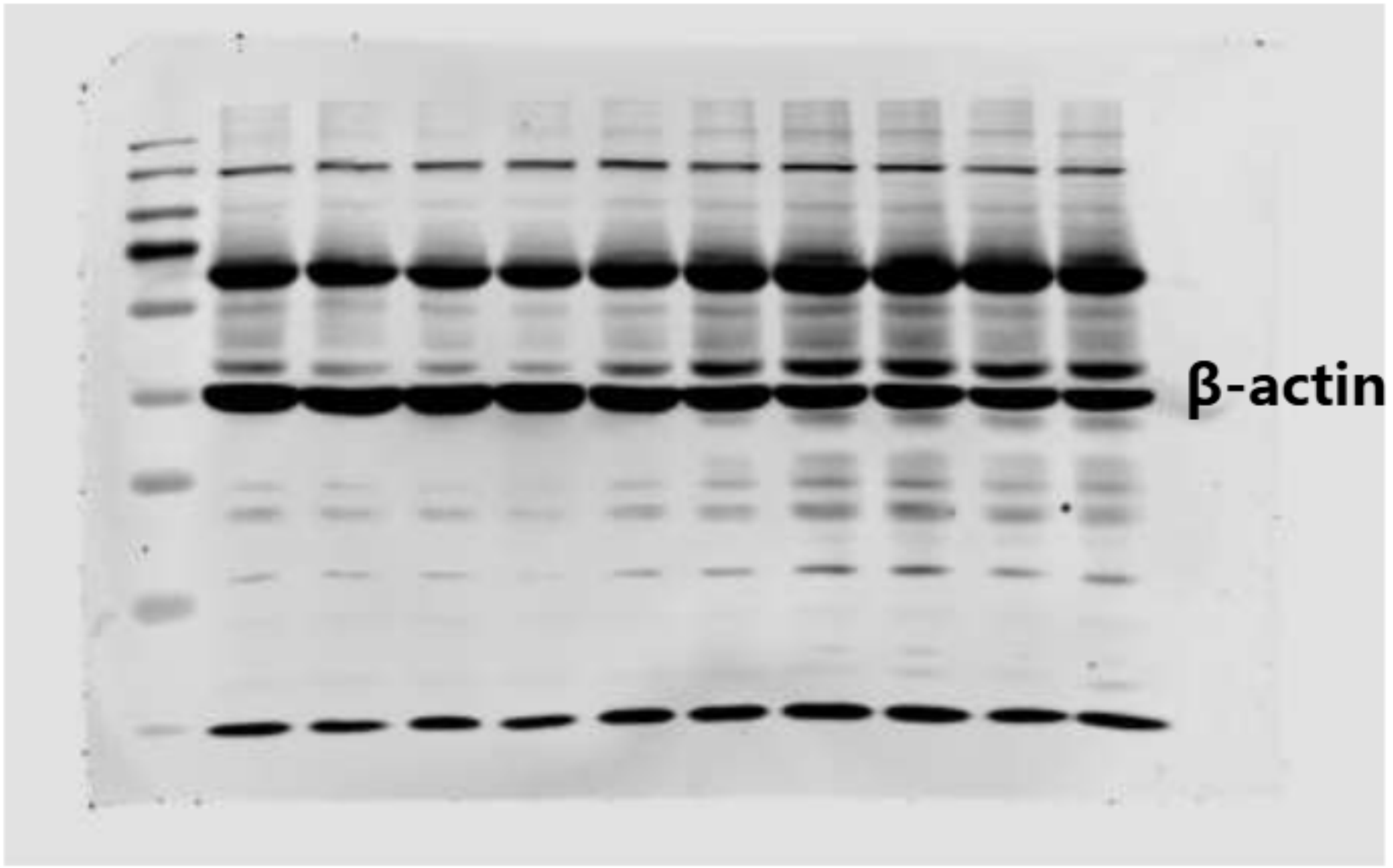

Lanes 2-11 of the unedited blot correspond to those shown in the cropped images within the manuscript.

Full unedited blot for Figure 1L

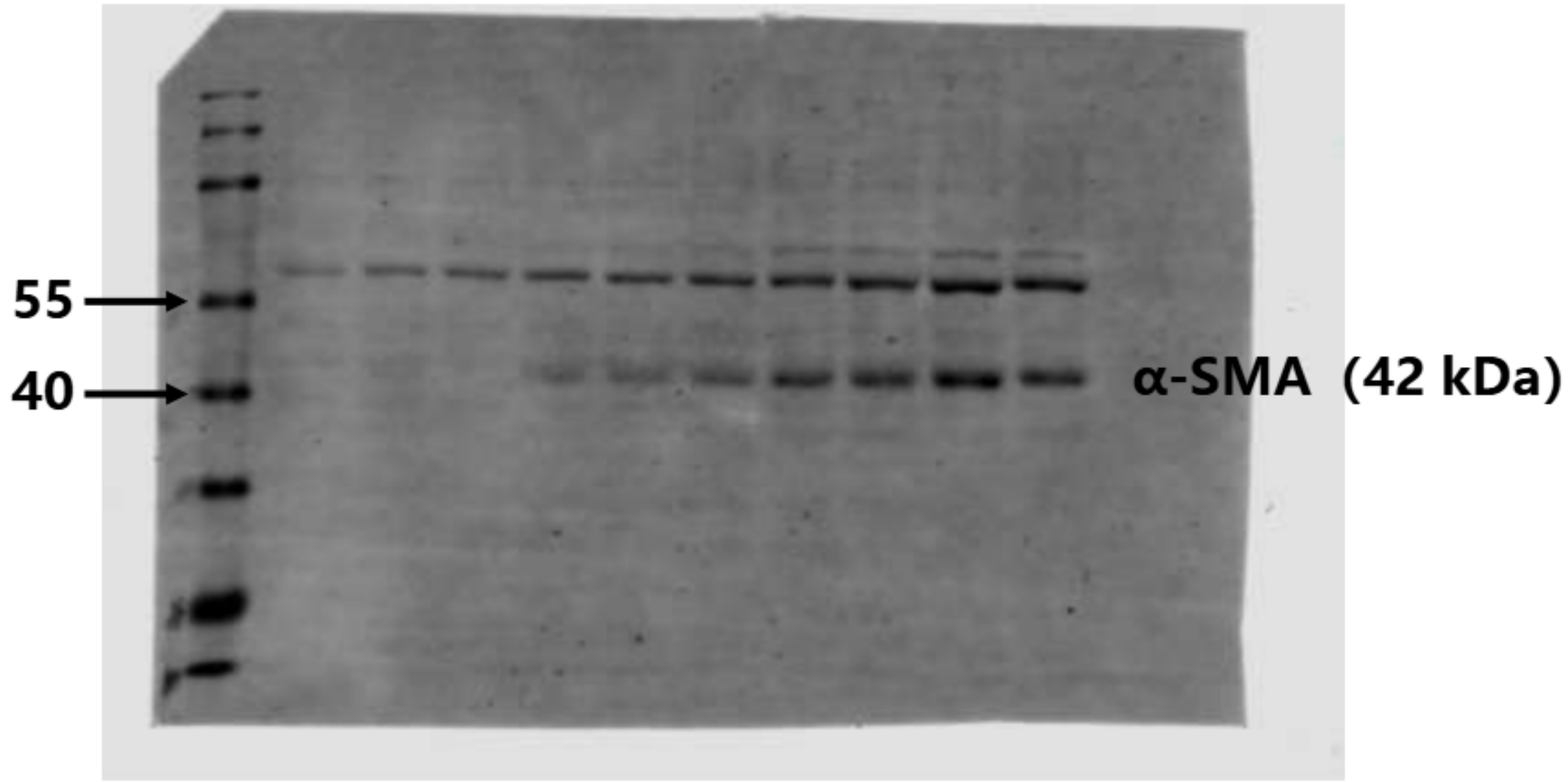

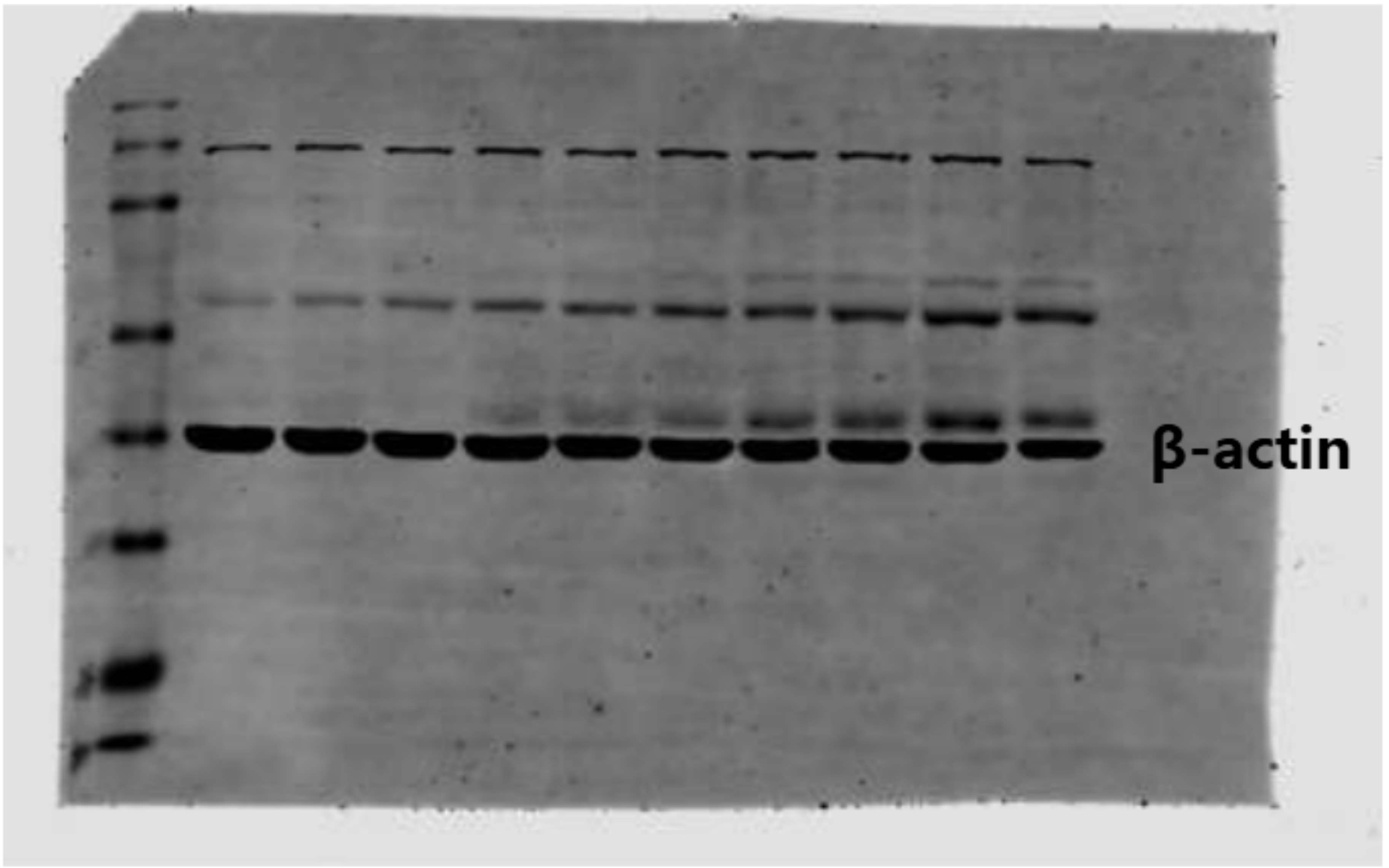

Full unedited blot for Figure 1L

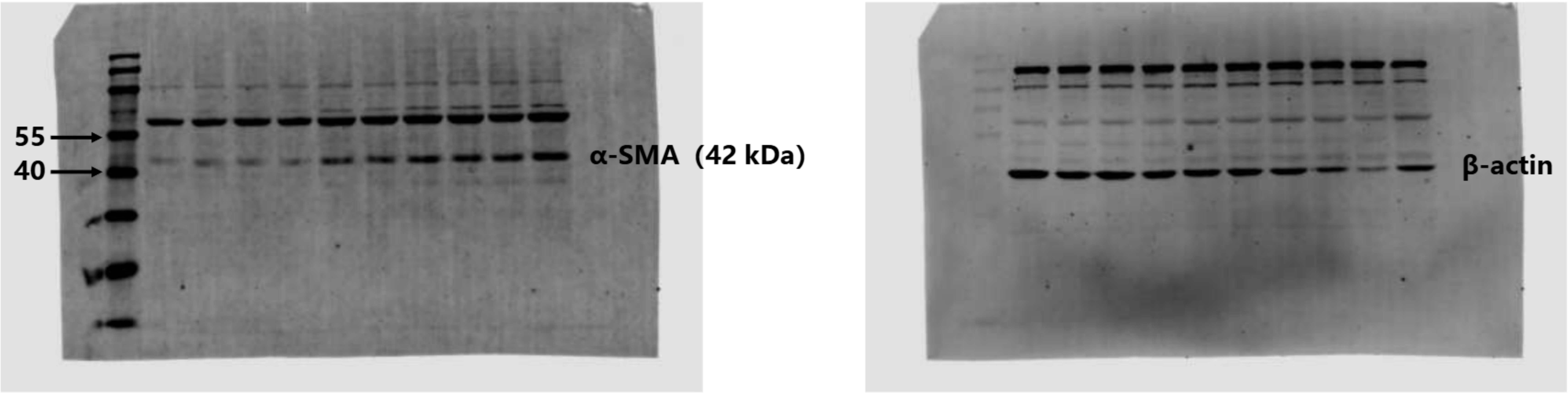

Full unedited blot for Figure 1L

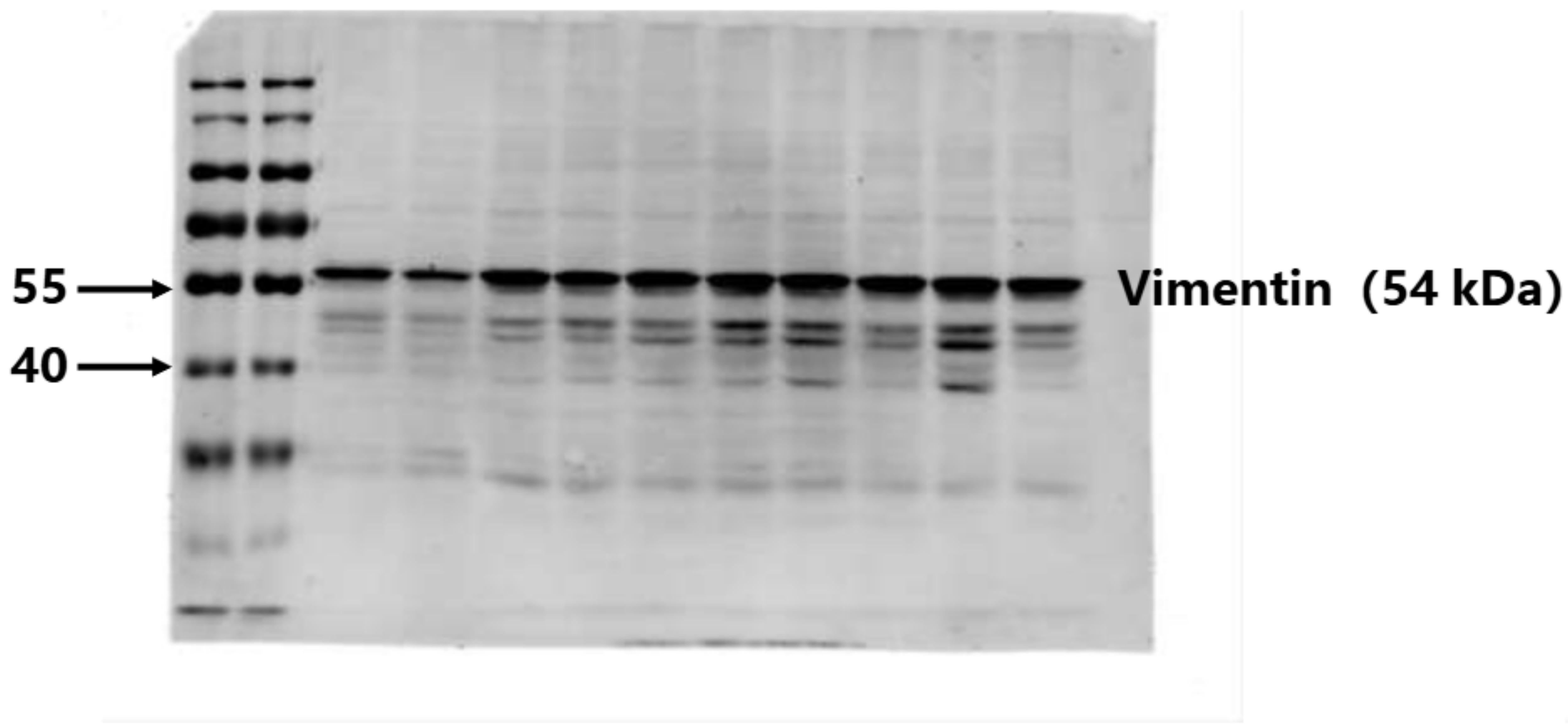

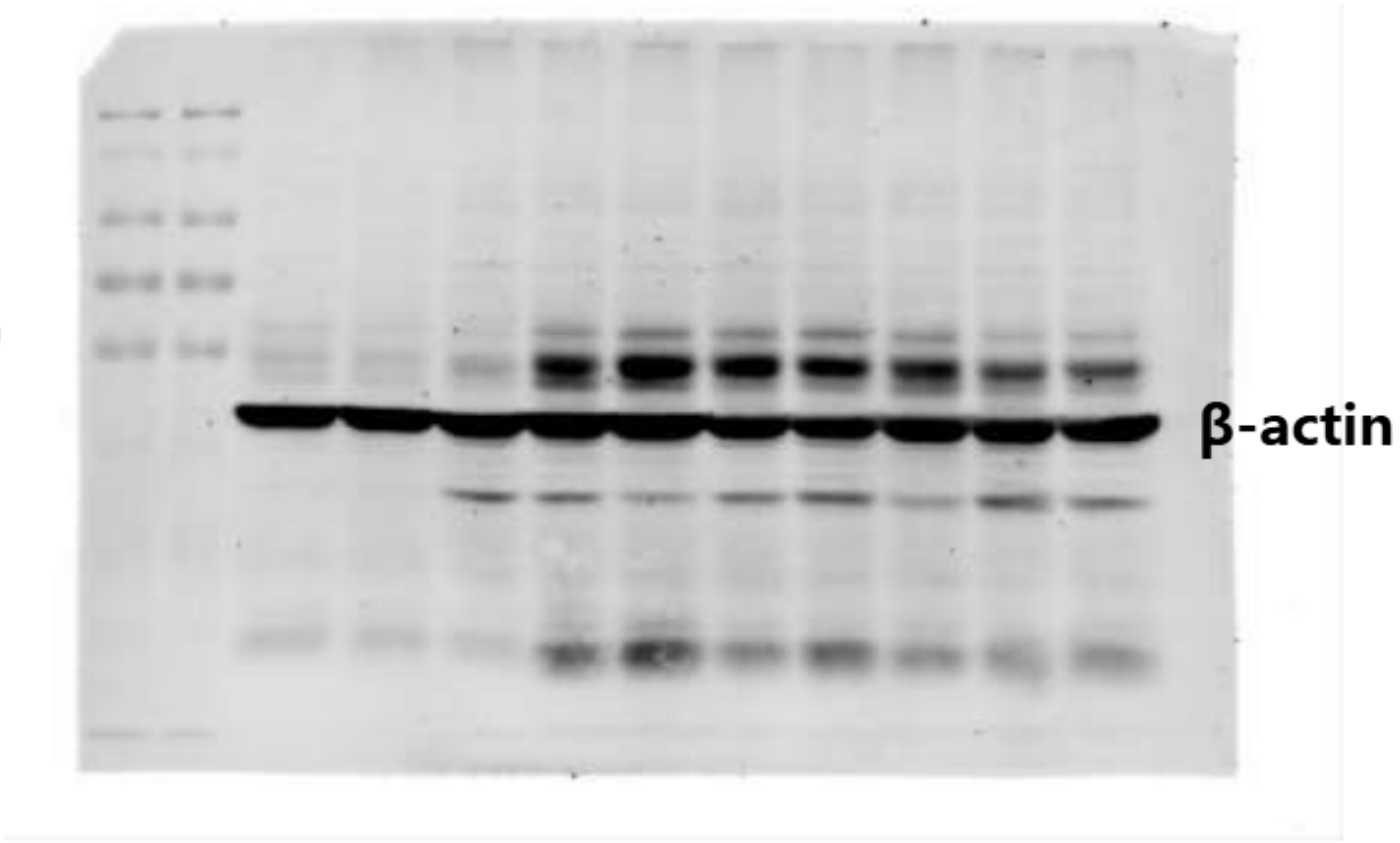

Lanes 3-12 of the unedited blot correspond to those shown in the cropped images within the manuscript.

Full unedited blot for Figure 1L

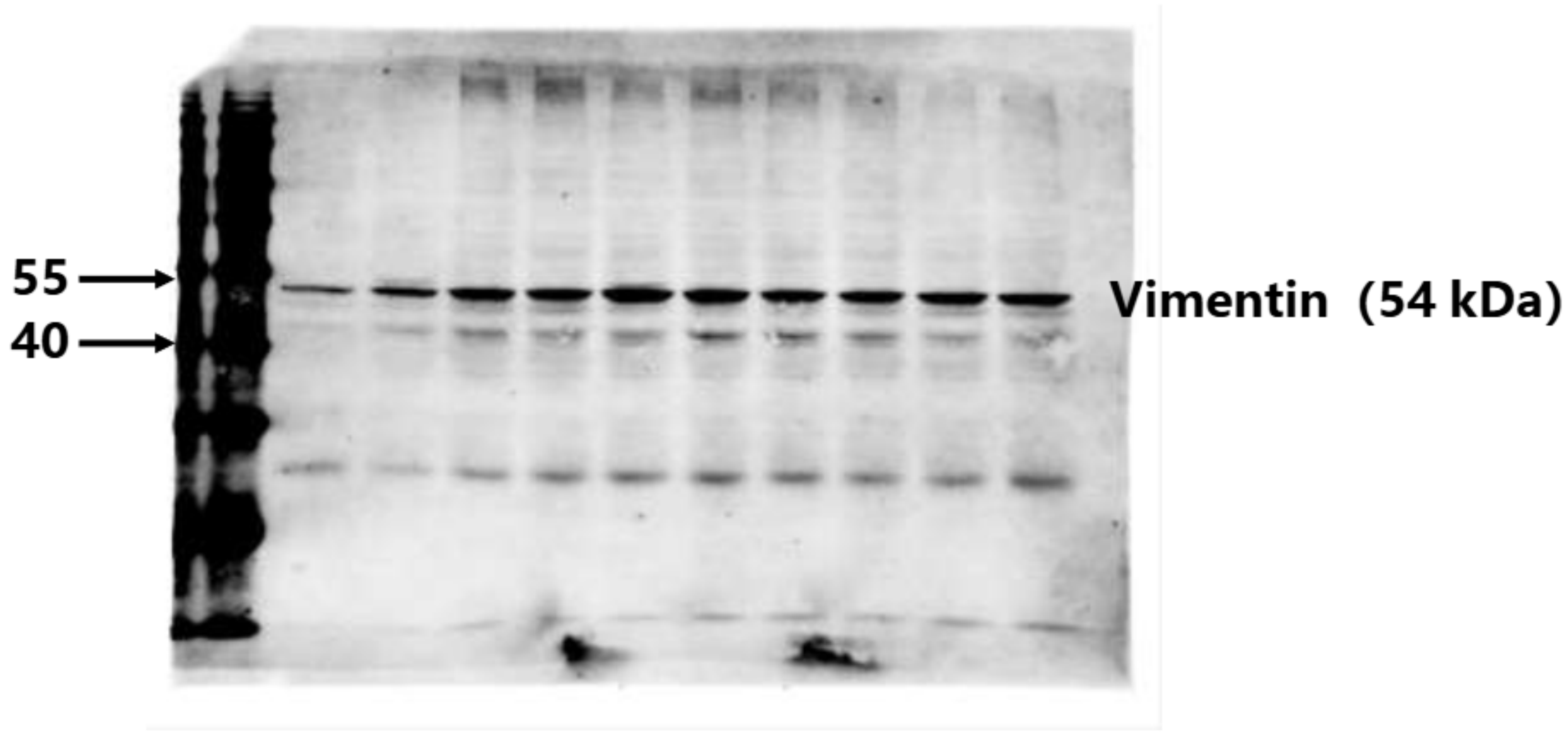

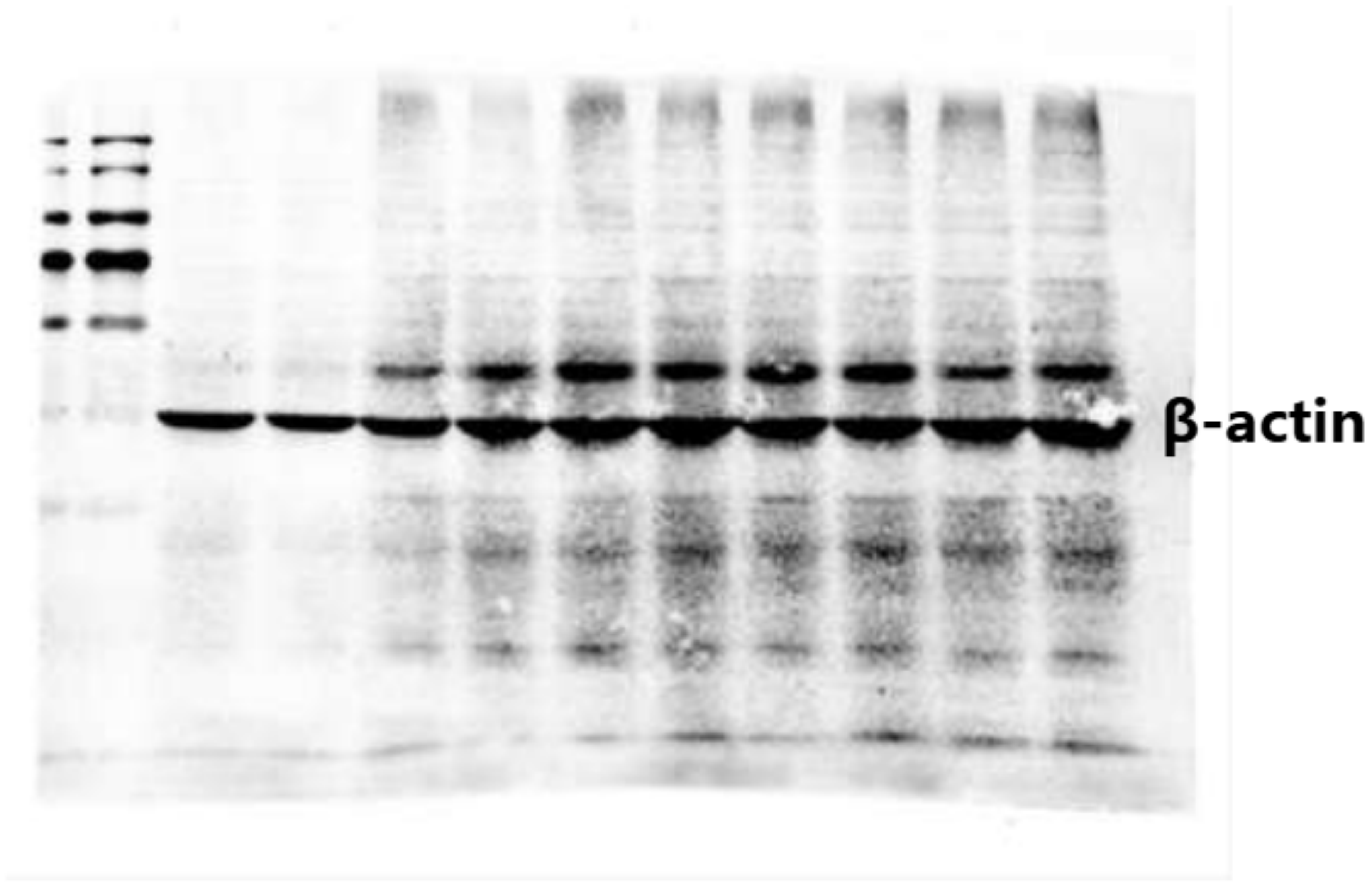

Full unedited blot for Figure 1L

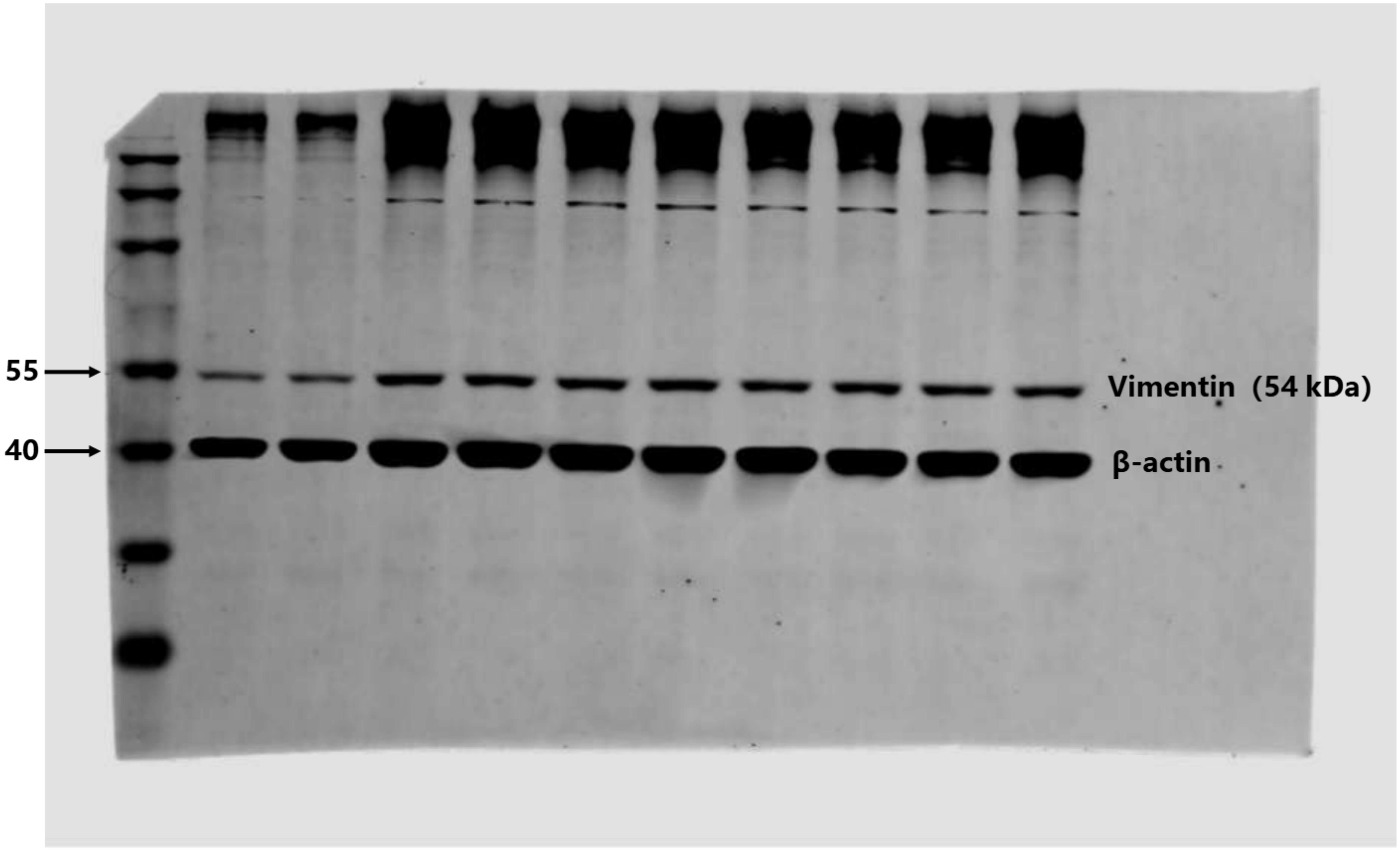

Full unedited blot for Figure 1L

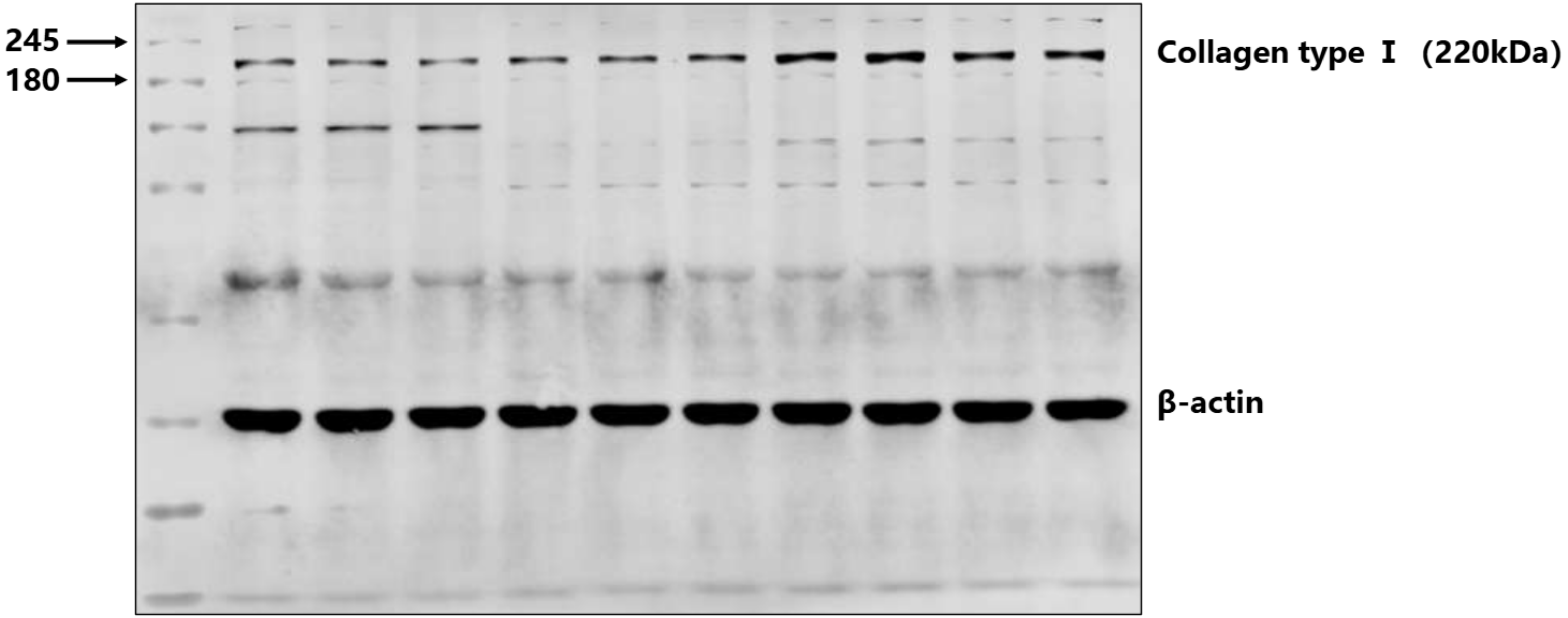

Lanes 2-11 of the unedited blot correspond to those shown in the cropped images within the manuscript.

Full unedited blot for Figure 1L

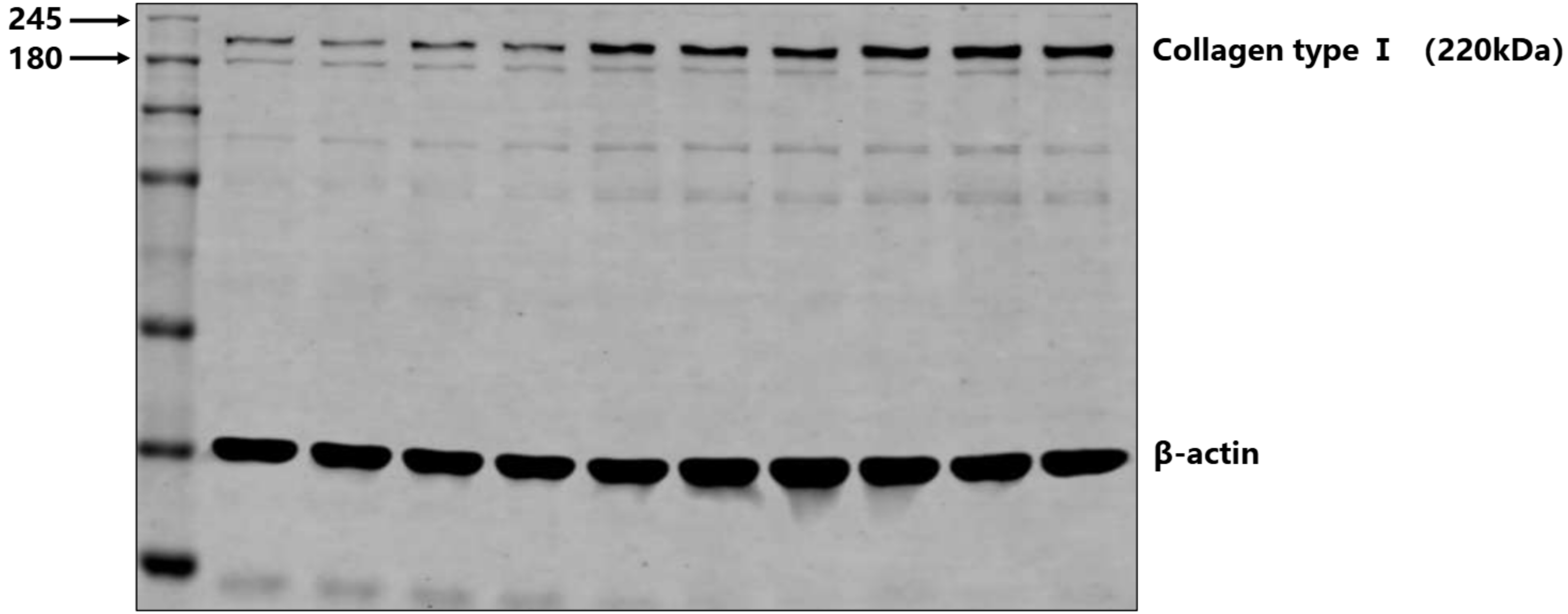

Full unedited blot for Figure 1L

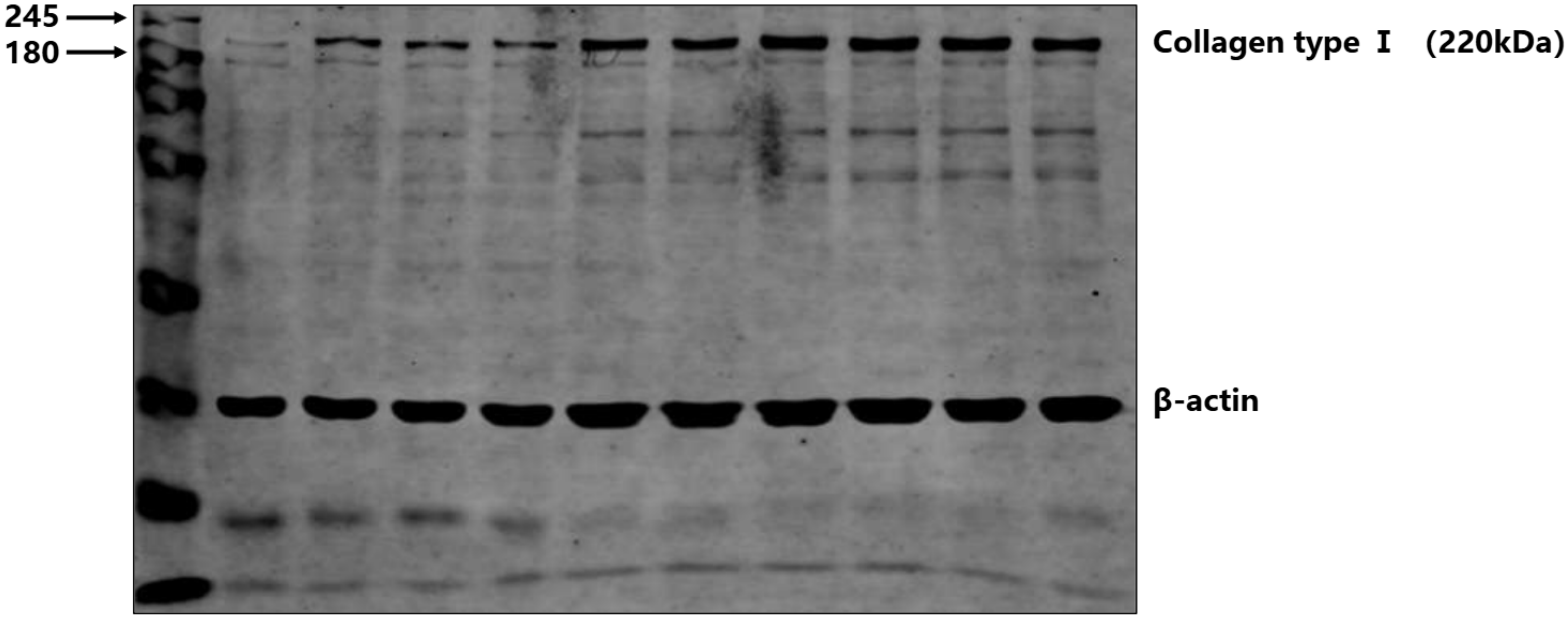

Full unedited blot for Figure 1L

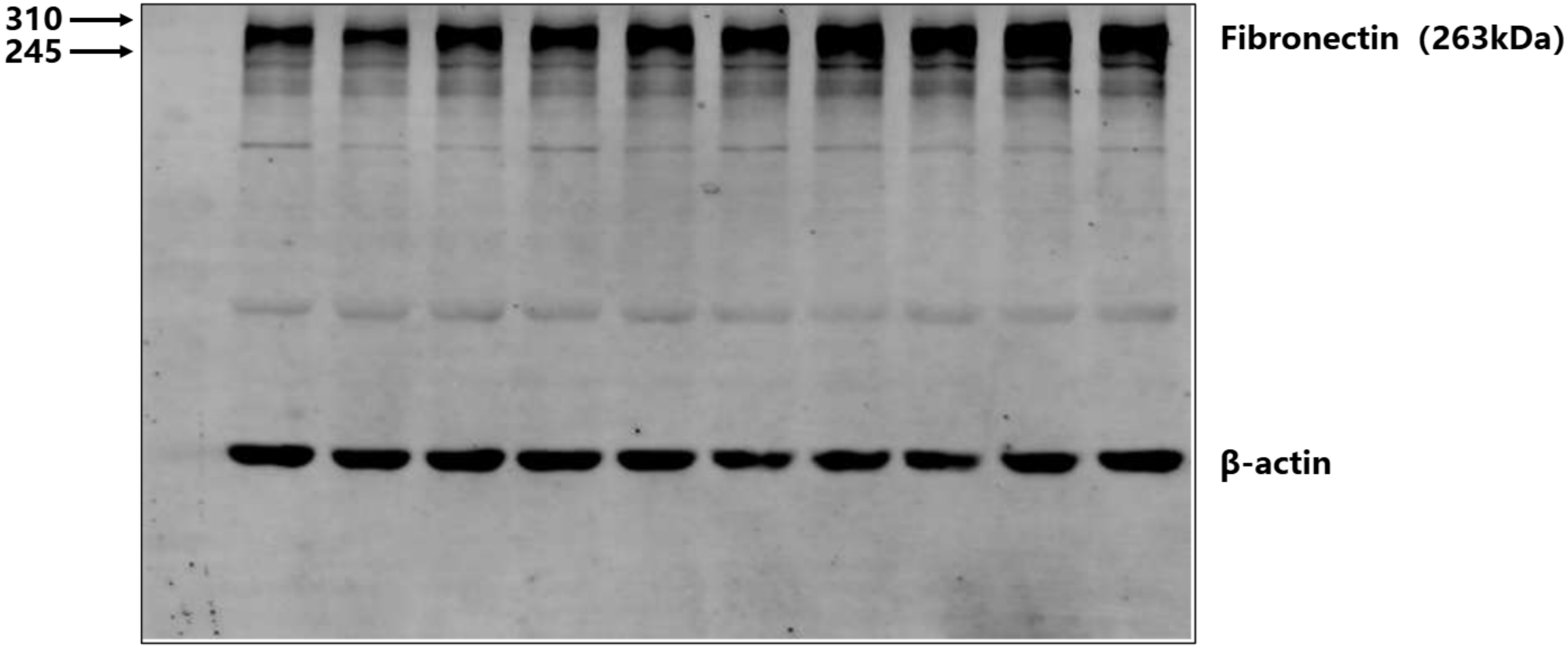

Lanes 2-11 of the unedited blot correspond to those shown in the cropped images within the manuscript.

Full unedited blot for Figure 1L

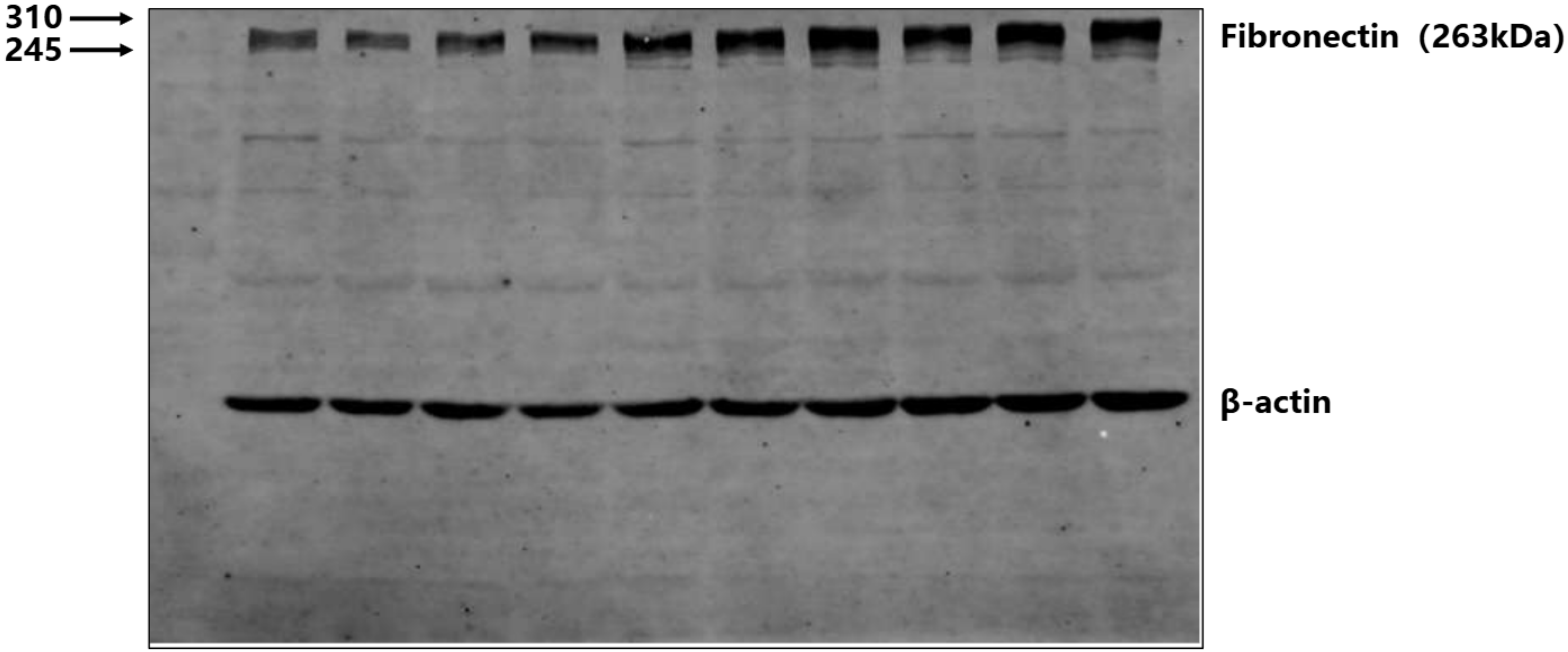

Full unedited blot for Figure 1L

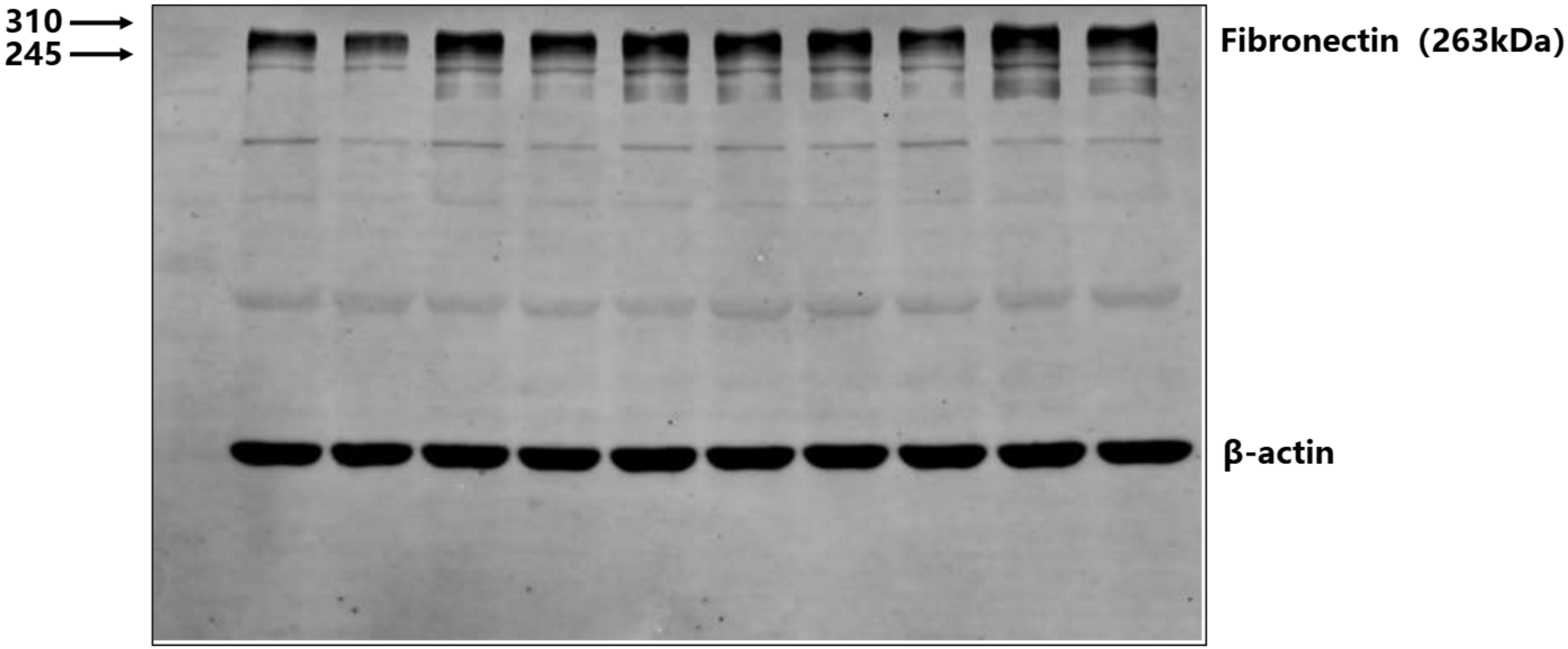

Full unedited blot for Figure 1M

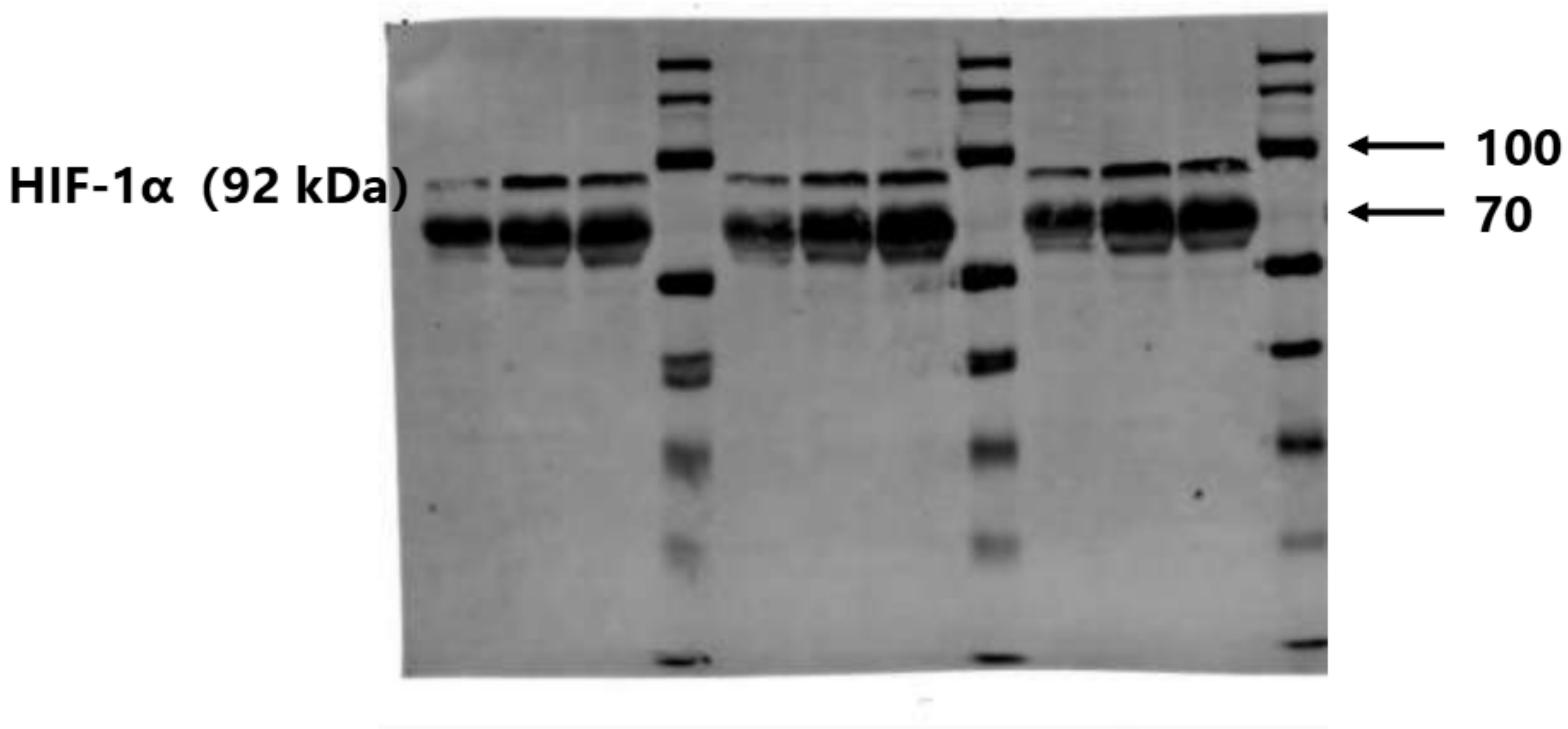

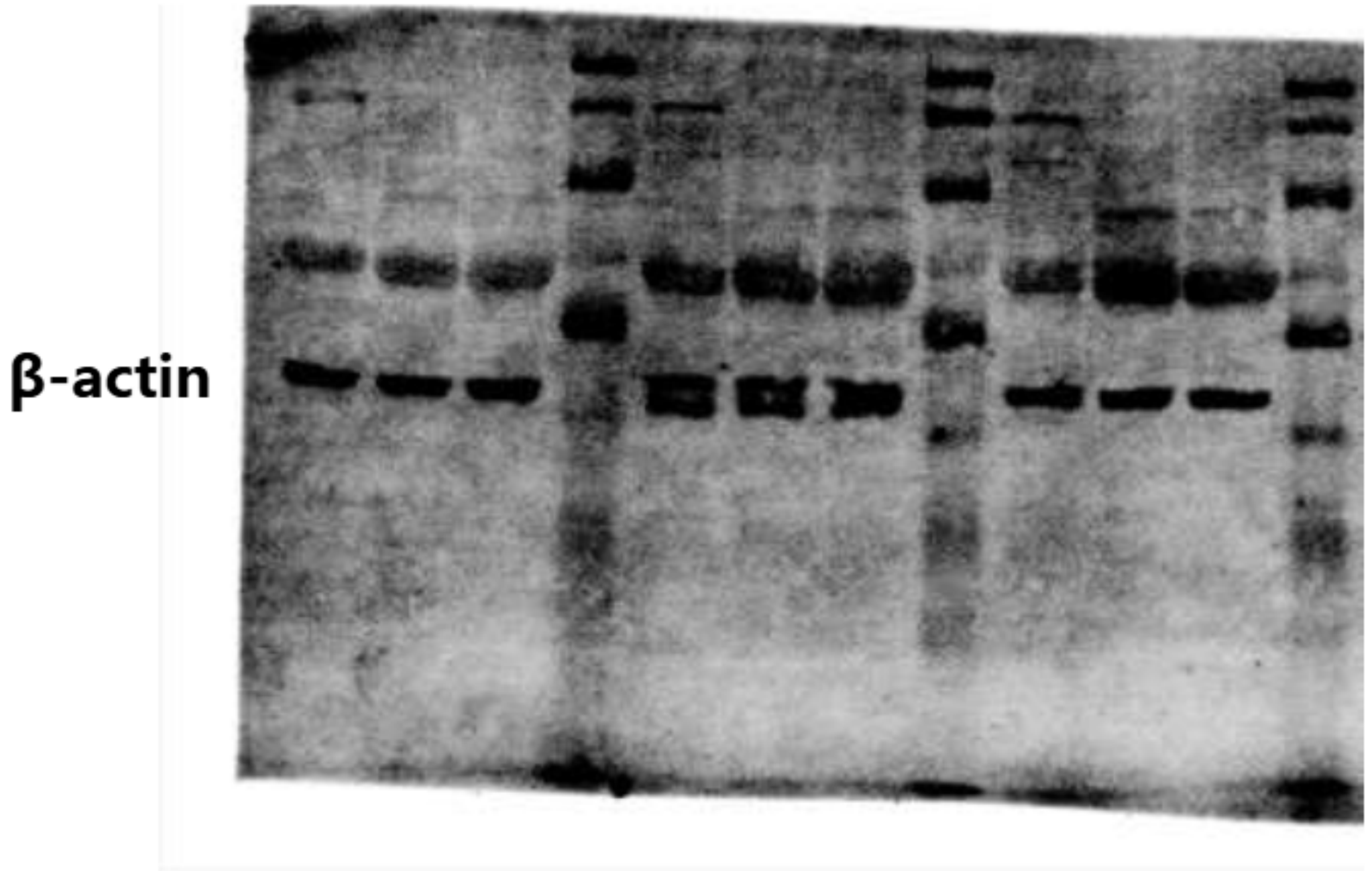

Lanes 5-7 of the unedited blot correspond to those shown in the cropped images within the manuscript.

Full unedited blot for Figure 1M

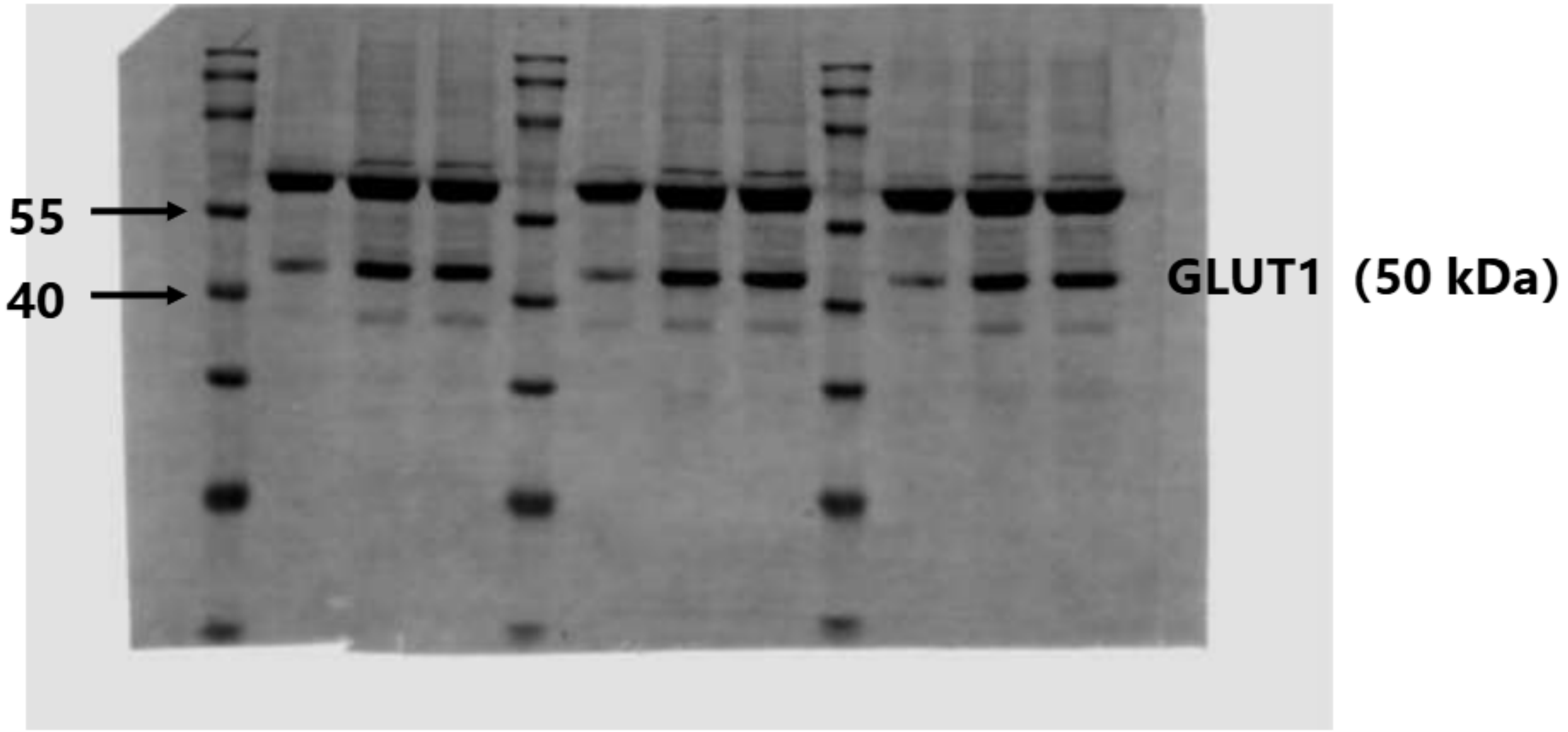

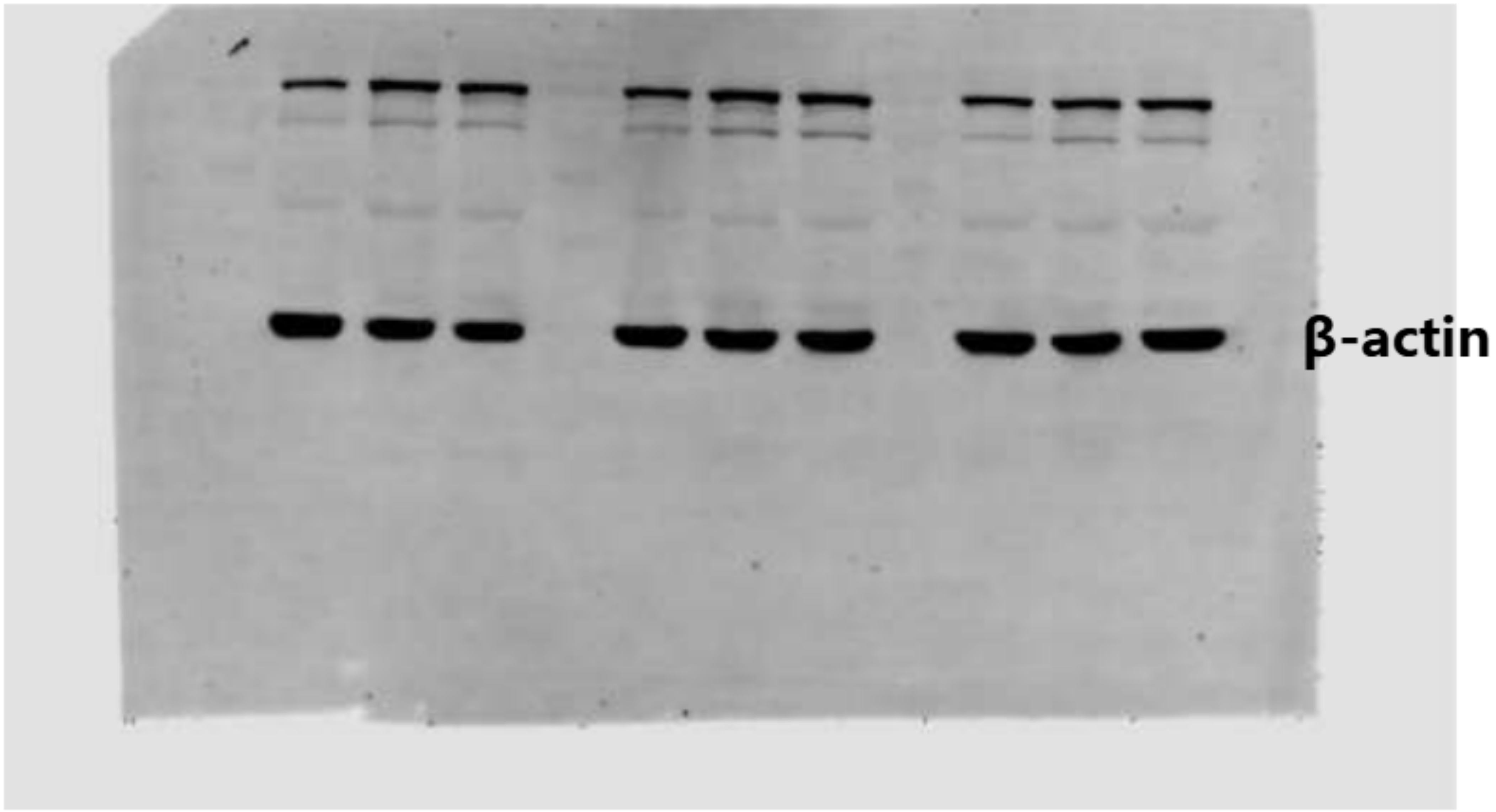

Lanes 6-8 of the unedited blot correspond to those shown in the cropped images within the manuscript.

Full unedited blot for Figure 1M

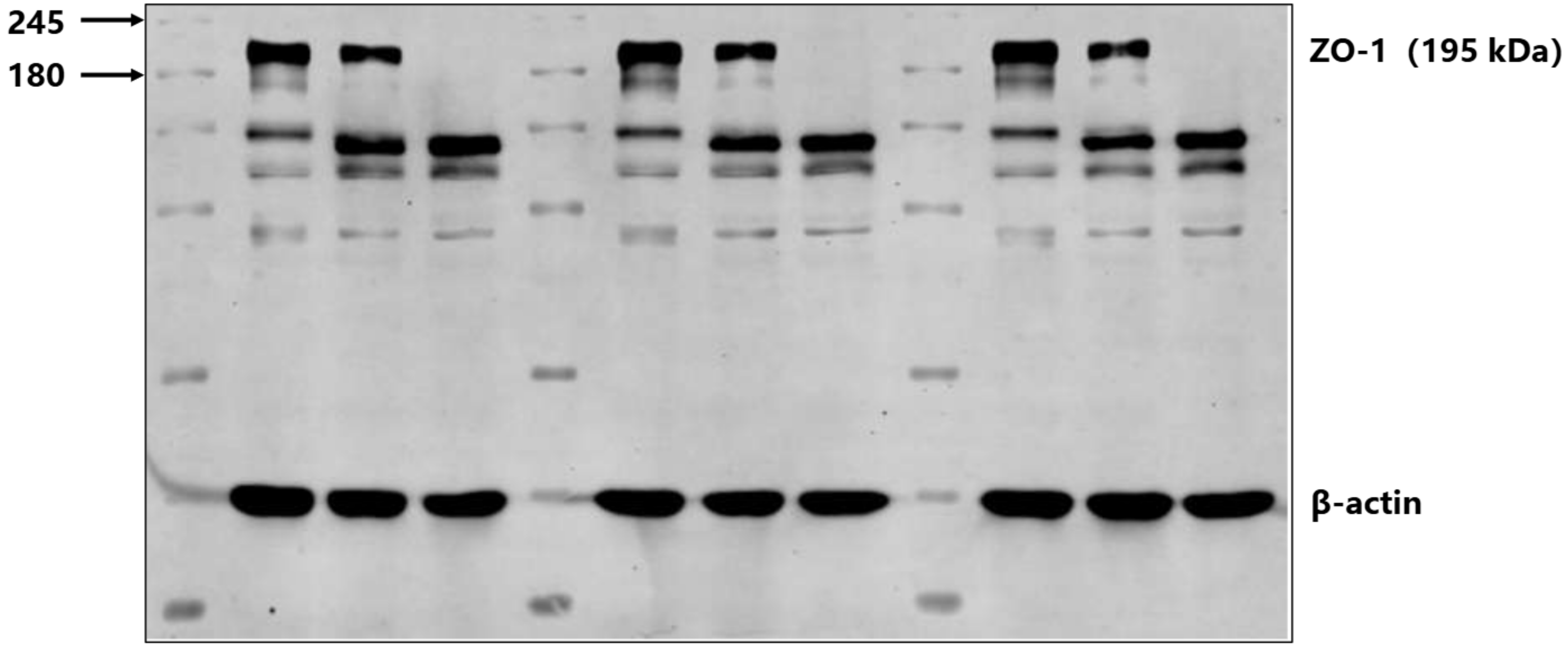

Lanes 2-4 of the unedited blot correspond to those shown in the cropped images within the manuscript.

Full unedited blot for Figure 1M

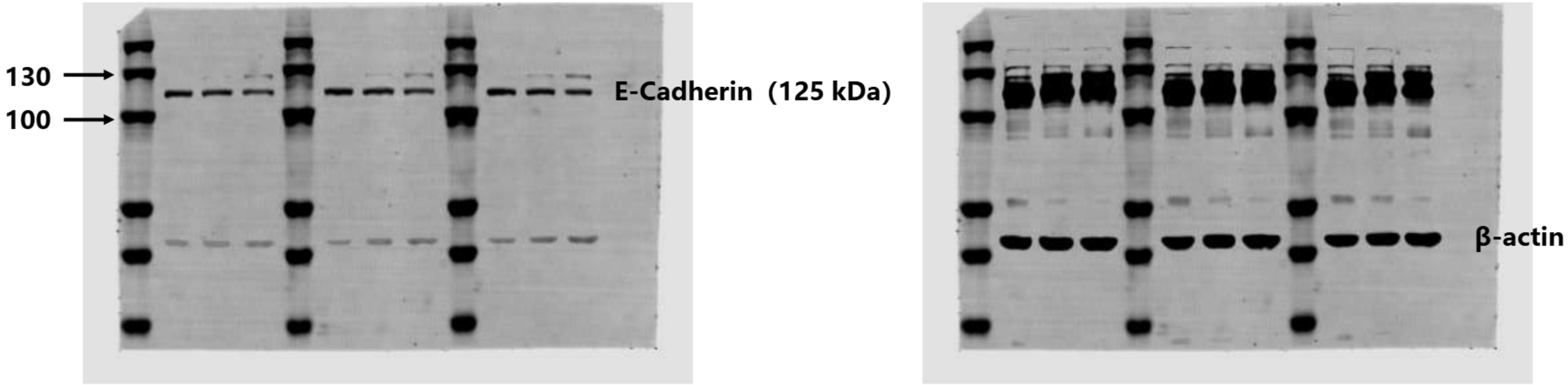

Lanes 10-12 of the unedited blot correspond to those shown in the cropped images within the manuscript.

Full unedited blot for Figure 1M

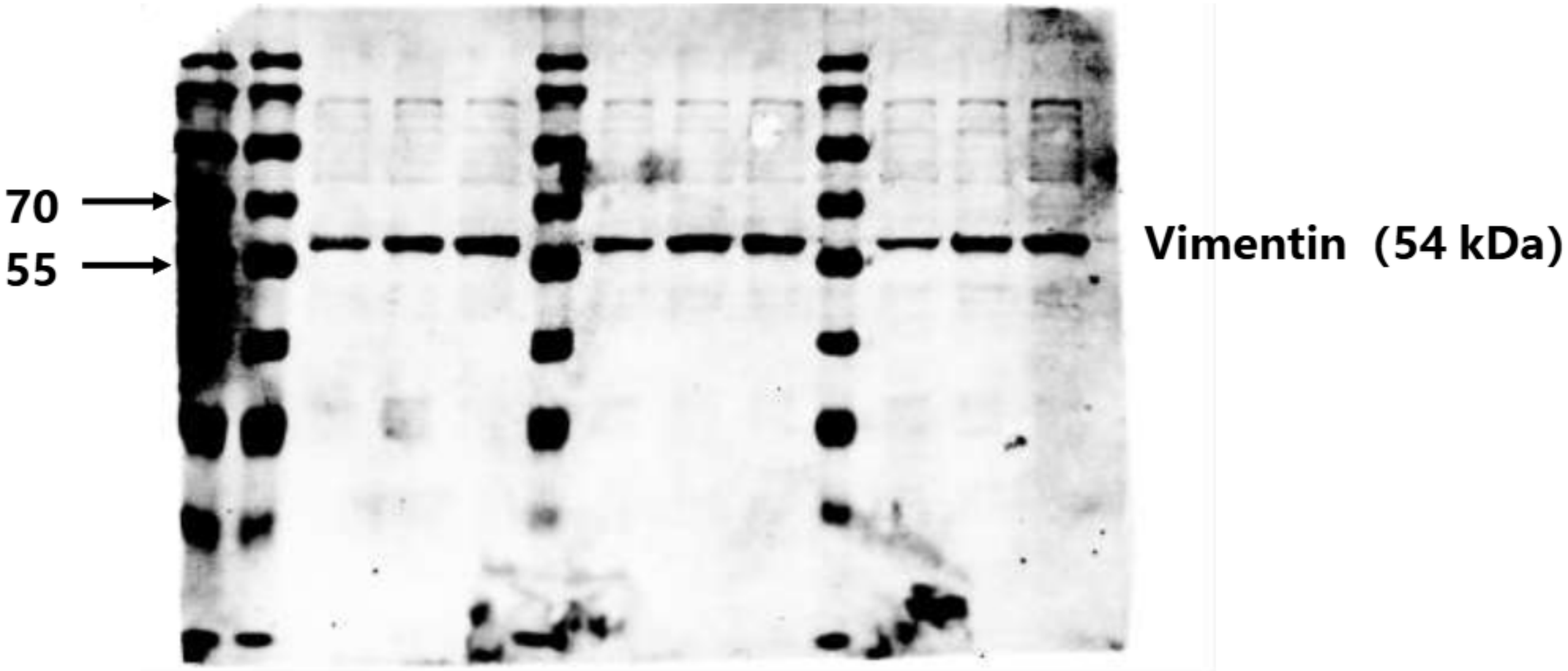

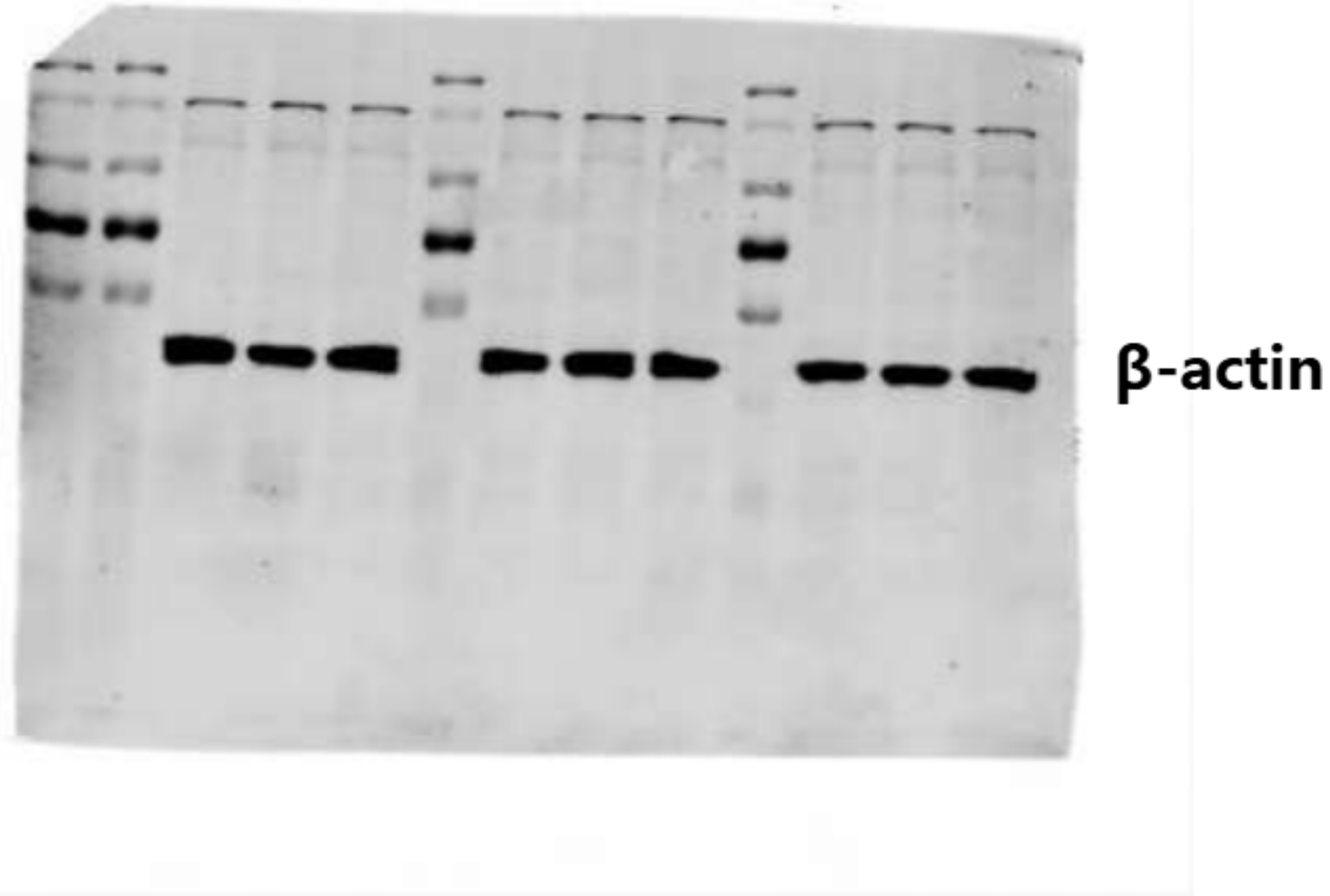

Lanes 11-13 of the unedited blot correspond to those shown in the cropped images within the manuscript.

Full unedited blot for Figure 1M

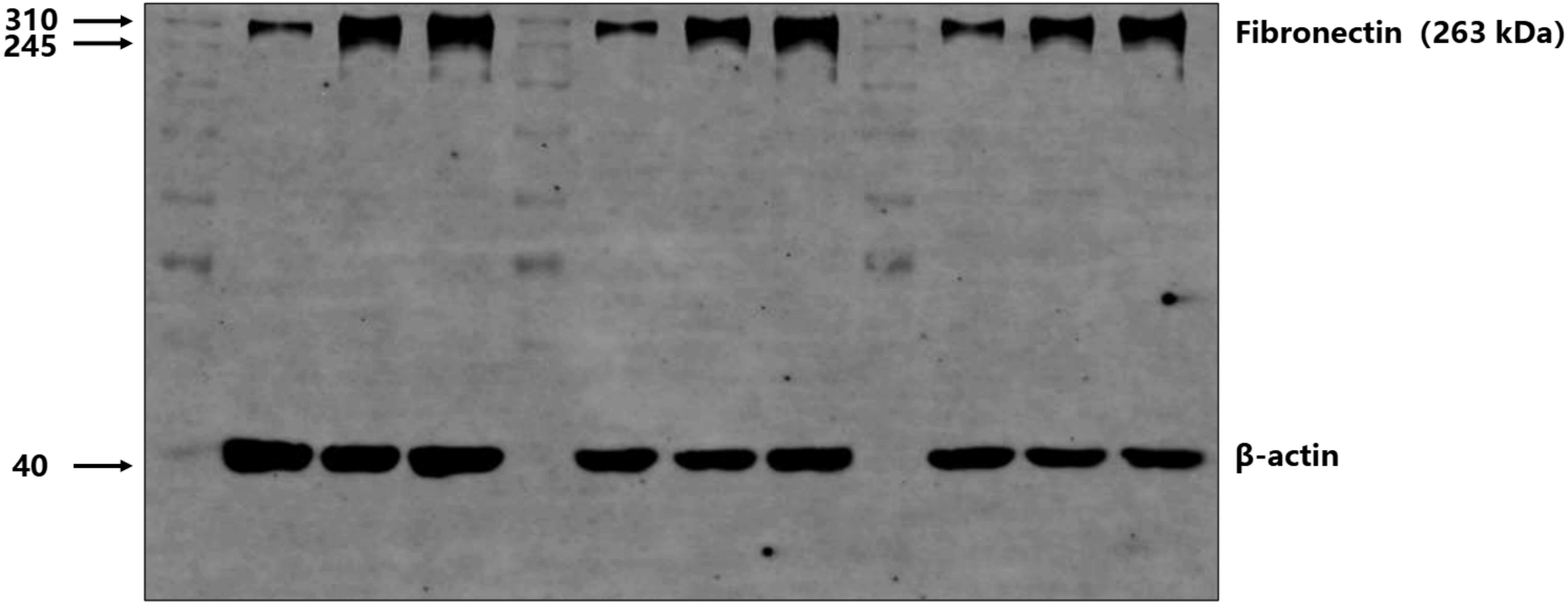

Lanes 10-12 of the unedited blot correspond to those shown in the cropped images within the manuscript.

Full unedited blot for Figure 1N

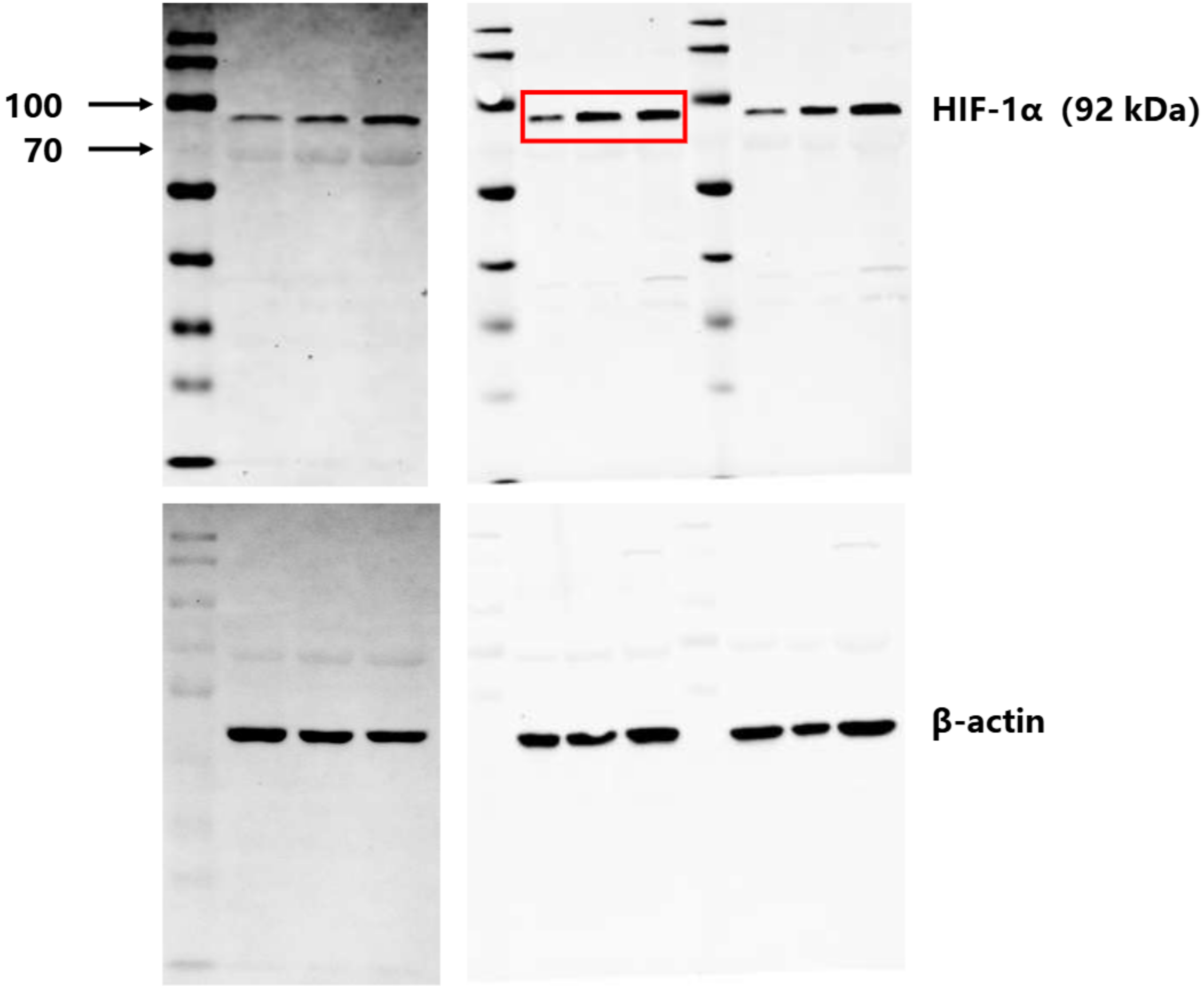

Lanes of the unedited blot correspond to those shown in the cropped images within the manuscript.

Full unedited blot for Figure 1N

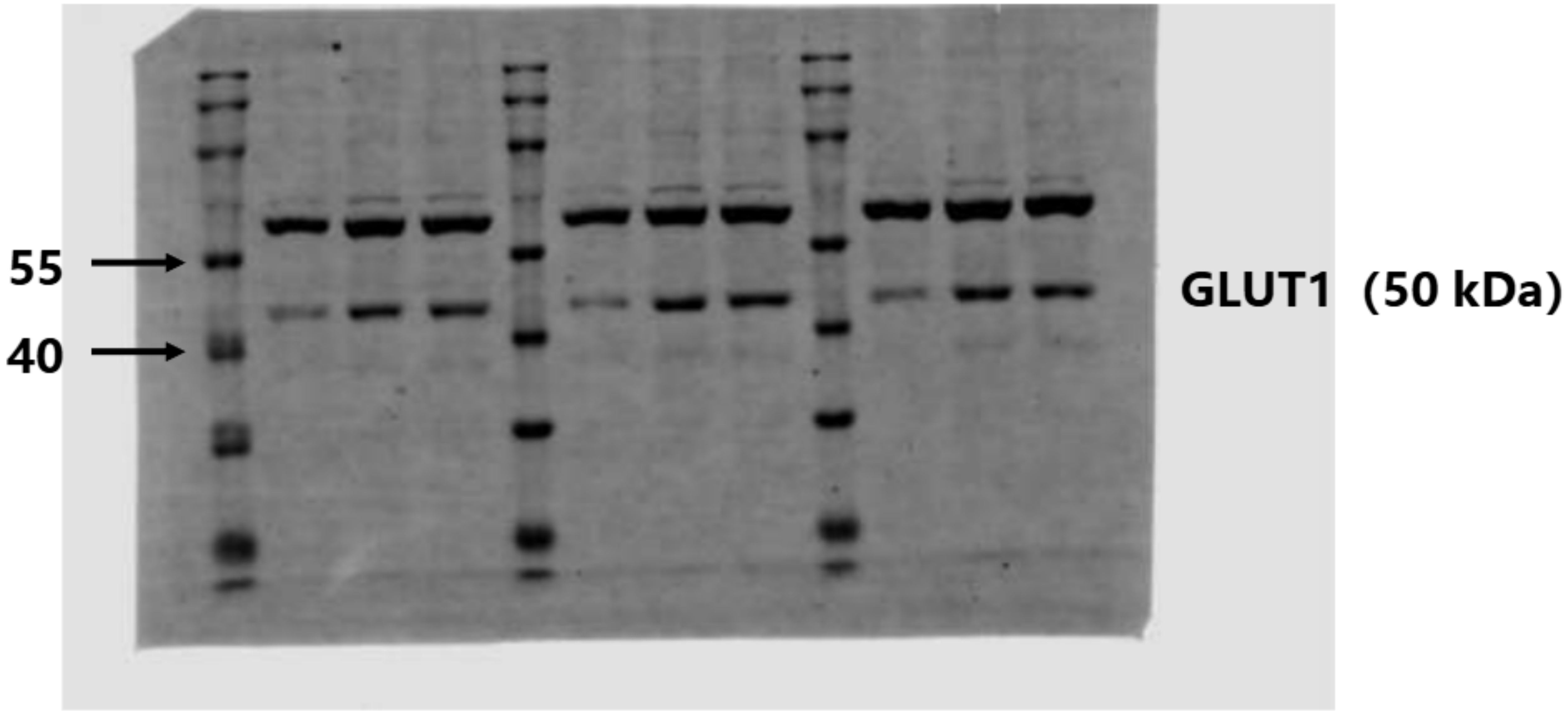

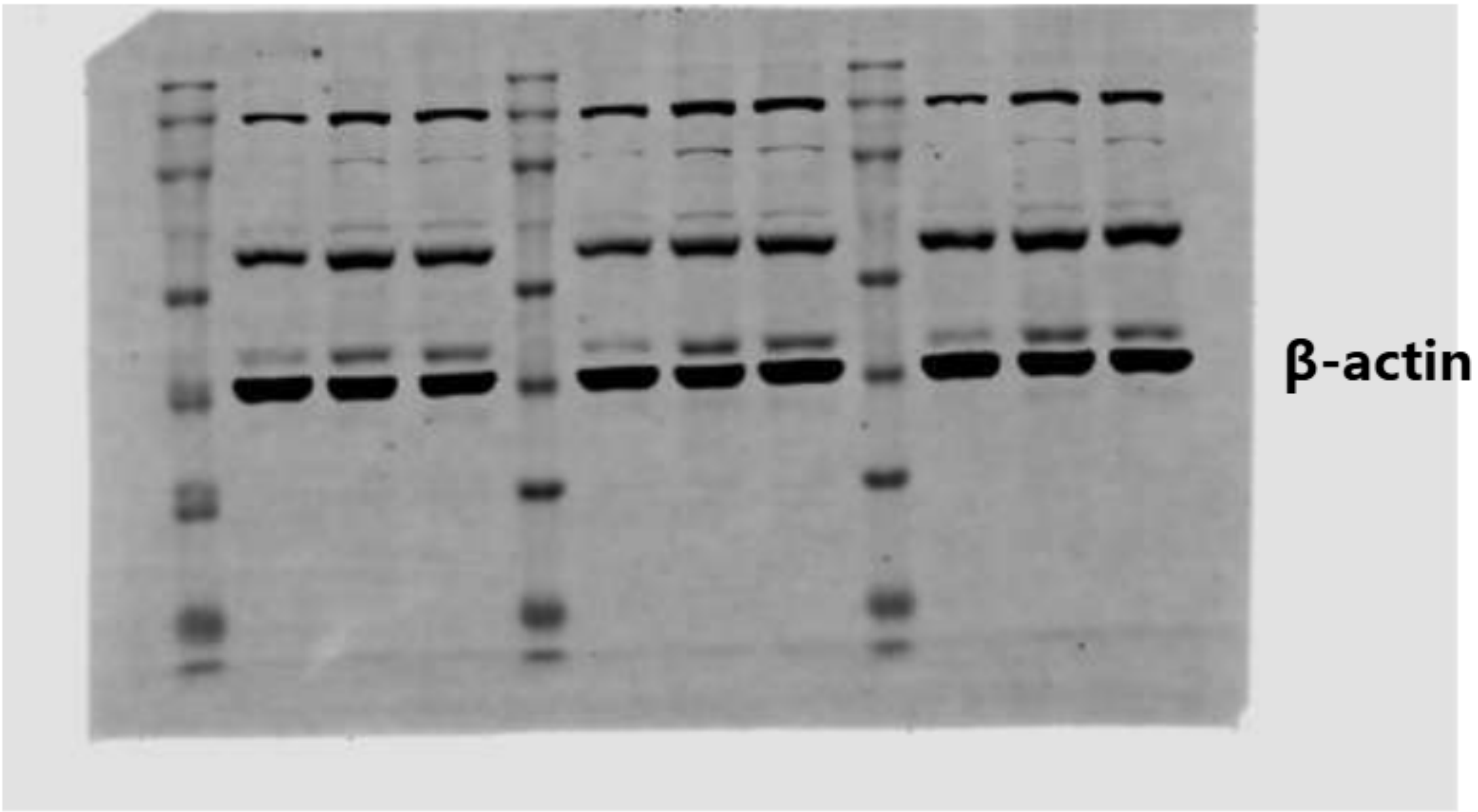

Lanes 6-8 of the unedited blot correspond to those shown in the cropped images within the manuscript.

Full unedited blot for Figure 1N

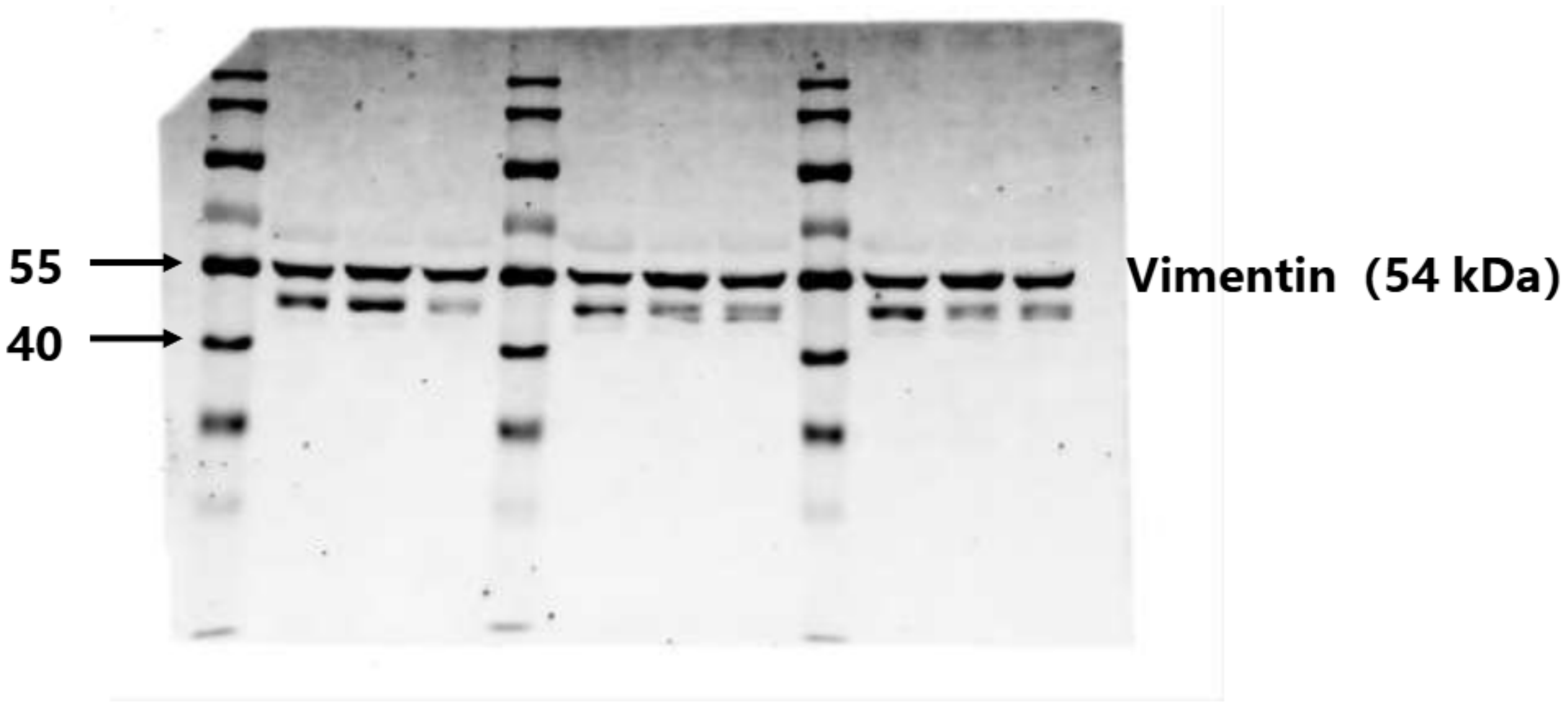

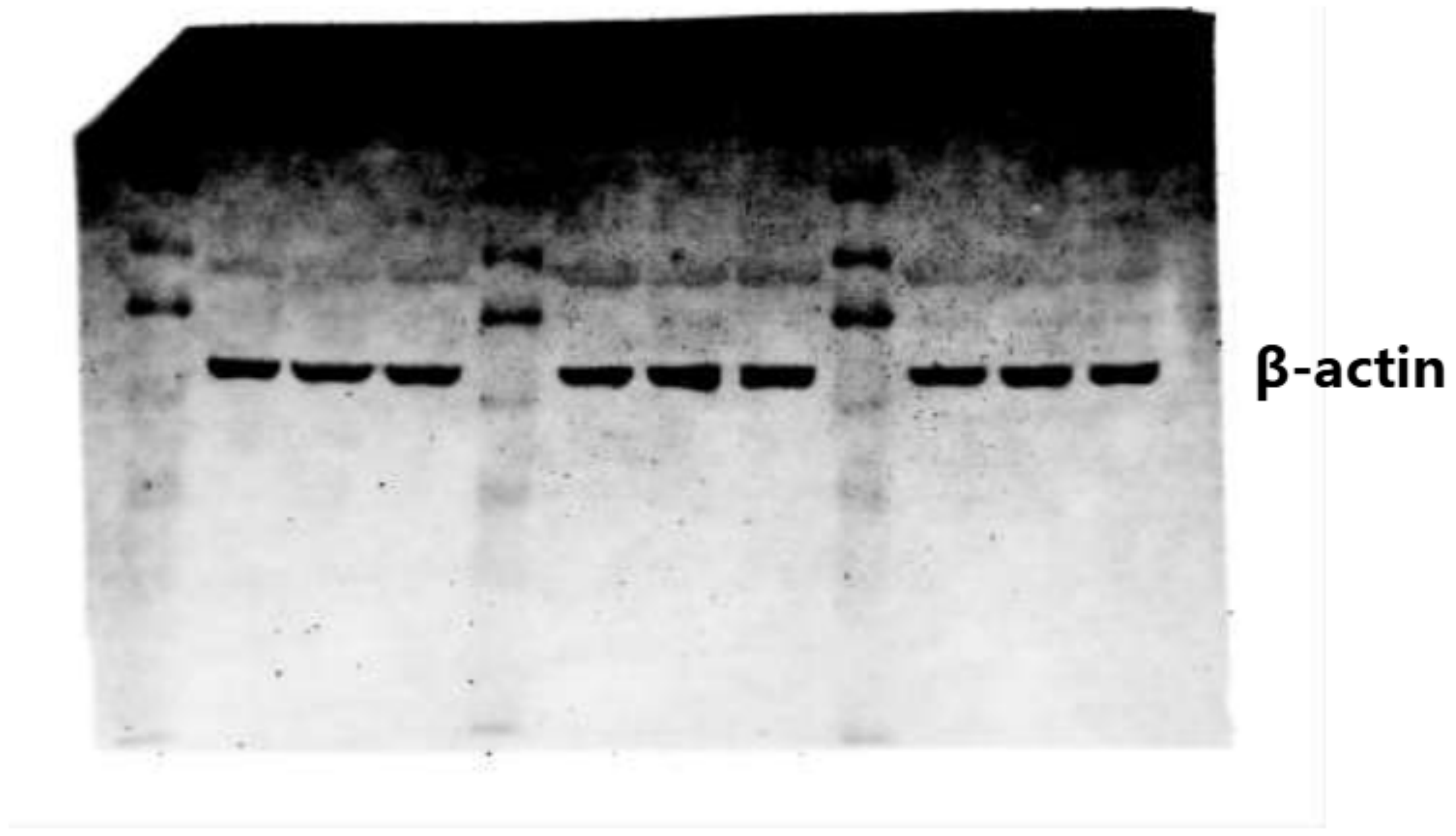

Lanes 2-4 of the unedited blot correspond to those shown in the cropped images within the manuscript.

Full unedited blot for Figure 1N

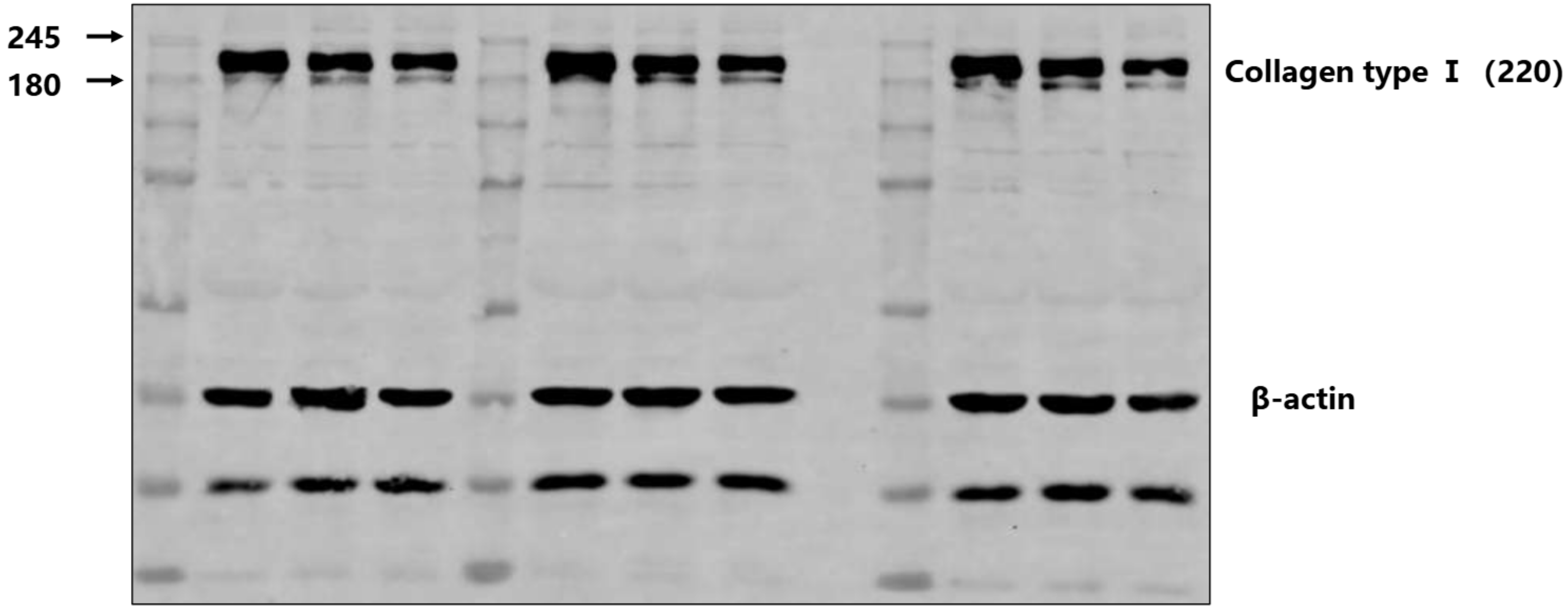

Lanes 6-8 of the unedited blot correspond to those shown in the cropped images within the manuscript.

Full unedited blot for Figure 1N

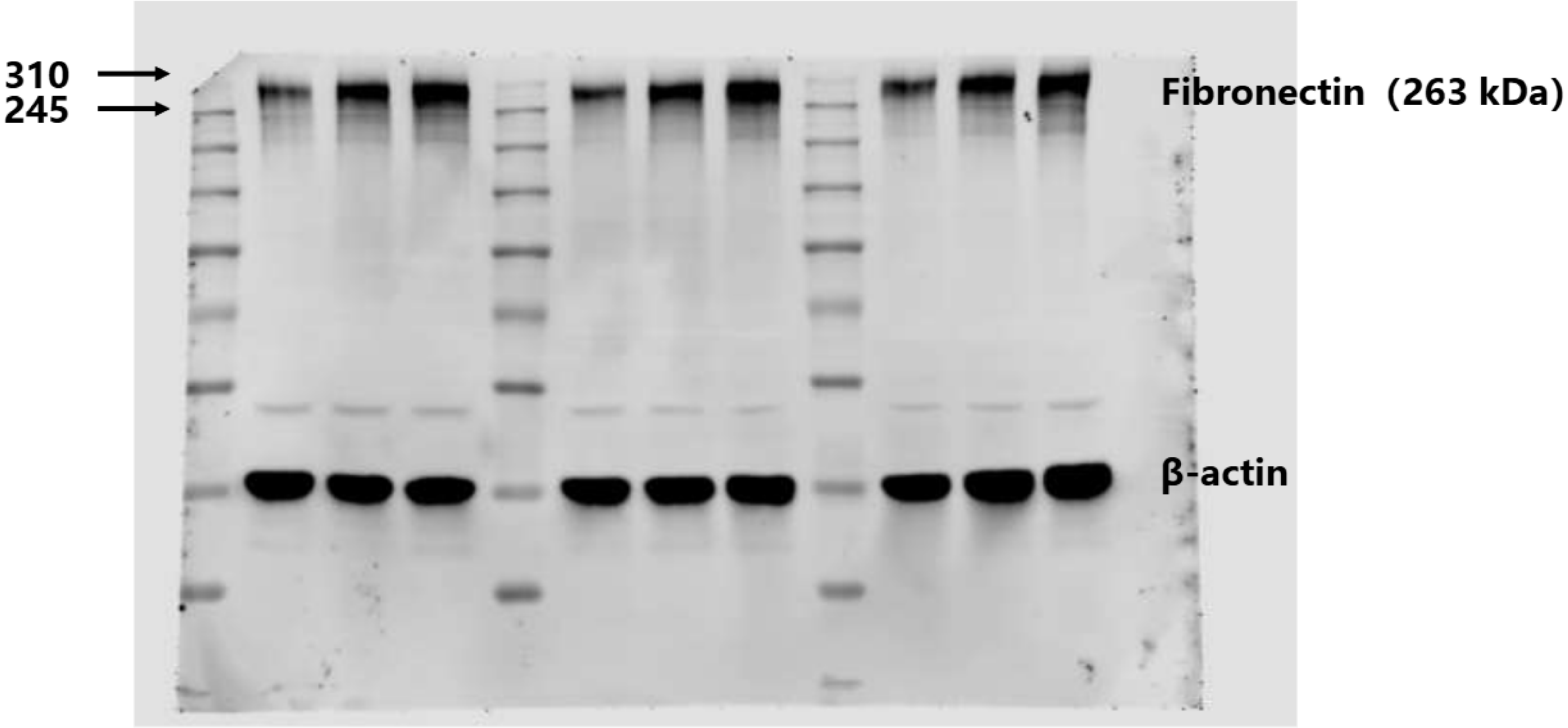

Lanes 6-8 of the unedited blot correspond to those shown in the cropped images within the manuscript.

Full unedited blot for Figure 1O

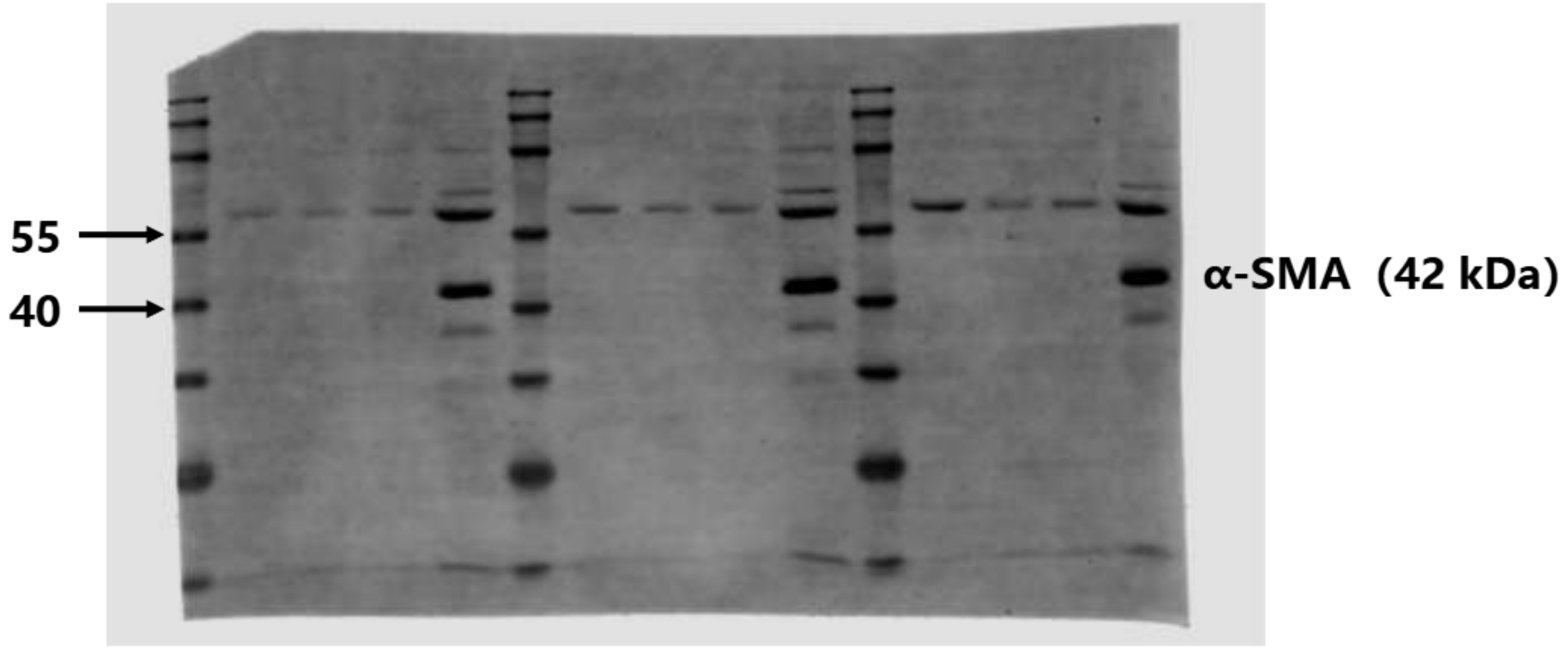

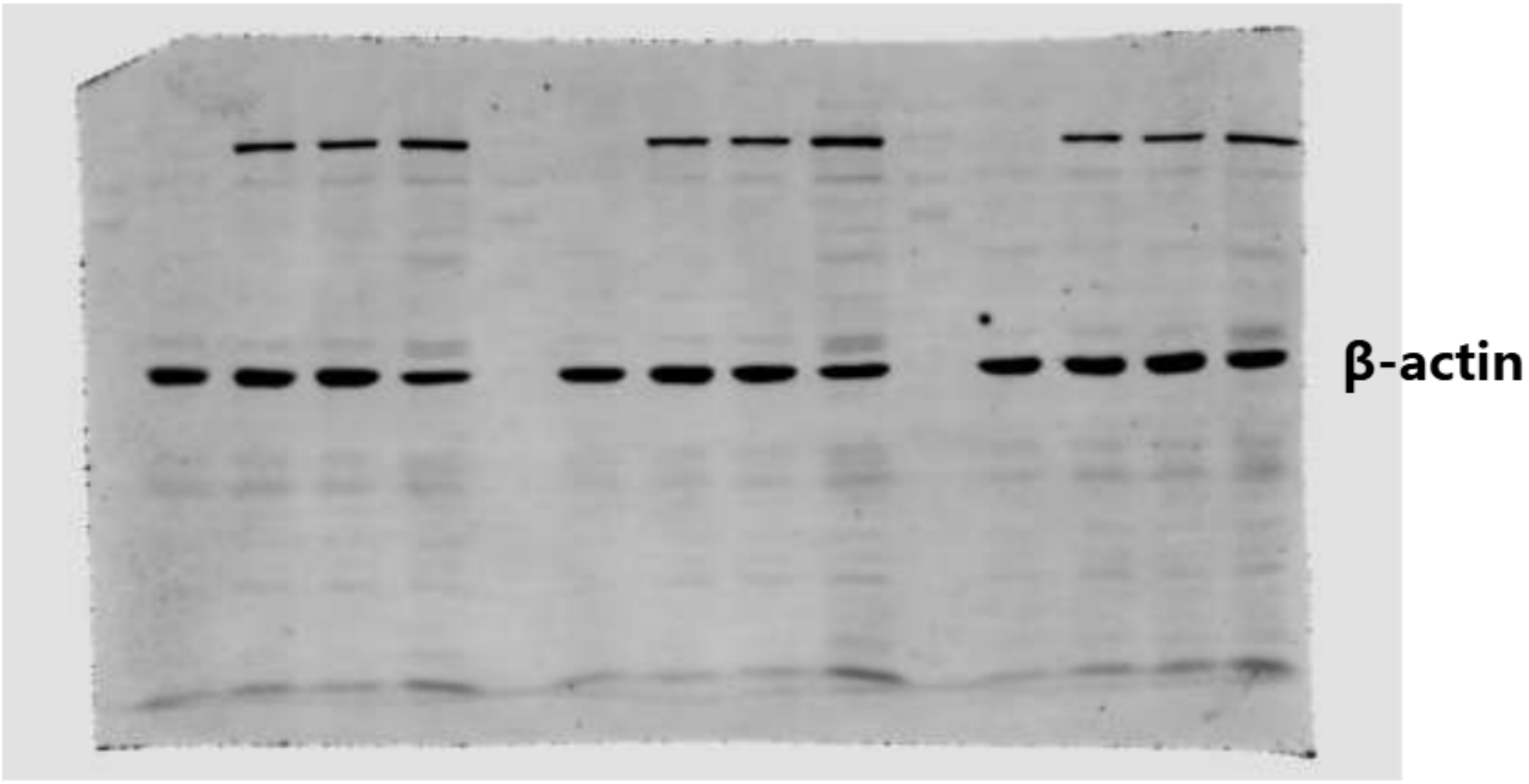

Lanes 12-15 of the unedited blot correspond to those shown in the cropped images within the manuscript.

Full unedited blot for Figure 1O

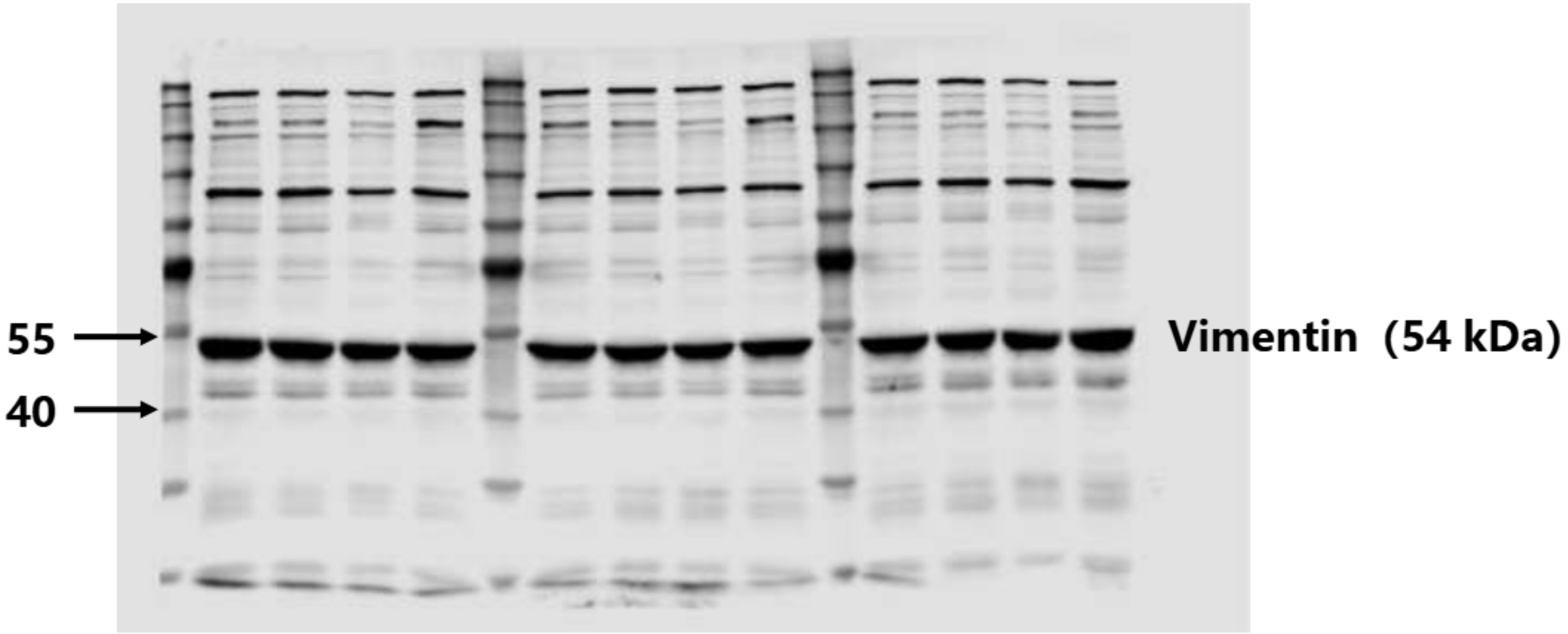

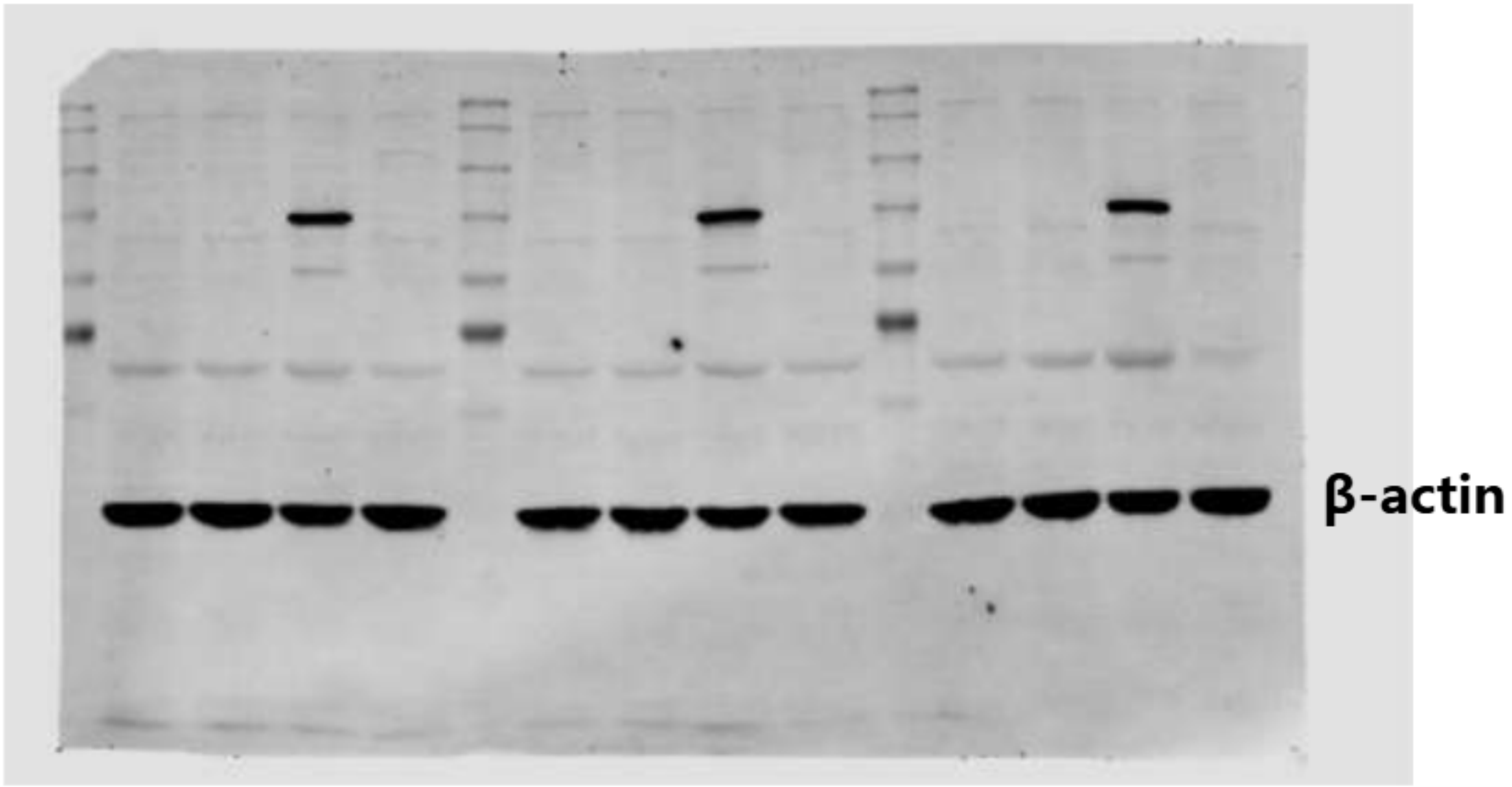

Lanes 12-15 of the unedited blot correspond to those shown in the cropped images within the manuscript.

Full unedited blot for Figure 1O

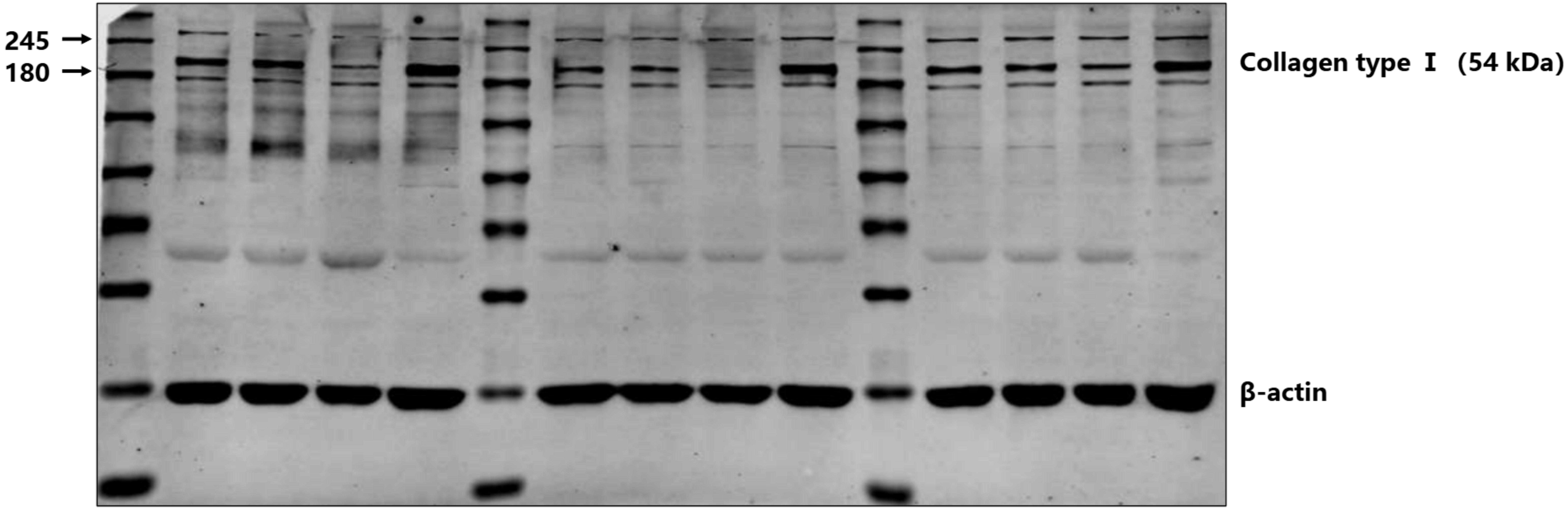

Lanes 12-15 of the unedited blot correspond to those shown in the cropped images within the manuscript.

Full unedited blot for Figure 1O

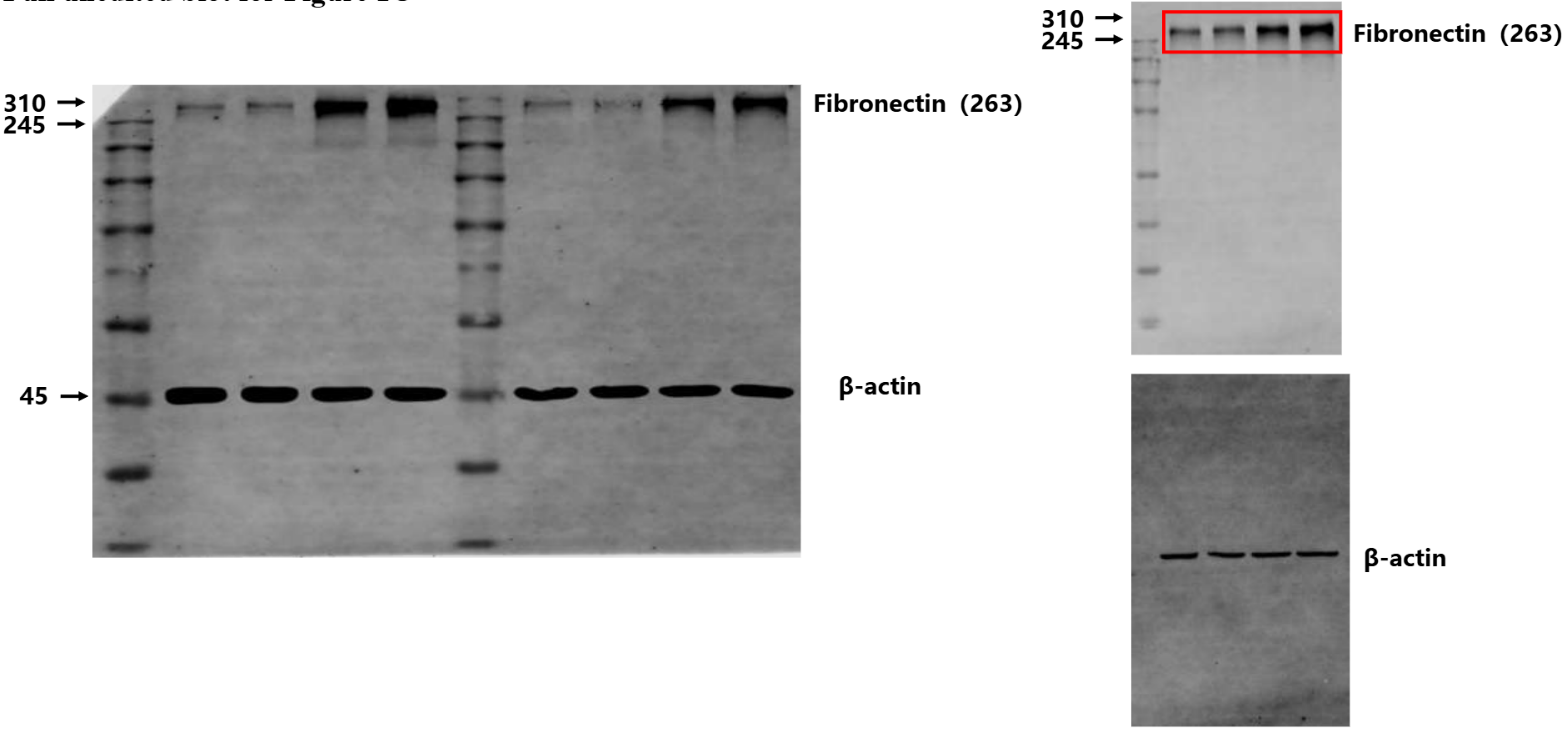

Lanes of the unedited blot correspond to those shown in the cropped images within the manuscript.

Full unedited blot for Figure 2C

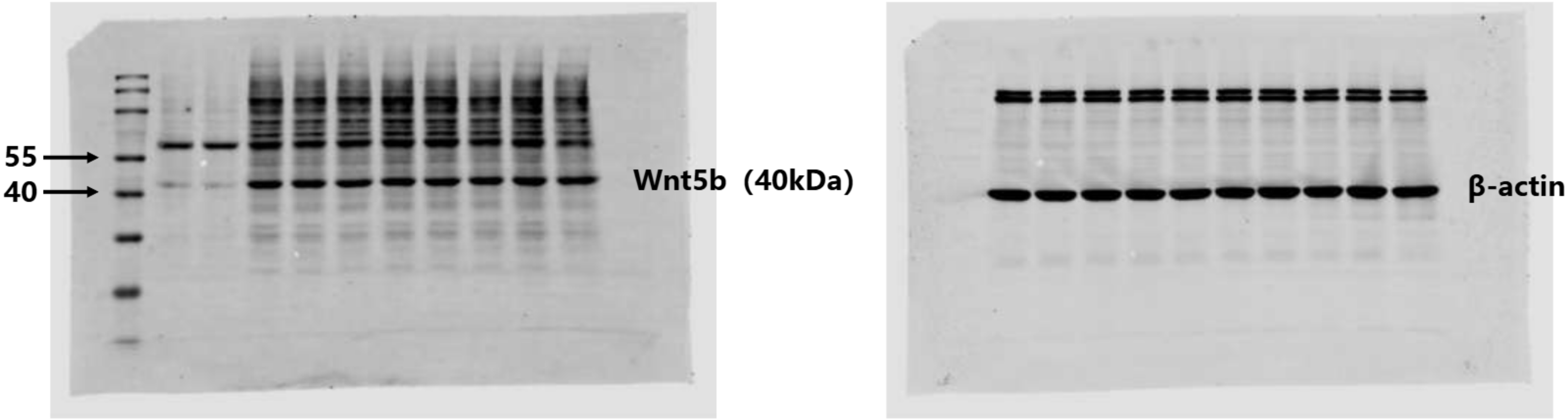

Lanes 2-11 of the unedited blot correspond to those shown in the cropped images within the manuscript.

Full unedited blot for Figure 2C

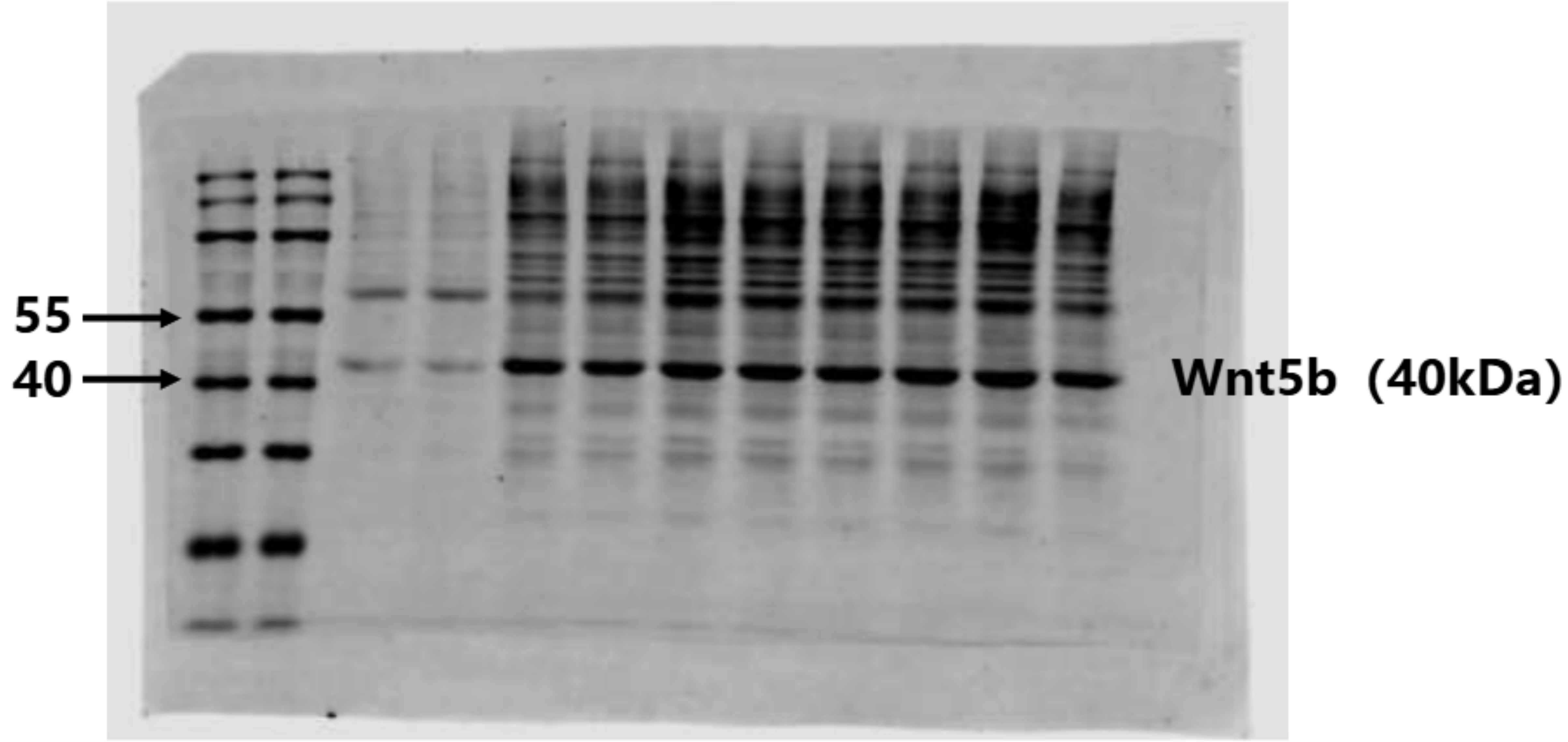

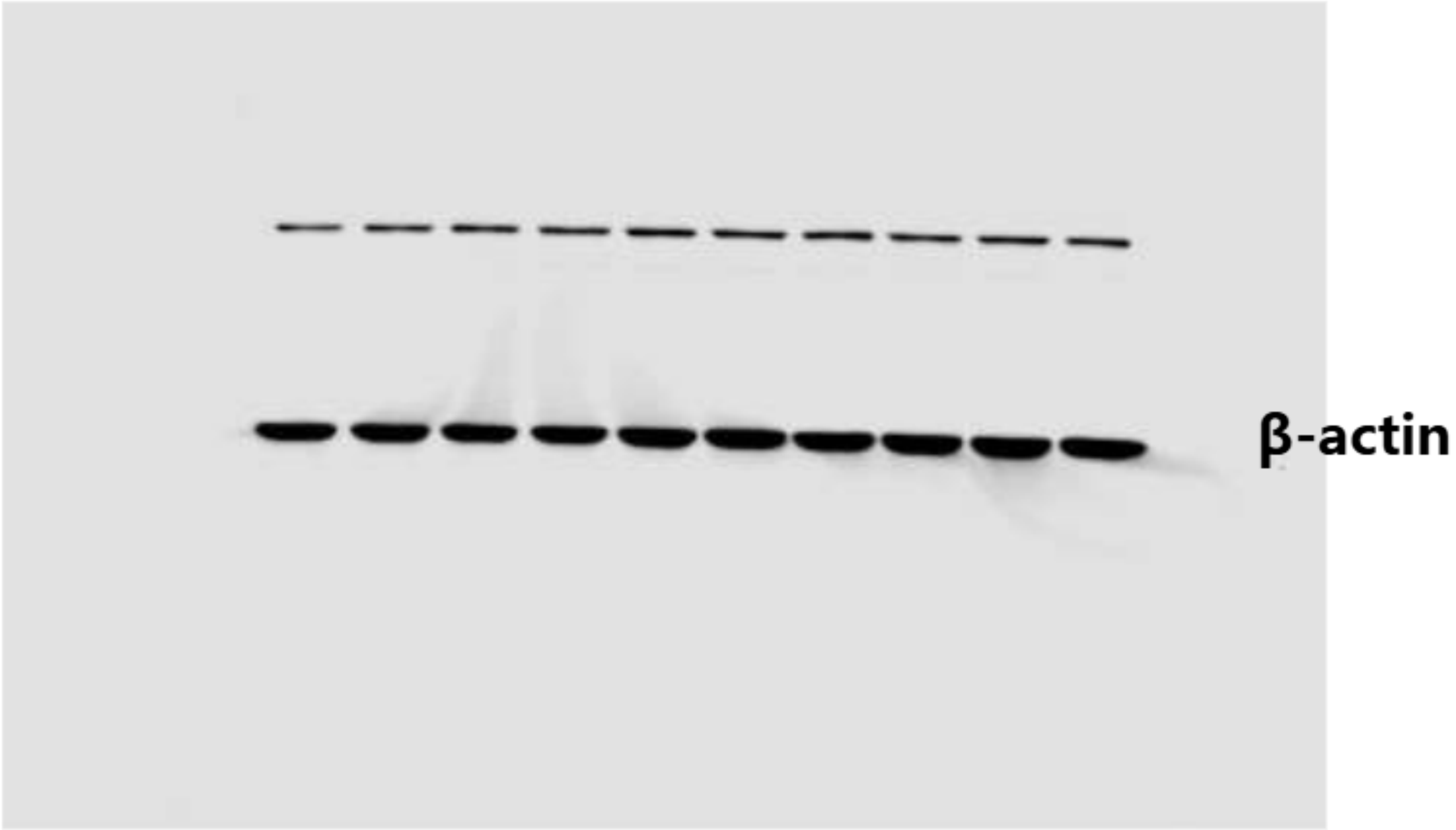

Full unedited blot for Figure 2C

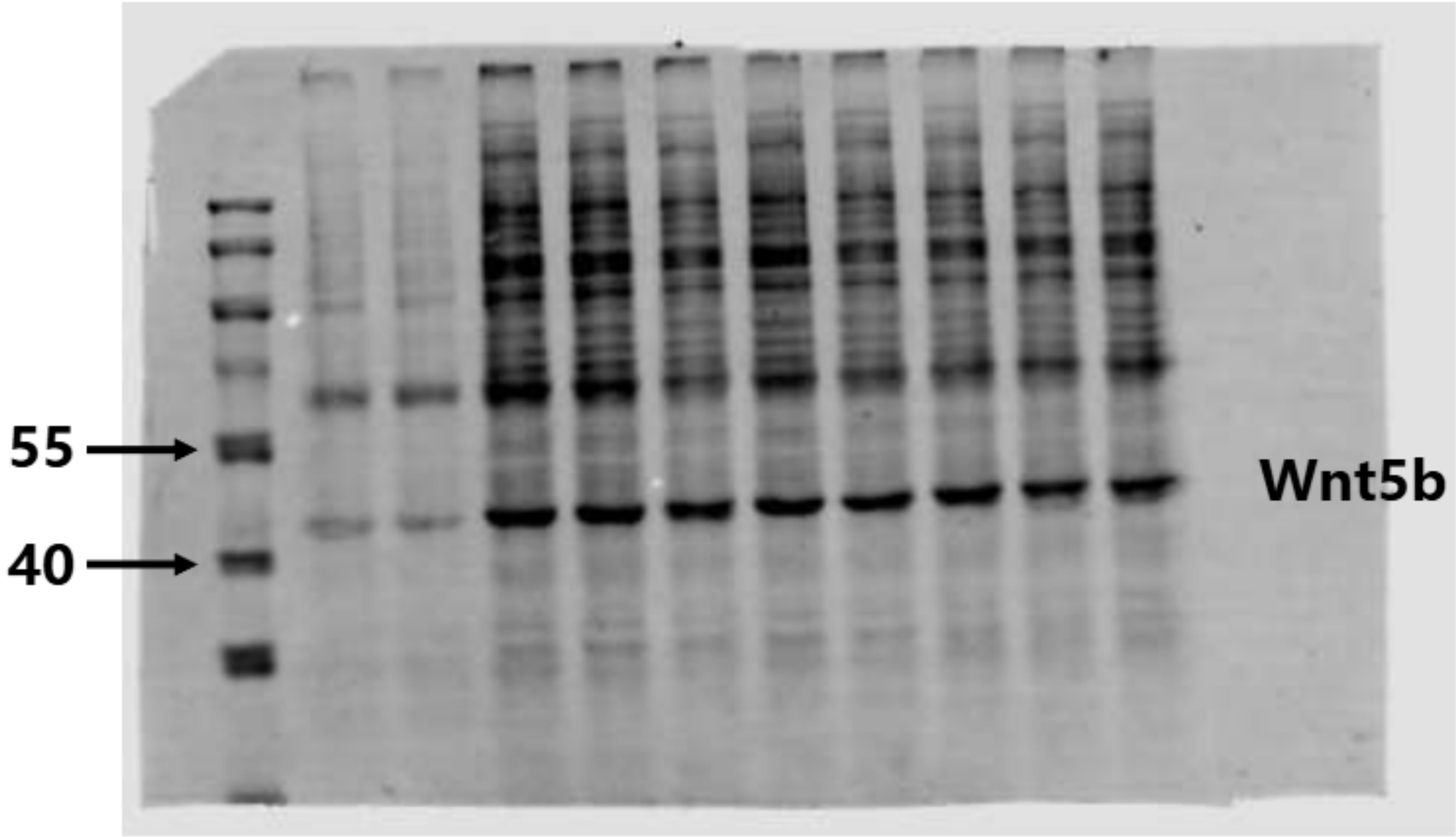

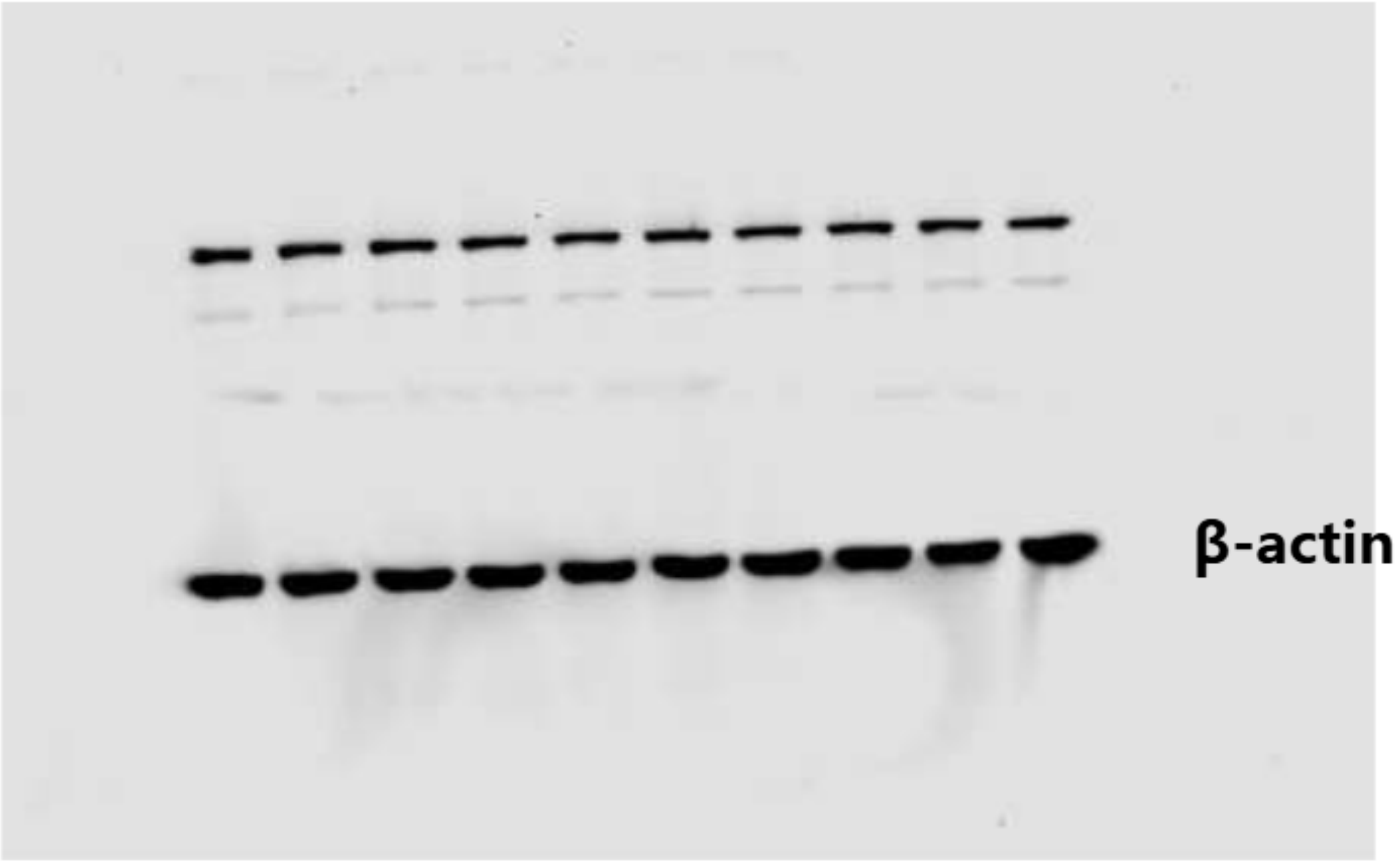

Full unedited blot for Figure 2C

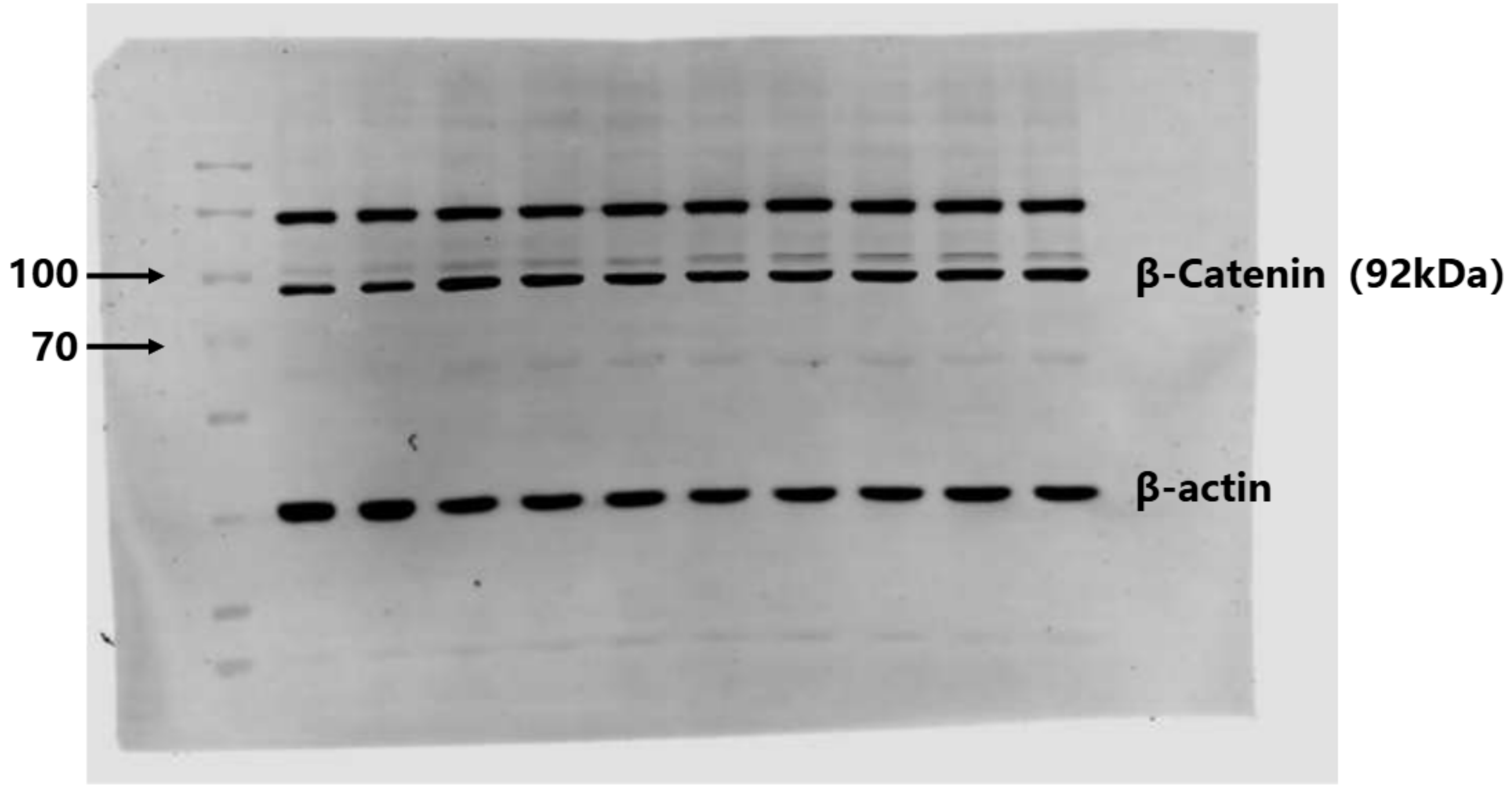

Lanes 2-11 of the unedited blot correspond to those shown in the cropped images within the manuscript.

Full unedited blot for Figure 2C

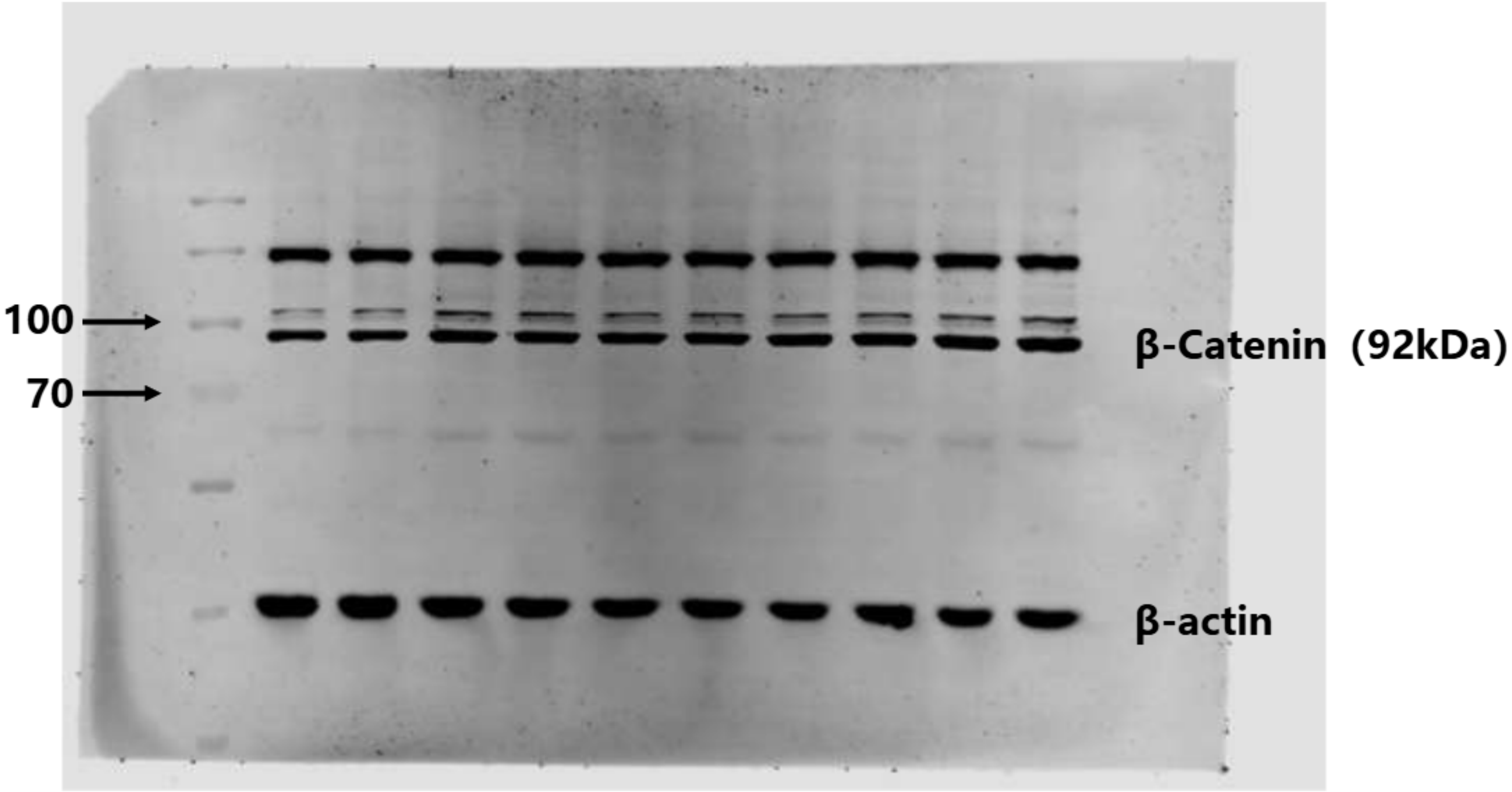

Full unedited blot for Figure 2C

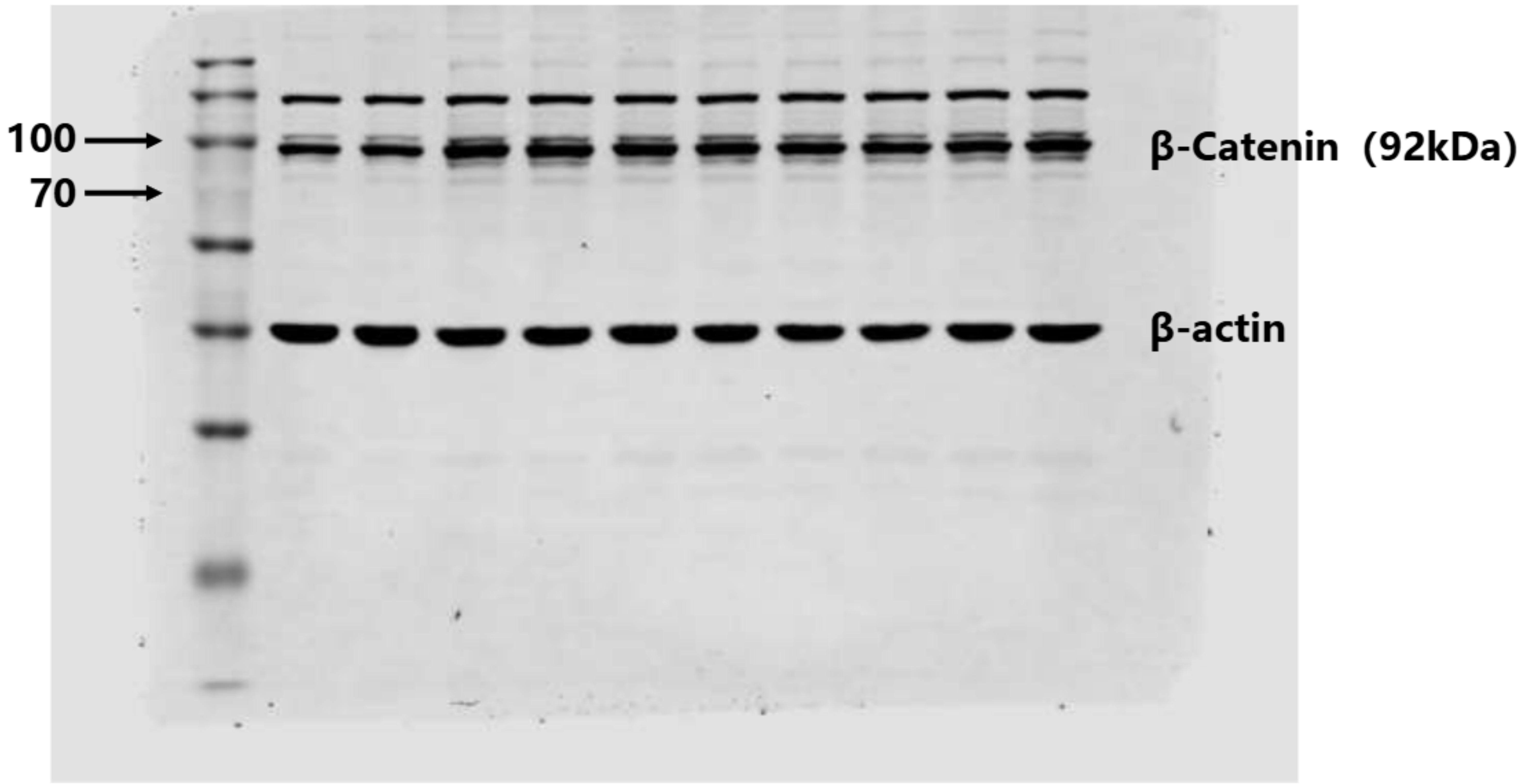

Full unedited blot for Figure 2F

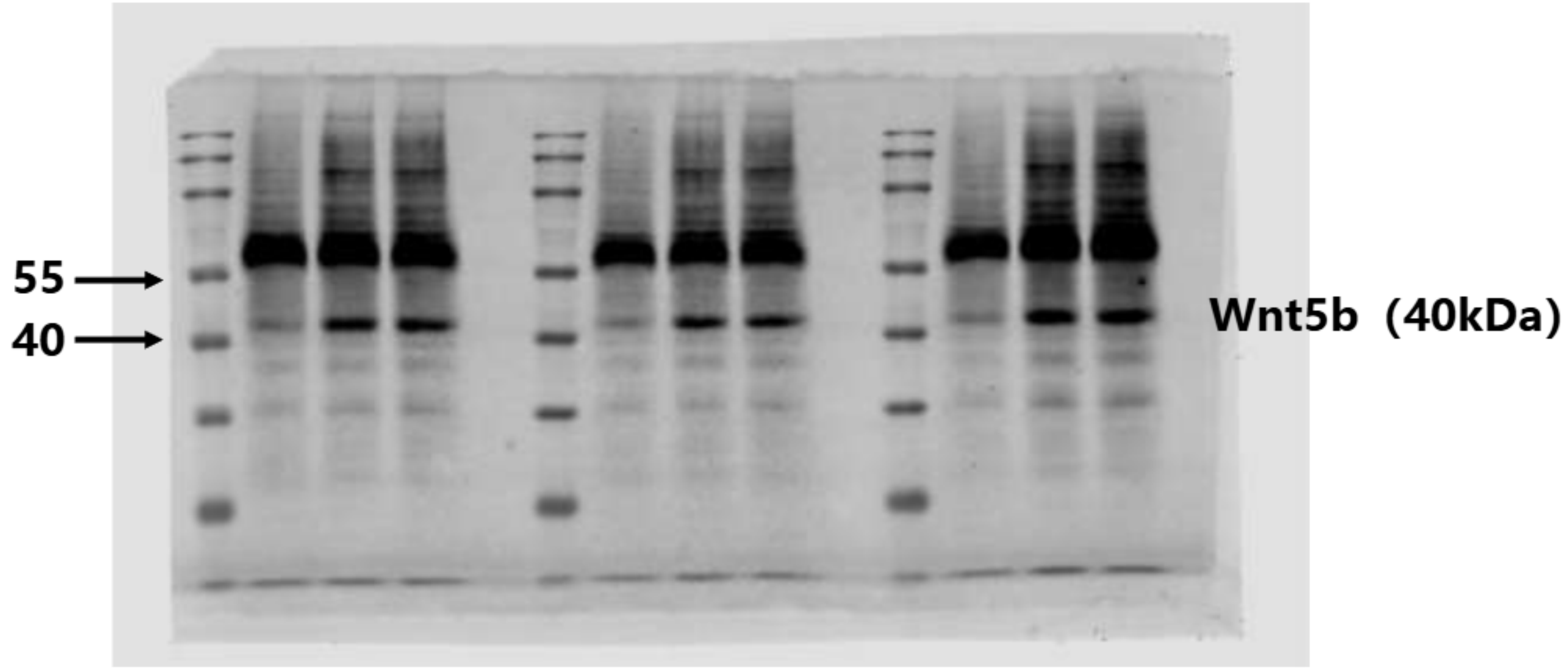

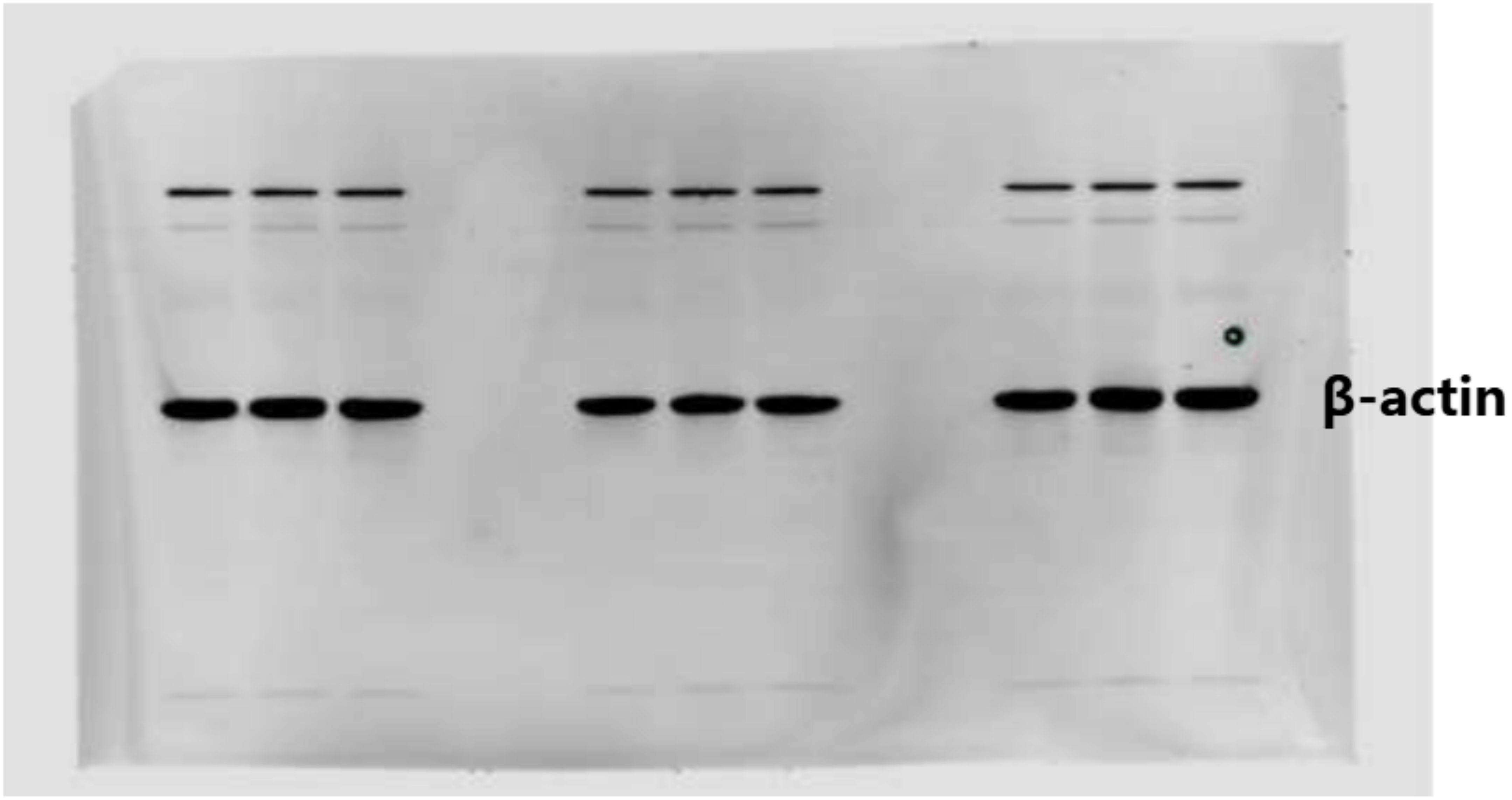

Lanes 2-4 of the unedited blot correspond to those shown in the cropped images within the manuscript.

Full unedited blot for Figure 2G

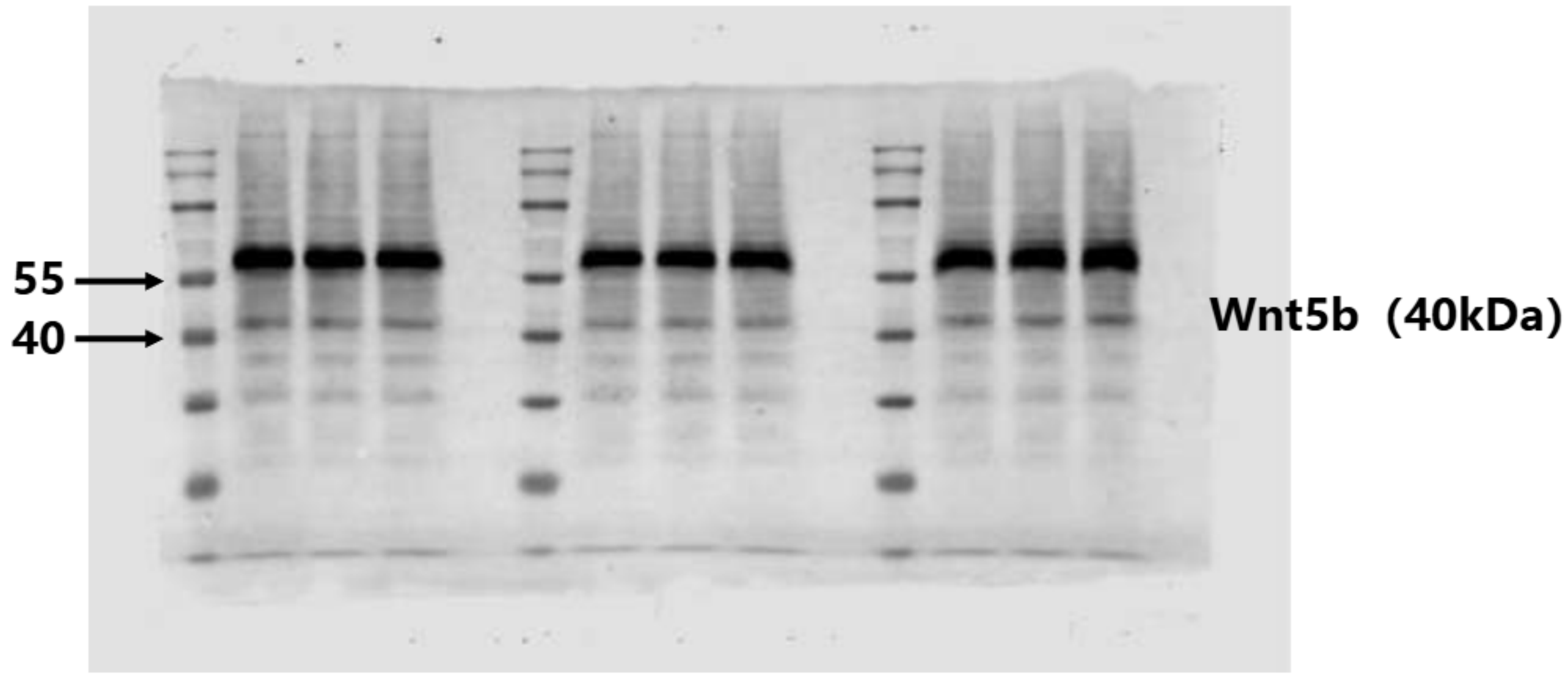

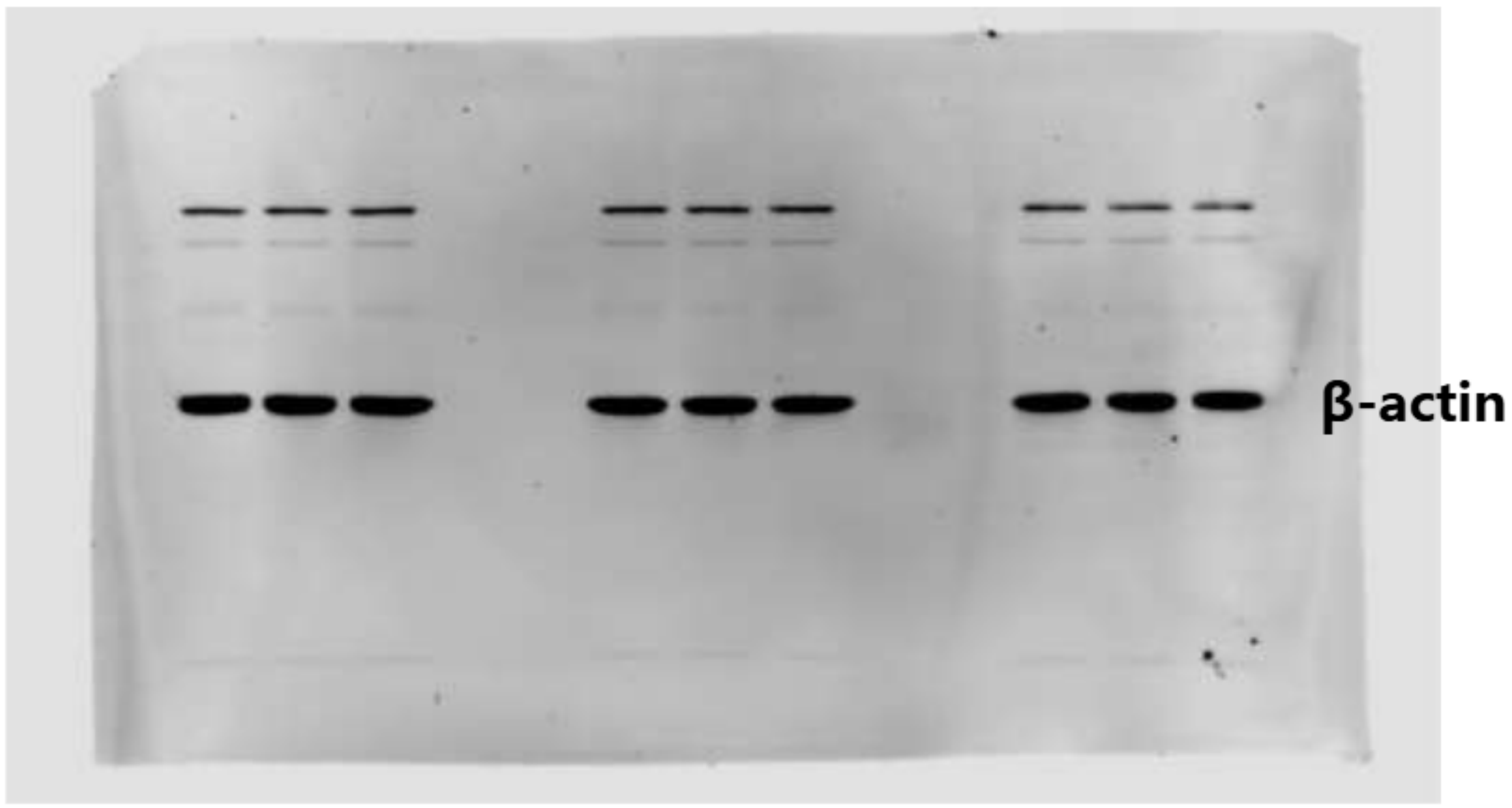

Lanes 2-4 of the unedited blot correspond to those shown in the cropped images within the manuscript.

Full unedited blot for Figure 2H

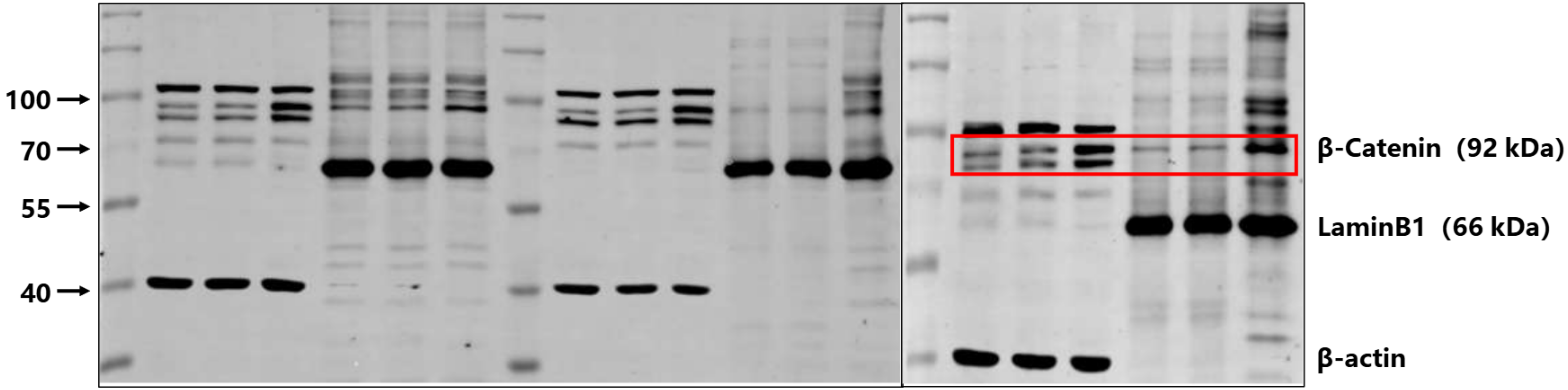

Lanes of the unedited blot correspond to those shown in the cropped images within the manuscript.

Full unedited blot for Figure 3A

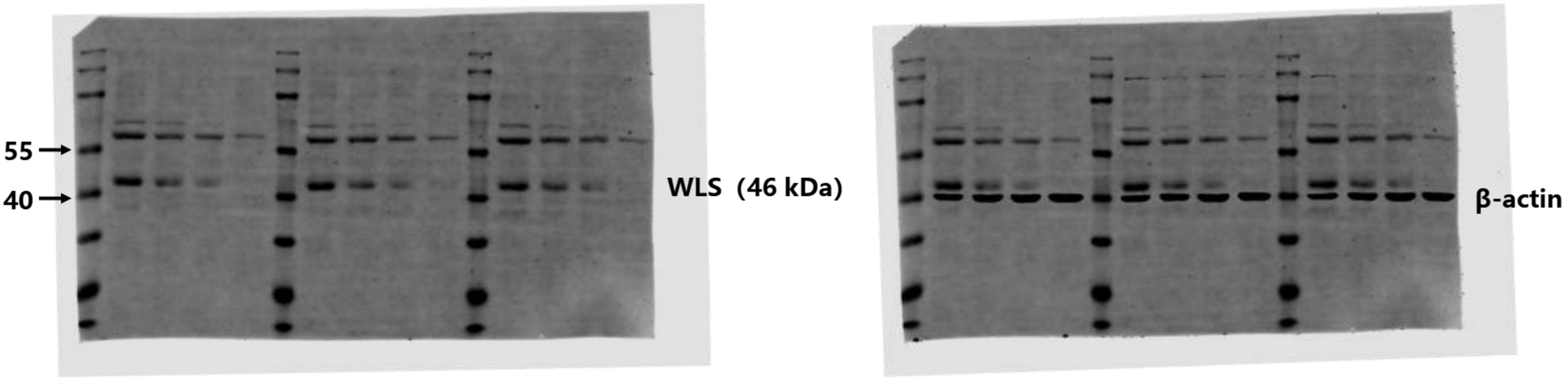

Lanes 7-10 of the unedited blot correspond to those shown in the cropped images within the manuscript.

Full unedited blot for Figure 3B

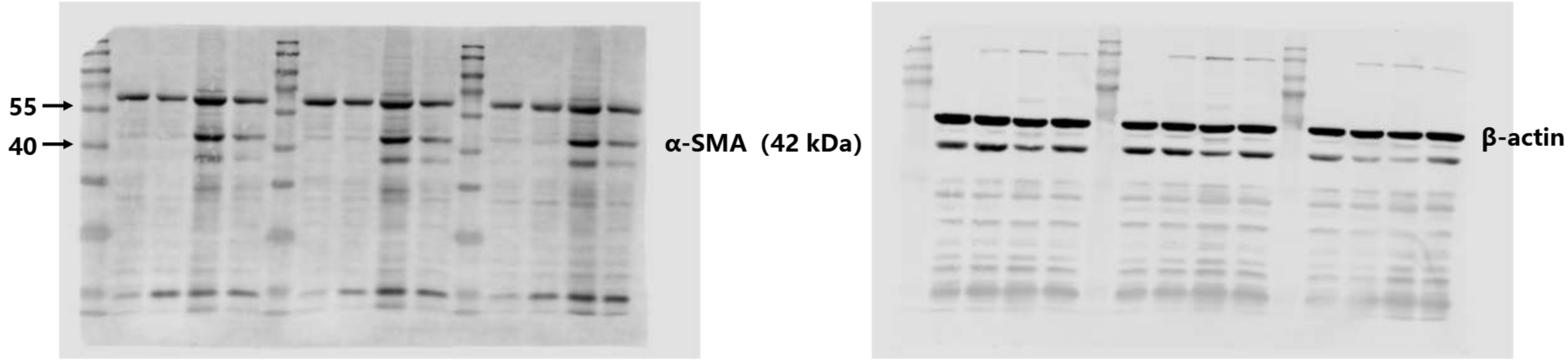

Lanes 12-15 of the unedited blot correspond to those shown in the cropped images within the manuscript.

Full unedited blot for Figure 3B

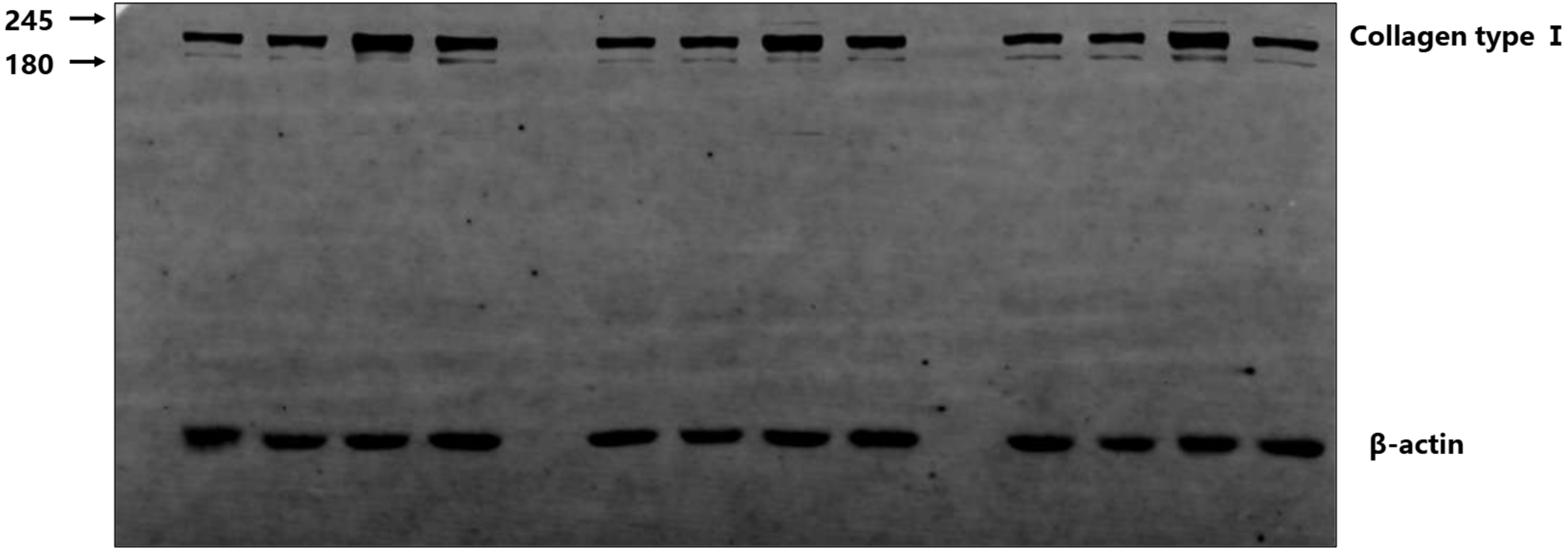

Lanes 2-5 of the unedited blot correspond to those shown in the cropped images within the manuscript.

Full unedited blot for Figure 3B

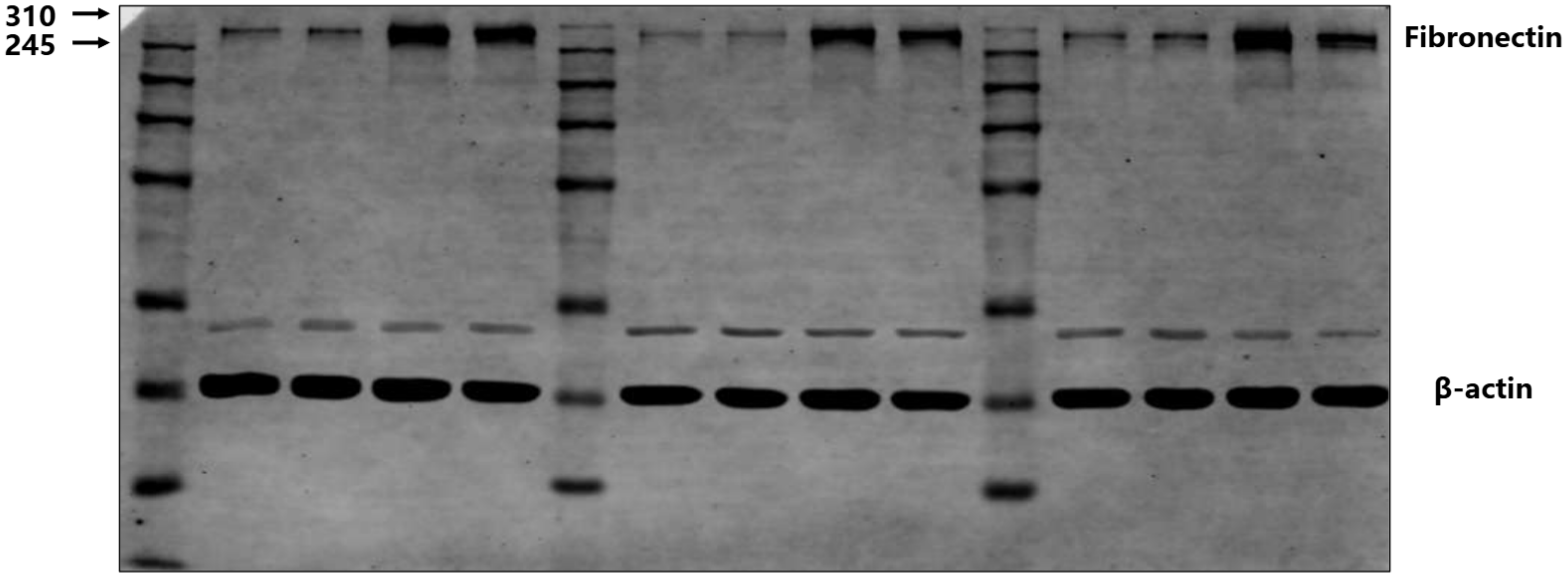

Lanes 12-15 of the unedited blot correspond to those shown in the cropped images within the manuscript.

Full unedited blot for Figure 3C

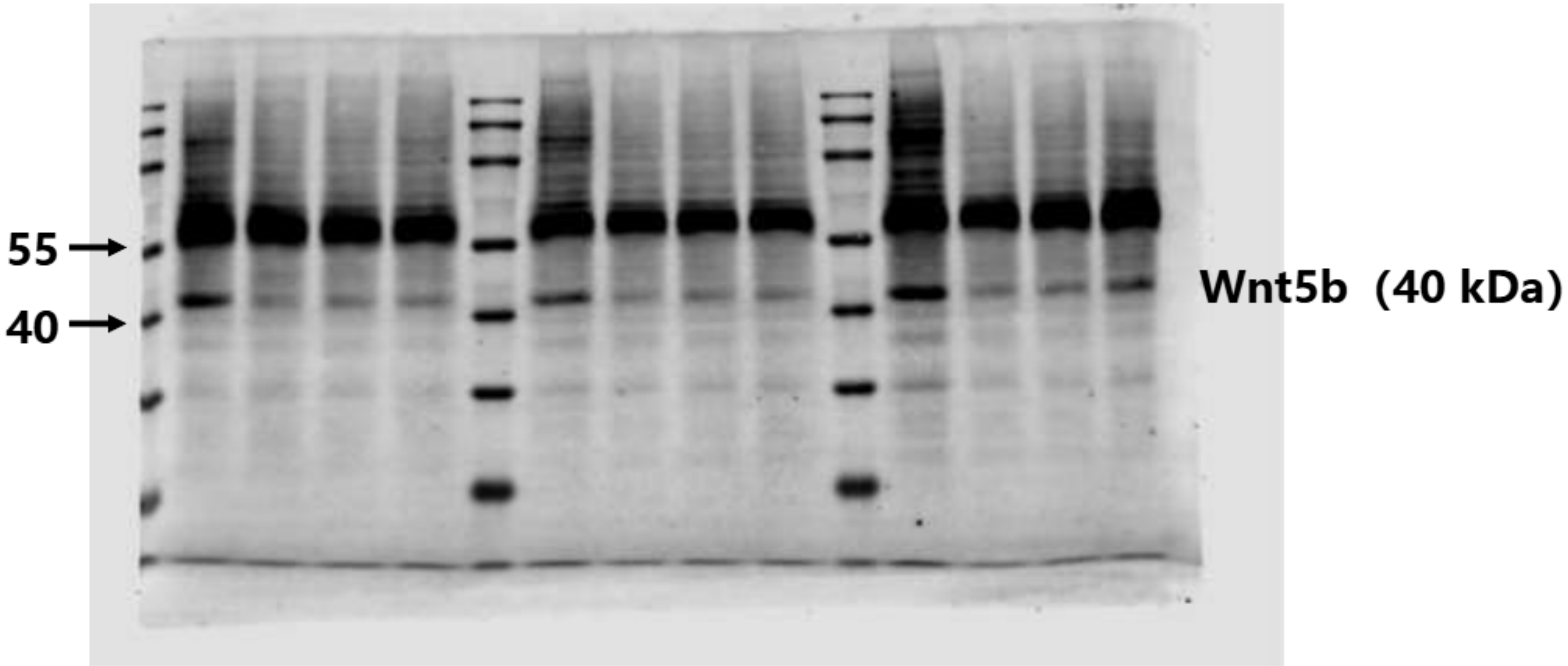

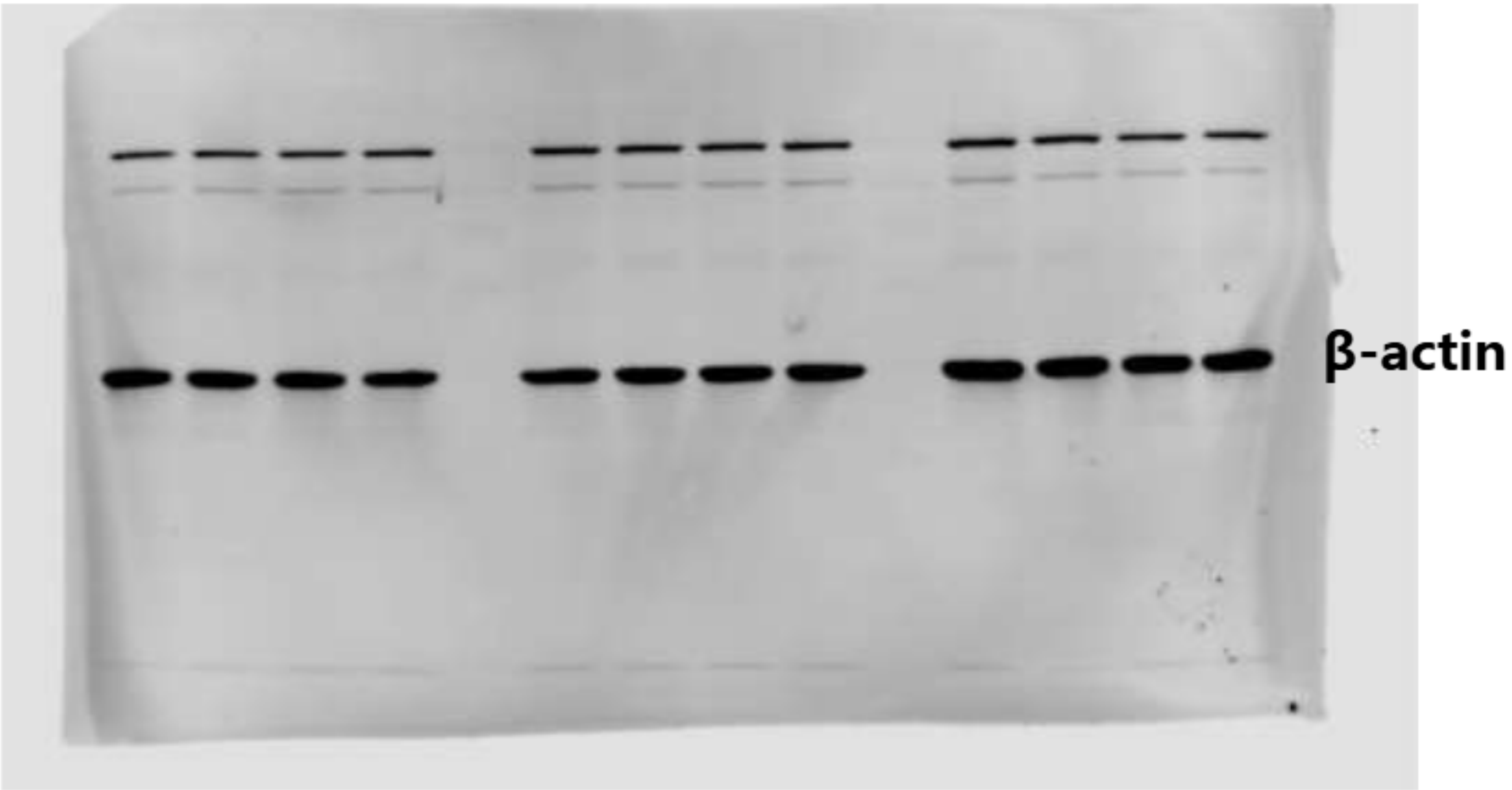

Lanes 12-15 of the unedited blot correspond to those shown in the cropped images within the manuscript.

Full unedited blot for Figure 3D

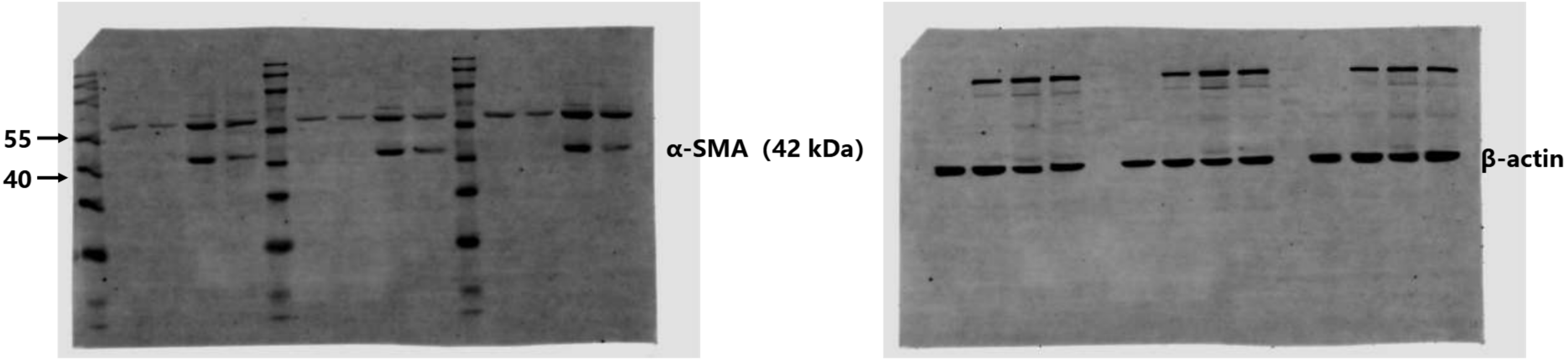

Lanes 2-5 of the unedited blot correspond to those shown in the cropped images within the manuscript.

Full unedited blot for Figure 3D

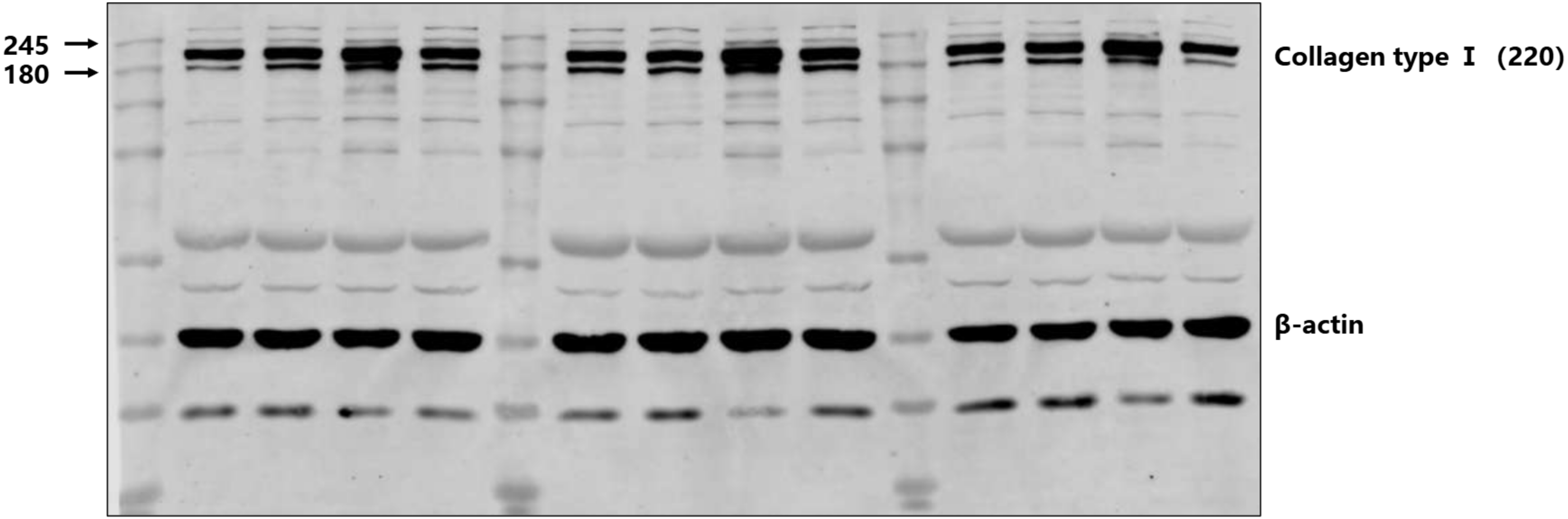

Lanes 7-10 of the unedited blot correspond to those shown in the cropped images within the manuscript.

Full unedited blot for Figure 3D

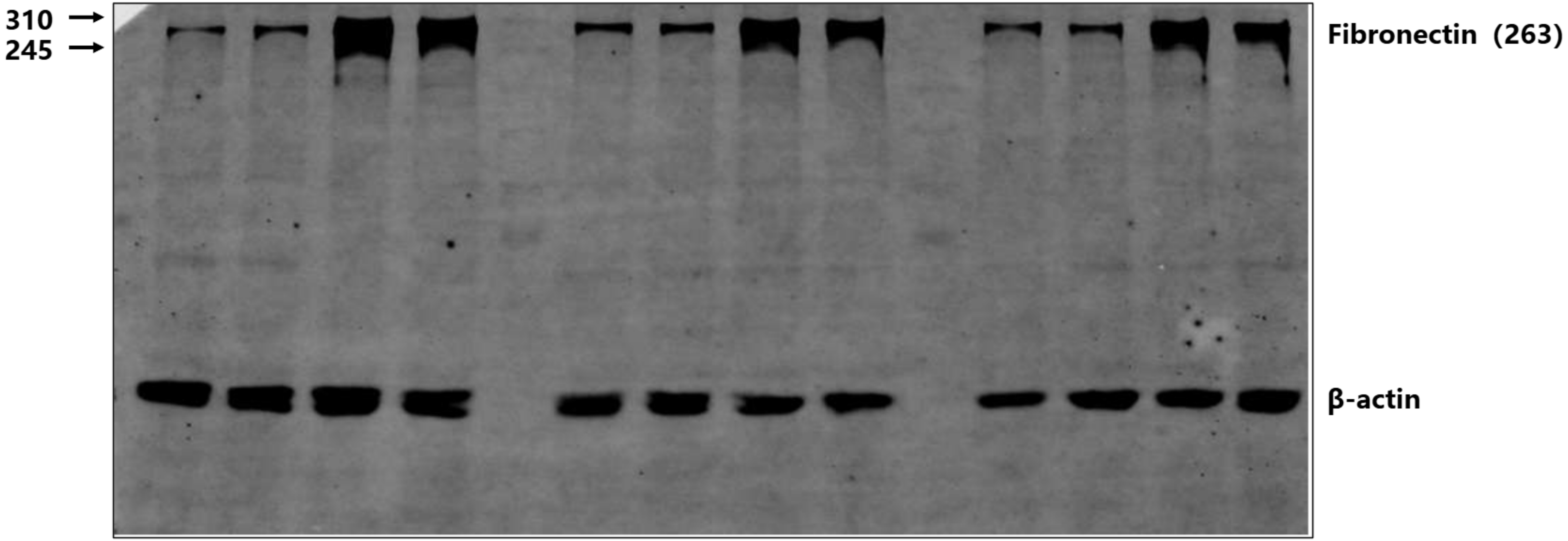

Lanes 7-10 of the unedited blot correspond to those shown in the cropped images within the manuscript.

Full unedited blot for Figure 3E

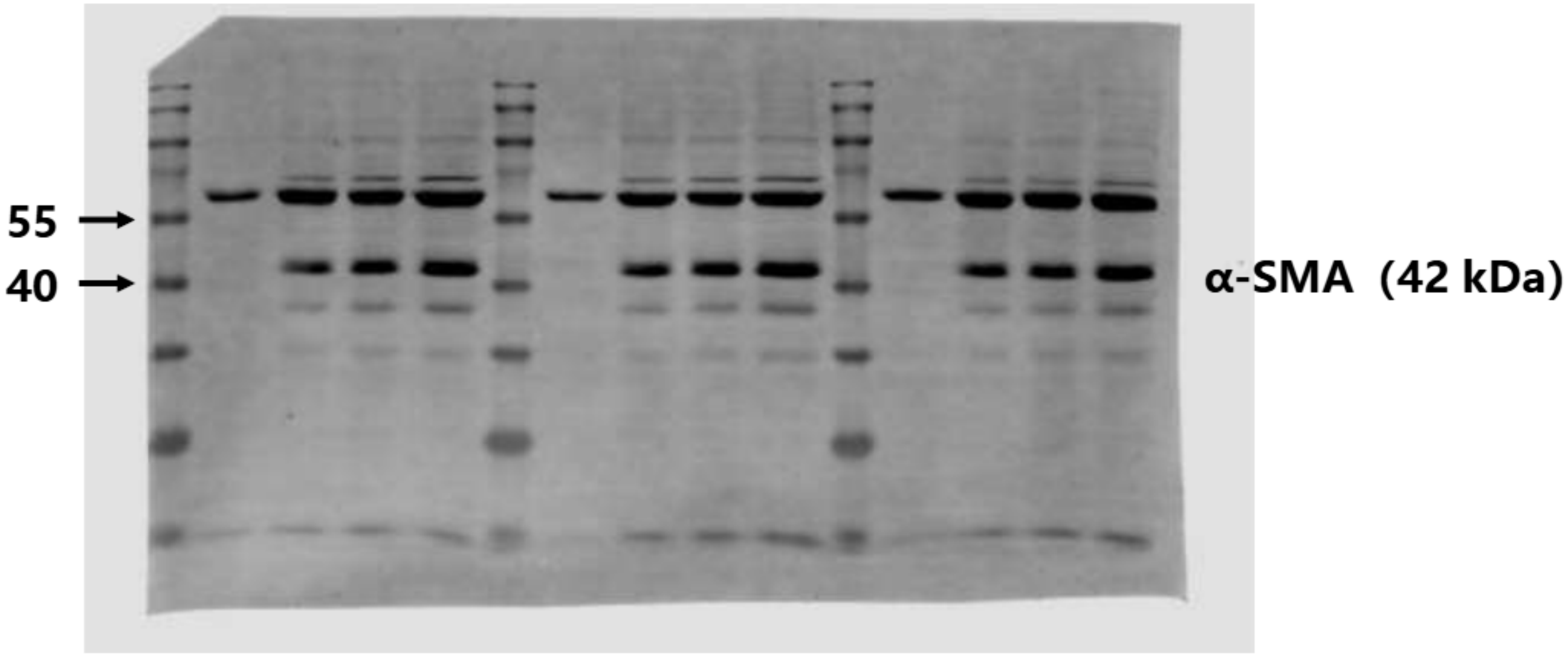

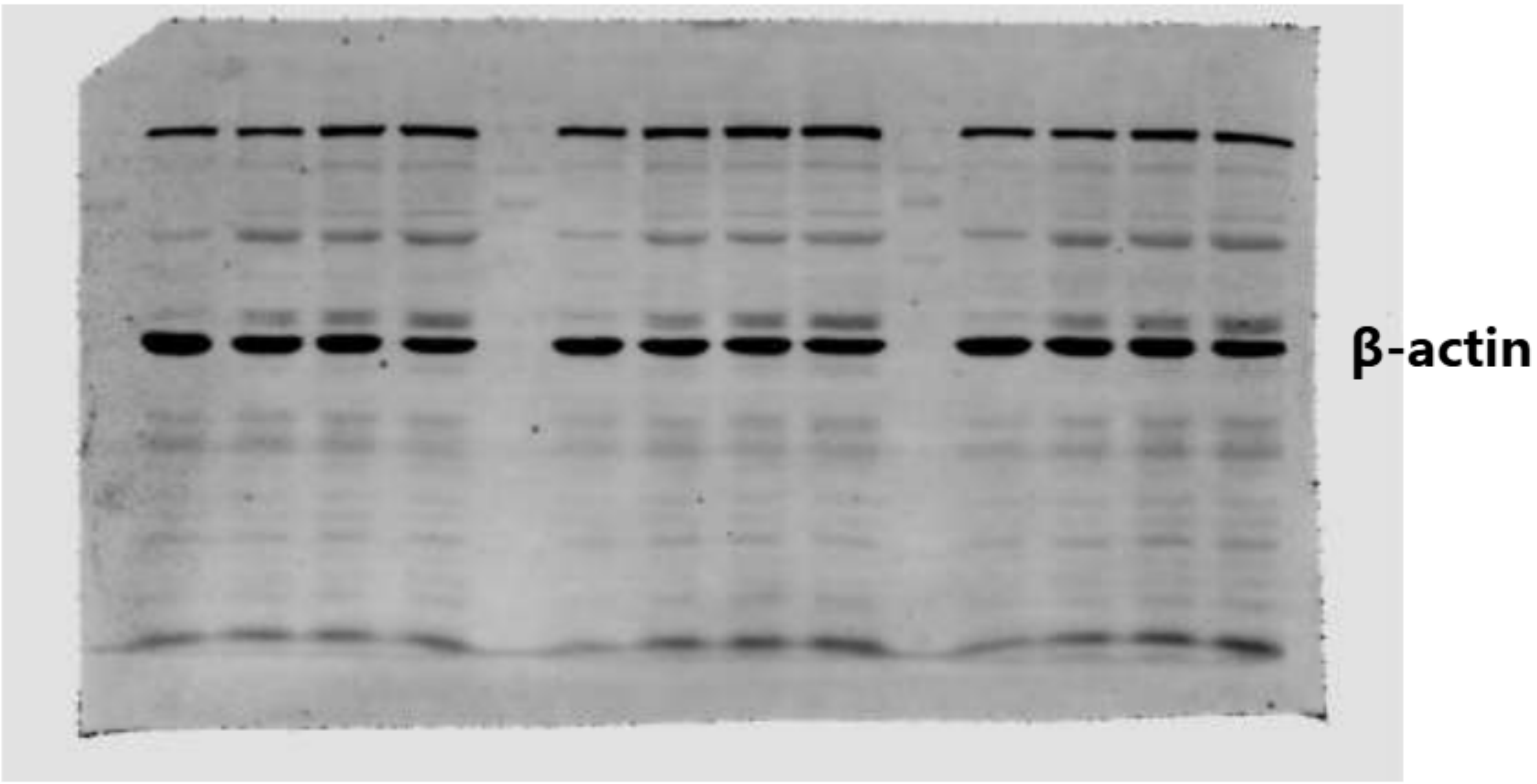

Lanes 12-15 of the unedited blot correspond to those shown in the cropped images within the manuscript.

Full unedited blot for Figure 3E

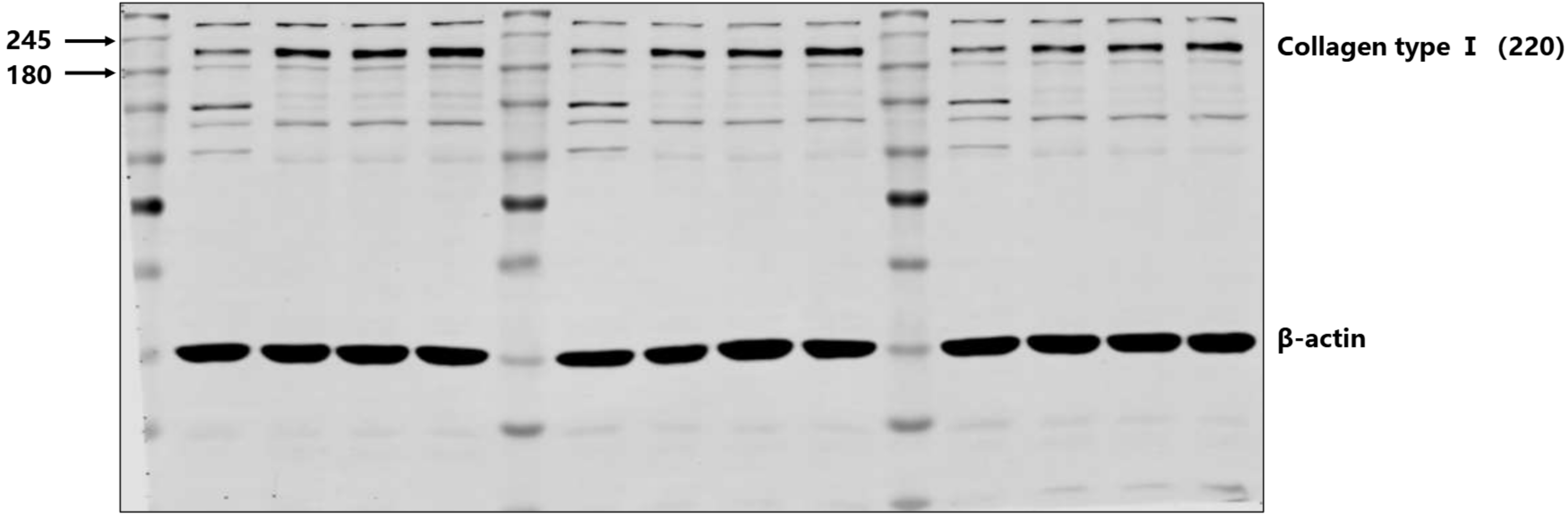

Lanes 2-5 of the unedited blot correspond to those shown in the cropped images within the manuscript.

Full unedited blot for Figure 3E

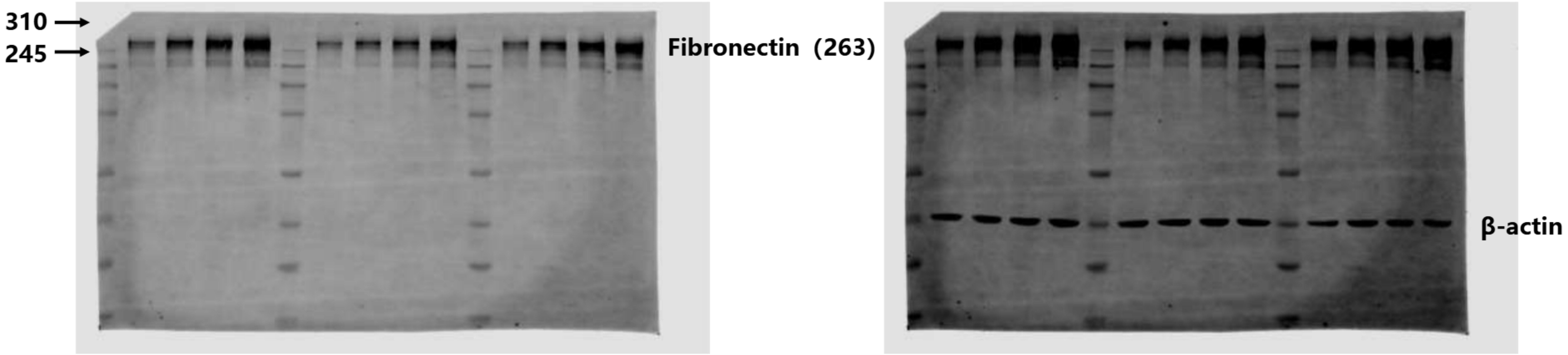

Lanes 12-15 of the unedited blot correspond to those shown in the cropped images within the manuscript.

Full unedited blot for Figure 3H

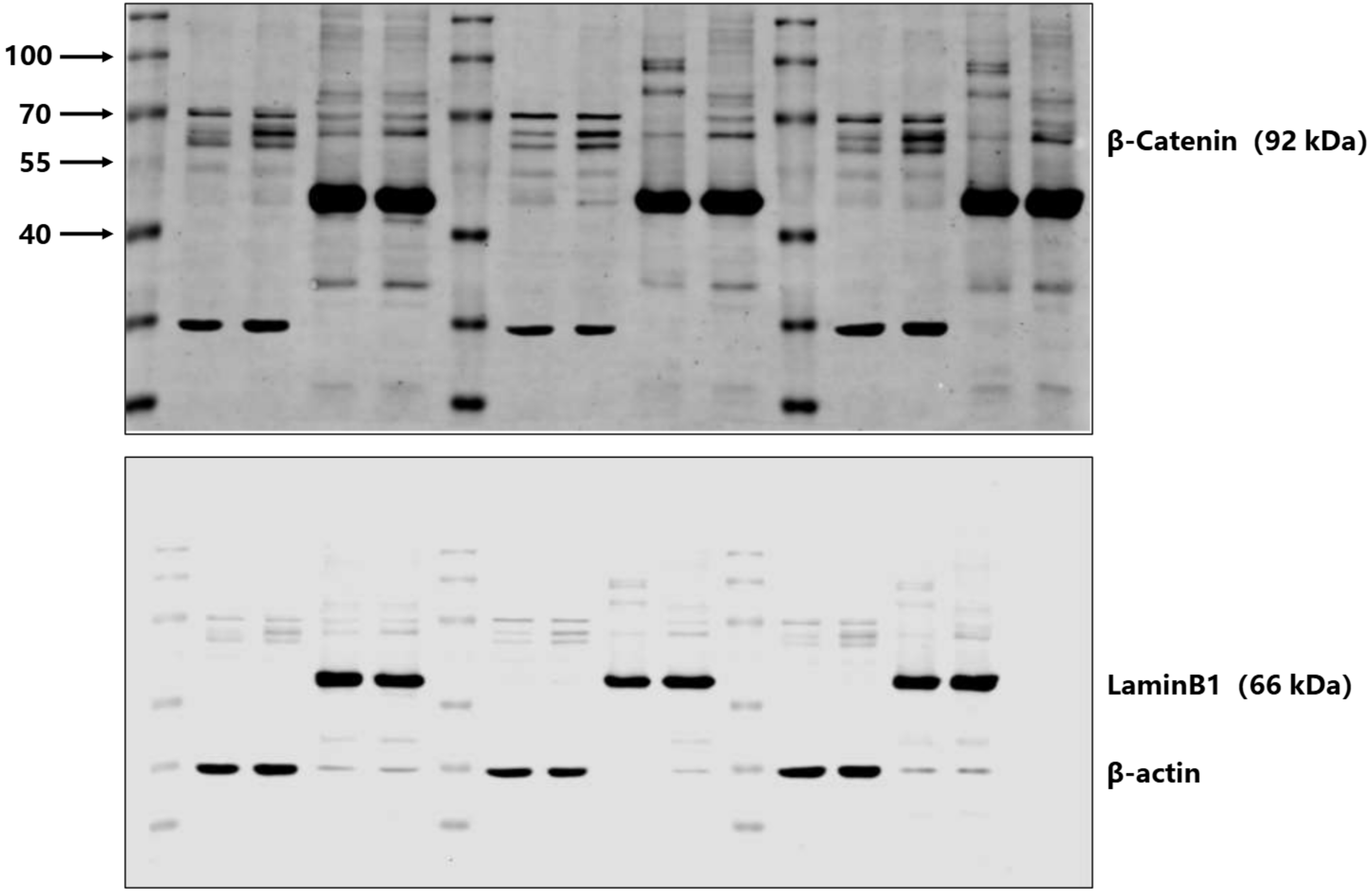

Lanes 12-15 of the unedited blot correspond to those shown in the cropped images within the manuscript.

Full unedited blot for Figure 3K

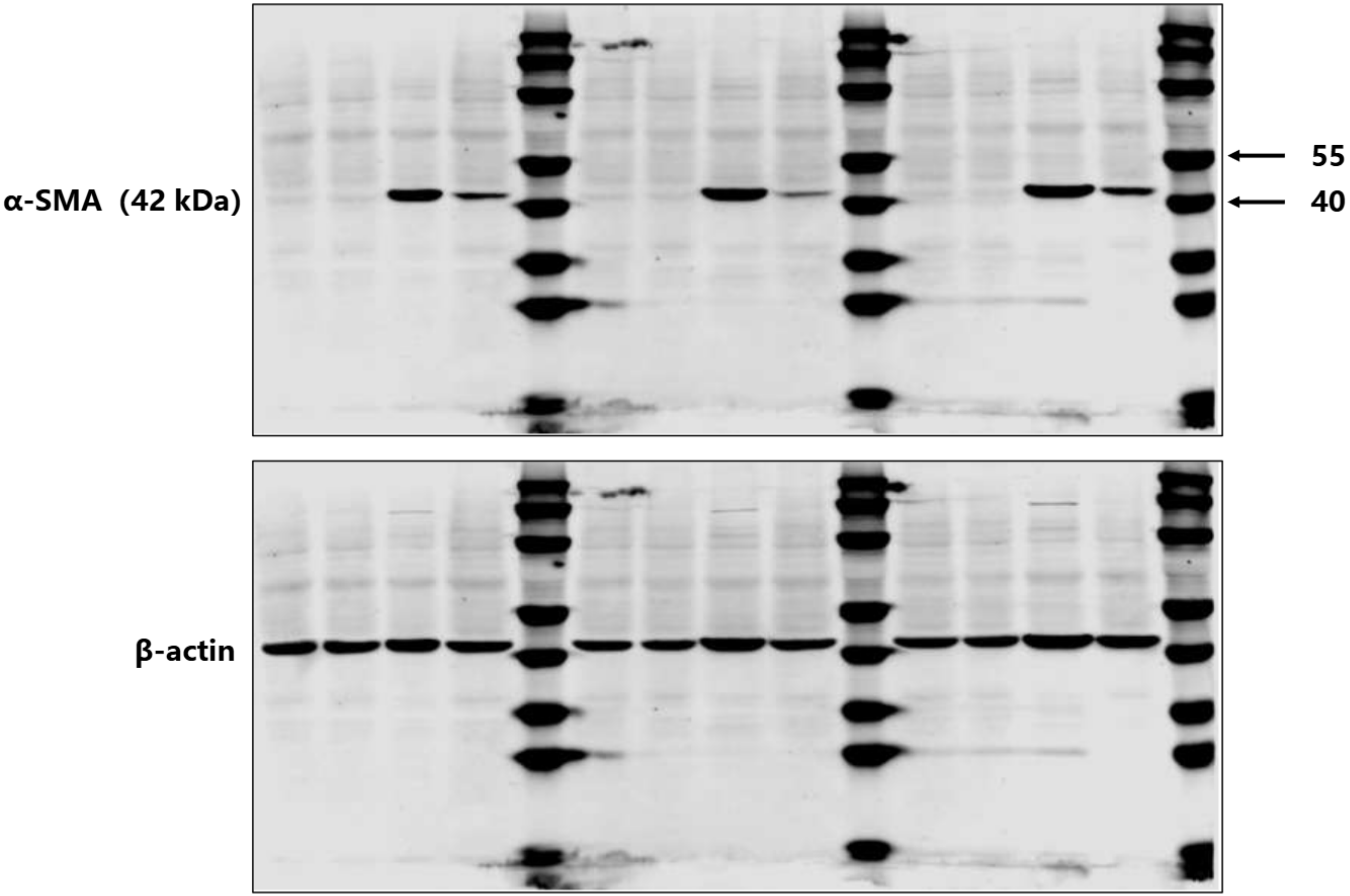

Lanes 6-9 of the unedited blot correspond to those shown in the cropped images within the manuscript.

Full unedited blot for Figure 3K

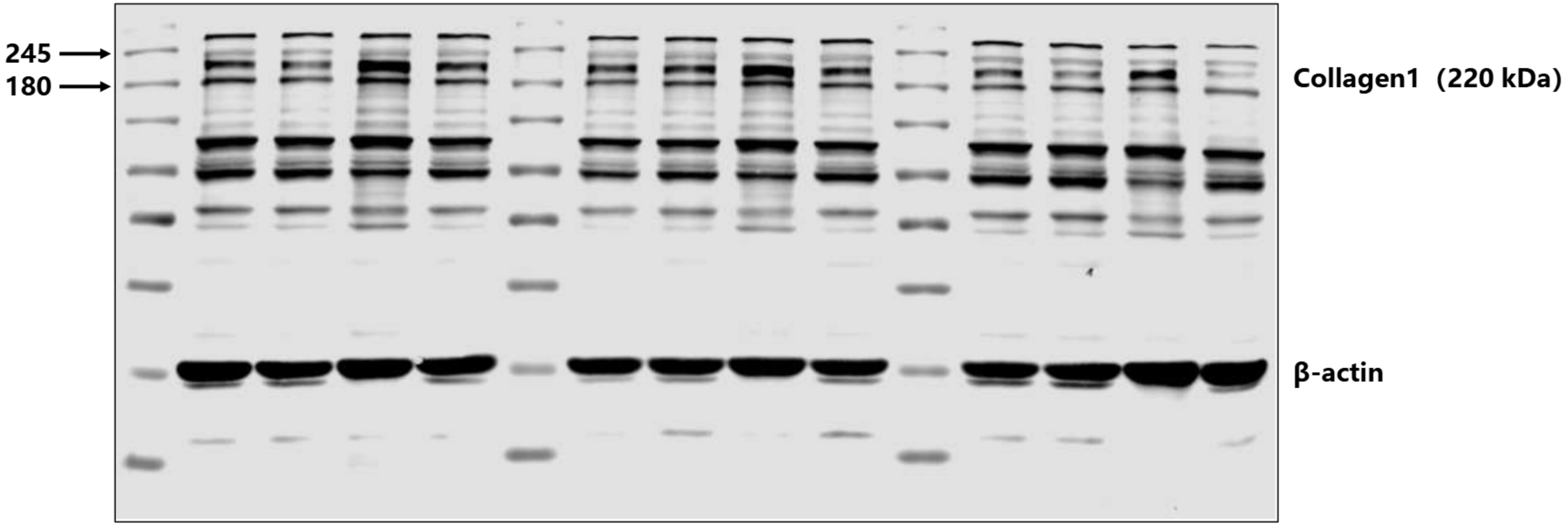

Lanes 7-10 of the unedited blot correspond to those shown in the cropped images within the manuscript.

Full unedited blot for Figure 3K

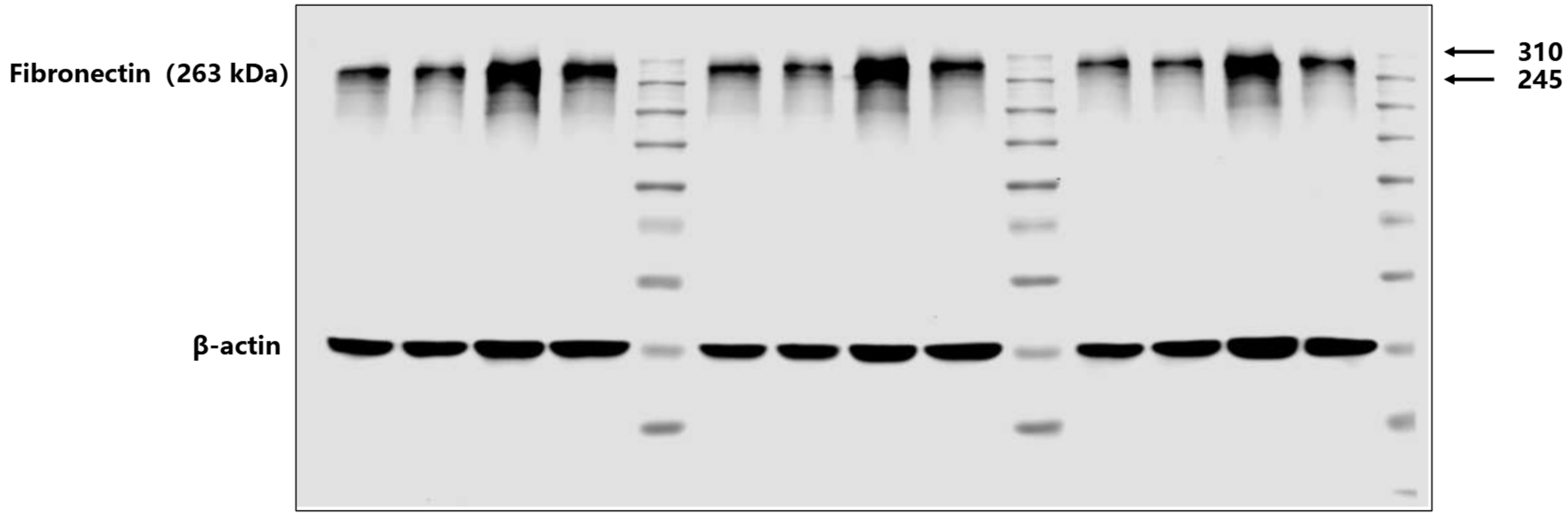

Lanes 6-9 of the unedited blot correspond to those shown in the cropped images within the manuscript.

Full unedited blot for Figure 3L

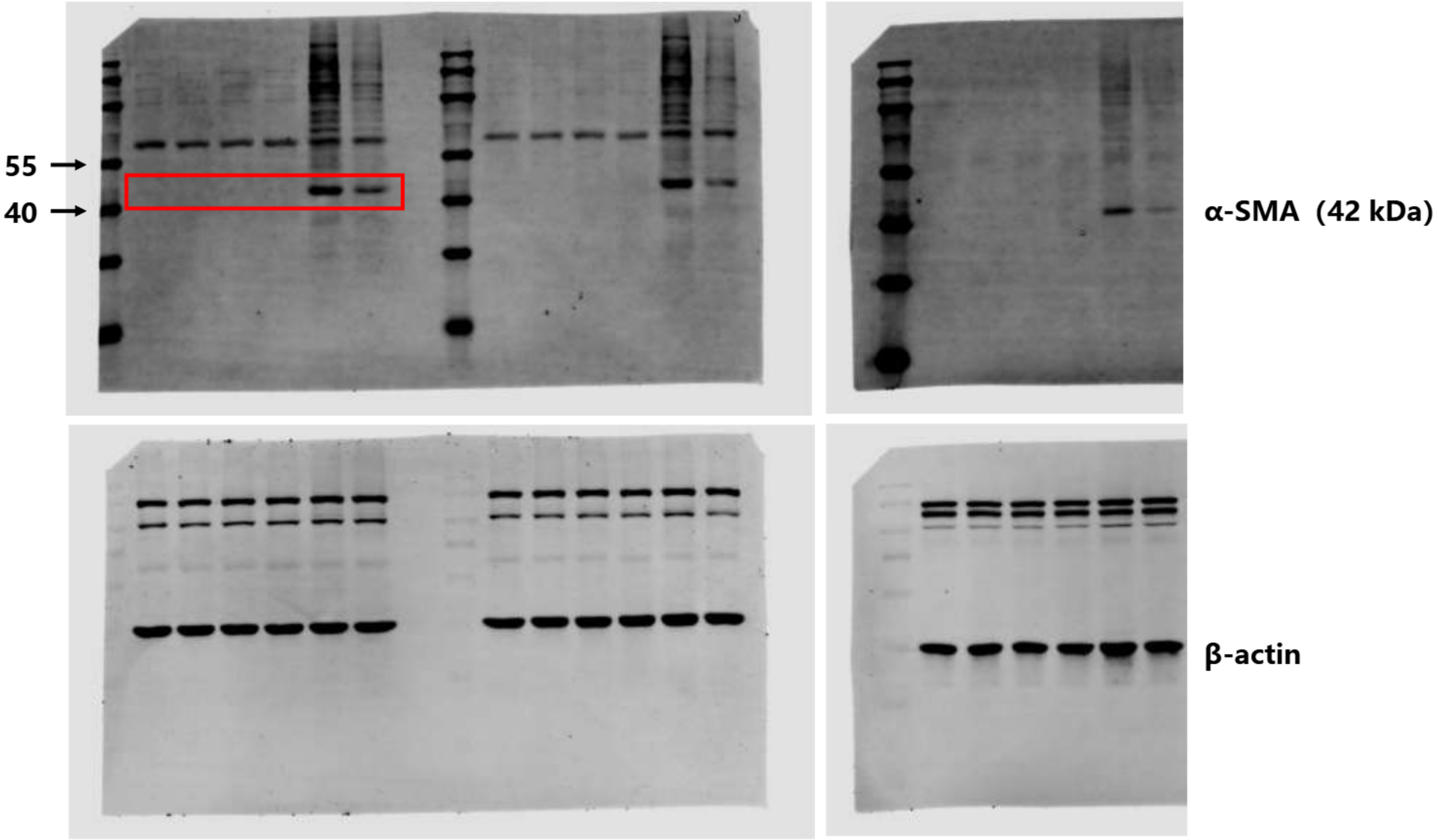

Lanes of the unedited blot correspond to those shown in the cropped images within the manuscript.

Full unedited blot for Figure 3L

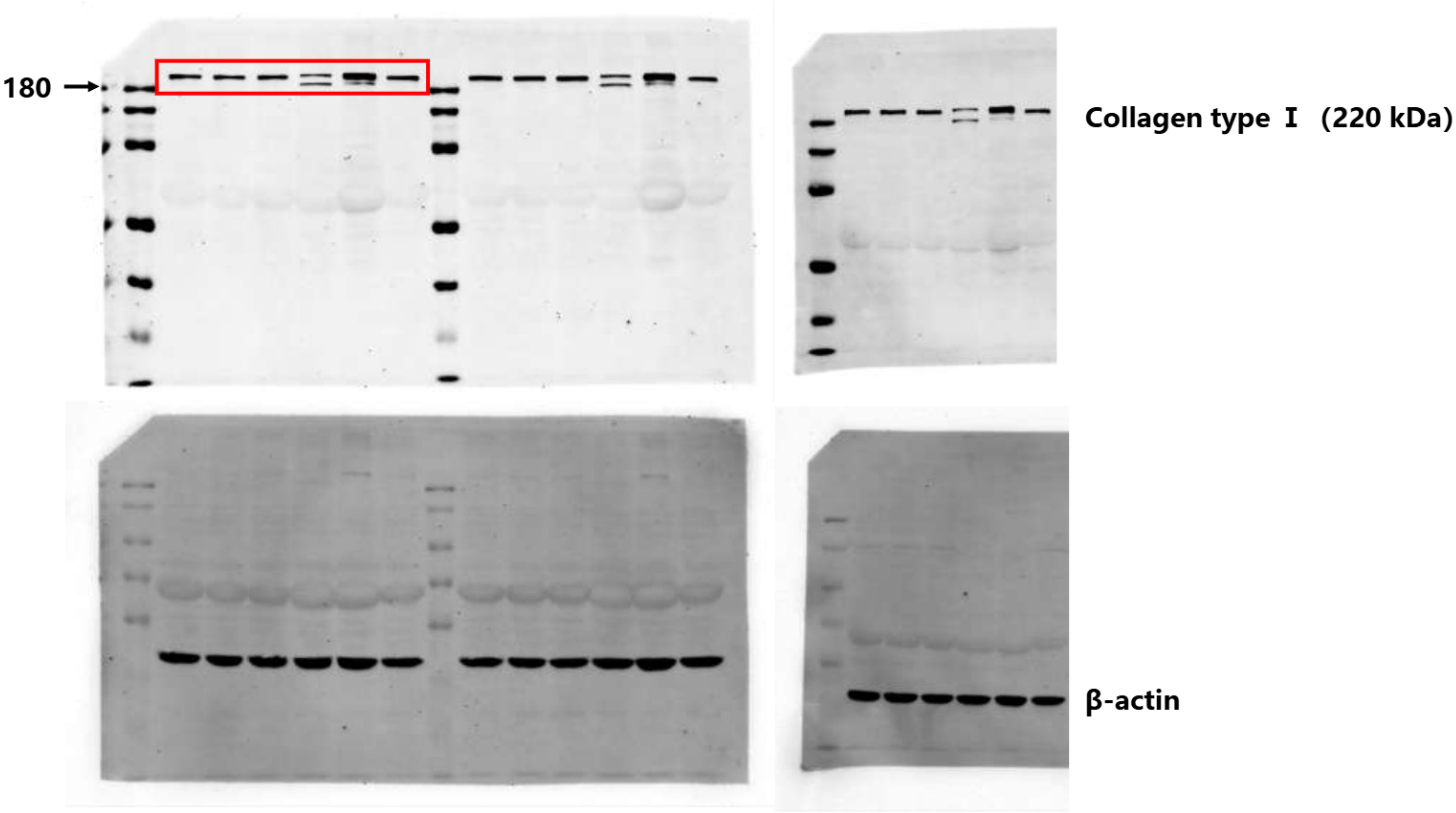

Lanes of the unedited blot correspond to those shown in the cropped images within the manuscript.

Full unedited blot for Figure 3L

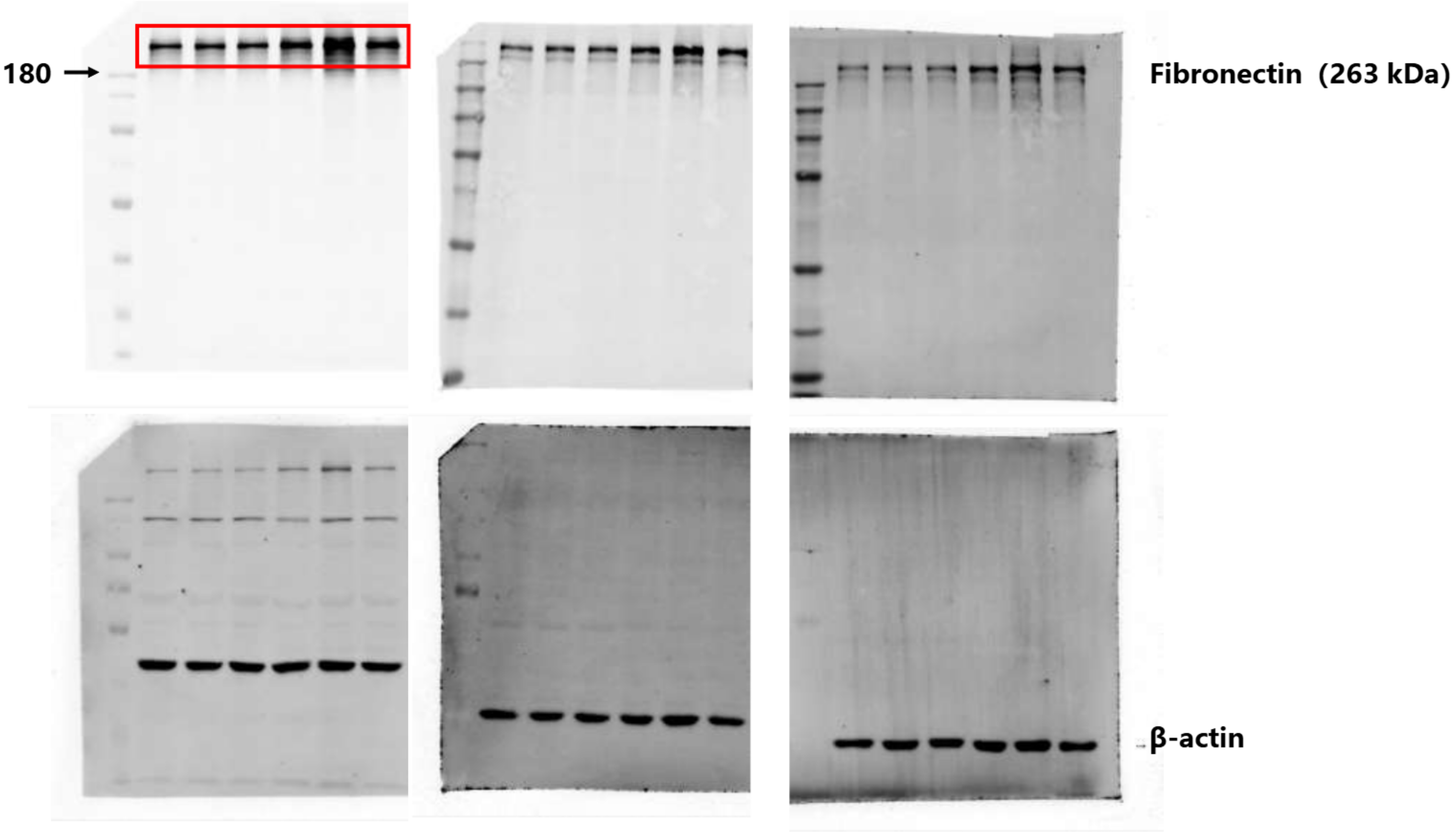

Lanes of the unedited blot correspond to those shown in the cropped images within the manuscript.

Full unedited blot for Figure 4A

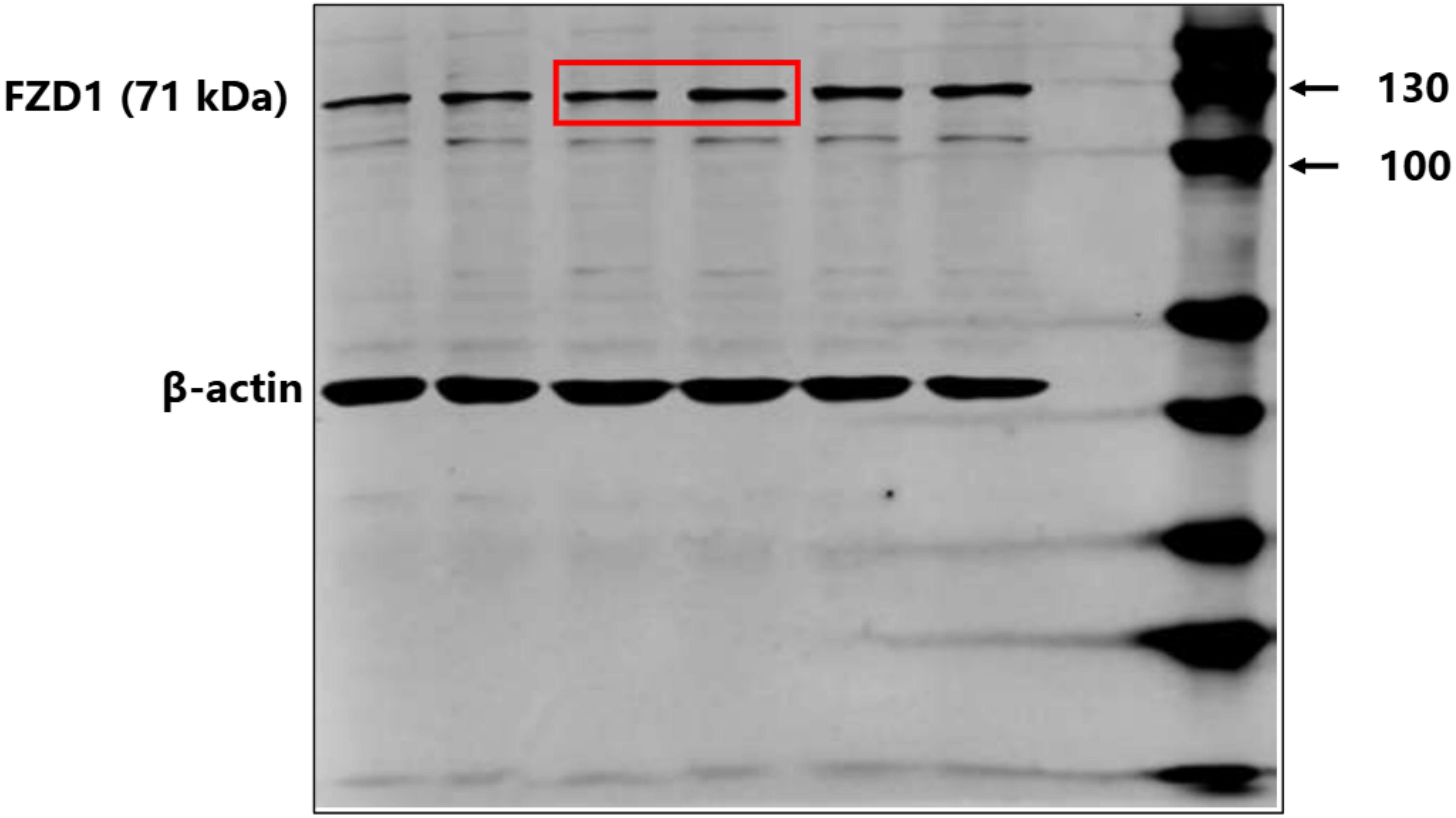

Lanes of the unedited blot correspond to those shown in the cropped images within the manuscript.

Full unedited blot for Figure 4A

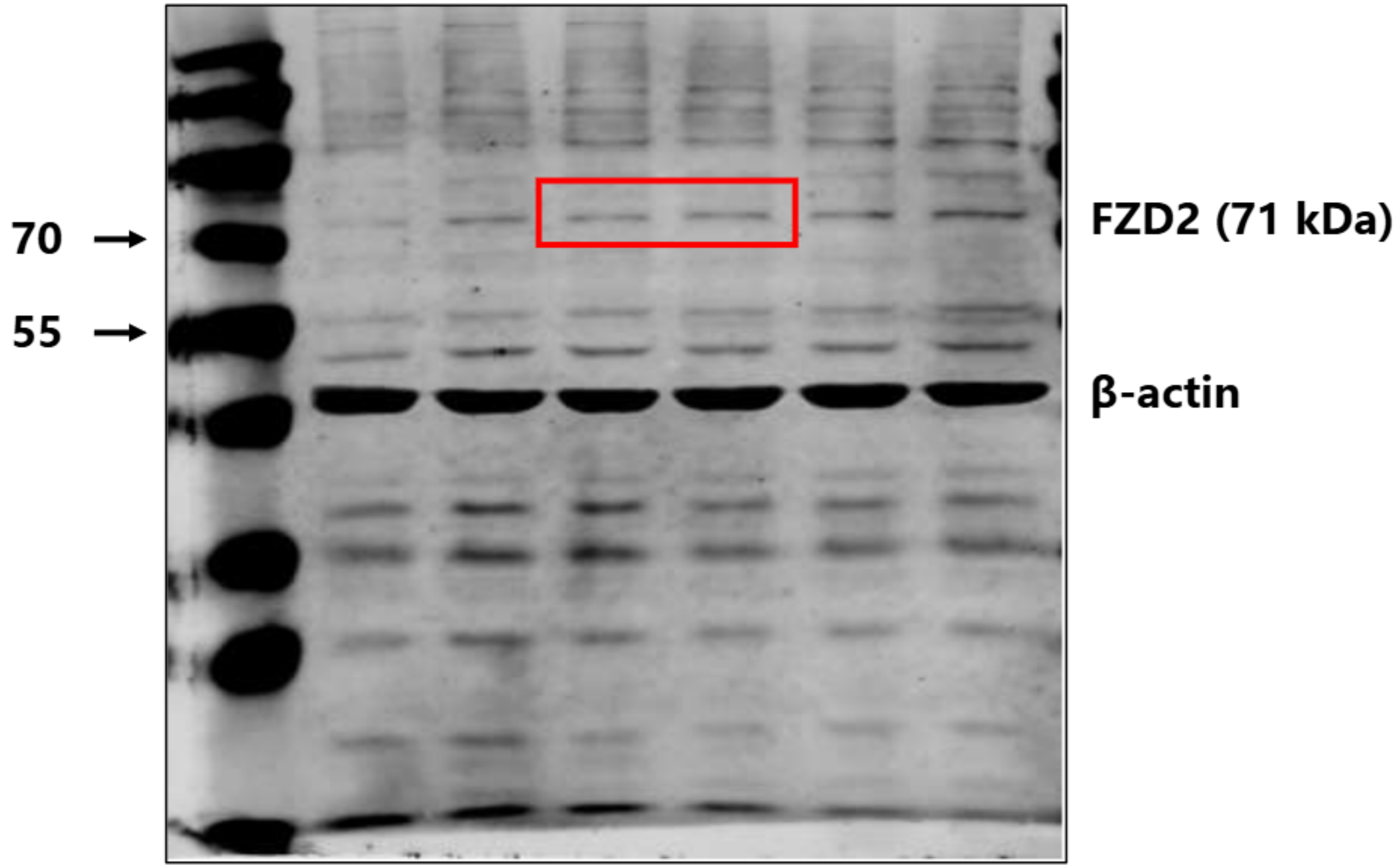

Lanes of the unedited blot correspond to those shown in the cropped images within the manuscript.

Full unedited blot for Figure 4B

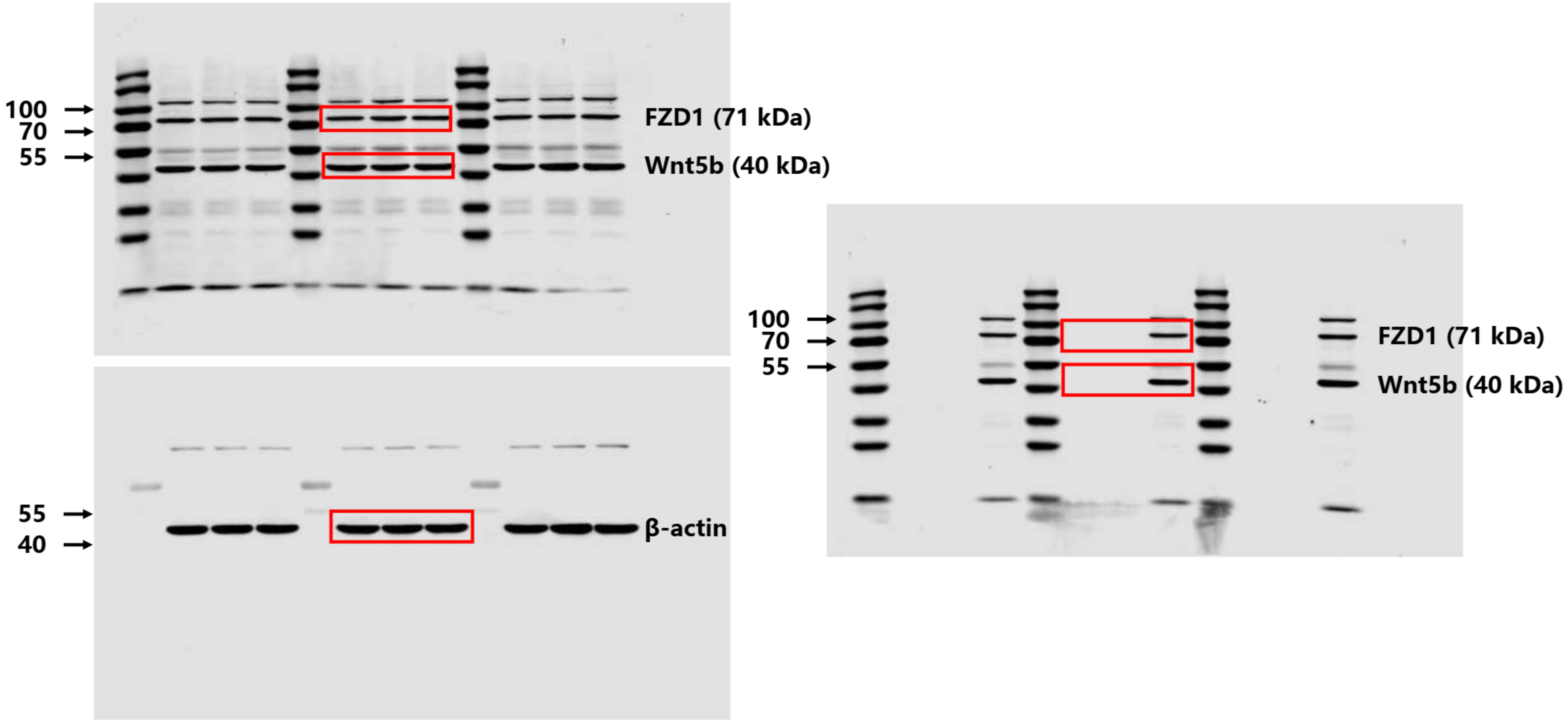

Lanes of the unedited blot correspond to those shown in the cropped images within the manuscript.

Full unedited blot for Figure 4B

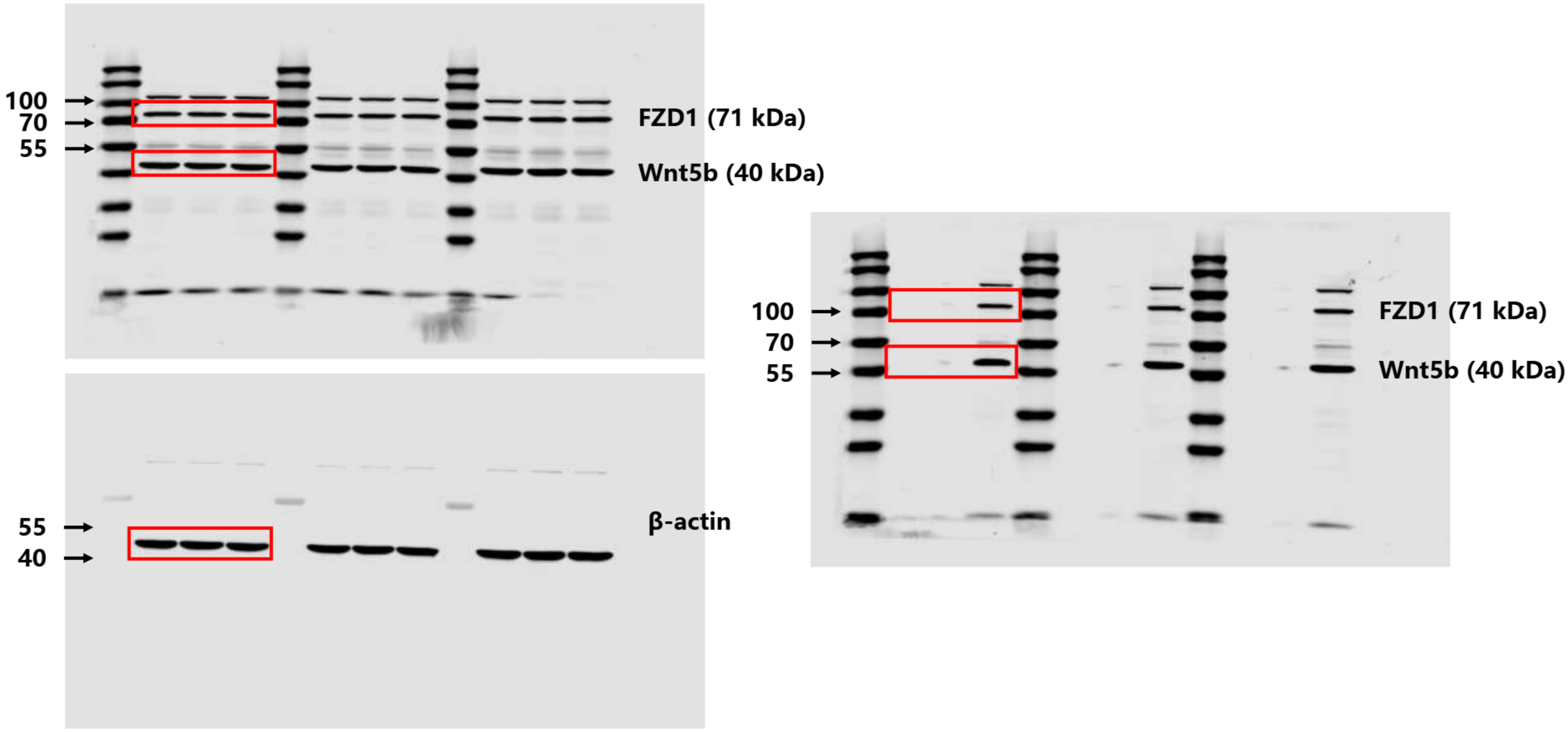

Lanes of the unedited blot correspond to those shown in the cropped images within the manuscript.

Full unedited blot for Figure 4C

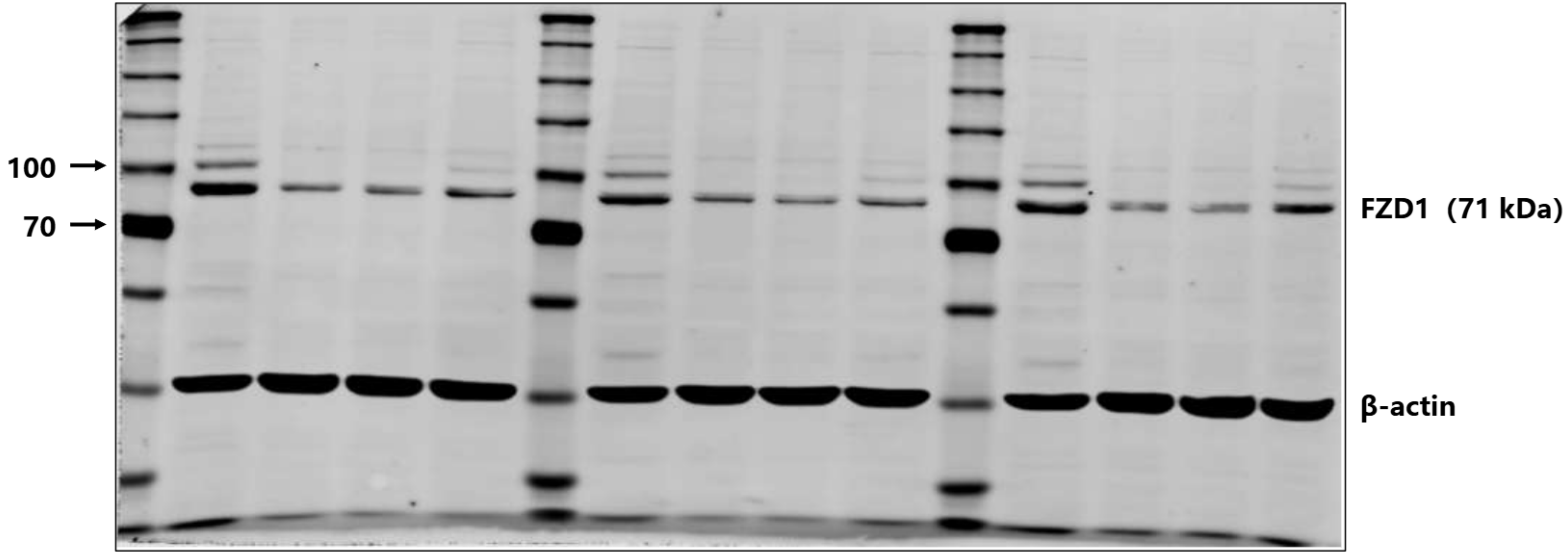

Lanes 2-5 of the unedited blot correspond to those shown in the cropped images within the manuscript.

Full unedited blot for Figure 4D

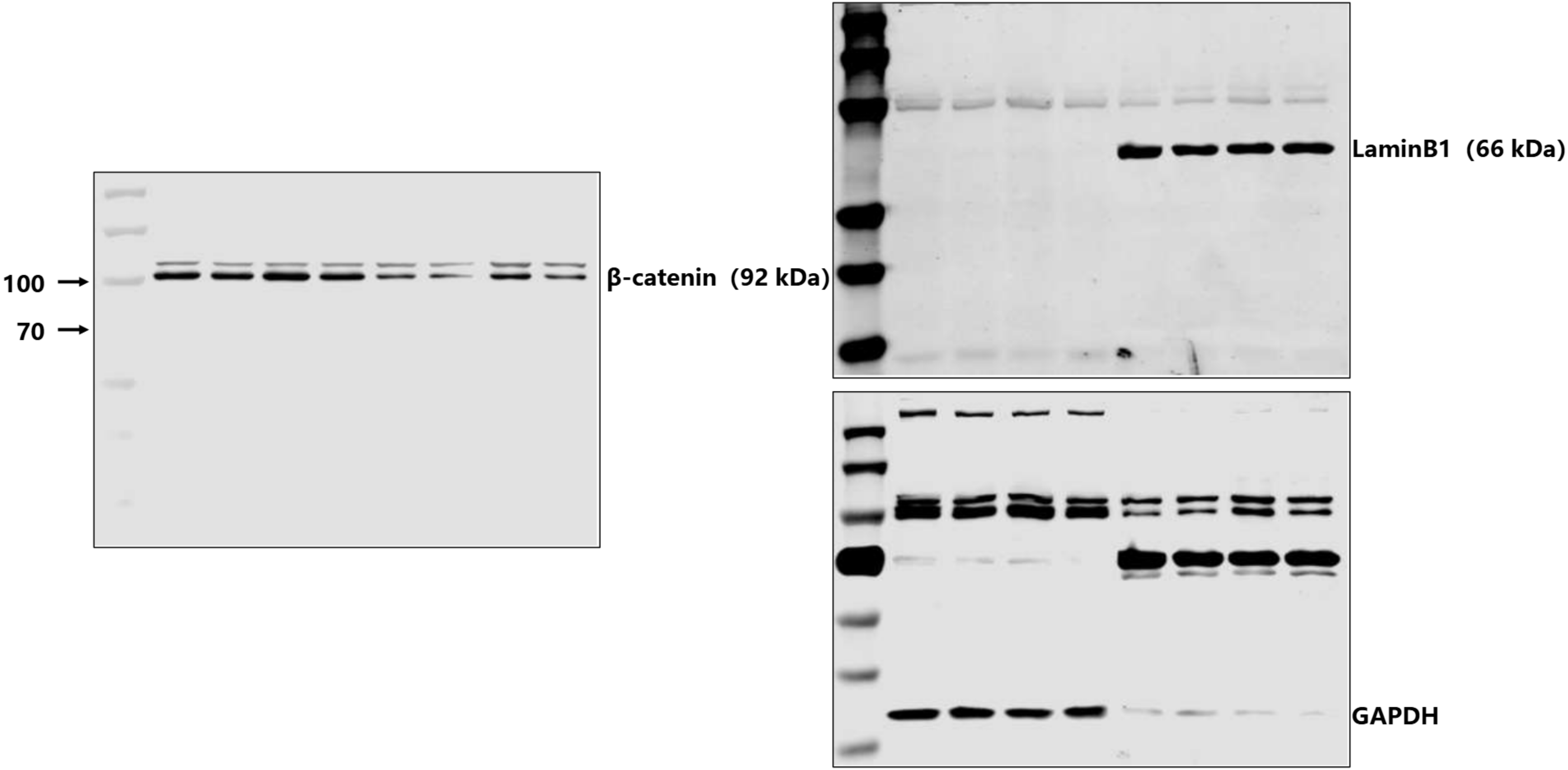

Lanes 2-9 of the unedited blot correspond to those shown in the cropped images within the manuscript.

Full unedited blot for Figure 4D

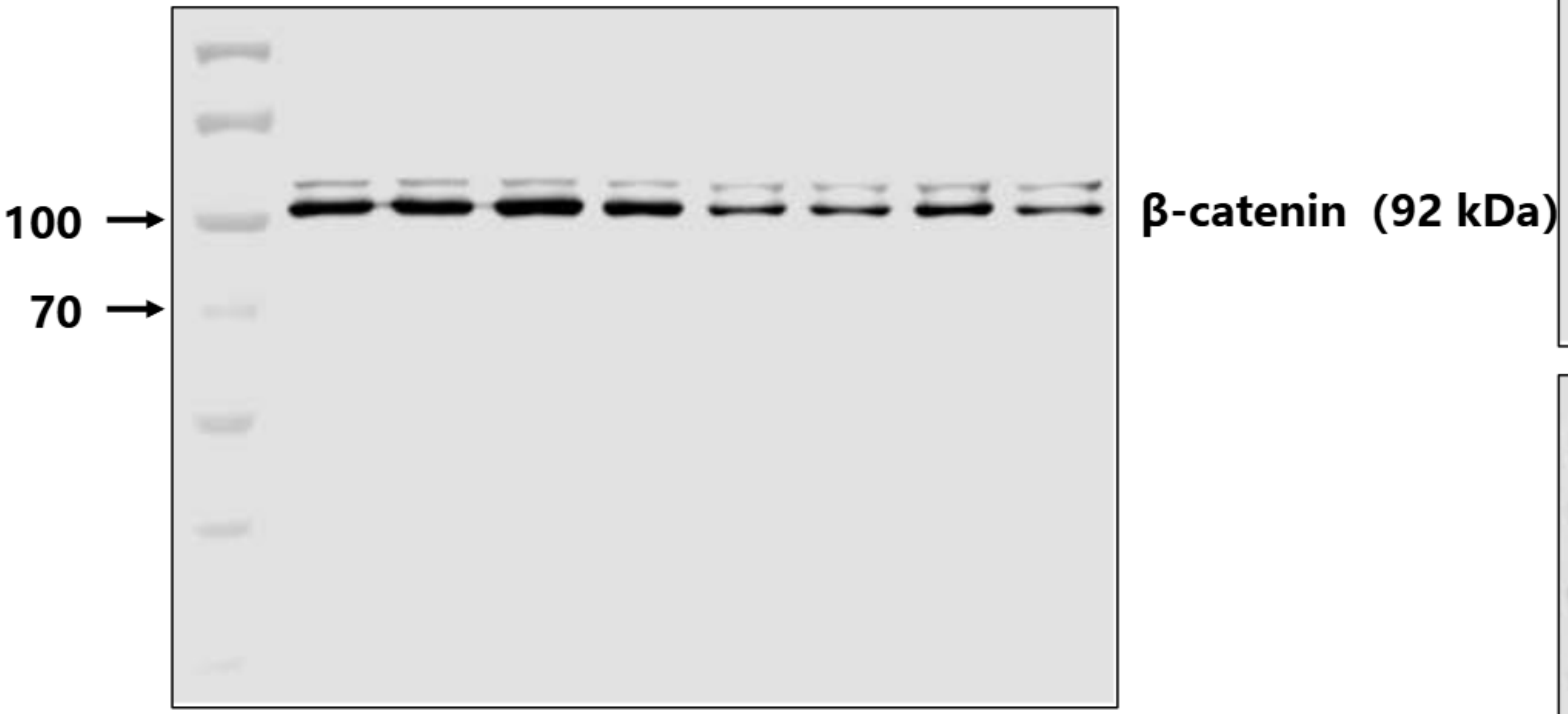

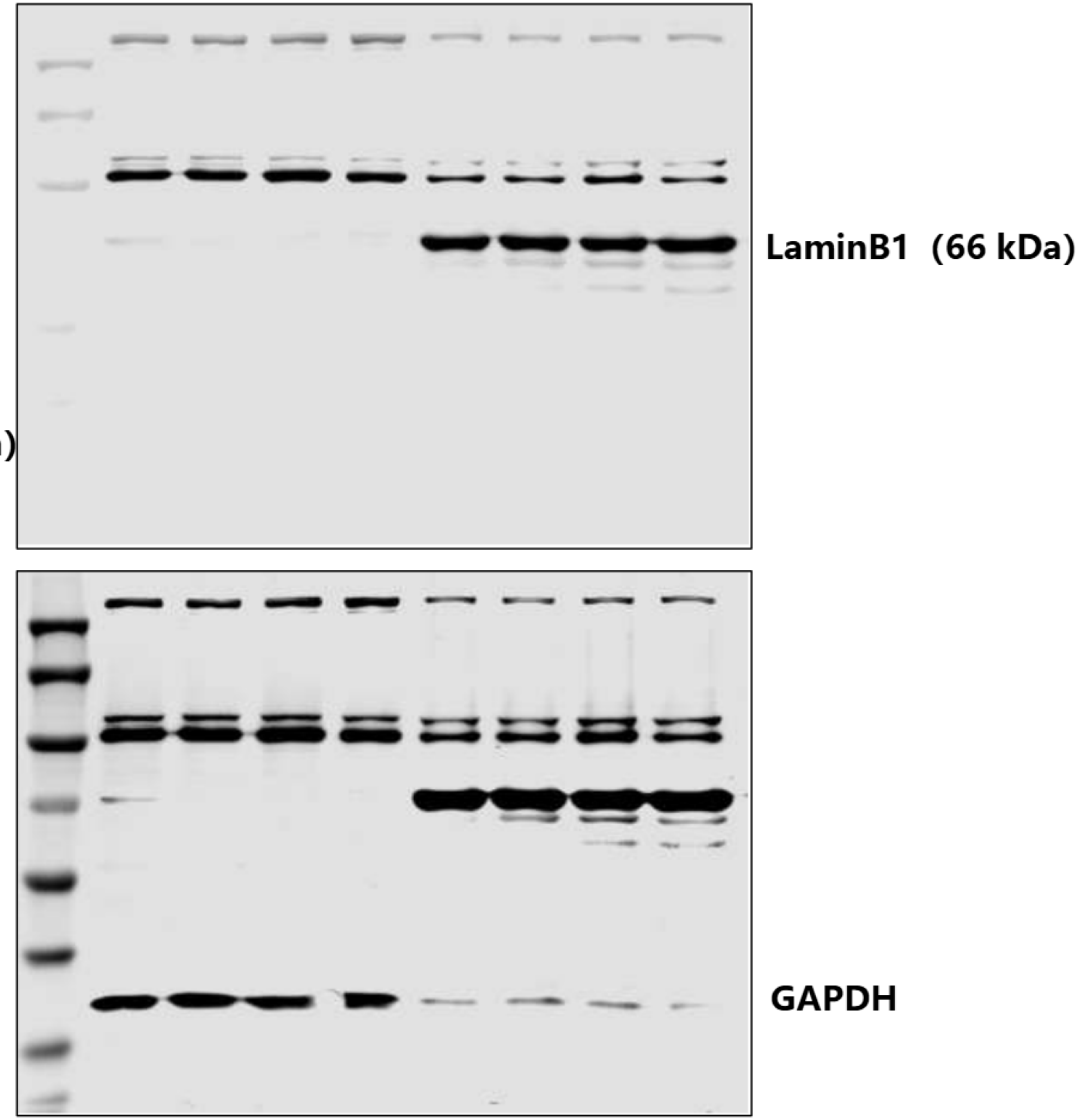

Full unedited blot for Figure 4D

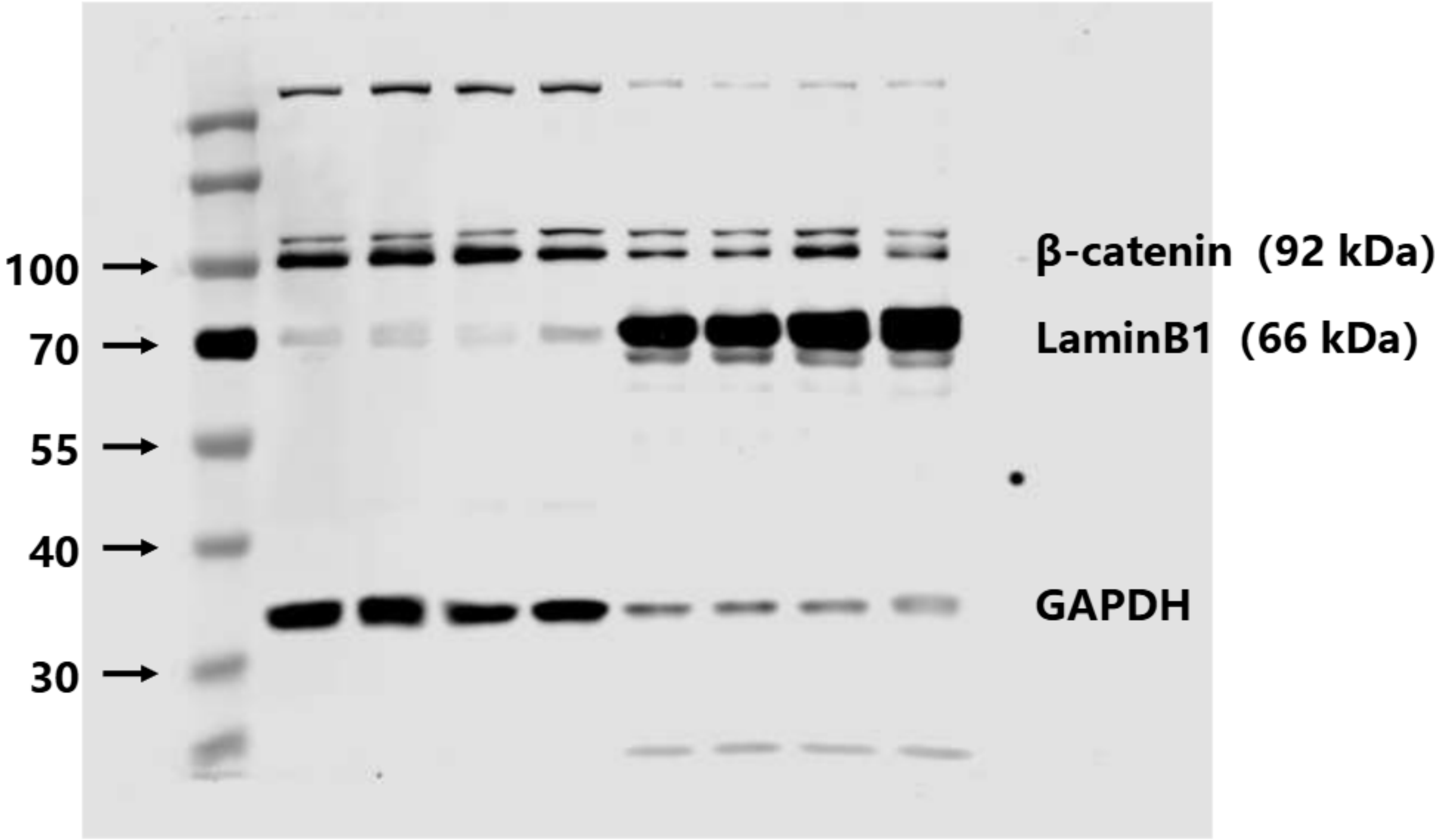

Full unedited blot for Figure 4E

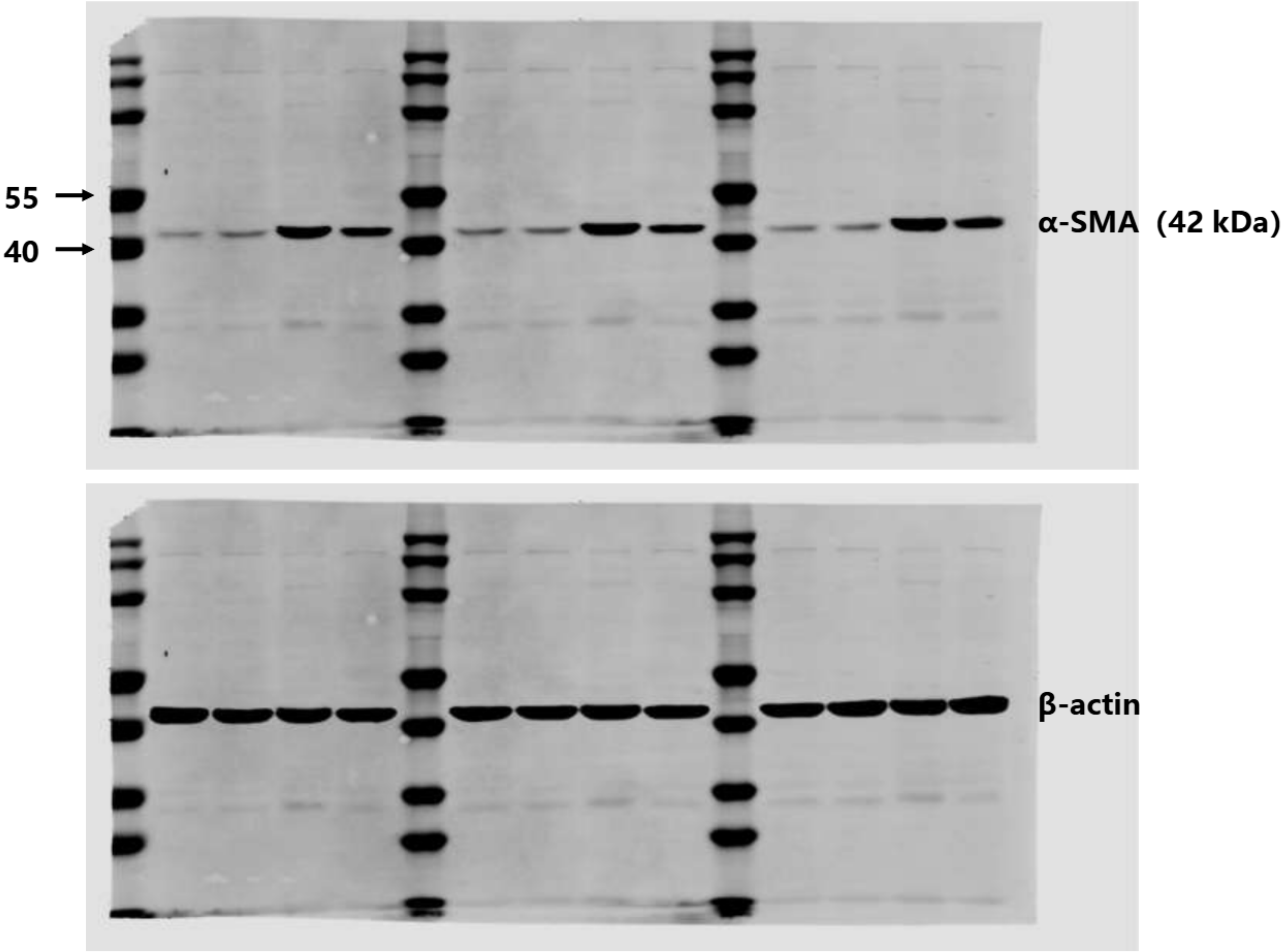

Lanes 2-5 of the unedited blot correspond to those shown in the cropped images within the manuscript.

Full unedited blot for Figure 4E

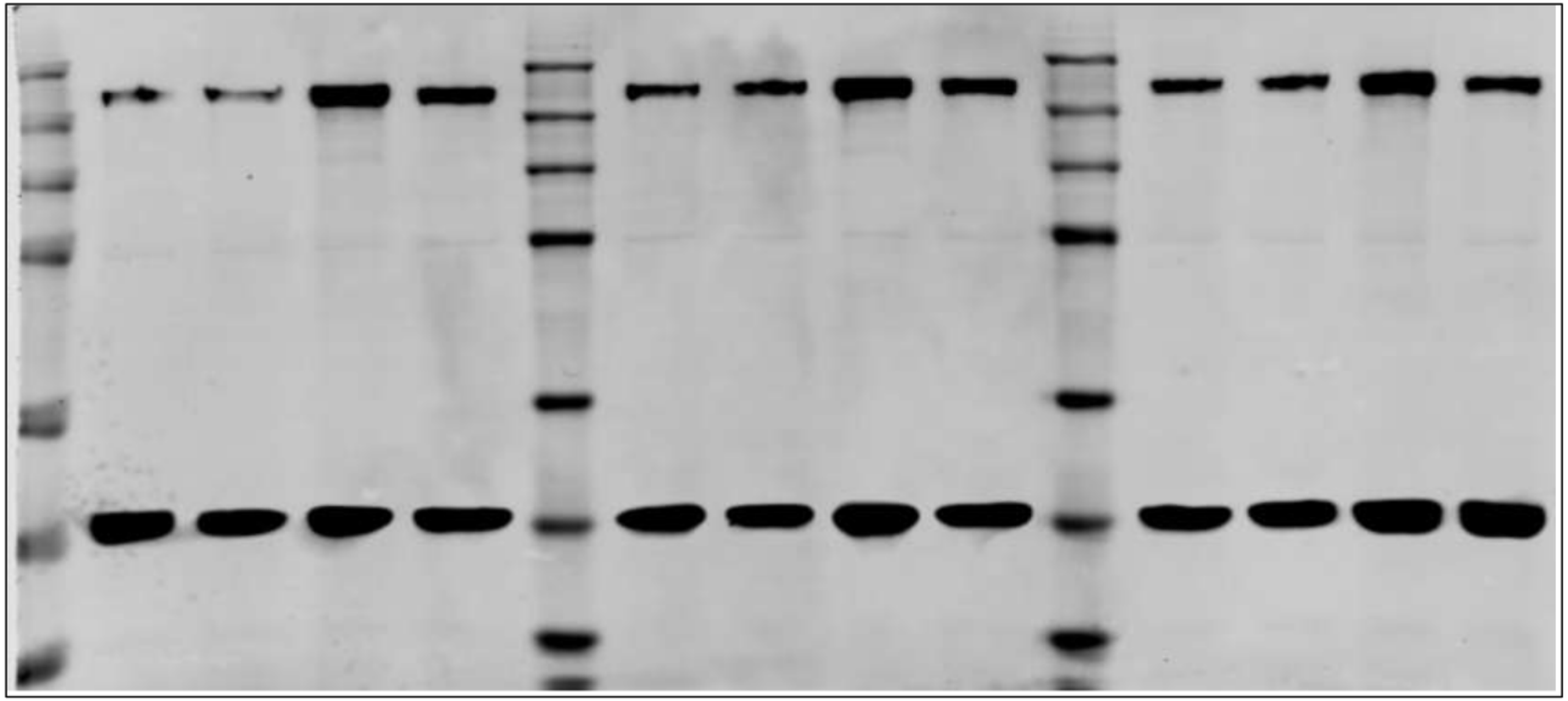

Lanes 12-15 of the unedited blot correspond to those shown in the cropped images within the manuscript.

Full unedited blot for Figure 4E

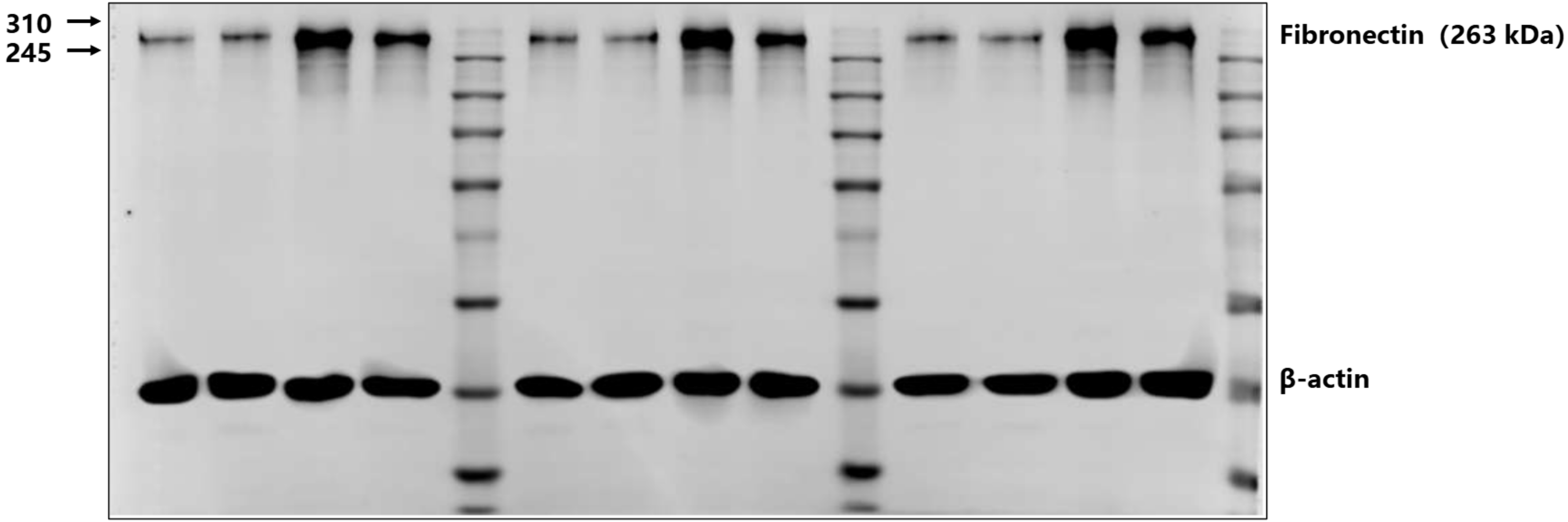

Lanes 2-5 of the unedited blot correspond to those shown in the cropped images within the manuscript.

Full unedited blot for Figure 4F

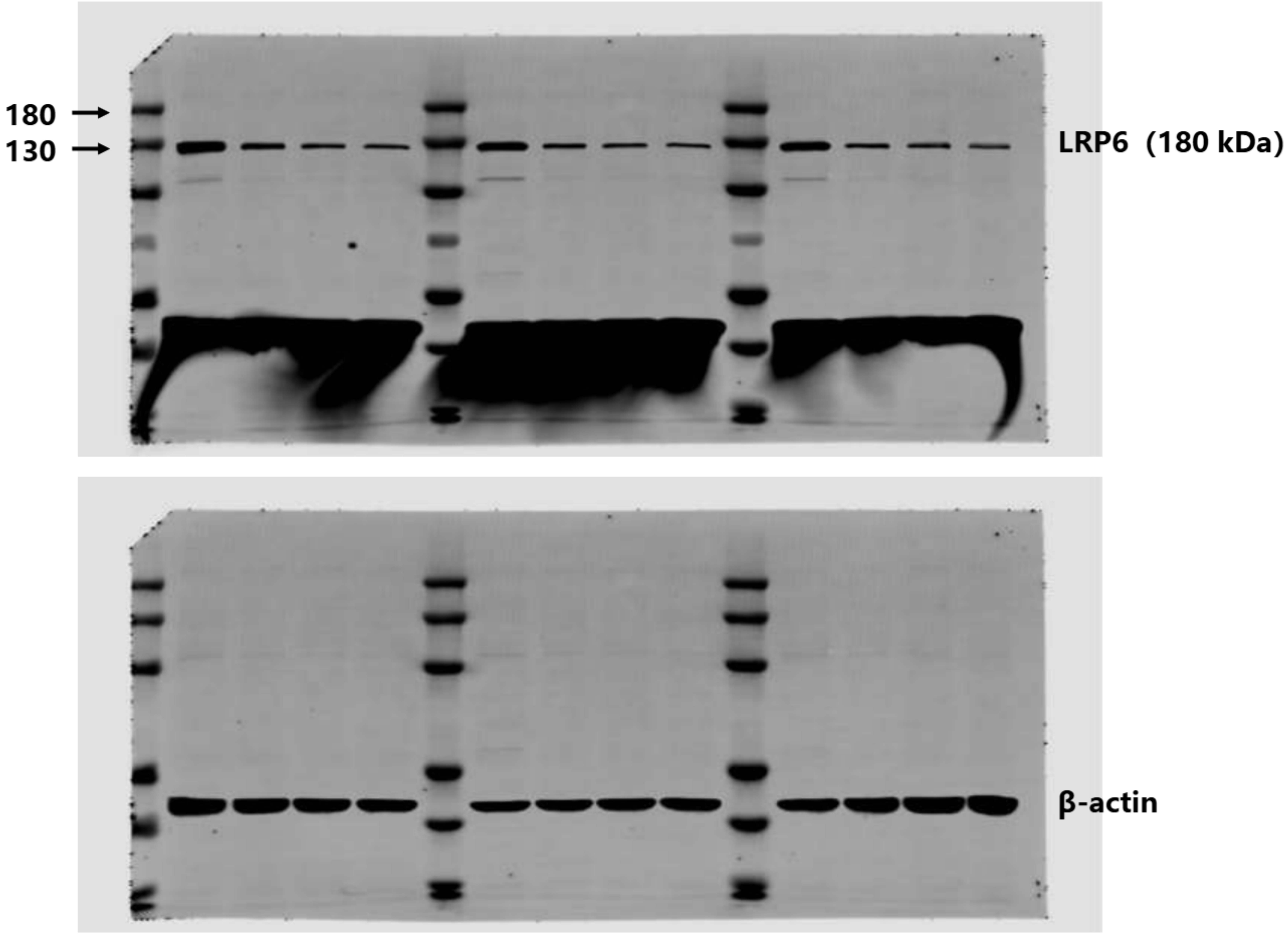

Lanes 7-10 of the unedited blot correspond to those shown in the cropped images within the manuscript.

Full unedited blot for Figure 4G

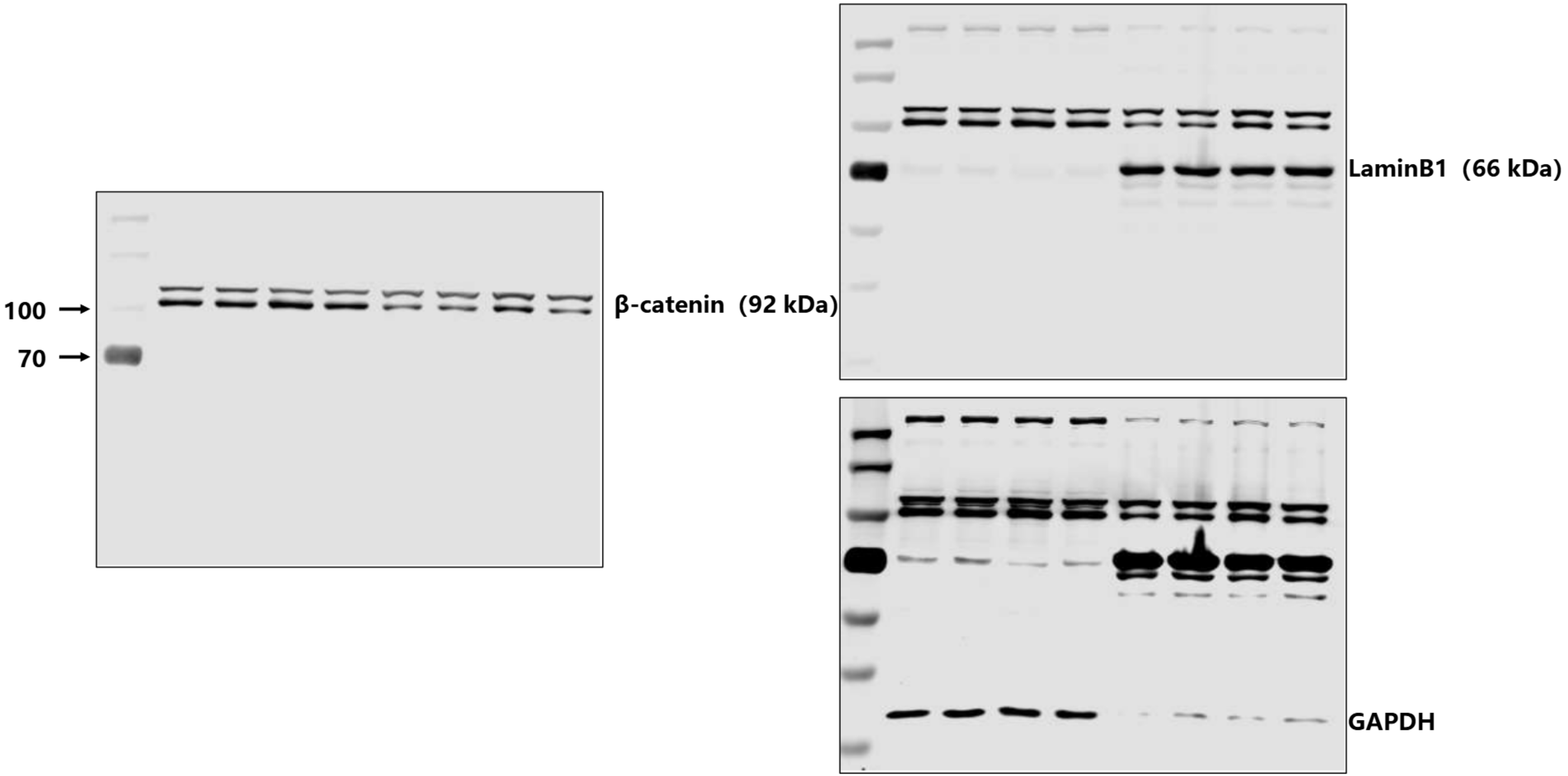

Lanes 2-9 of the unedited blot correspond to those shown in the cropped images within the manuscript.

Full unedited blot for Figure 4G

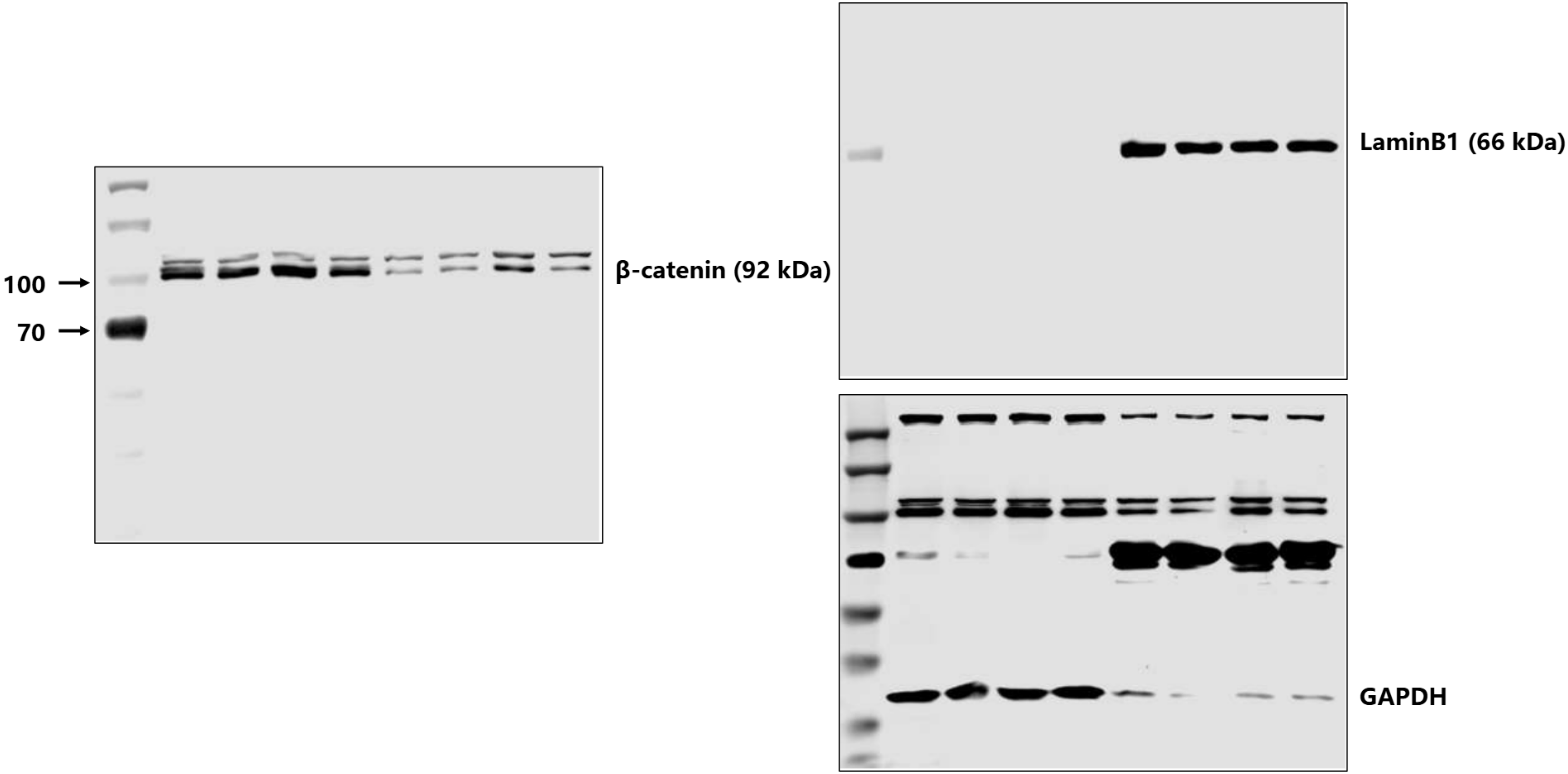

Full unedited blot for Figure 4G

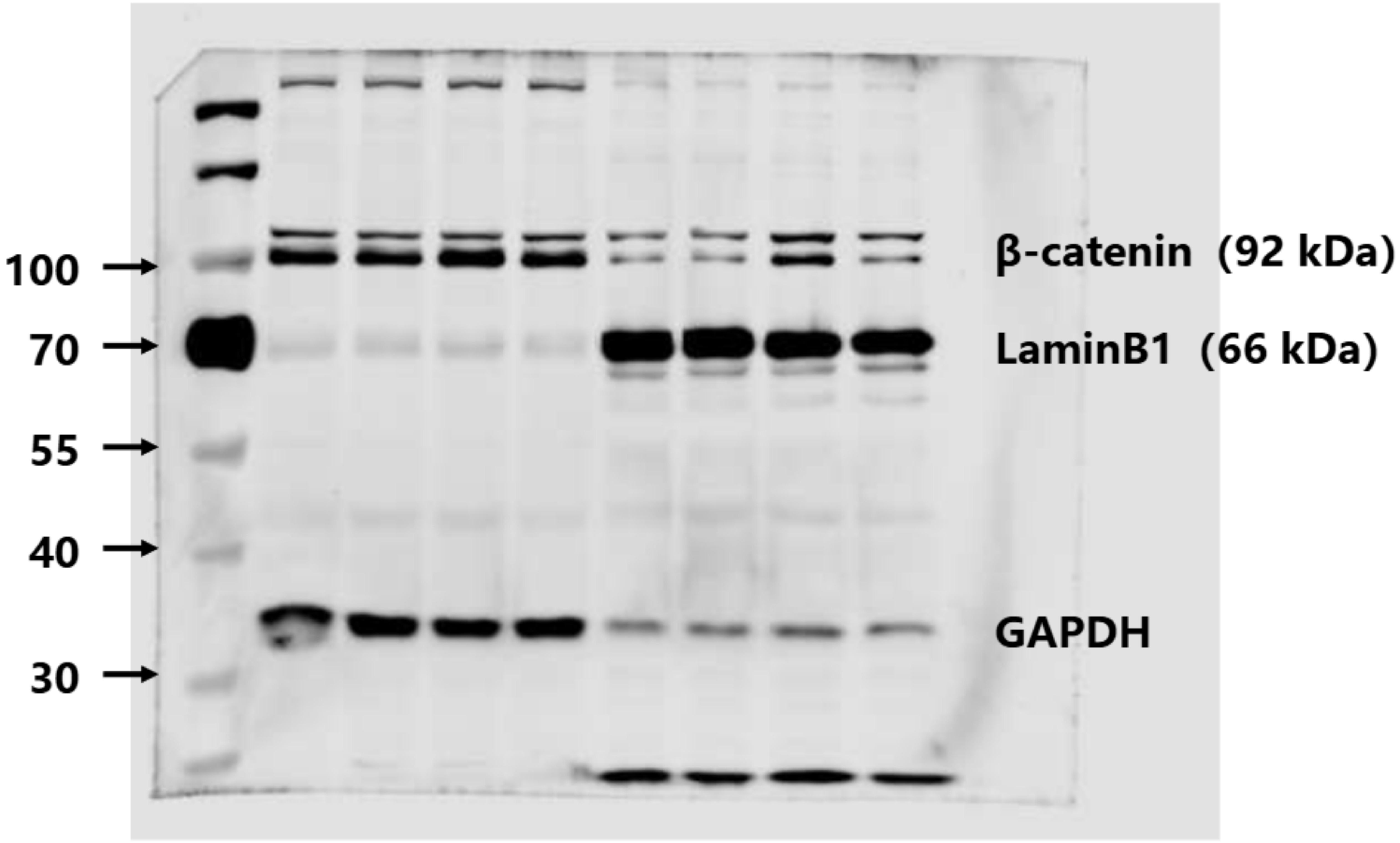

Full unedited blot for Figure 4H

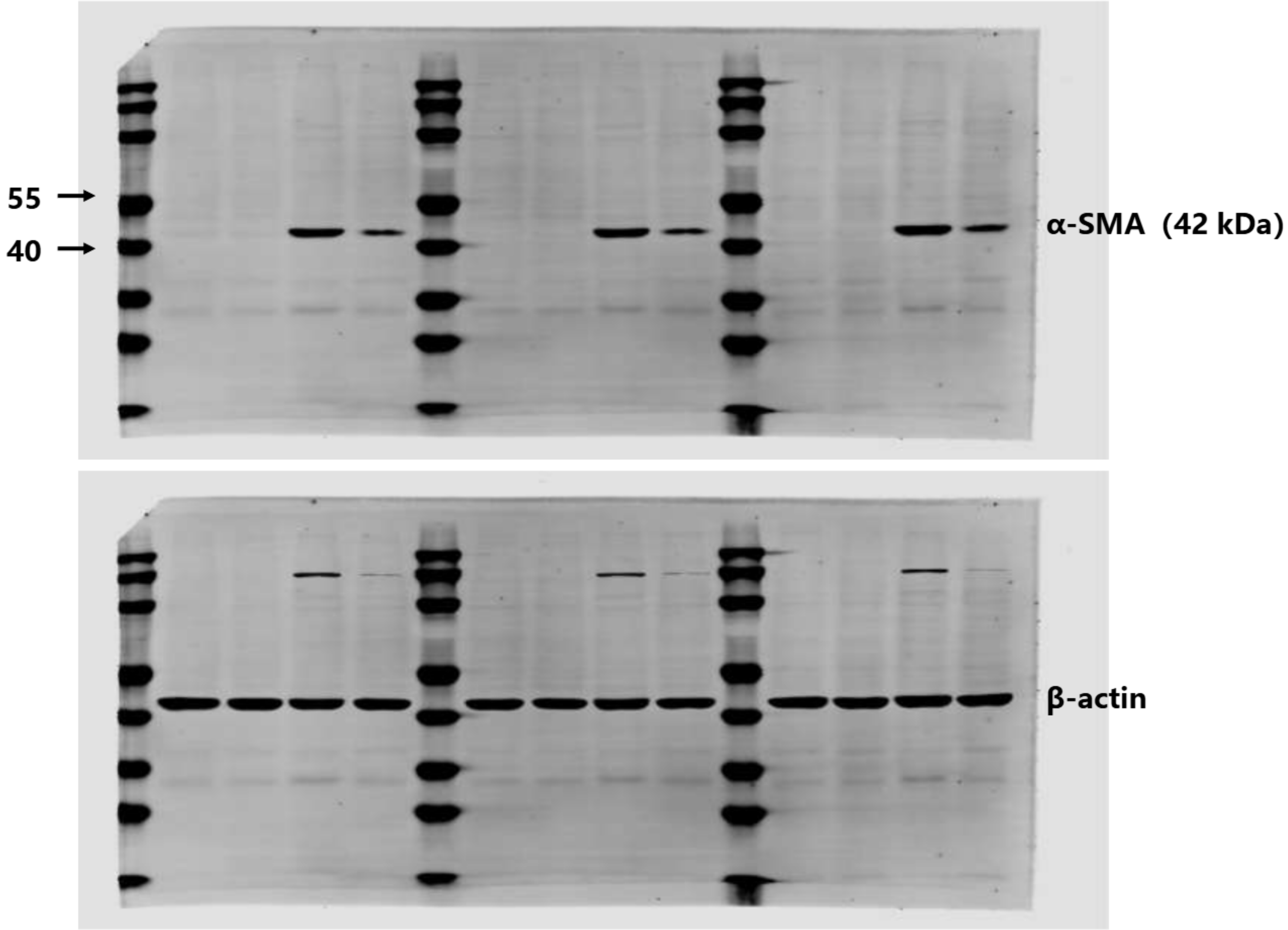

Lanes 2-5 of the unedited blot correspond to those shown in the cropped images within the manuscript.

Full unedited blot for Figure 4H

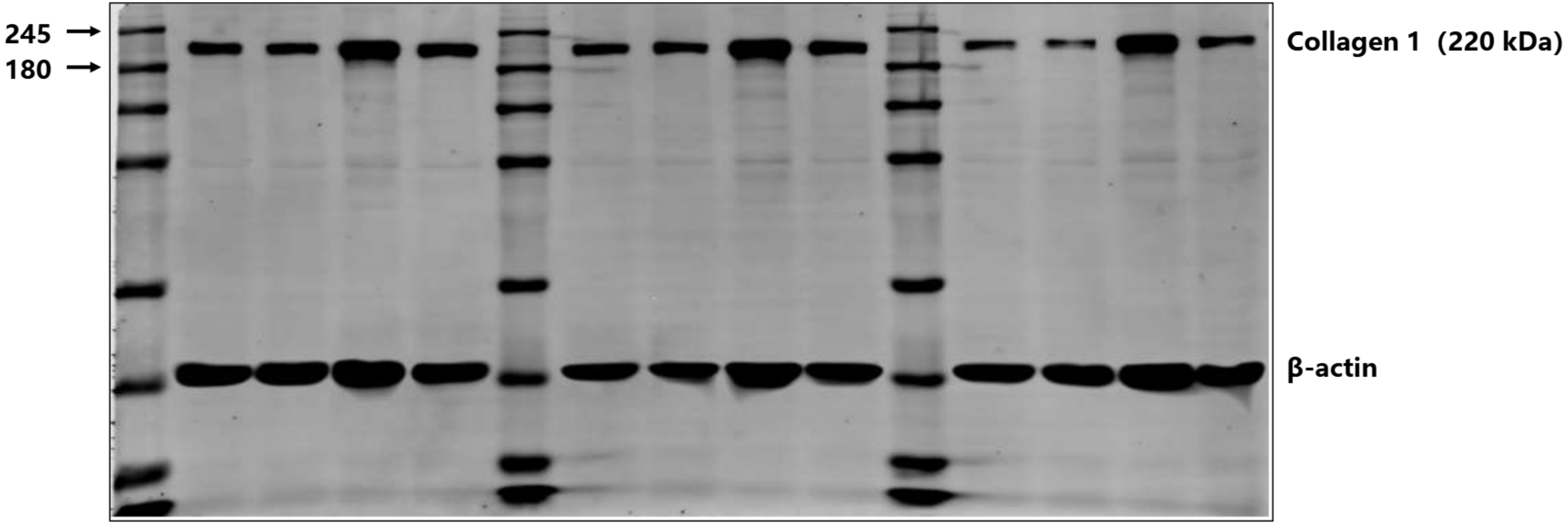

Lanes 2-5 of the unedited blot correspond to those shown in the cropped images within the manuscript.

Full unedited blot for Figure 4H

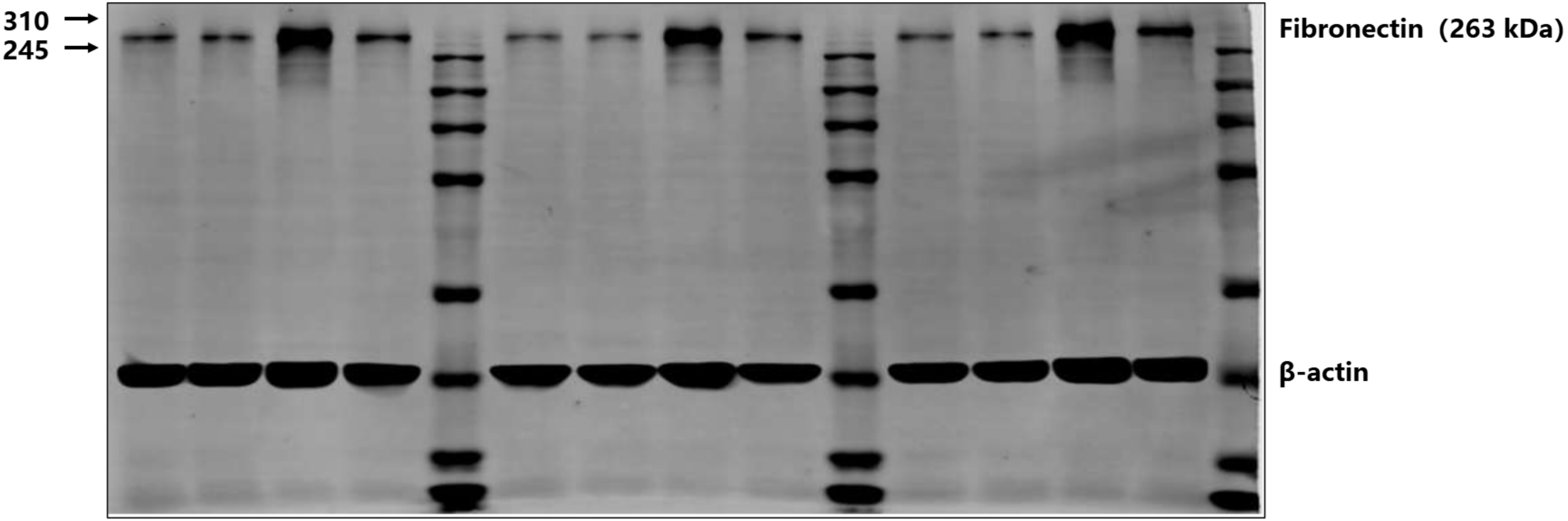

Lanes 11-14 of the unedited blot correspond to those shown in the cropped images within the manuscript.

Full unedited blot for Figure 4I

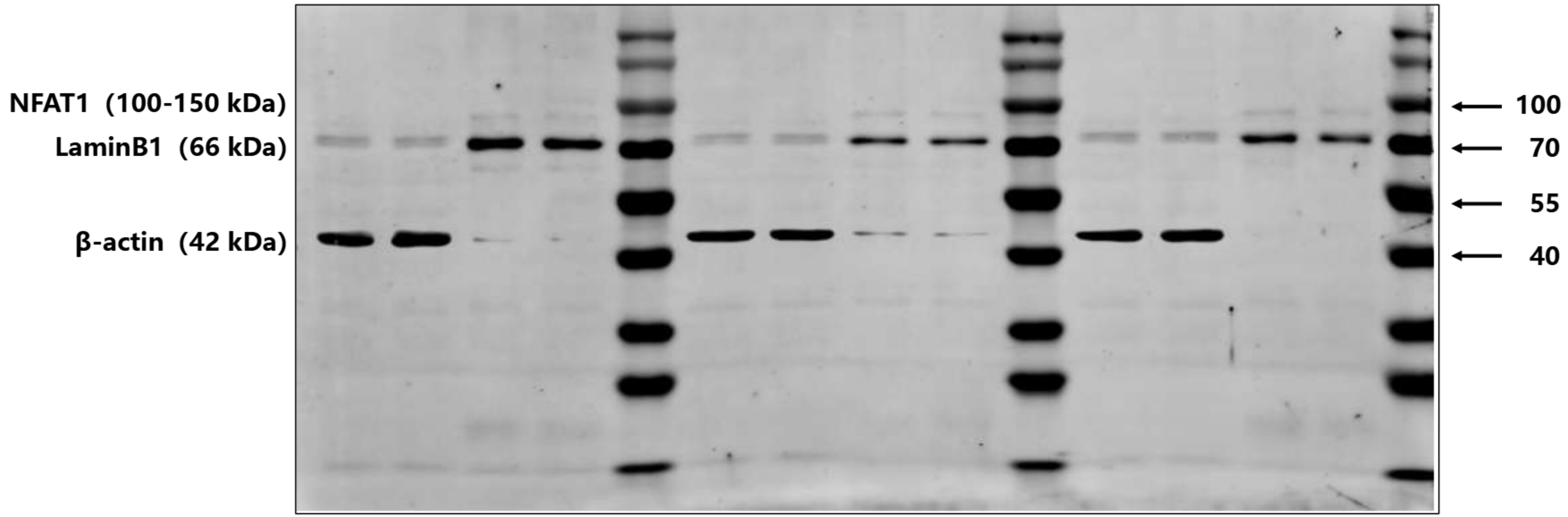

Lanes 11-14 of the unedited blot correspond to those shown in the cropped images within the manuscript.

Full unedited blot for Figure 4I

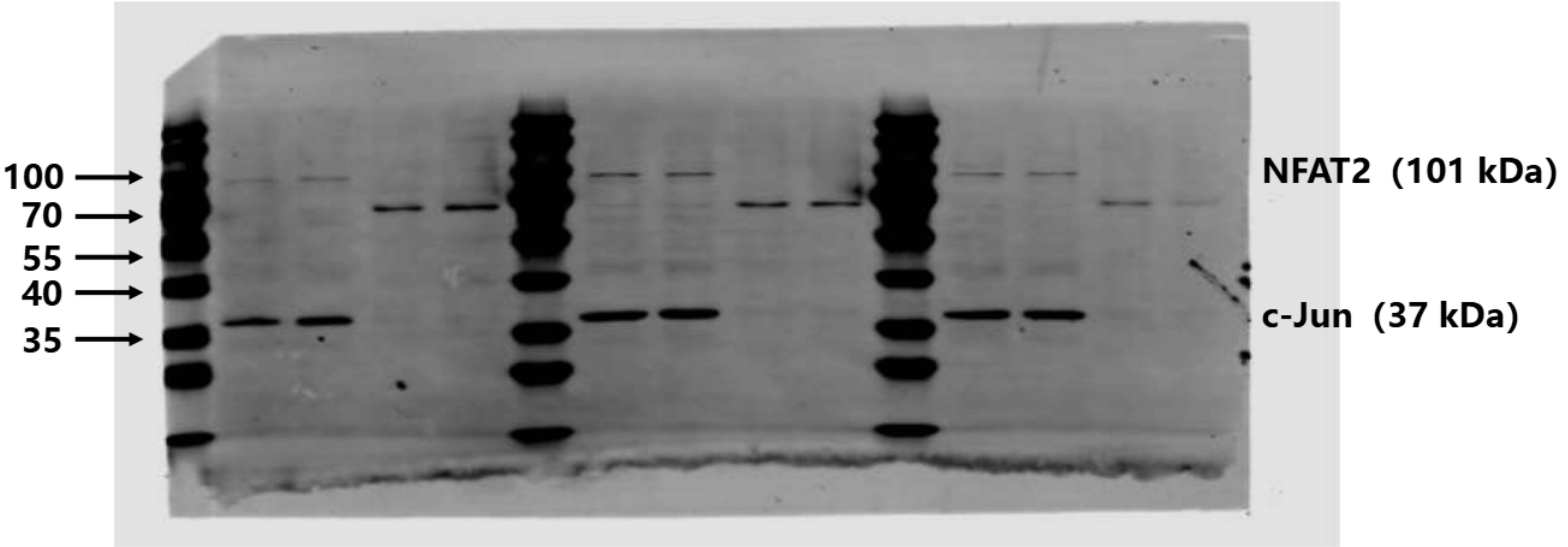

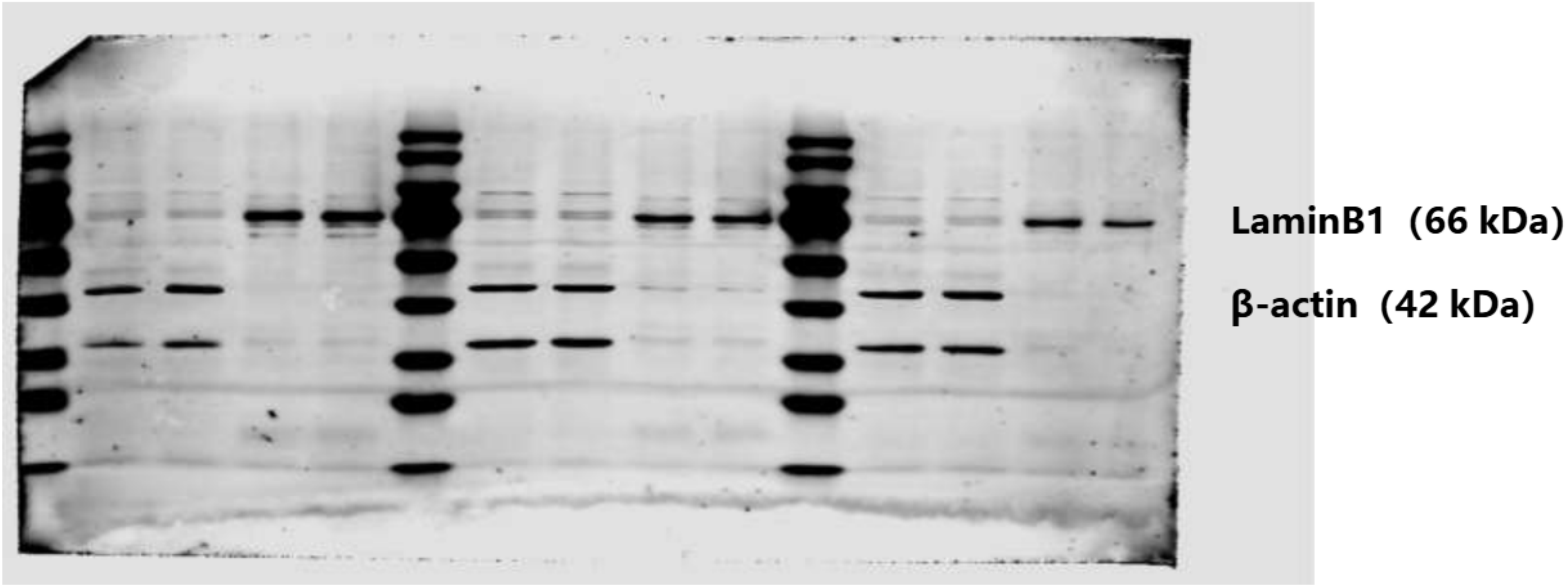

Lanes 7-10 of the unedited blot correspond to those shown in the cropped images within the manuscript.

Full unedited blot for Figure 4I

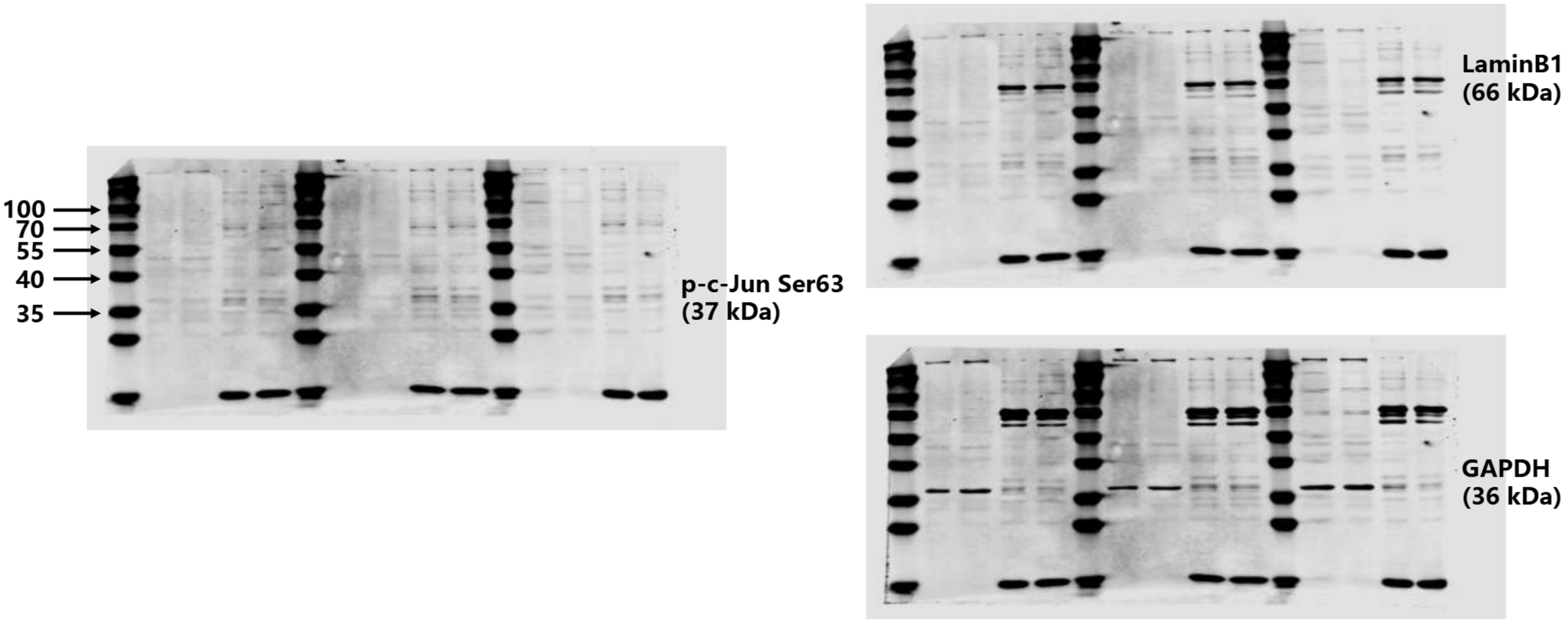

Lanes 12-15 of the unedited blot correspond to those shown in the cropped images within the manuscript.

Full unedited blot for Figure 4I

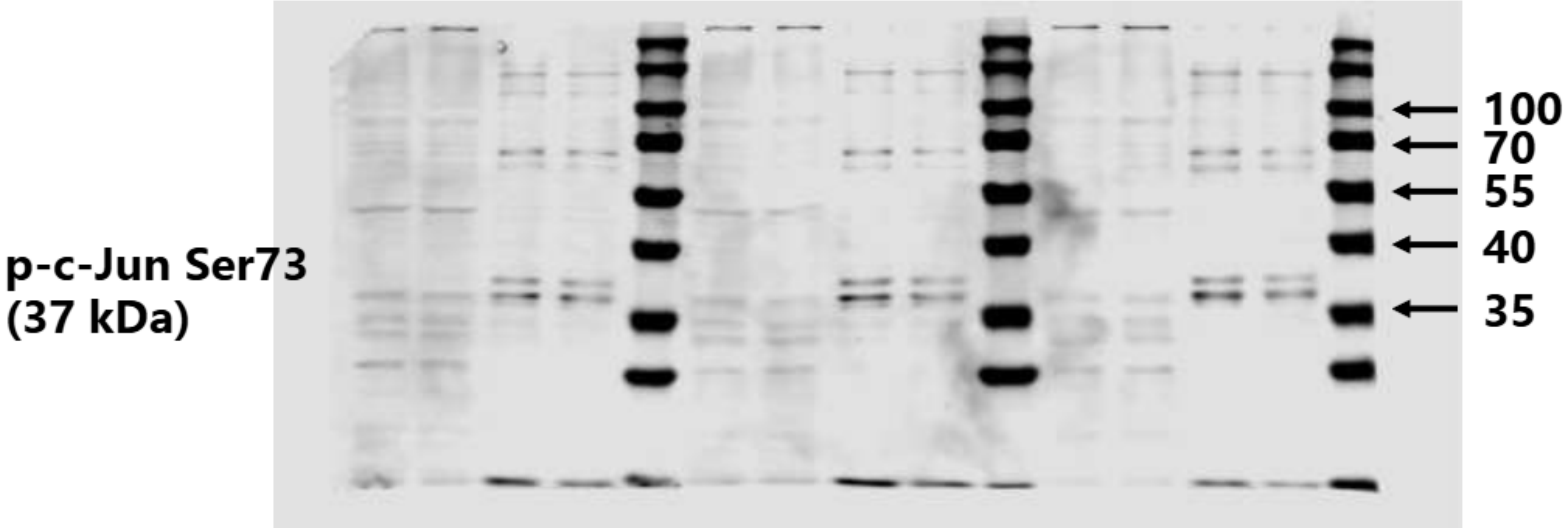

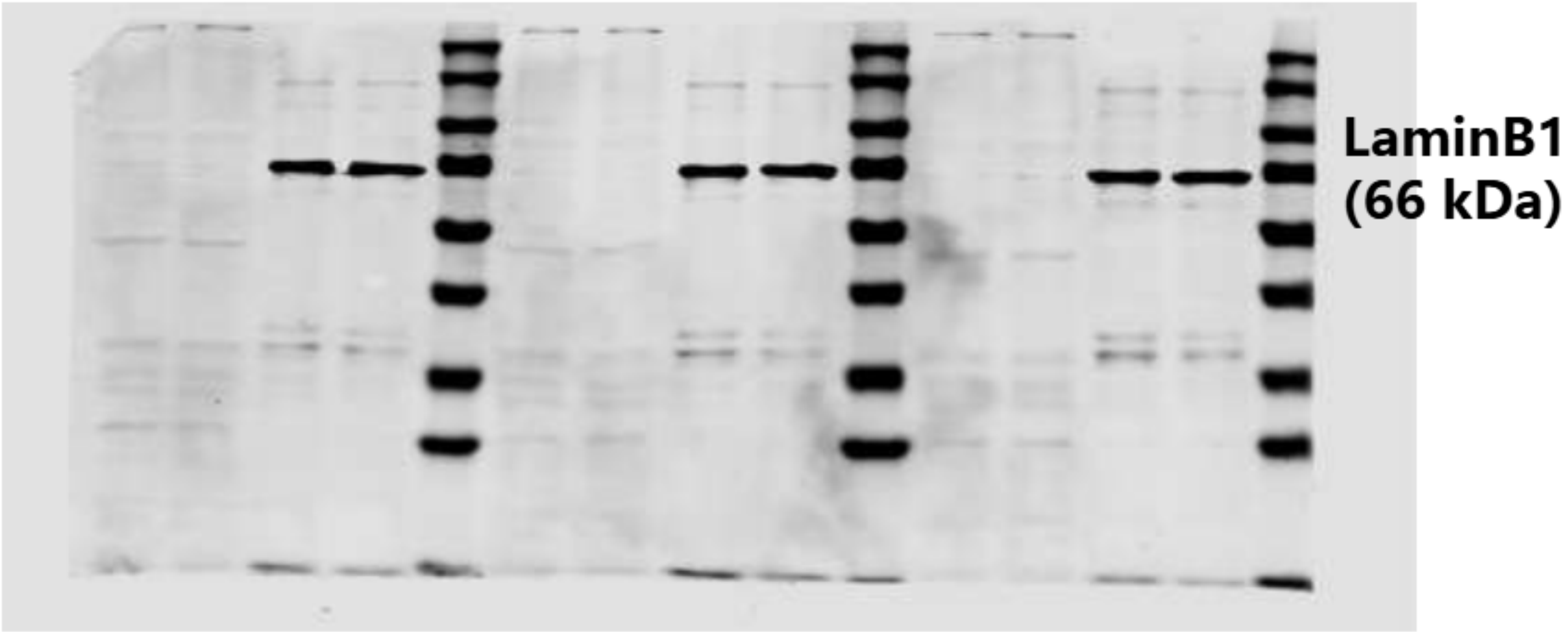

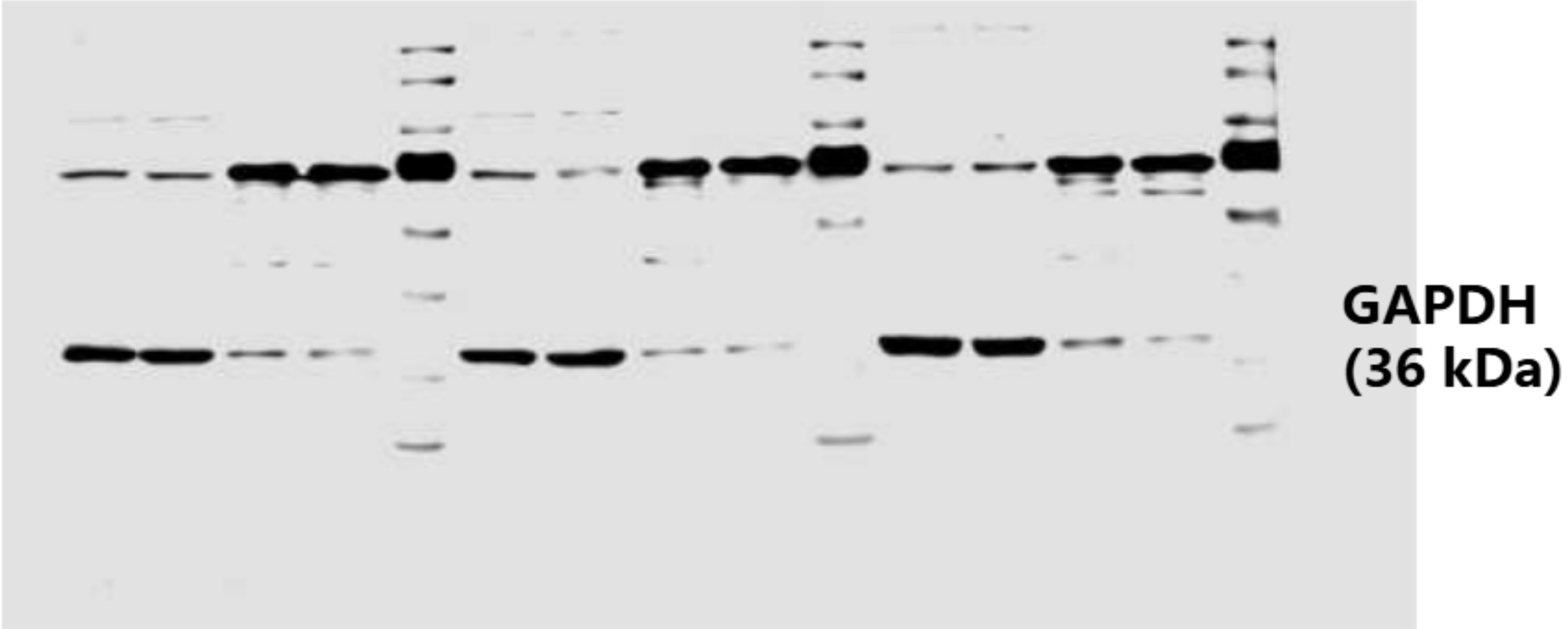

Lanes 2-5 of the unedited blot correspond to those shown in the cropped images within the manuscript.

Full unedited blot for Figure 5F

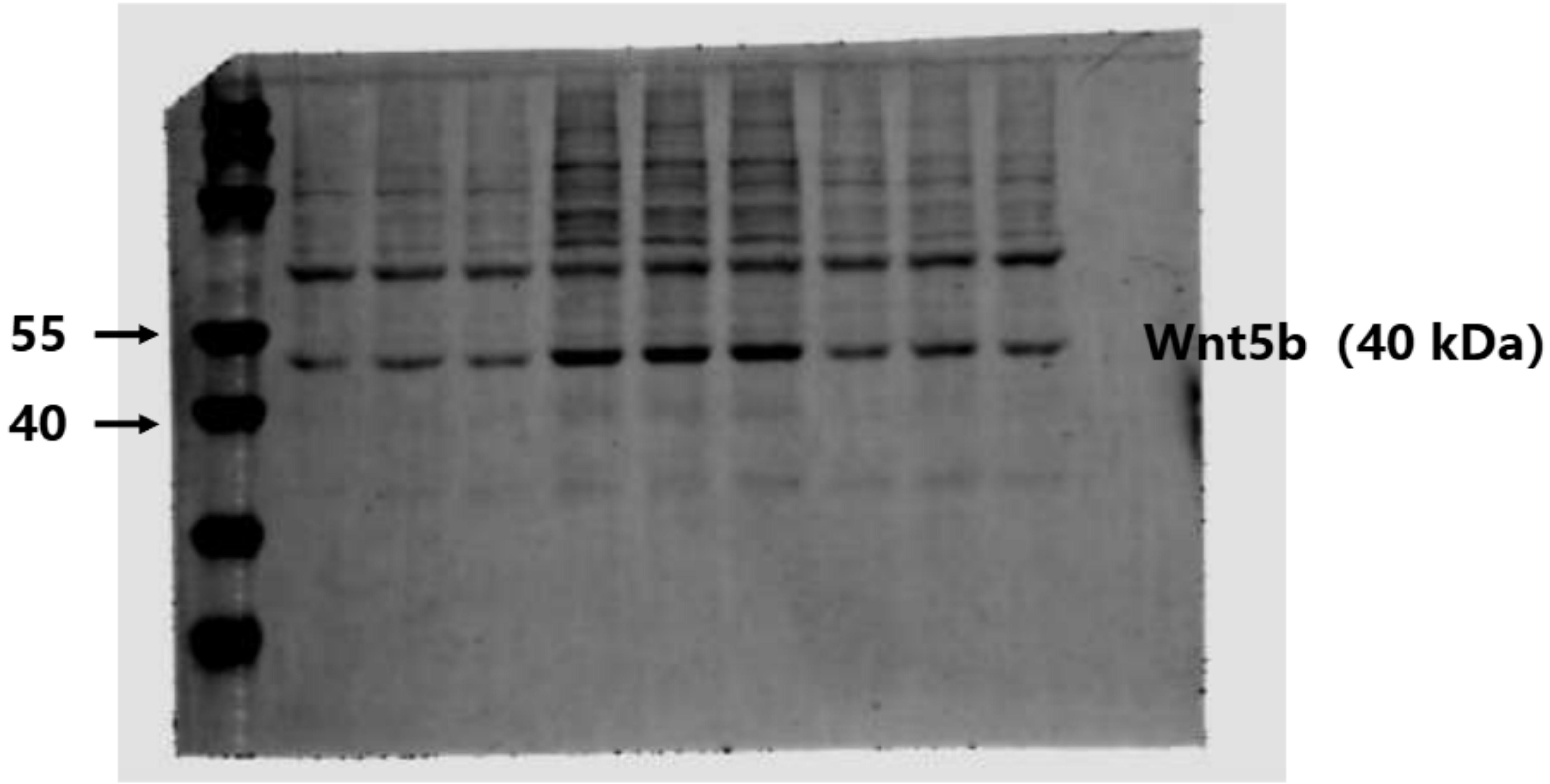

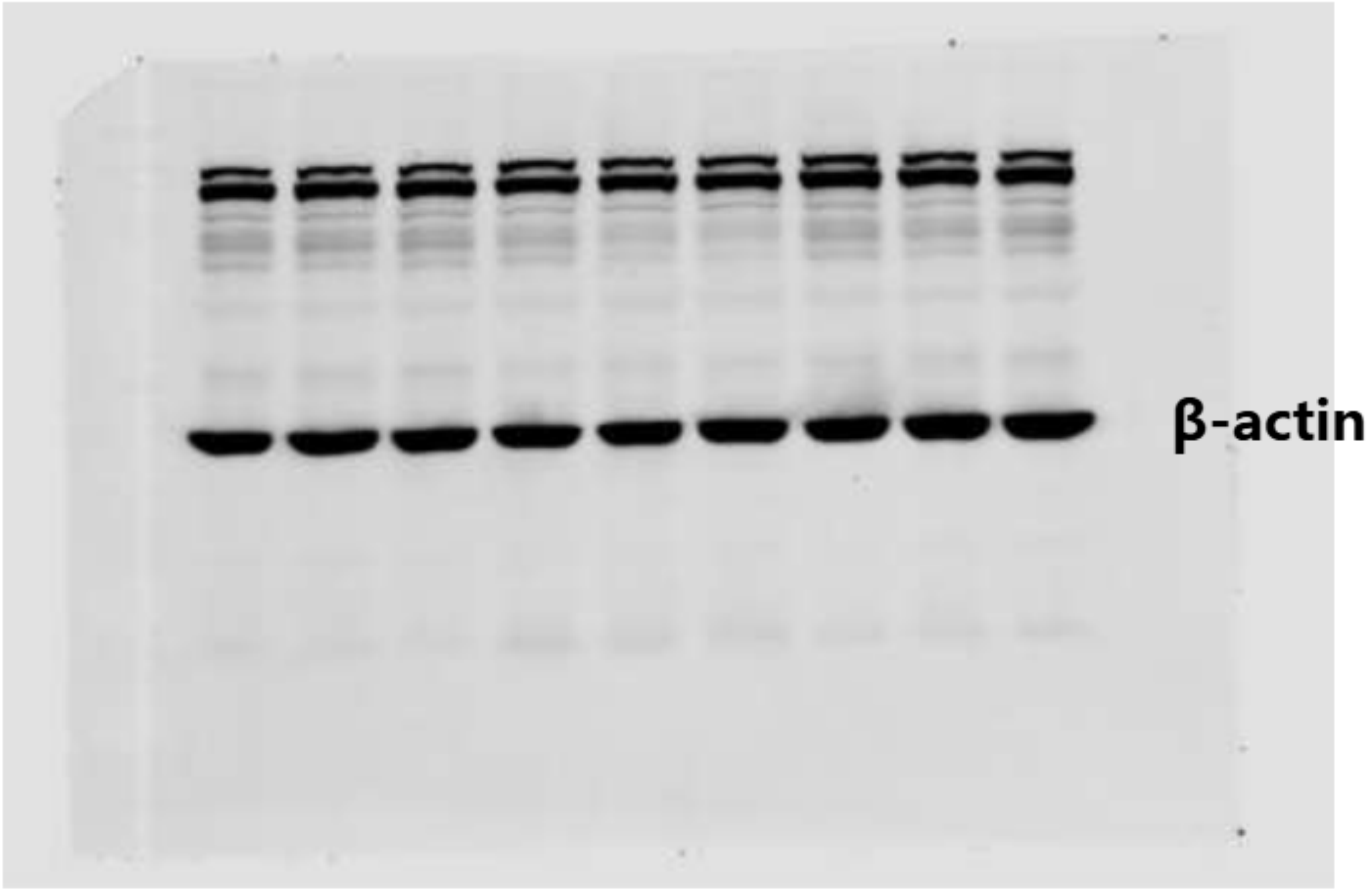

Lanes 2-10 of the unedited blot correspond to those shown in the cropped images within the manuscript.

Full unedited blot for Figure 5F

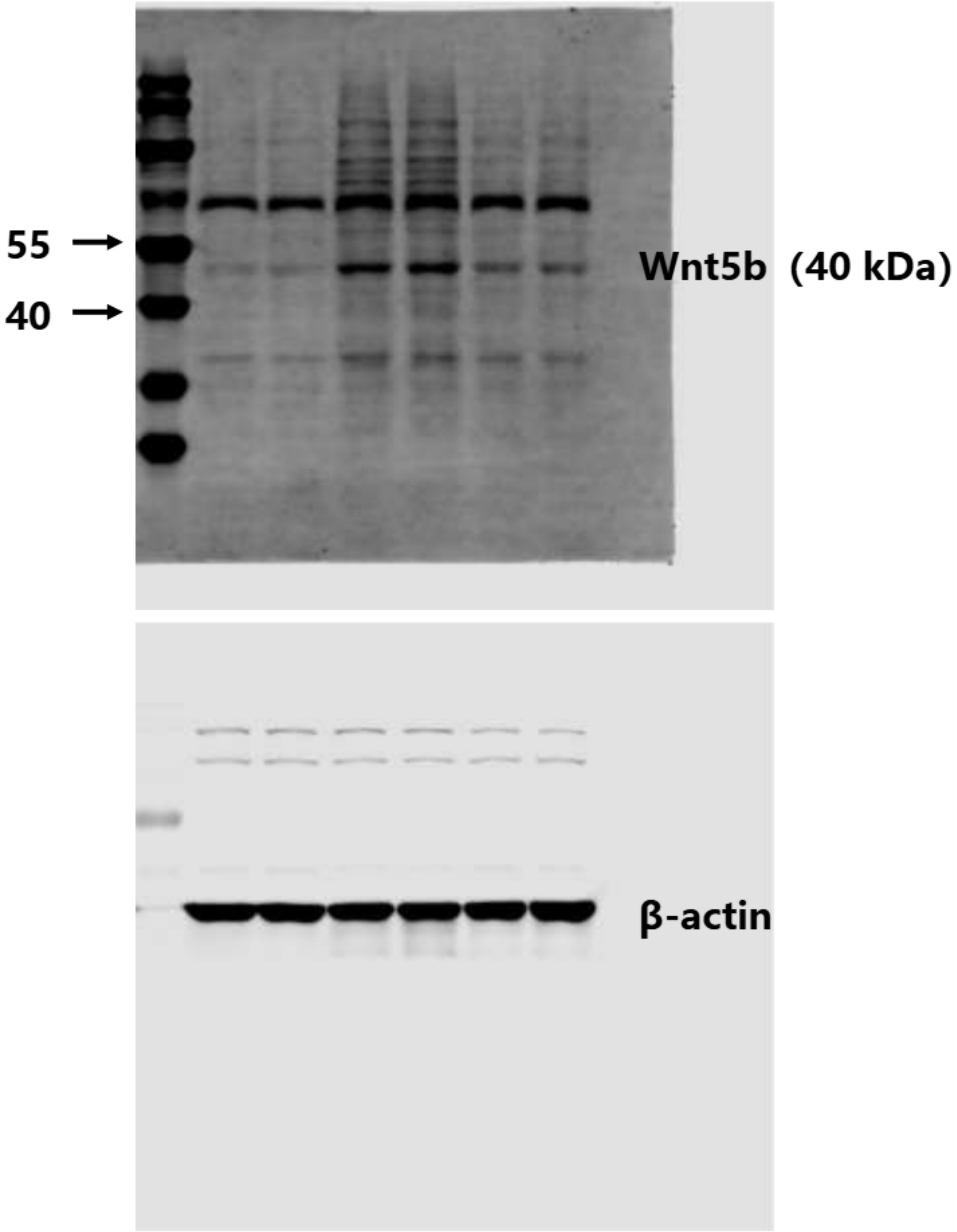

Full unedited blot for Figure 5F

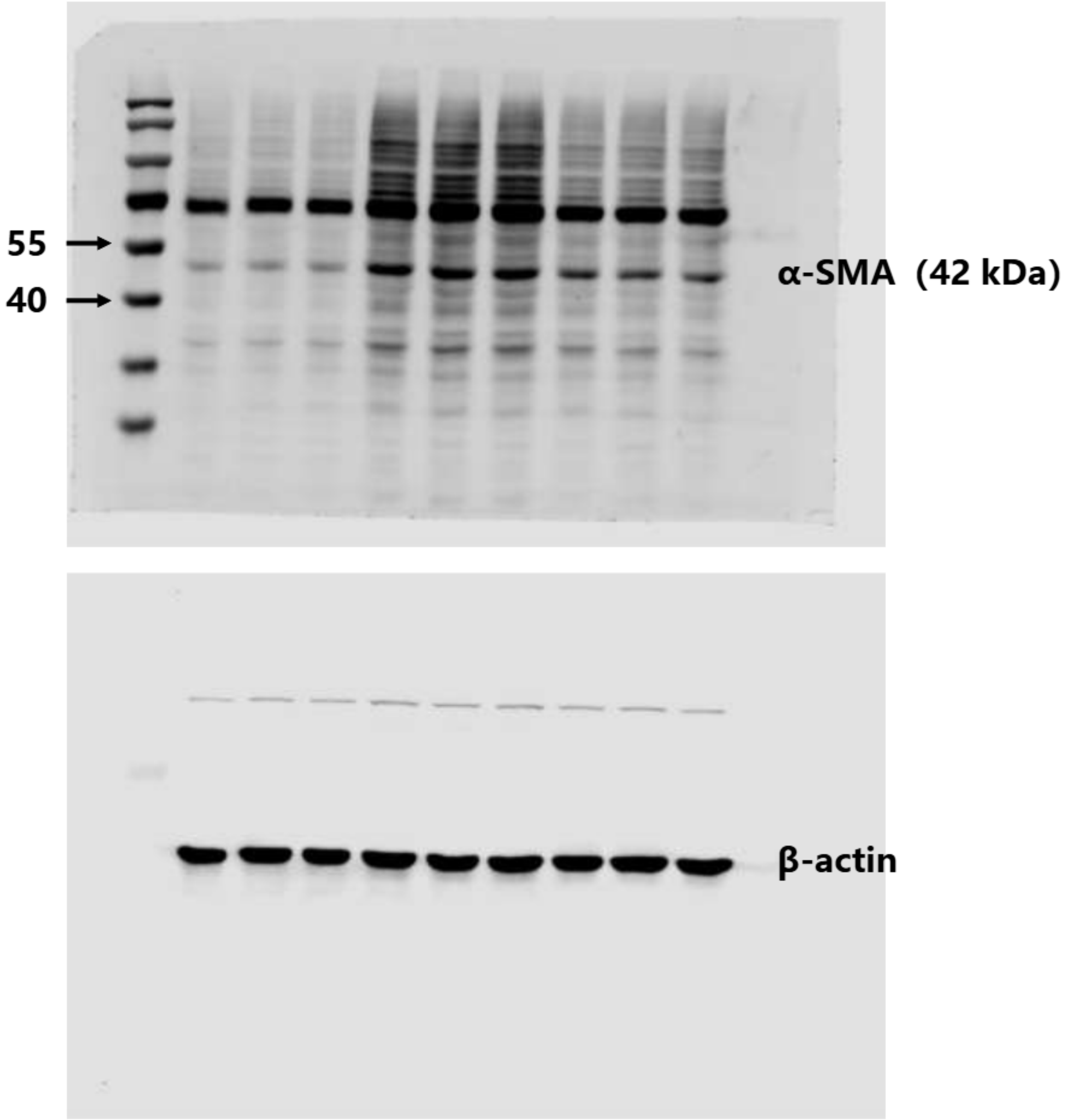

Lanes 2-10 of the unedited blot correspond to those shown in the cropped images within the manuscript.

Full unedited blot for Figure 5F

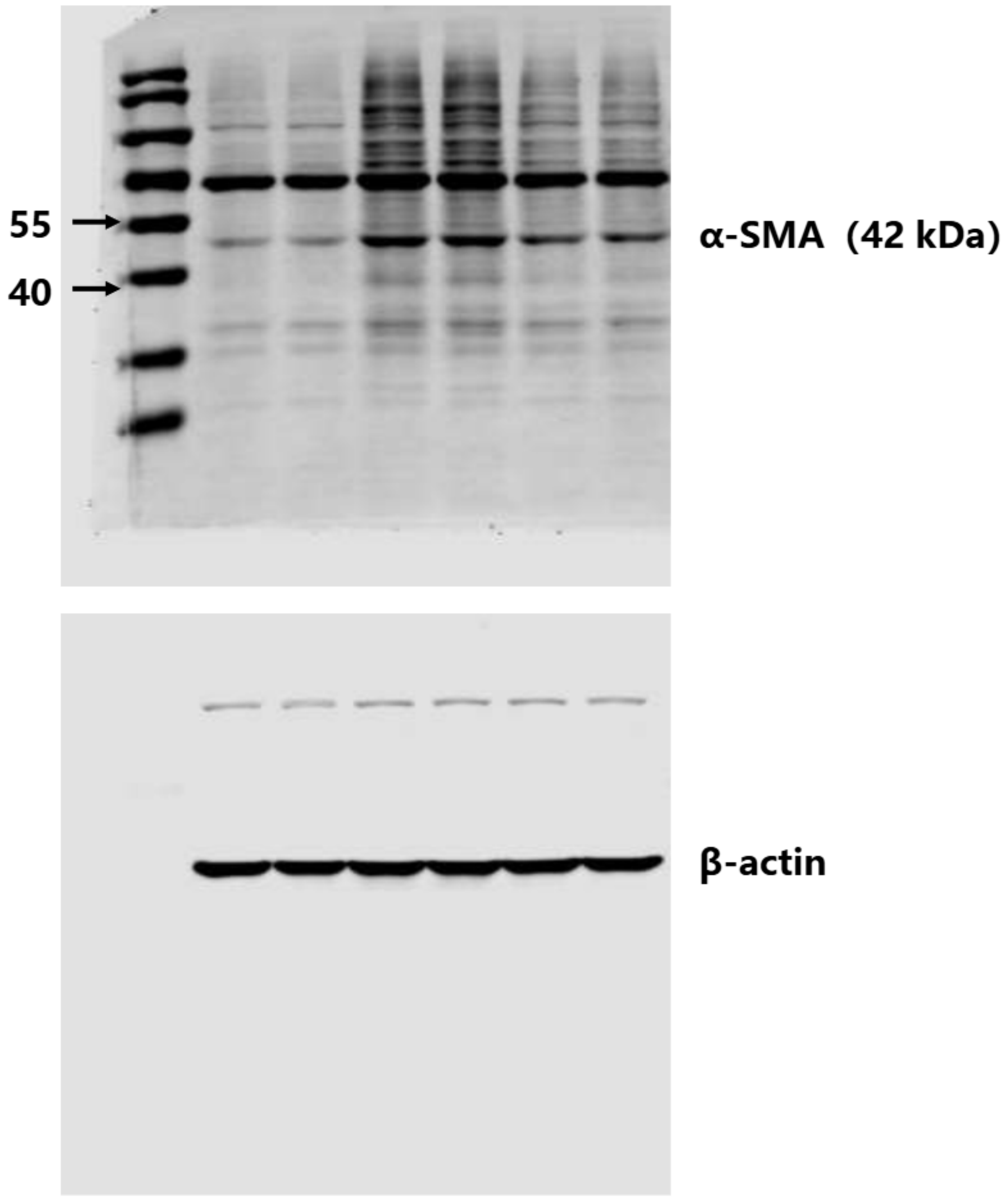

Full unedited blot for Figure 5F

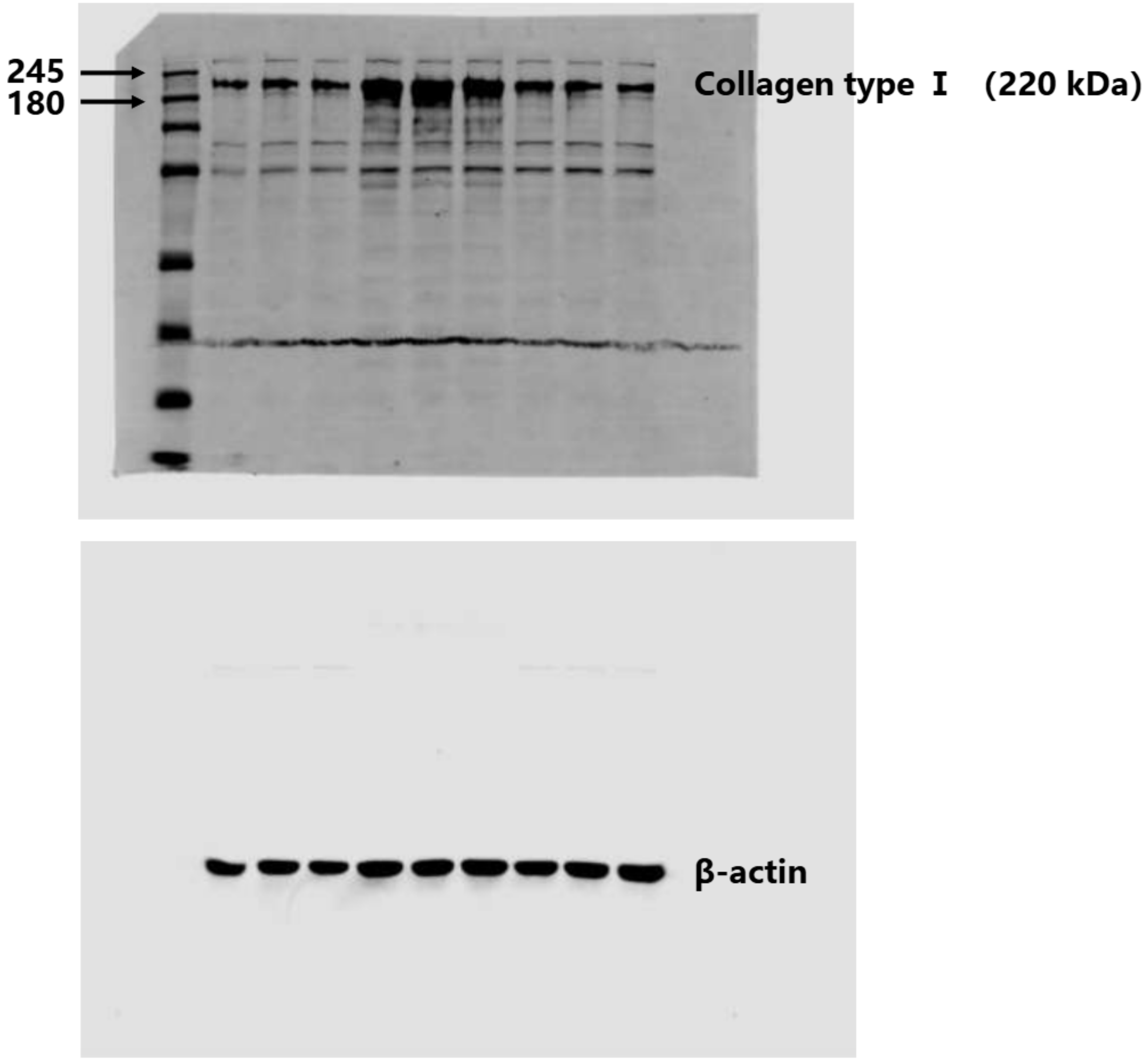

Lanes 2-10 of the unedited blot correspond to those shown in the cropped images within the manuscript.

Full unedited blot for Figure 5F

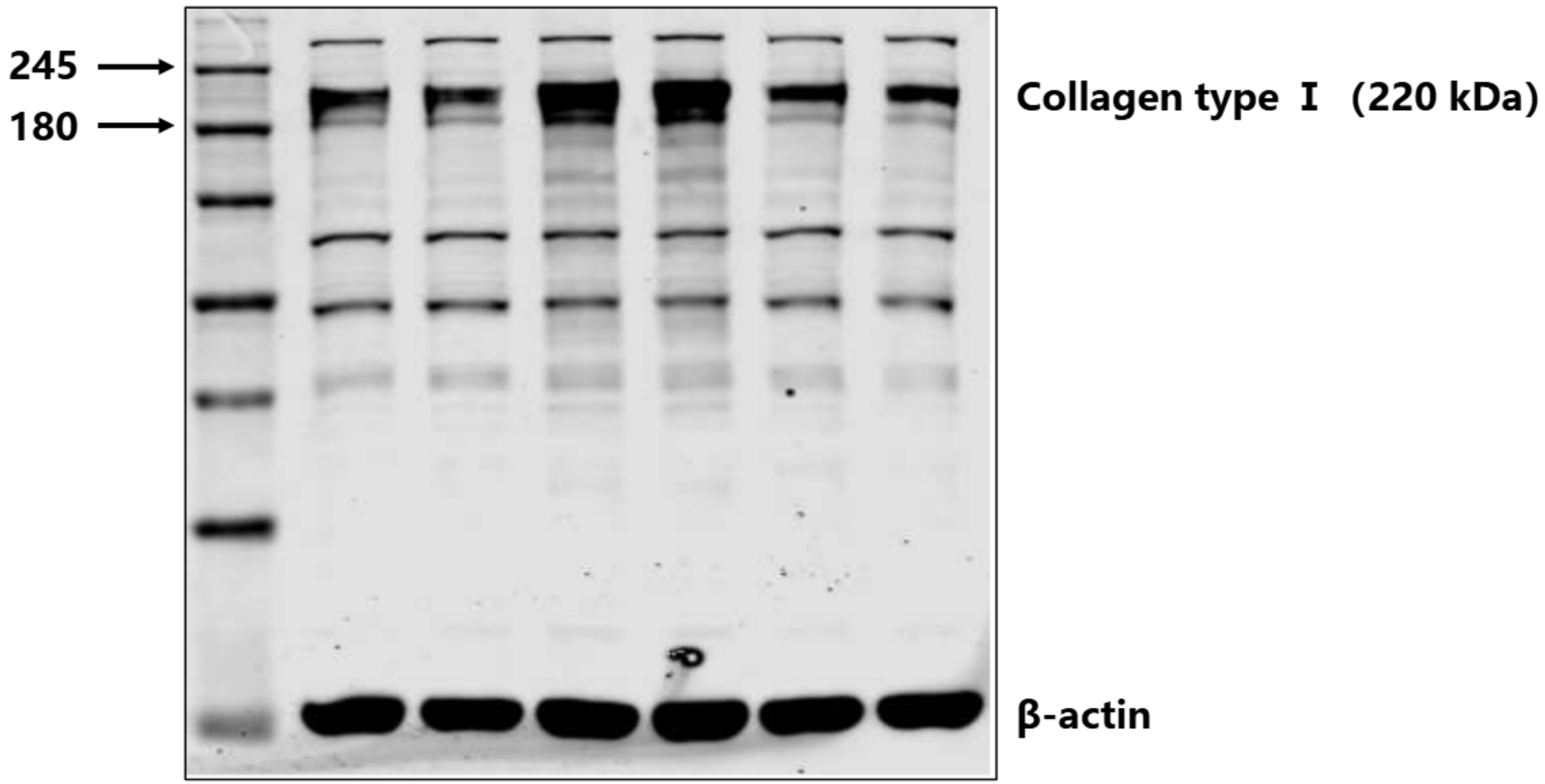

Full unedited blot for Figure 5F

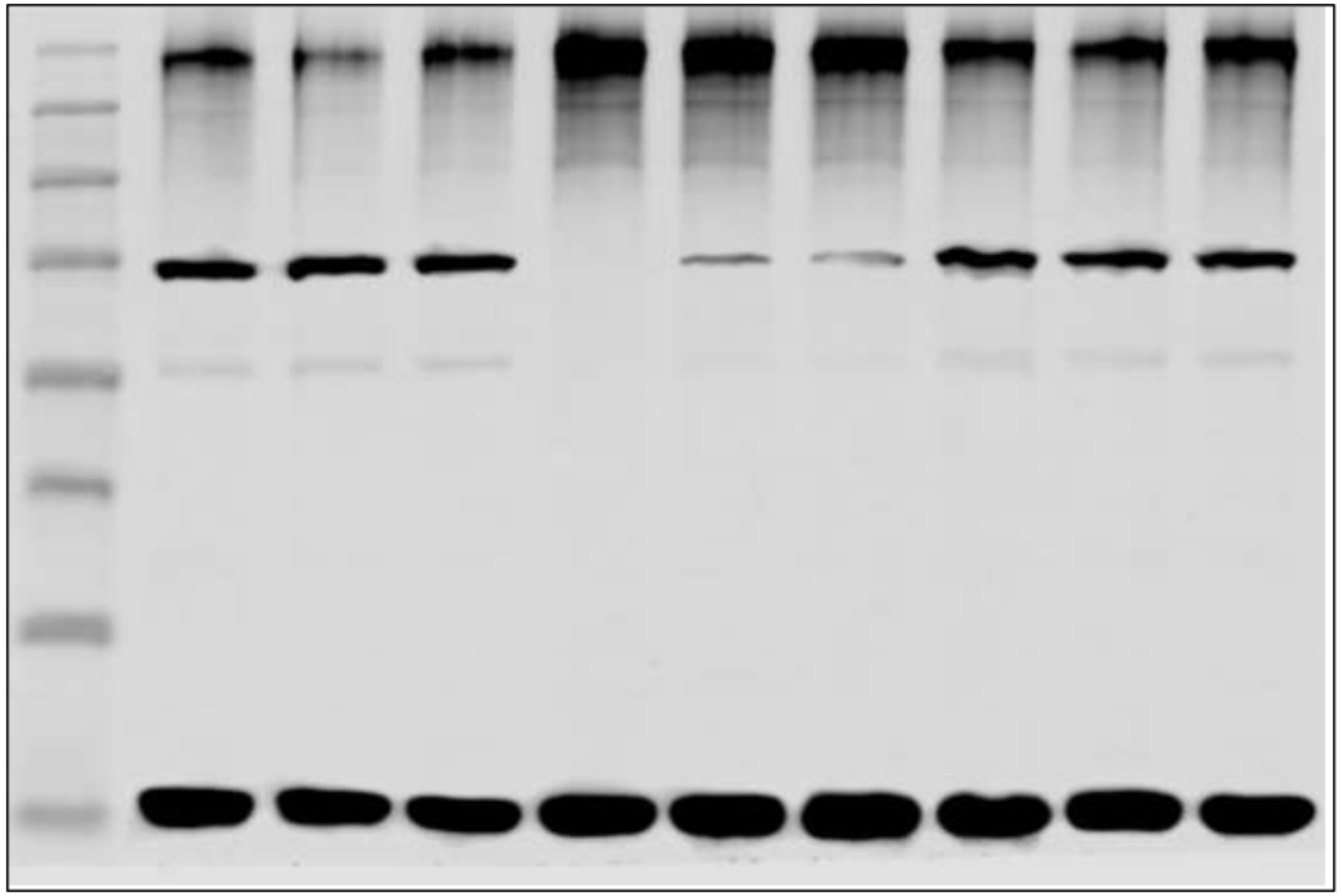

Lanes 2-10 of the unedited blot correspond to those shown in the cropped images within the manuscript.

Full unedited blot for Figure 5F

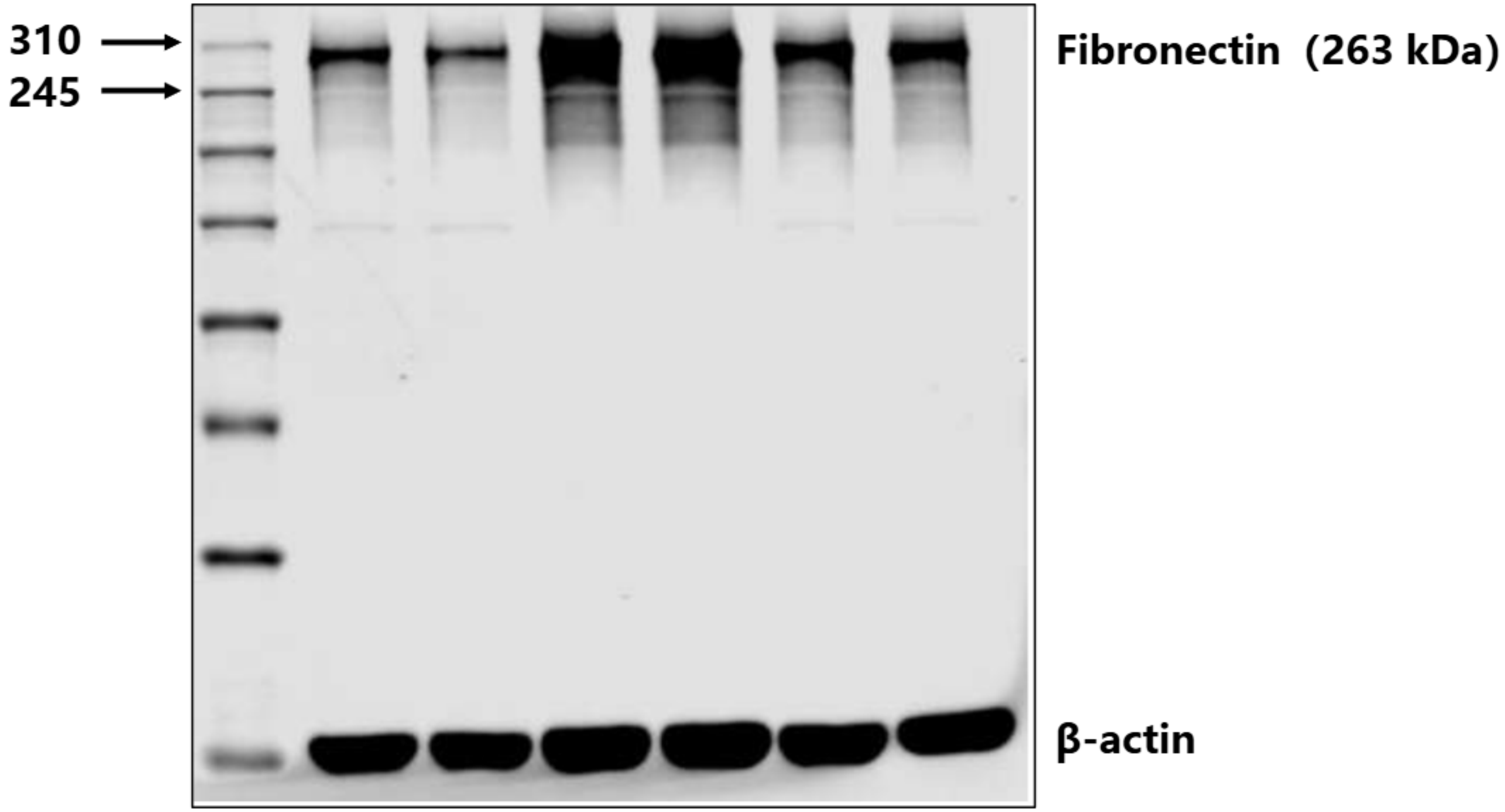

Full unedited blot for Figure 6A

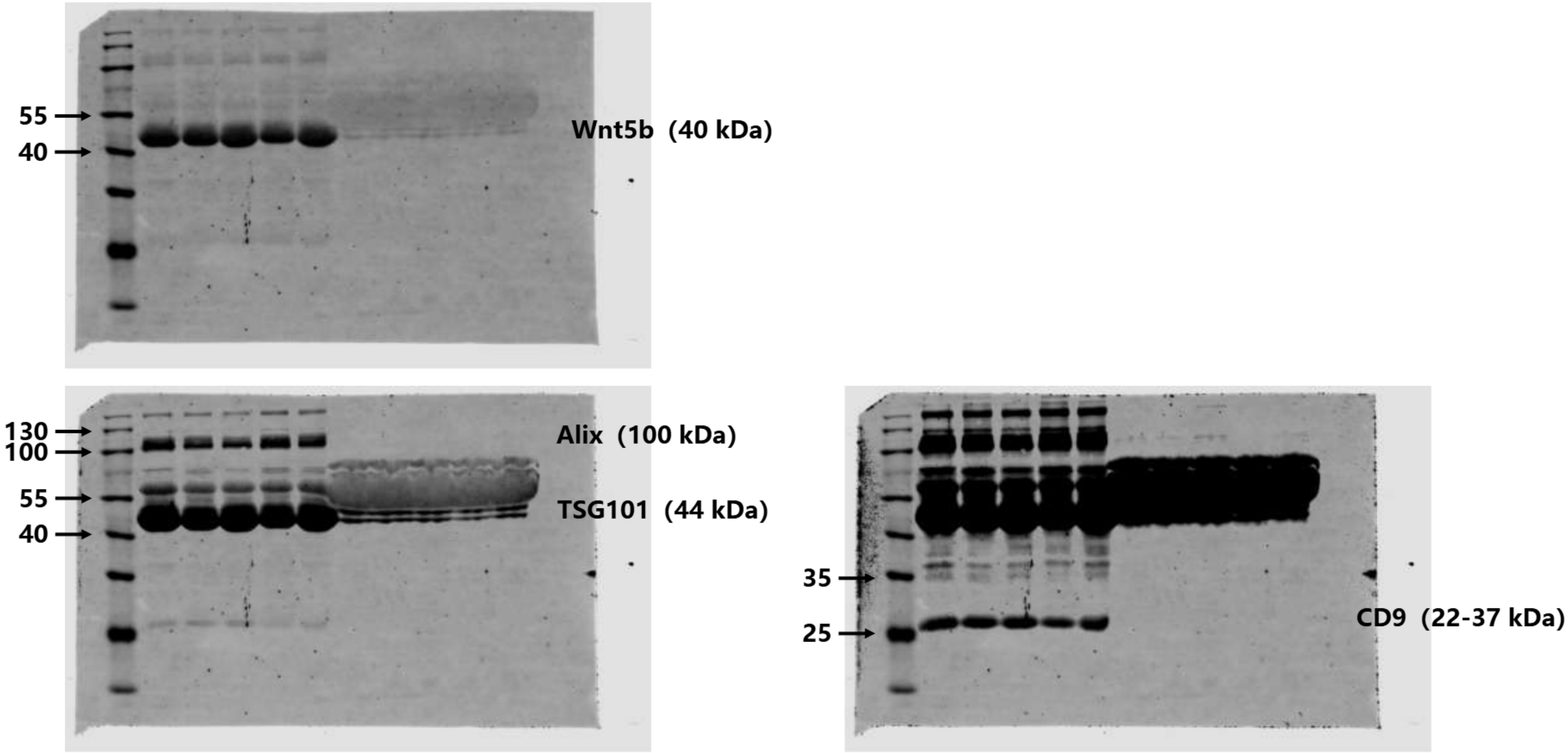

Lanes of the unedited blot correspond to those shown in the cropped images within the manuscript.

Full unedited blot for Figure 6A

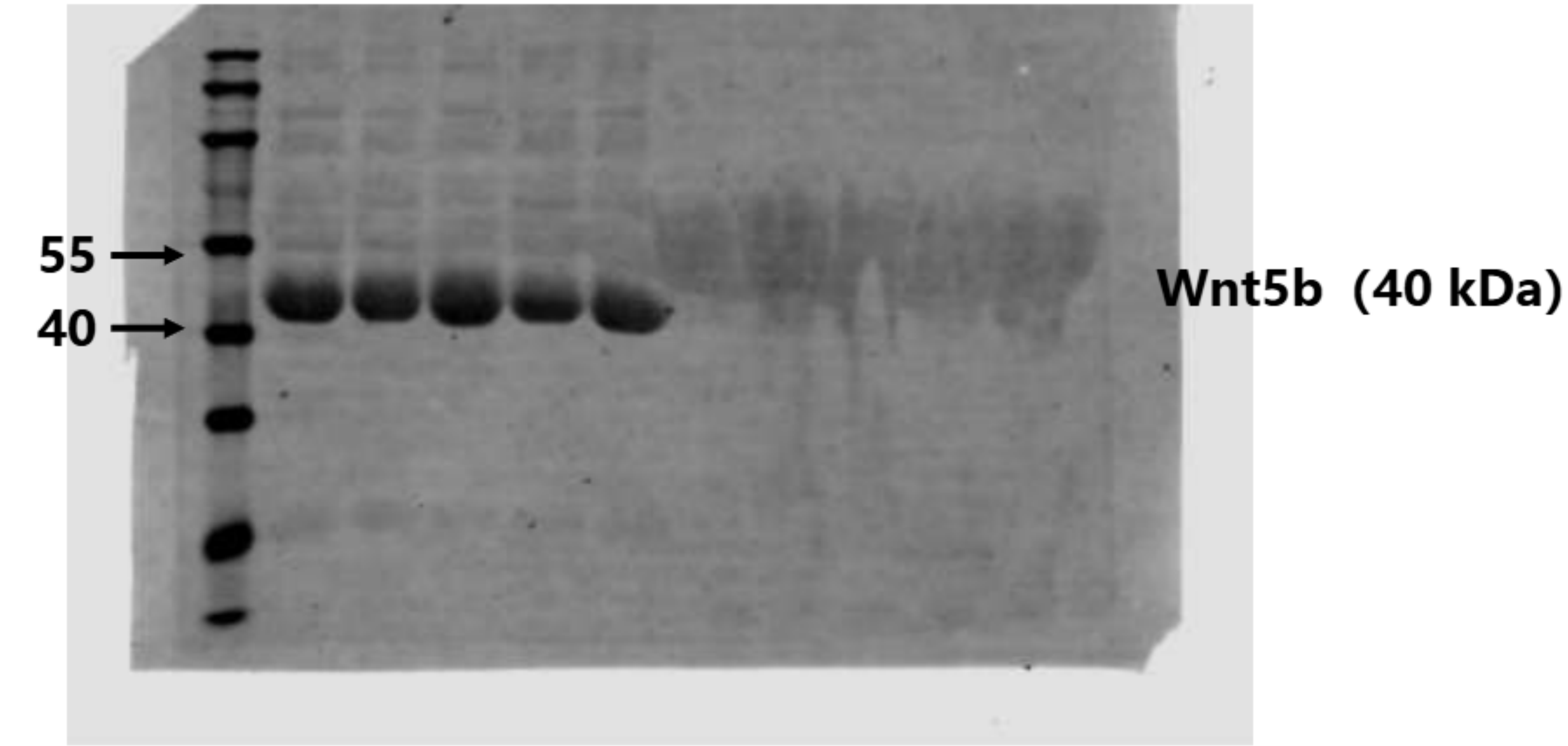

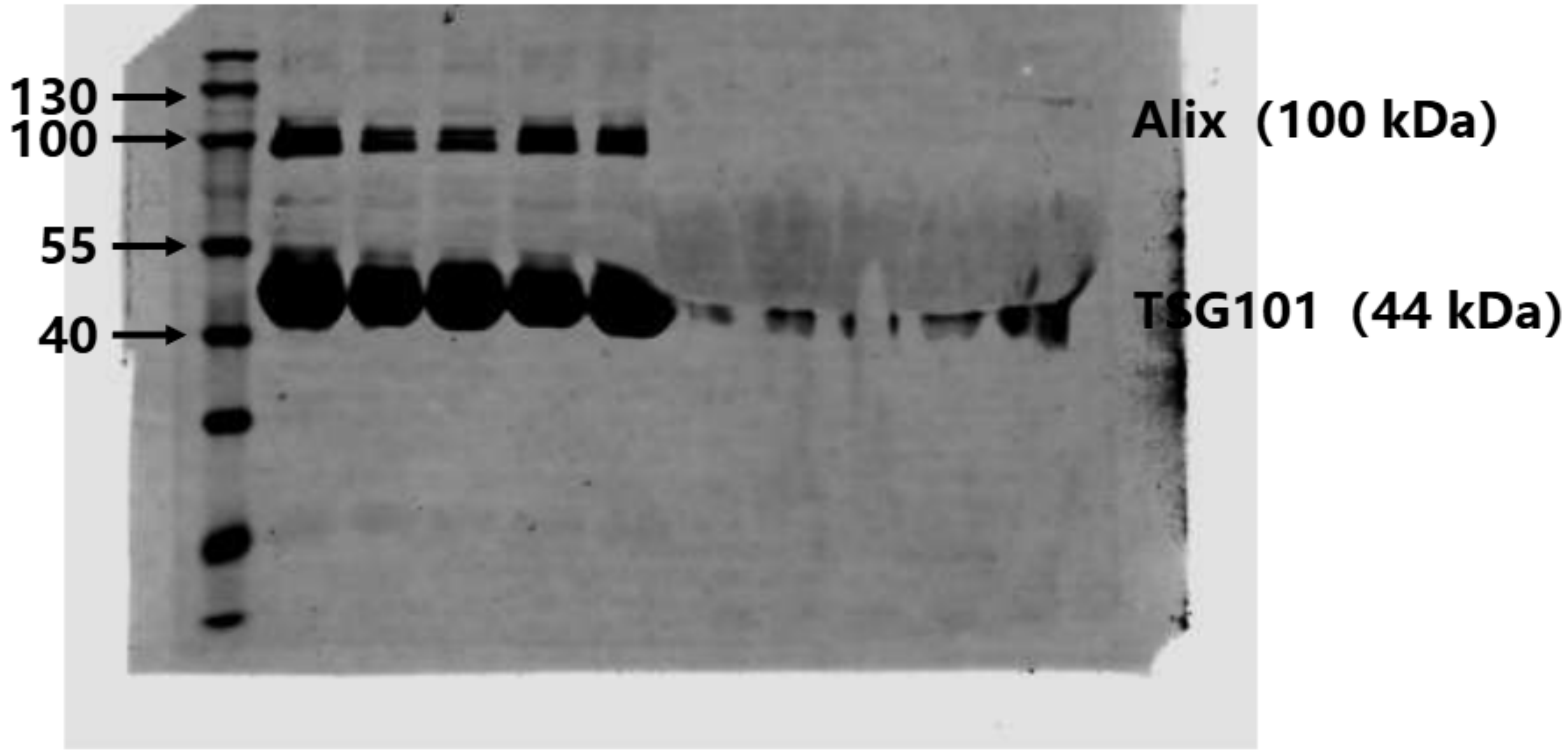

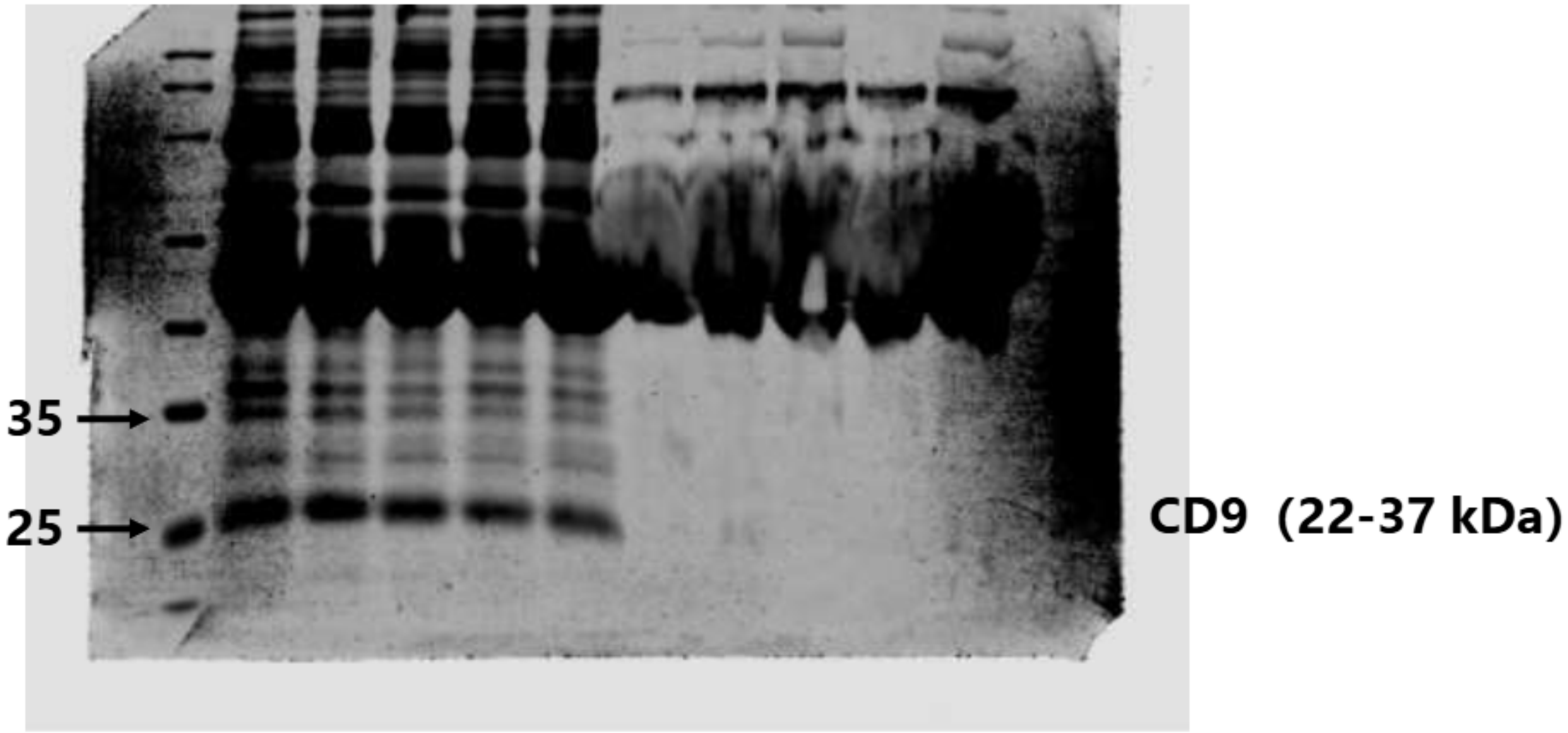

Lanes of the unedited blot correspond to those shown in the cropped images within the manuscript.

Full unedited blot for Figure 6A

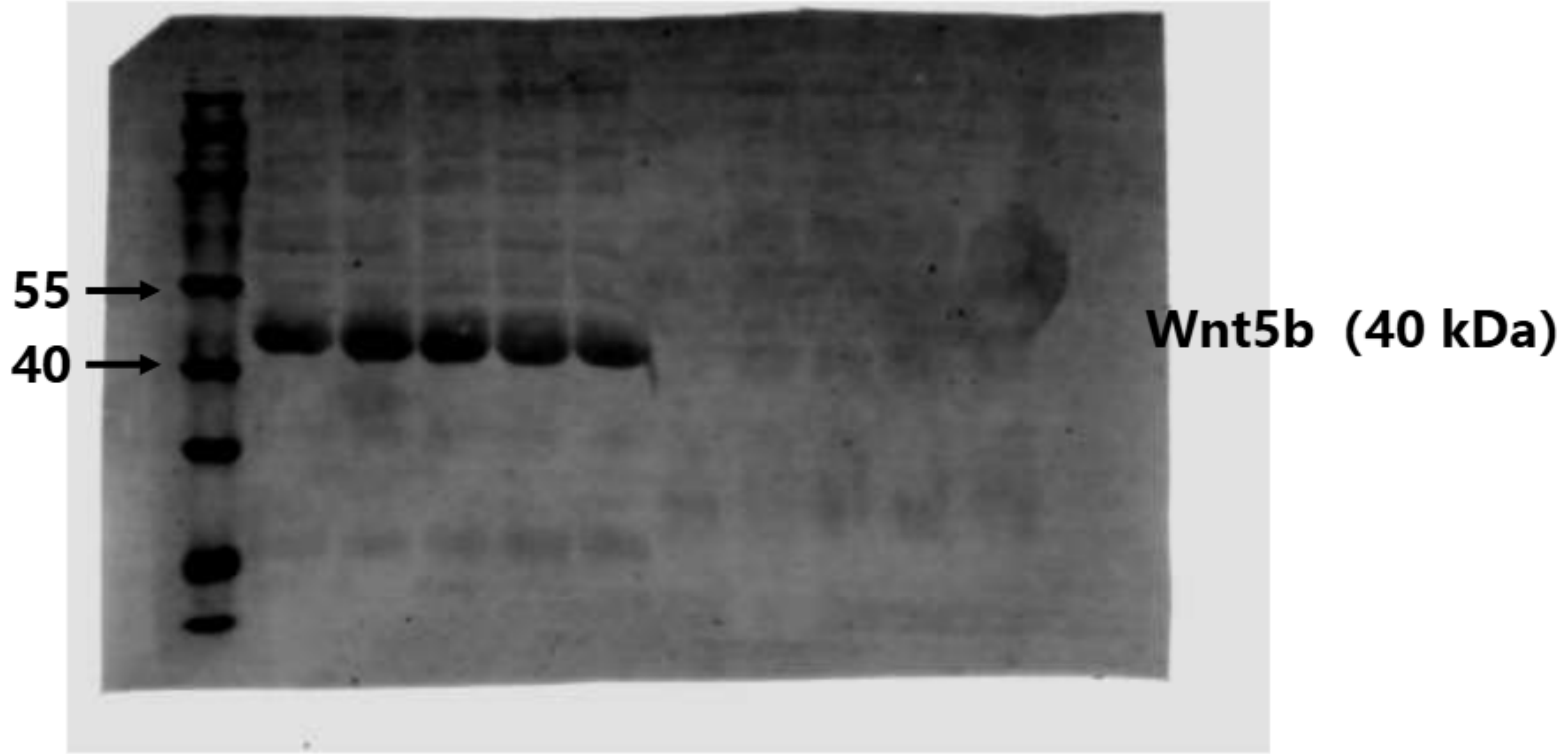

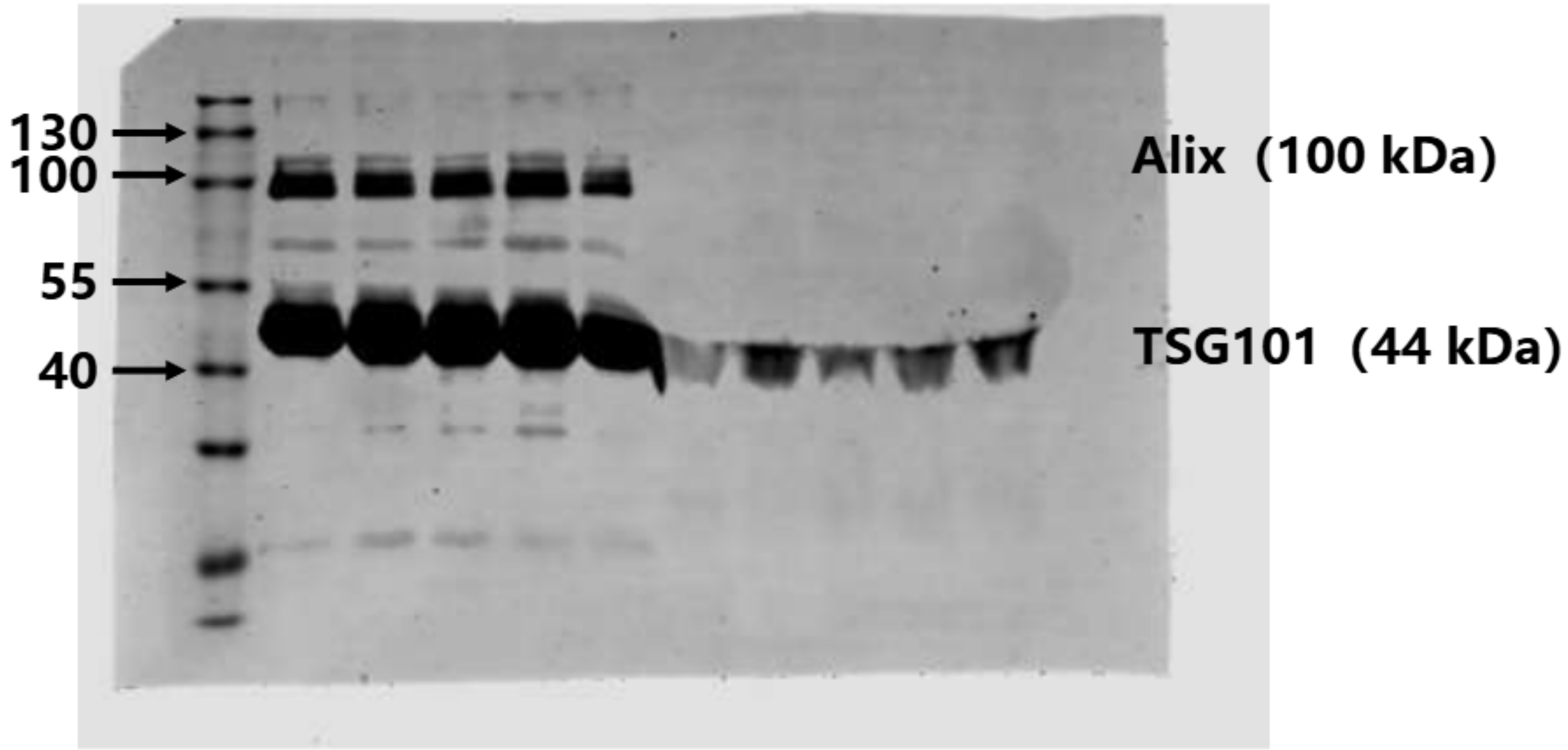

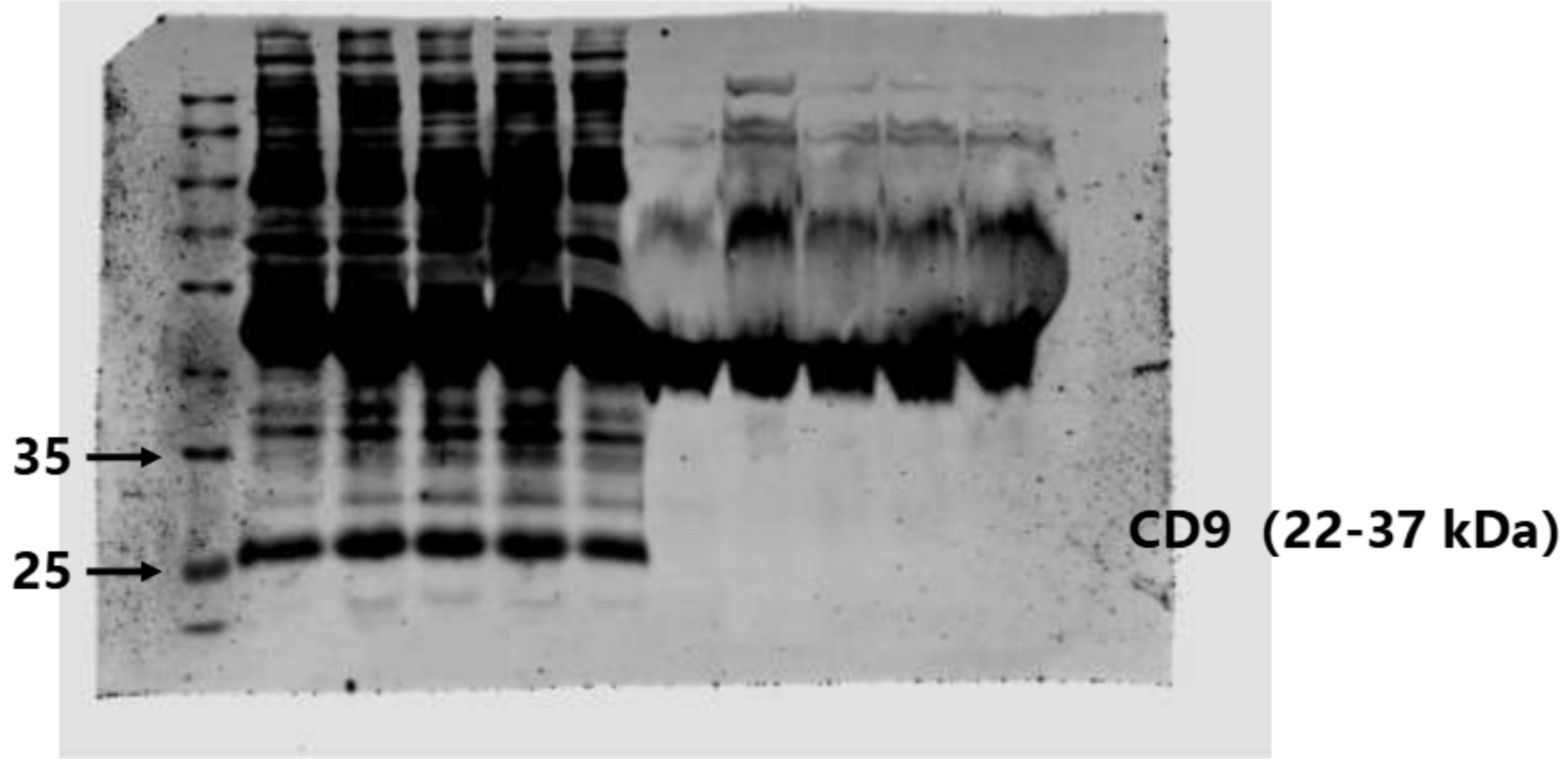

Lanes of the unedited blot correspond to those shown in the cropped images within the manuscript.

Full unedited blot for Figure 6B

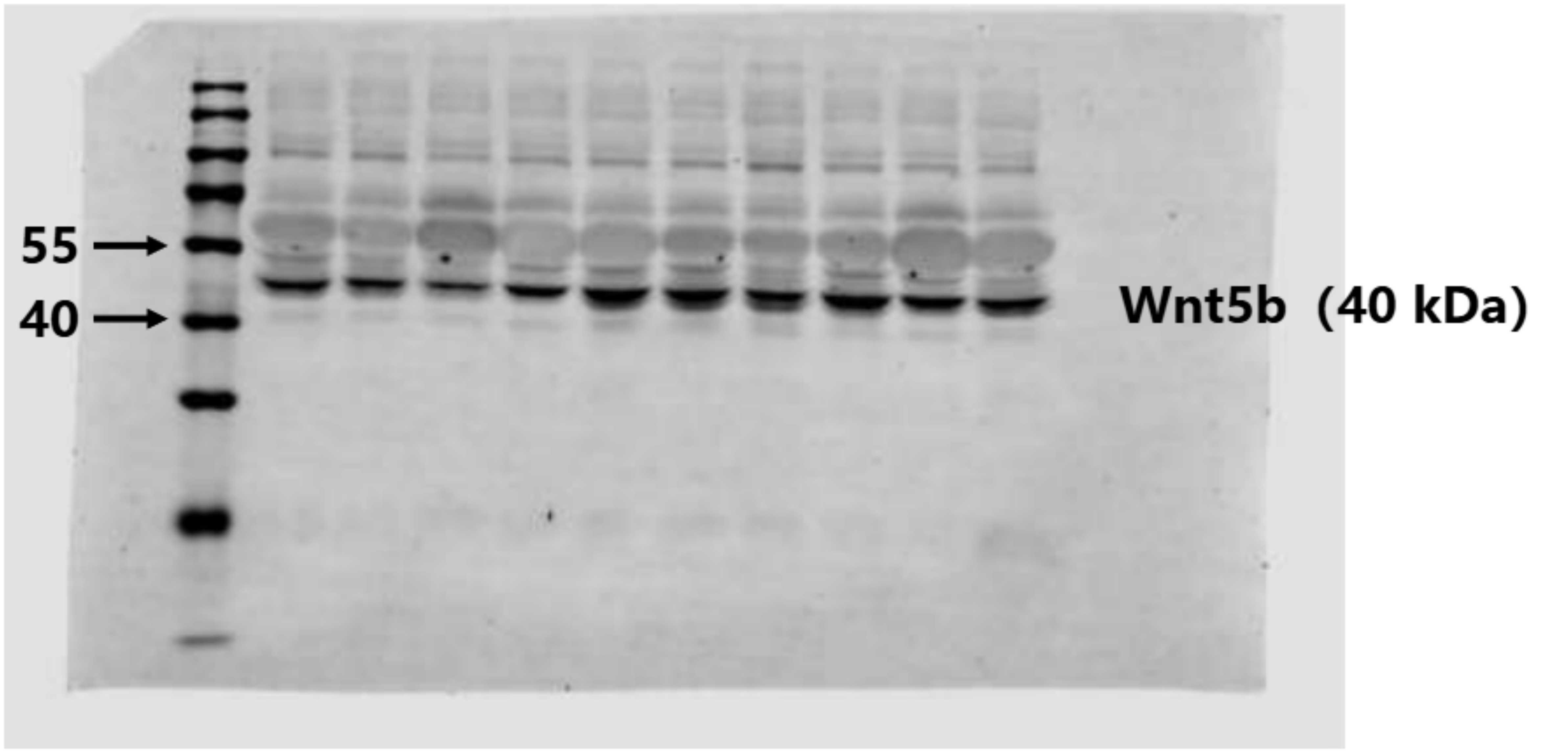

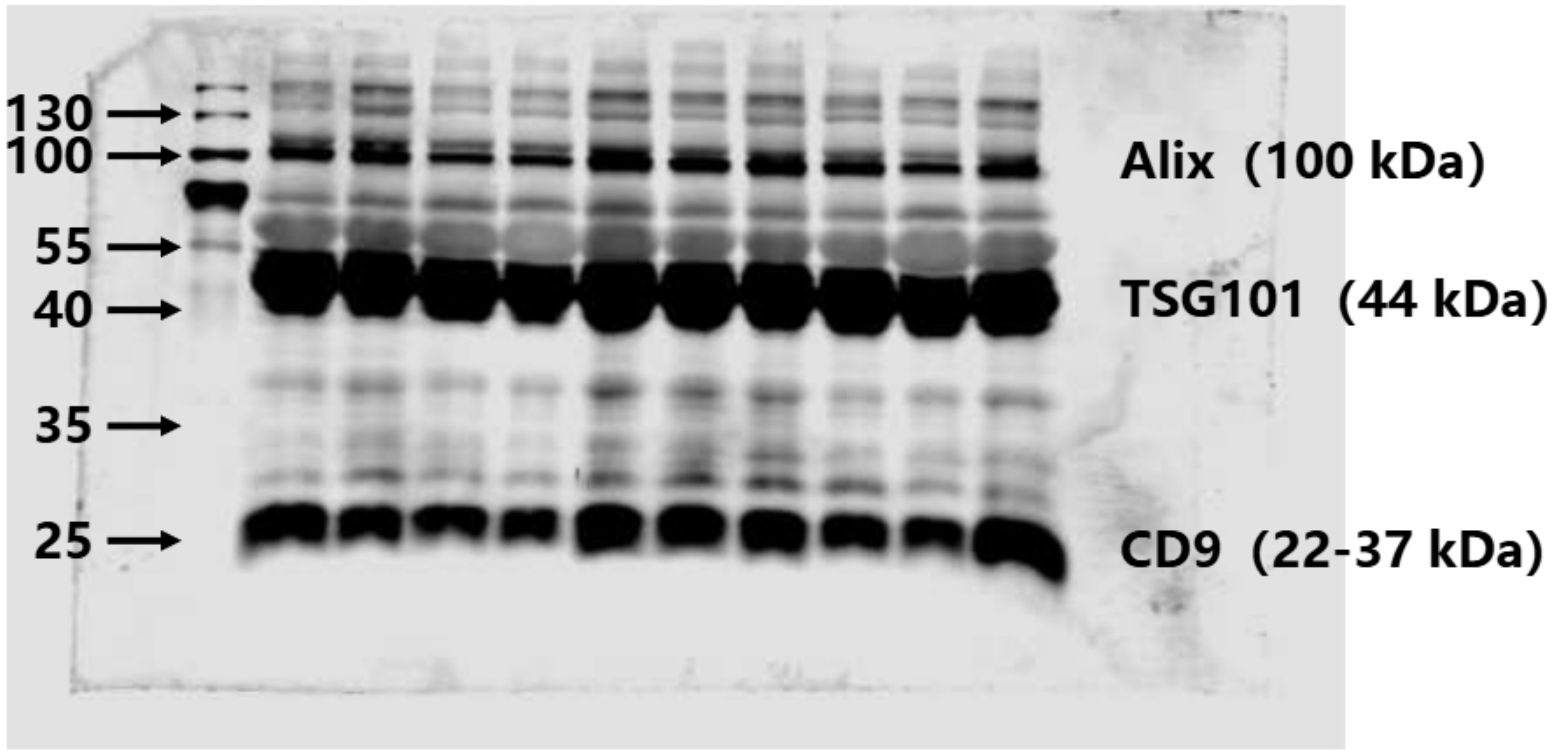

Lanes of the unedited blot correspond to those shown in the cropped images within the manuscript.

Full unedited blot for Figure 6B

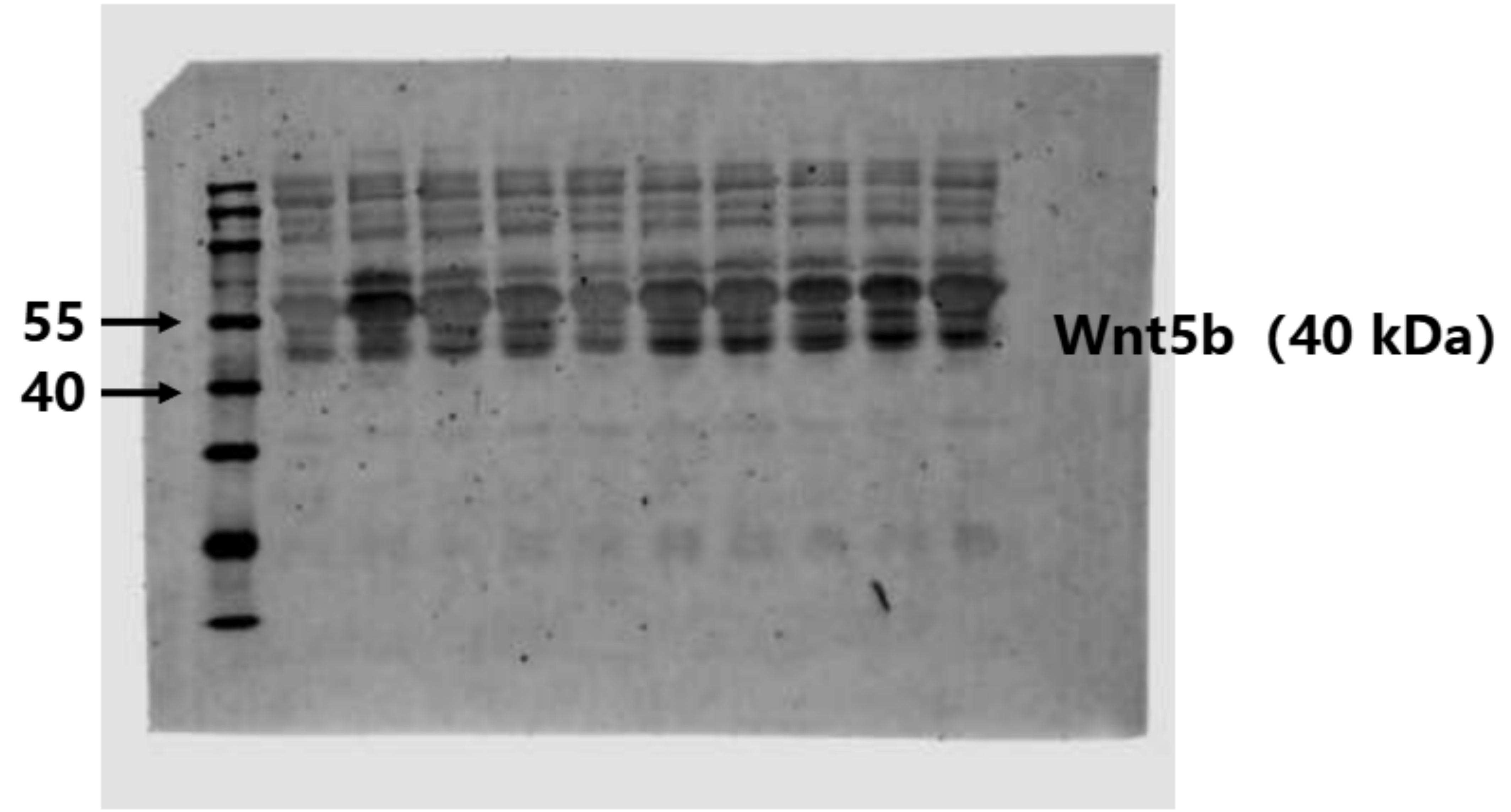

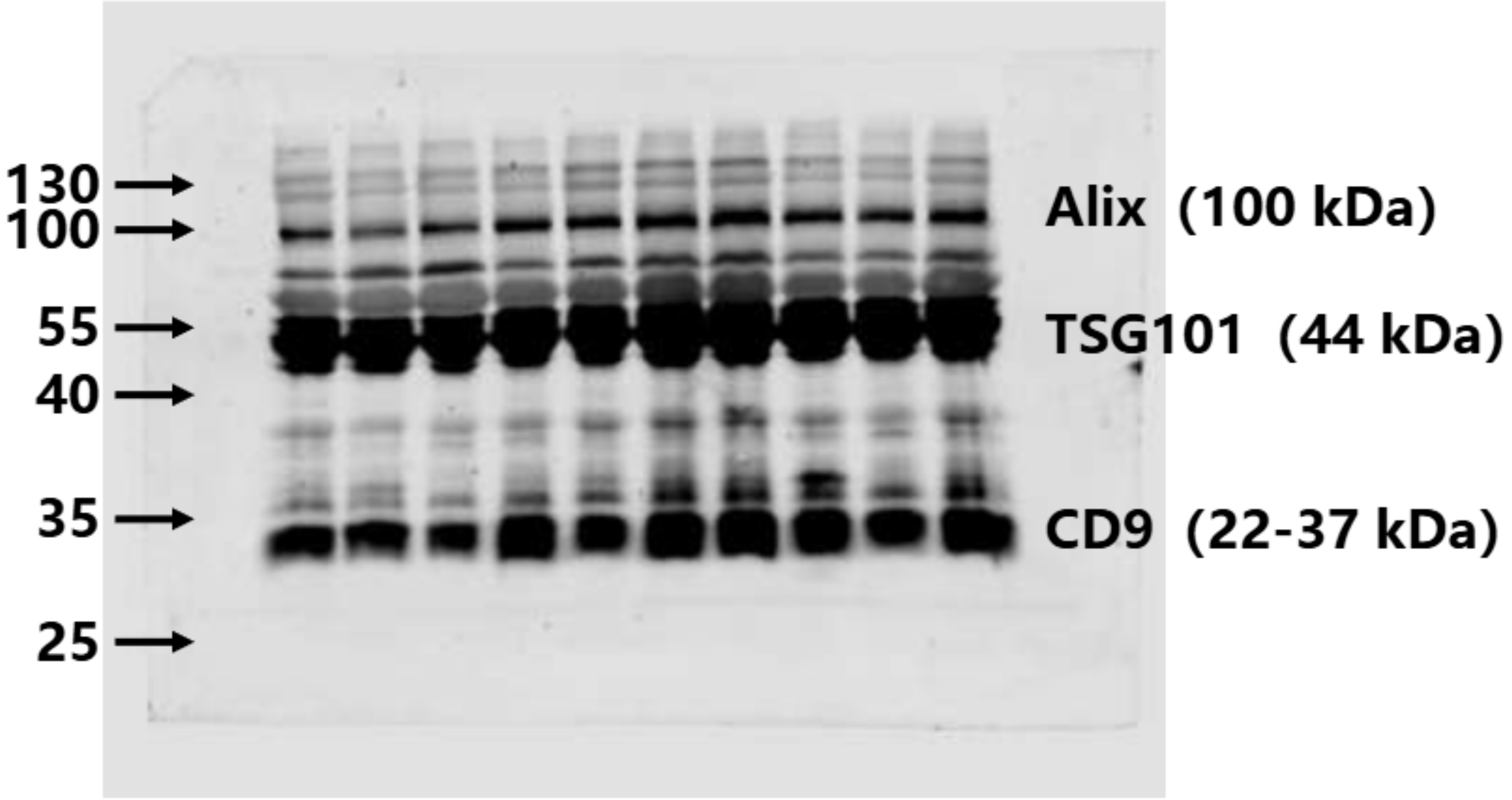

Lanes of the unedited blot correspond to those shown in the cropped images within the manuscript.

Full unedited blot for Figure 6B

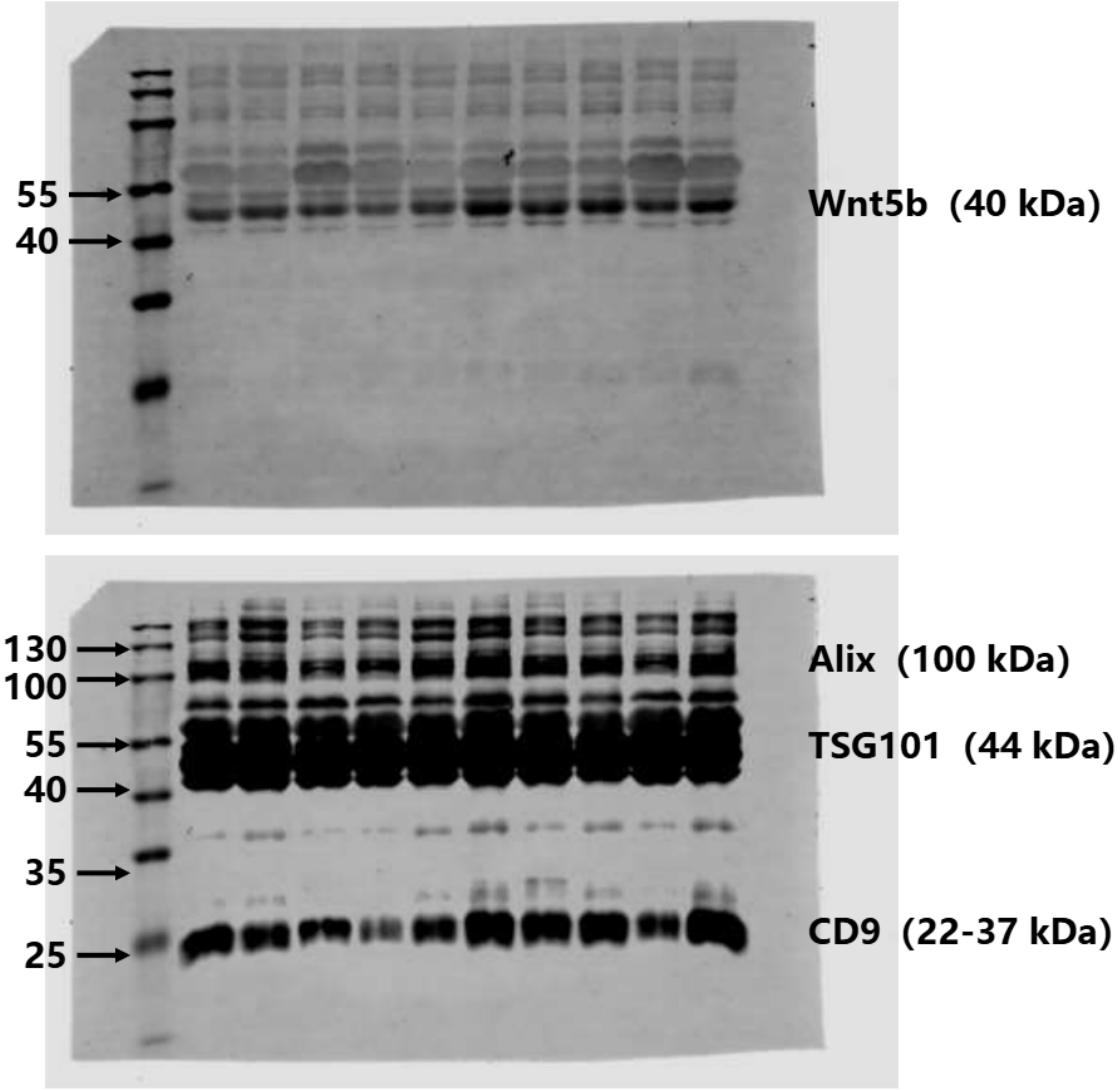

Lanes of the unedited blot correspond to those shown in the cropped images within the manuscript.

Full unedited blot for Figure 6C

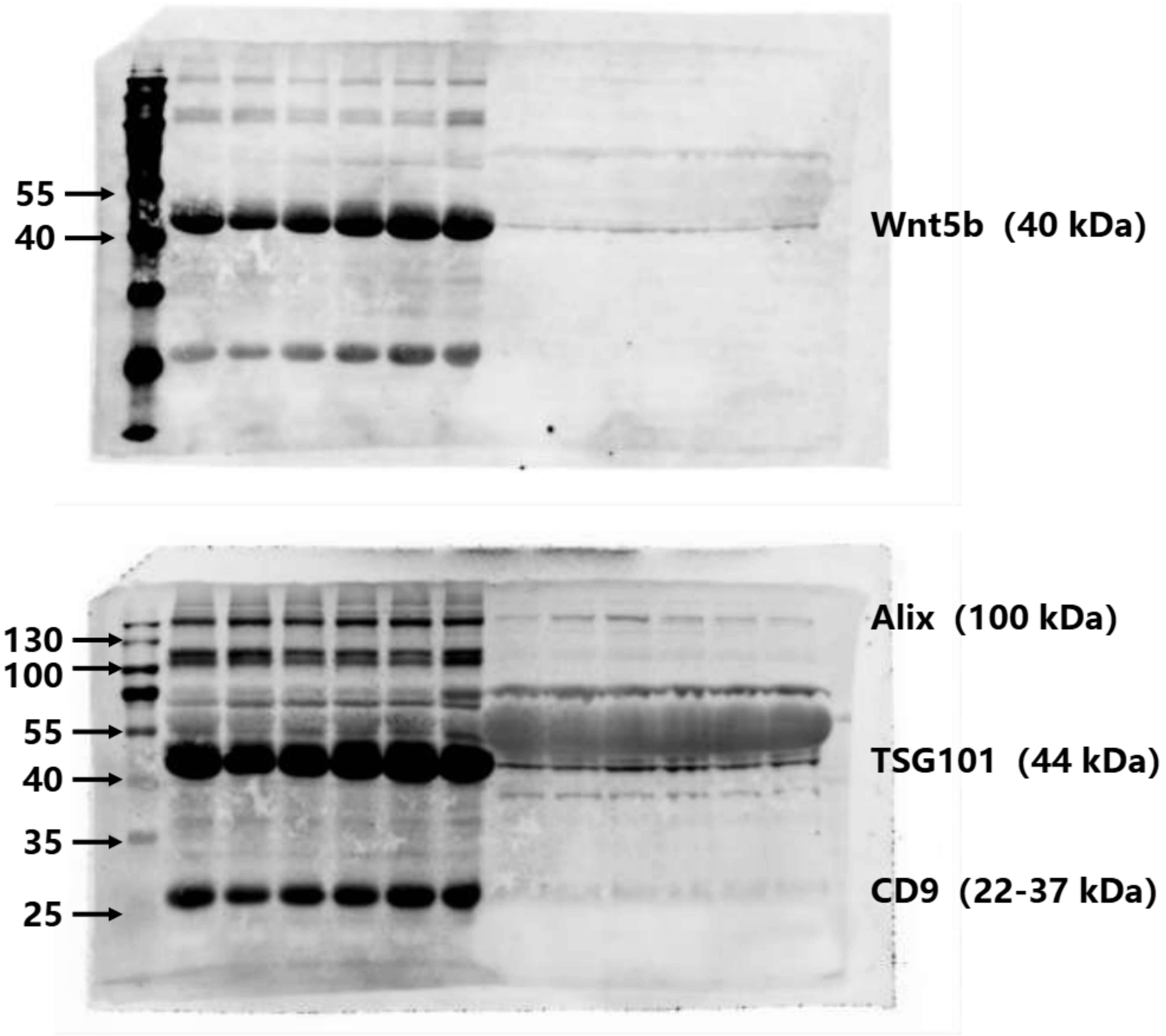

Lanes of the unedited blot correspond to those shown in the cropped images within the manuscript.

Full unedited blot for Figure 6D

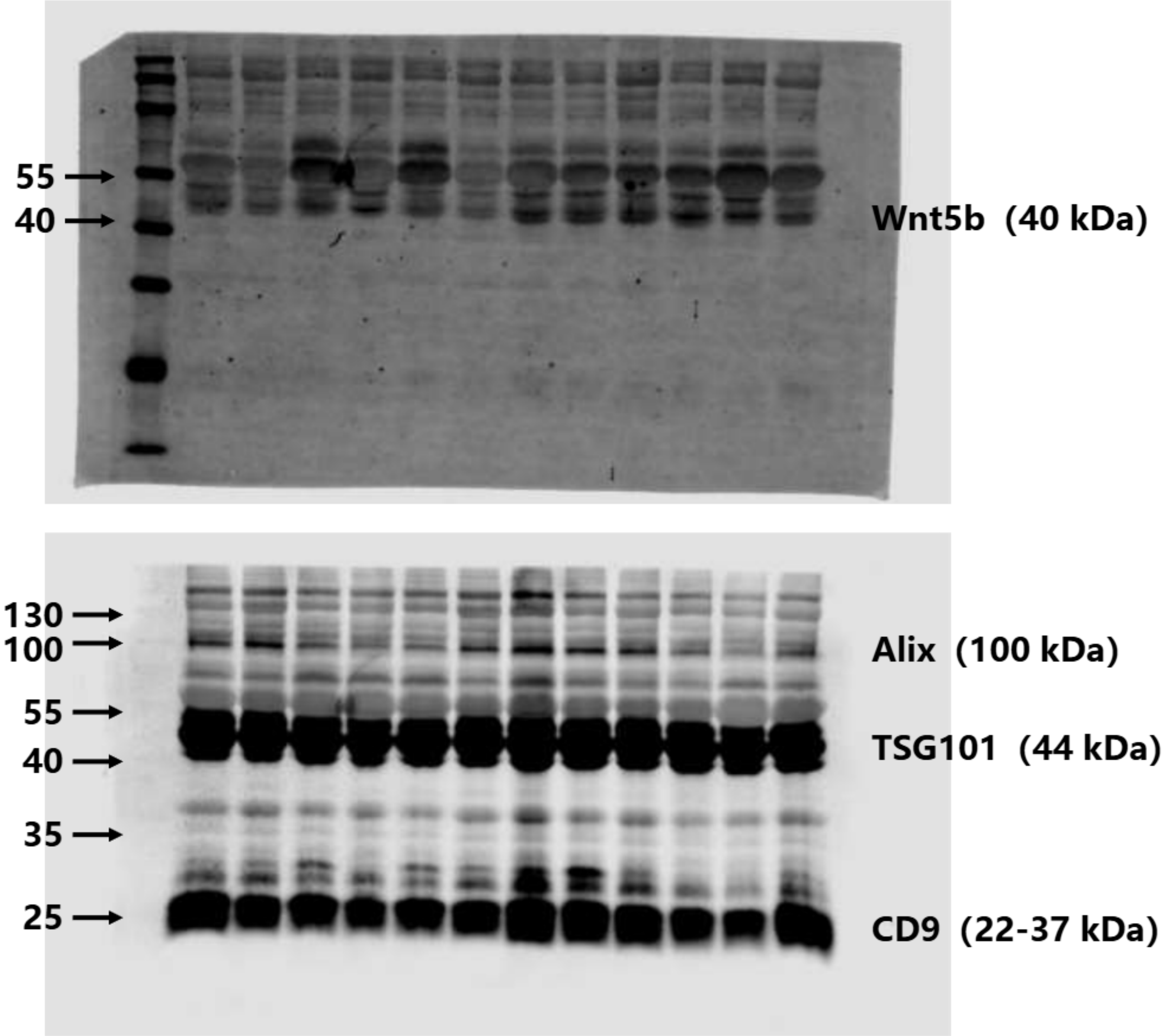

Lanes of the unedited blot correspond to those shown in the cropped images within the manuscript.

Full unedited blot for Figure 6E

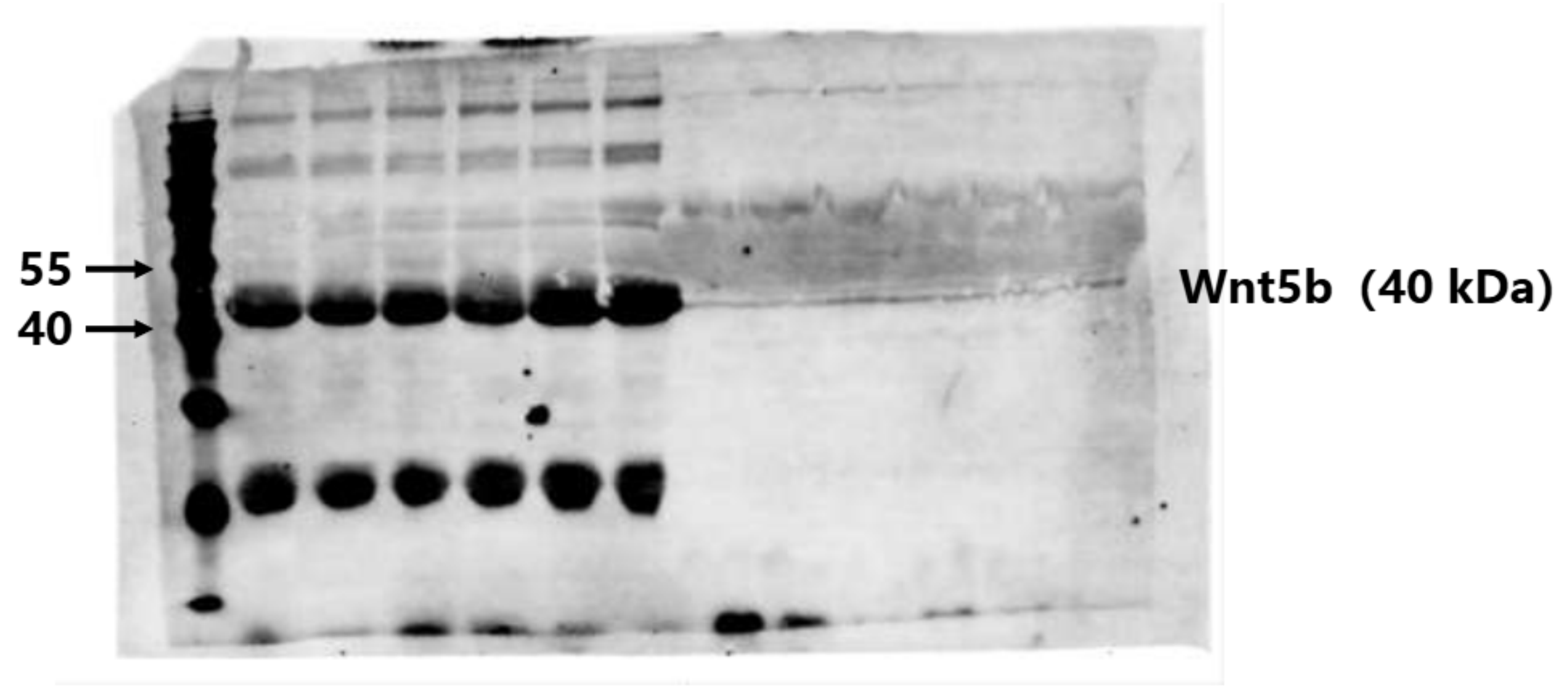

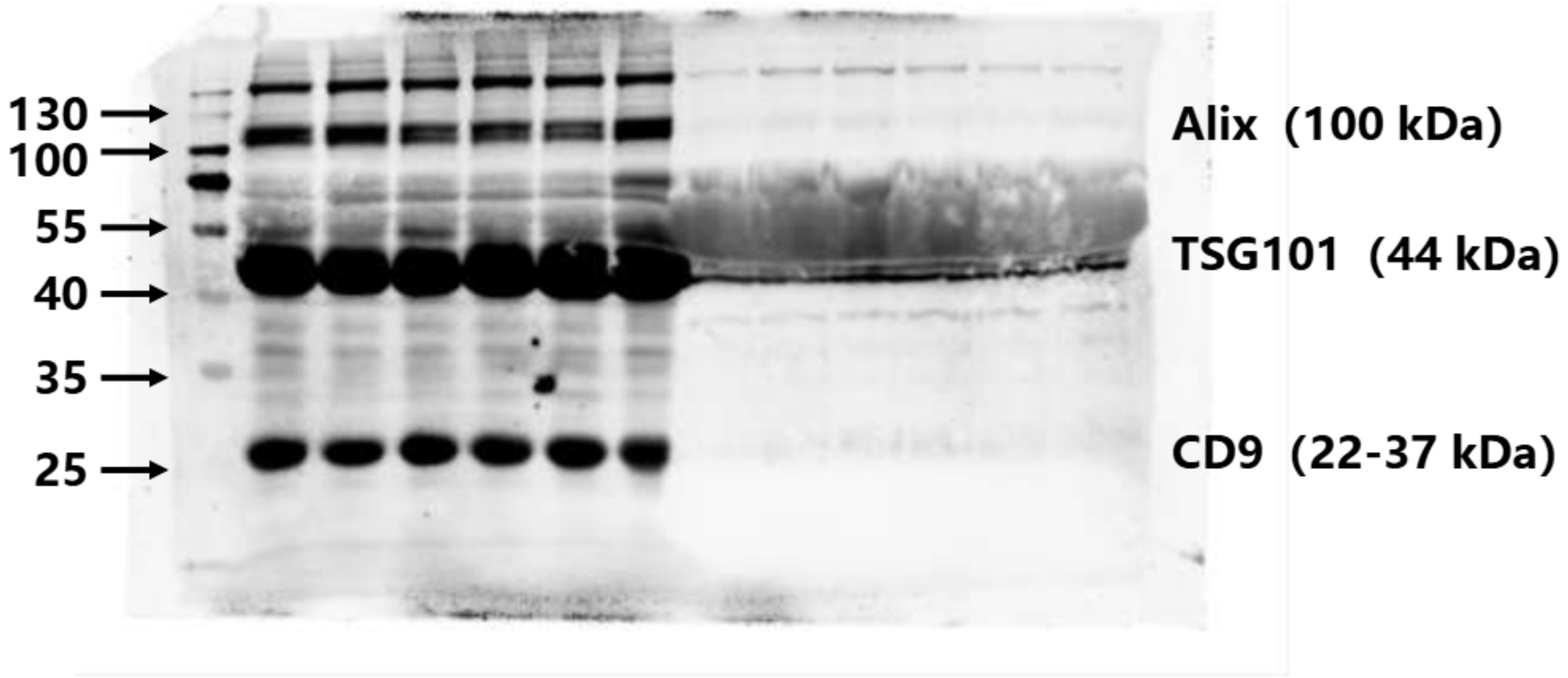

Lanes of the unedited blot correspond to those shown in the cropped images within the manuscript.

Full unedited blot for Figure 6F

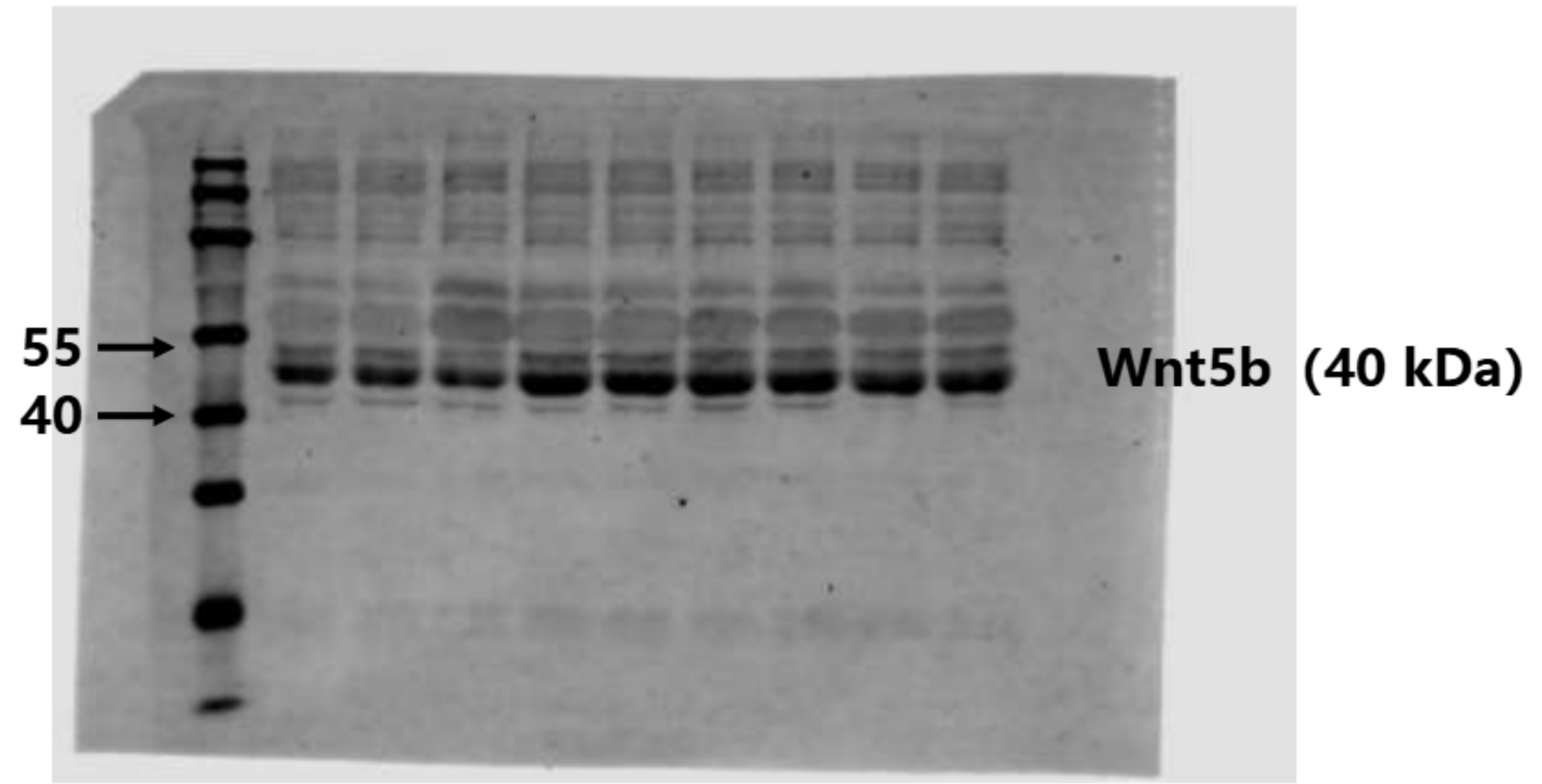

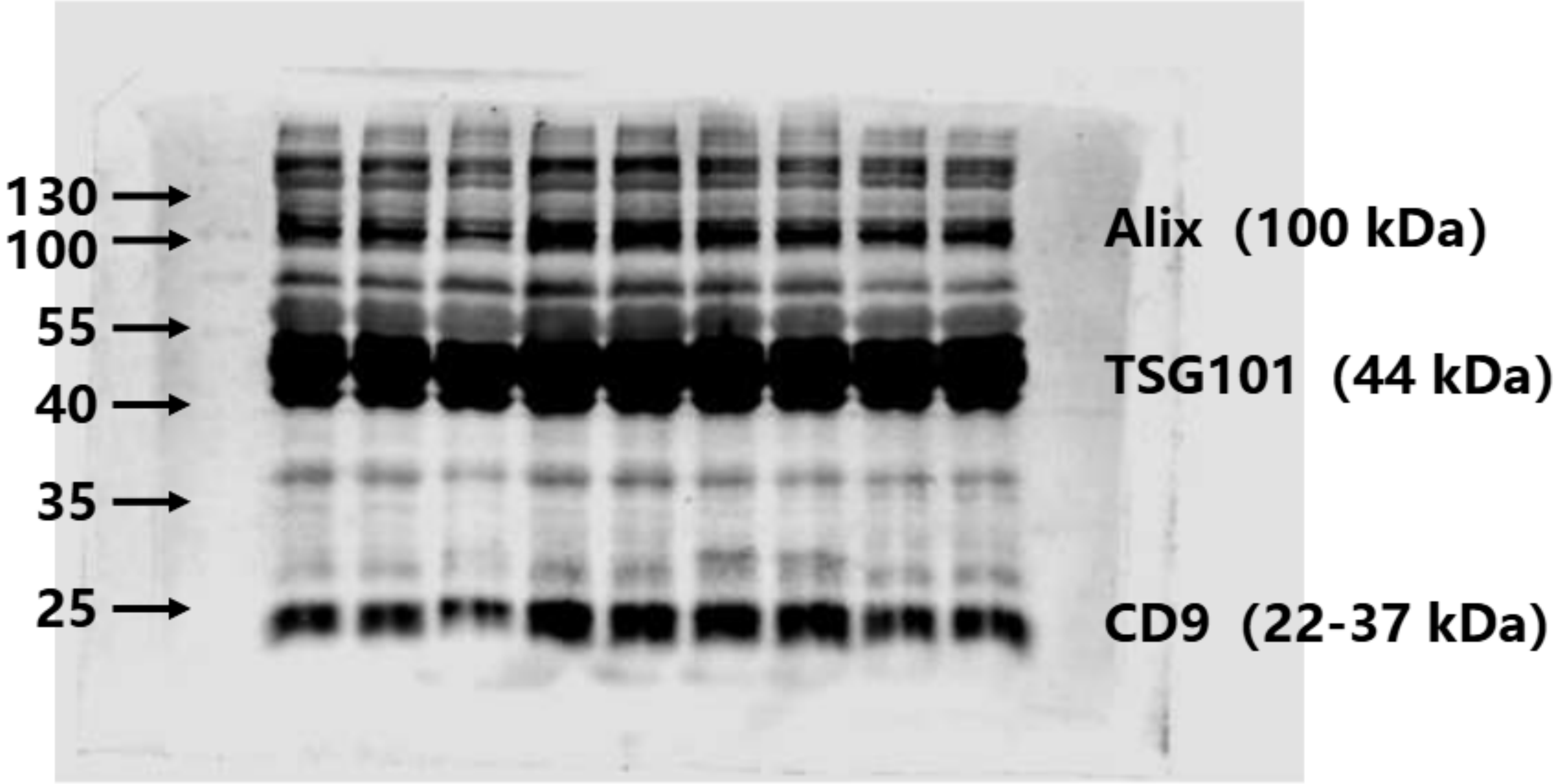

Lanes of the unedited blot correspond to those shown in the cropped images within the manuscript.

Full unedited blot for Figure 6H

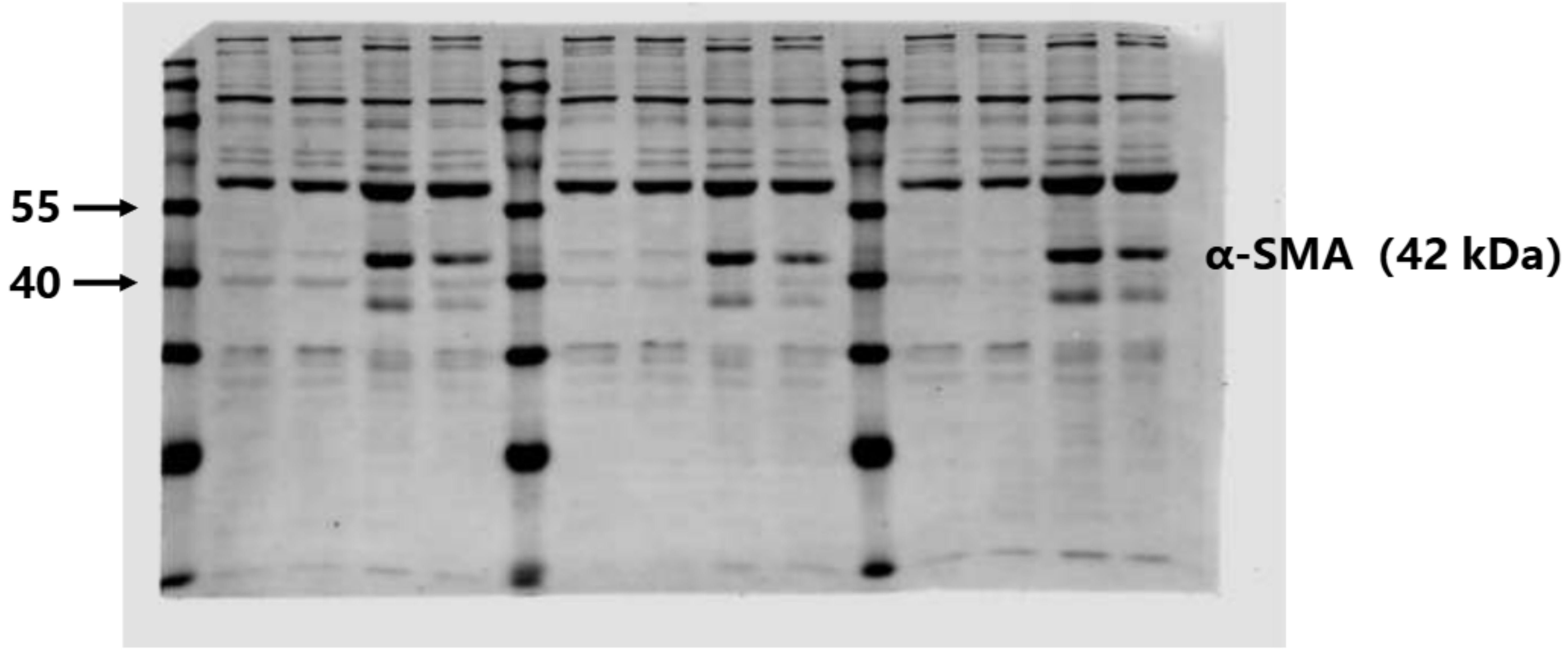

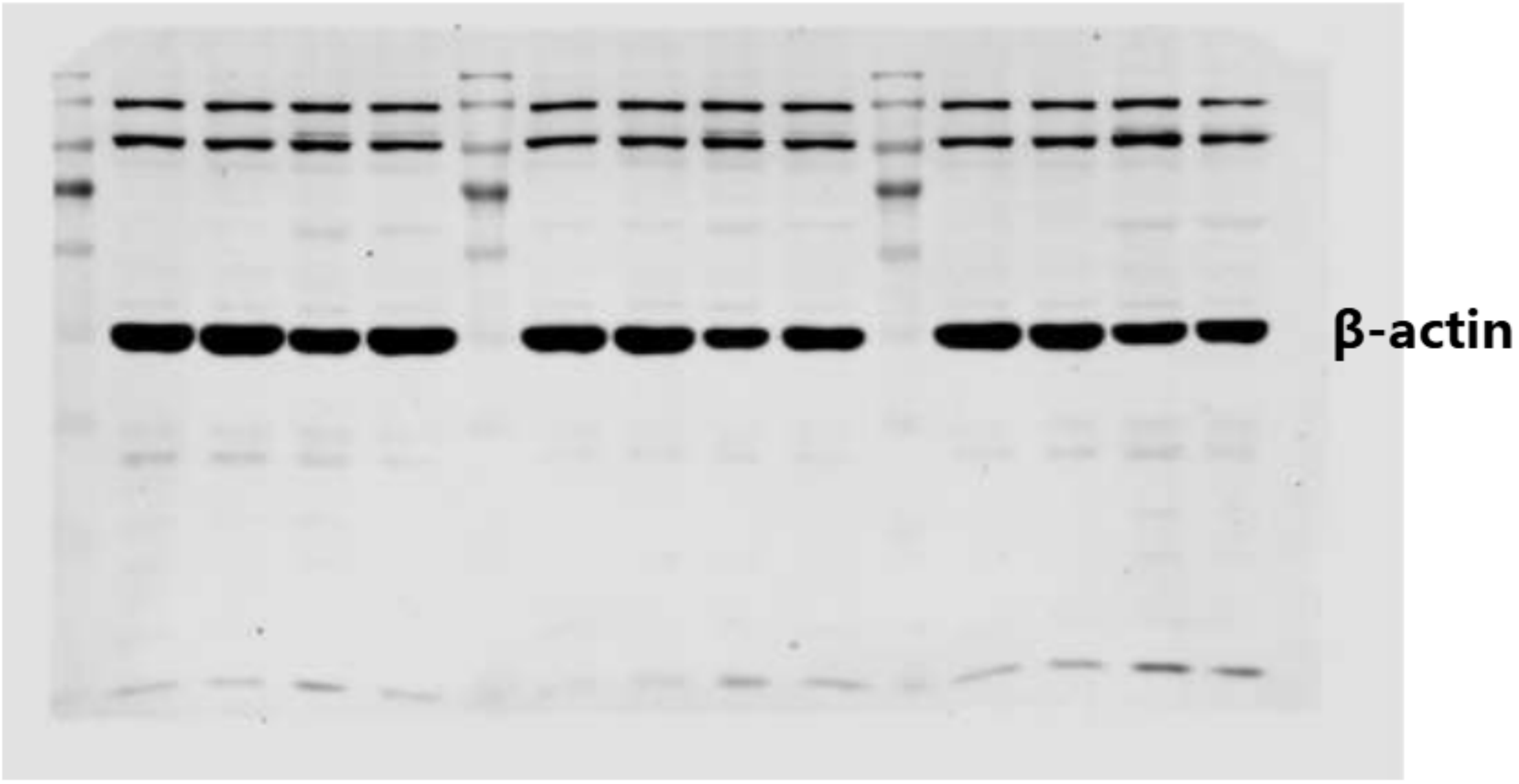

Lanes 12-15 of the unedited blot correspond to those shown in the cropped images within the manuscript.

Full unedited blot for Figure 6H

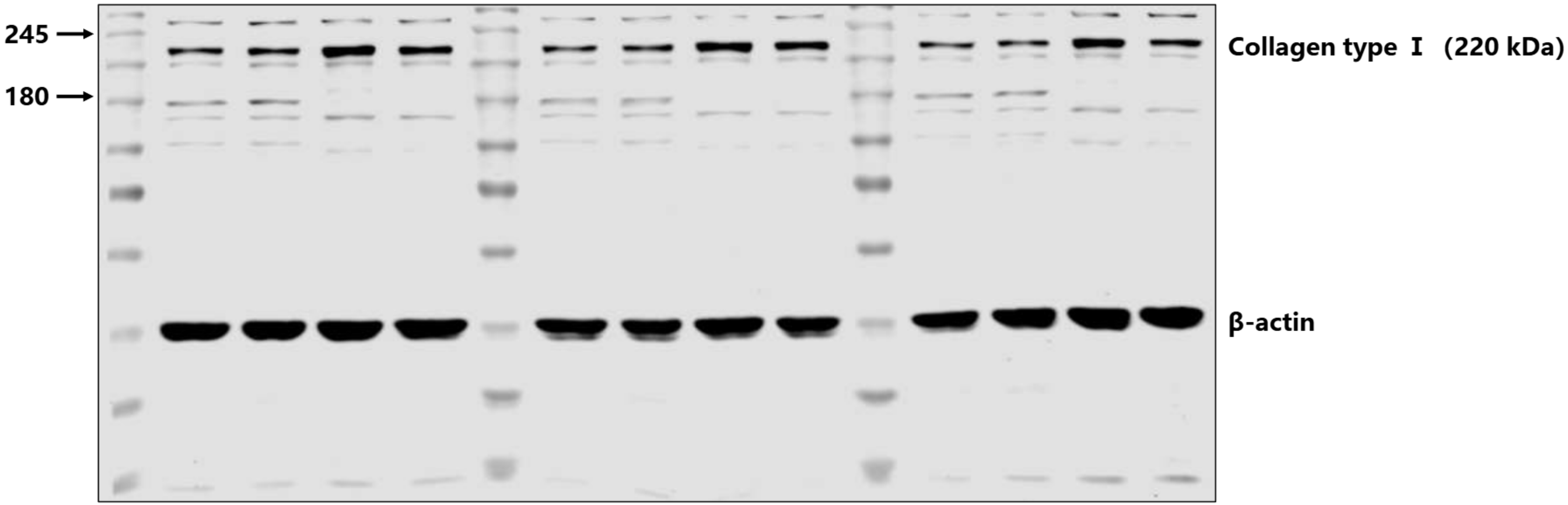

Lanes 2-5 of the unedited blot correspond to those shown in the cropped images within the manuscript.

Full unedited blot for Figure 6H

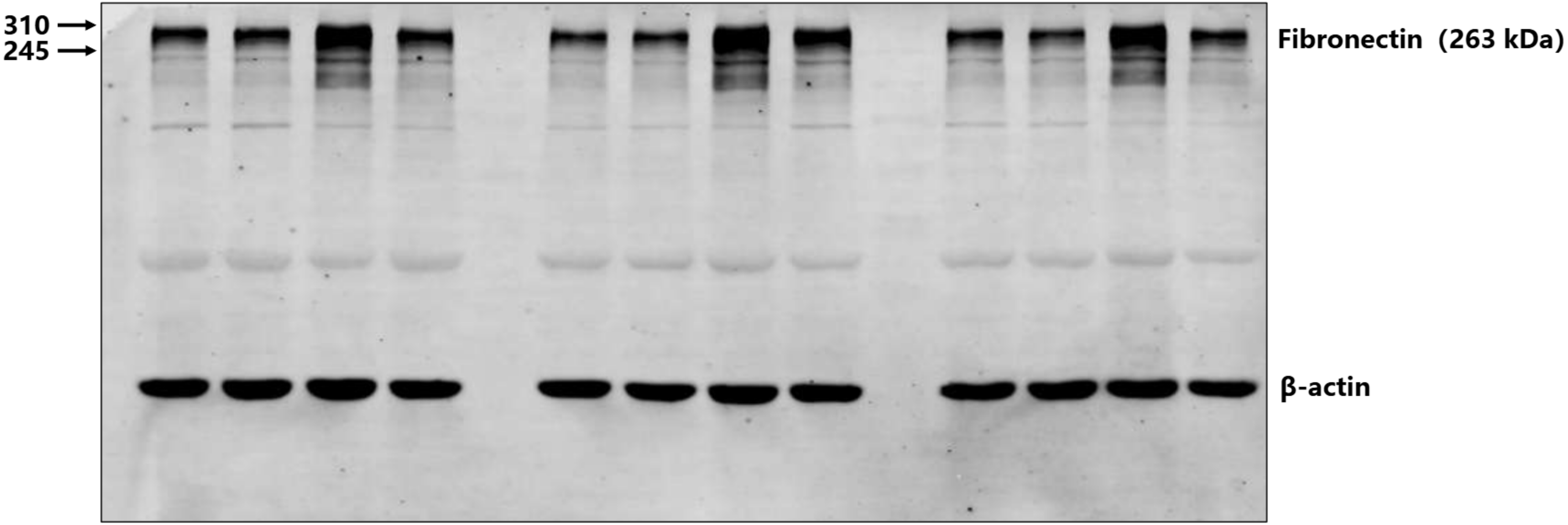

Lanes 7-10 of the unedited blot correspond to those shown in the cropped images within the manuscript.

Full unedited blot for Figure S2C

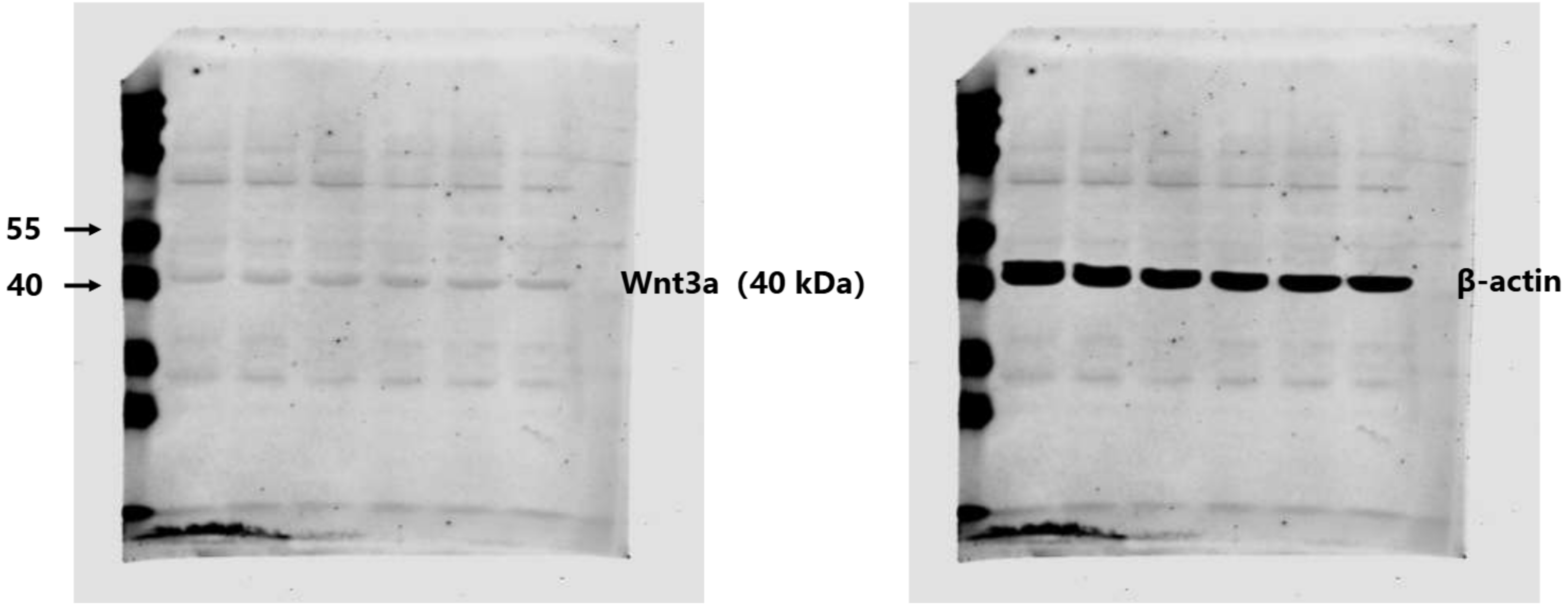

Lanes 4-5 of the unedited blot correspond to those shown in the cropped images within the supplementary materials.

Full unedited blot for Figure S2C

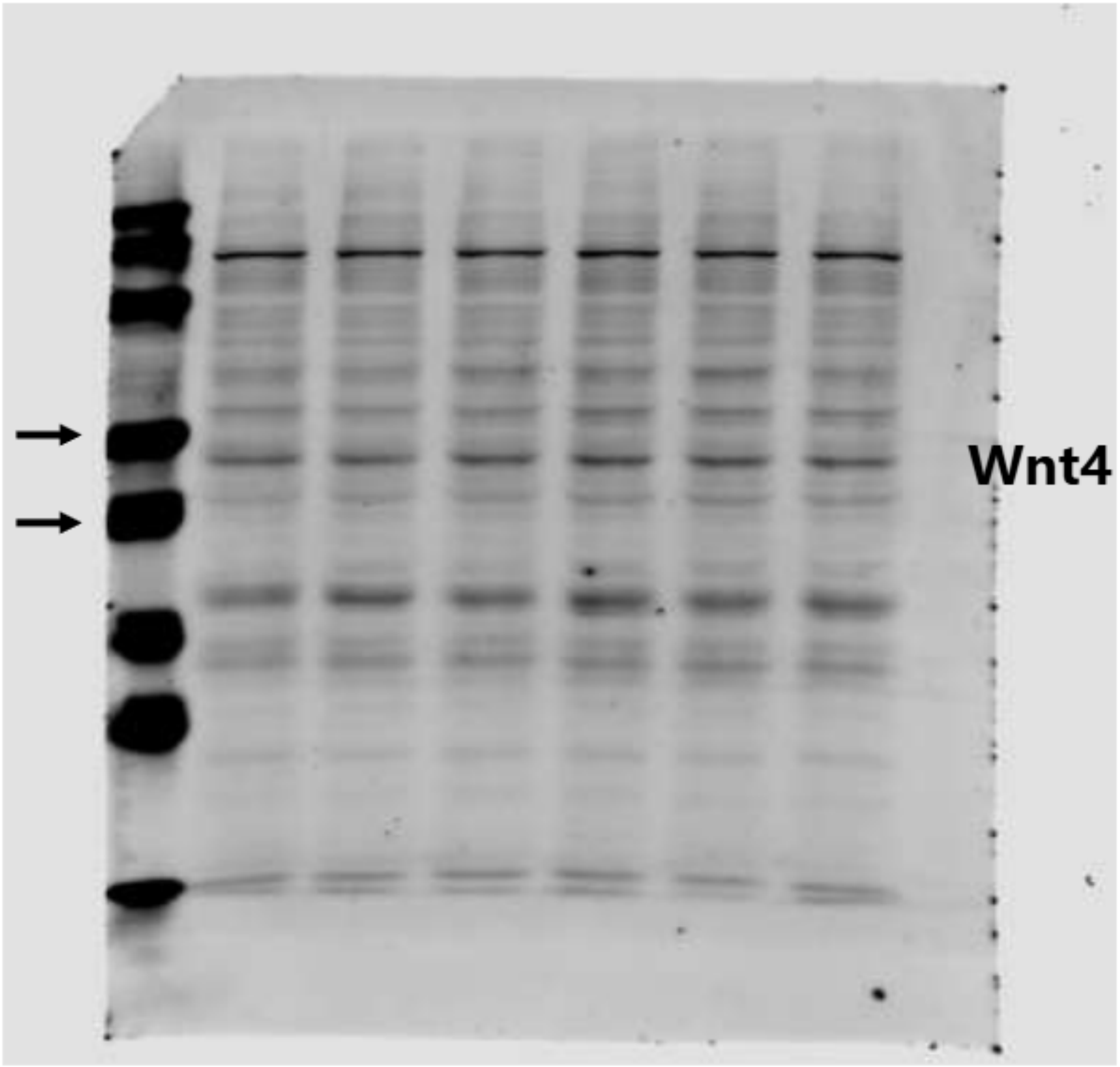

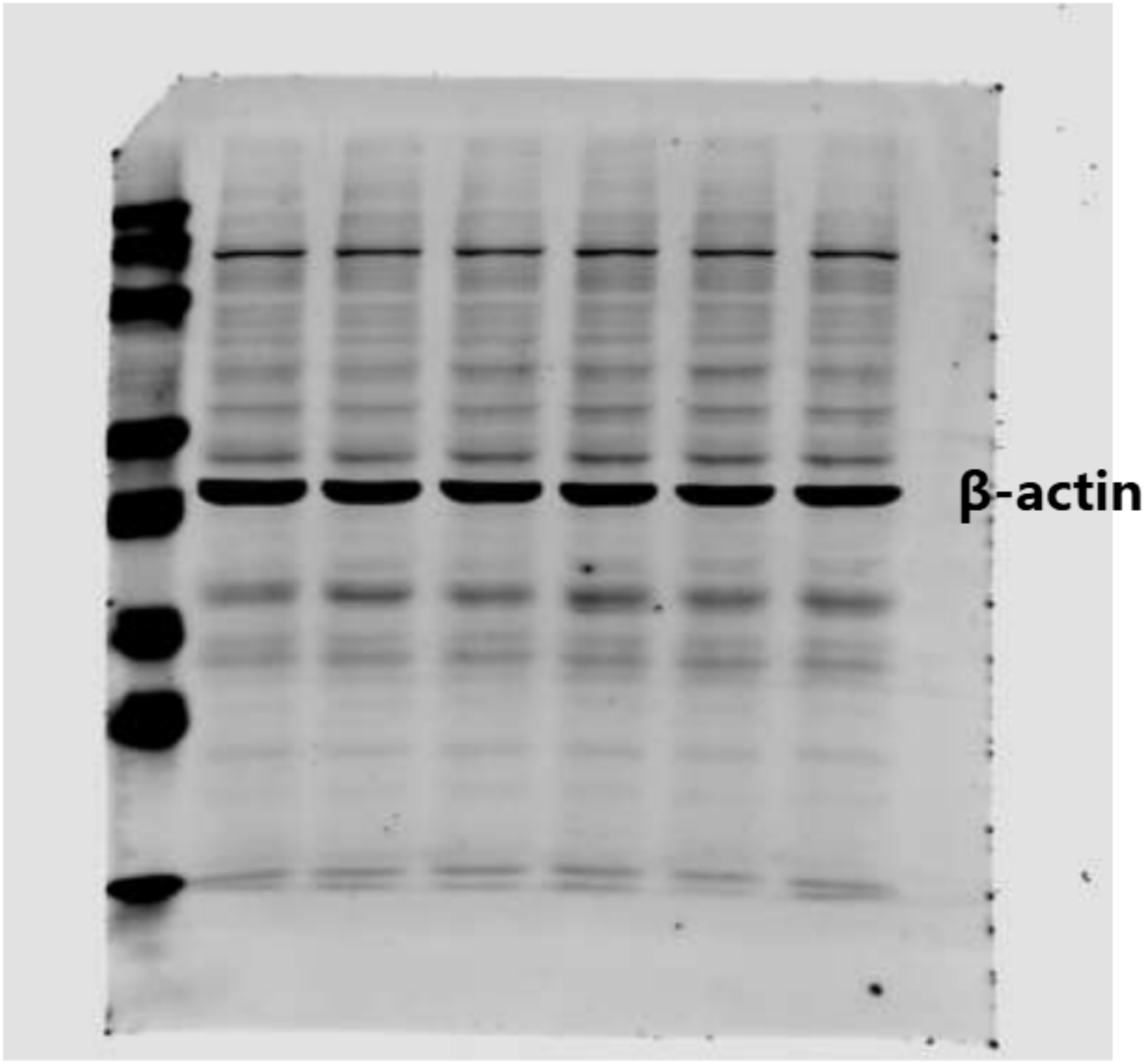

Lanes 4-5 of the unedited blot correspond to those shown in the cropped images within the supplementary materials.

Full unedited blot for Figure S2C

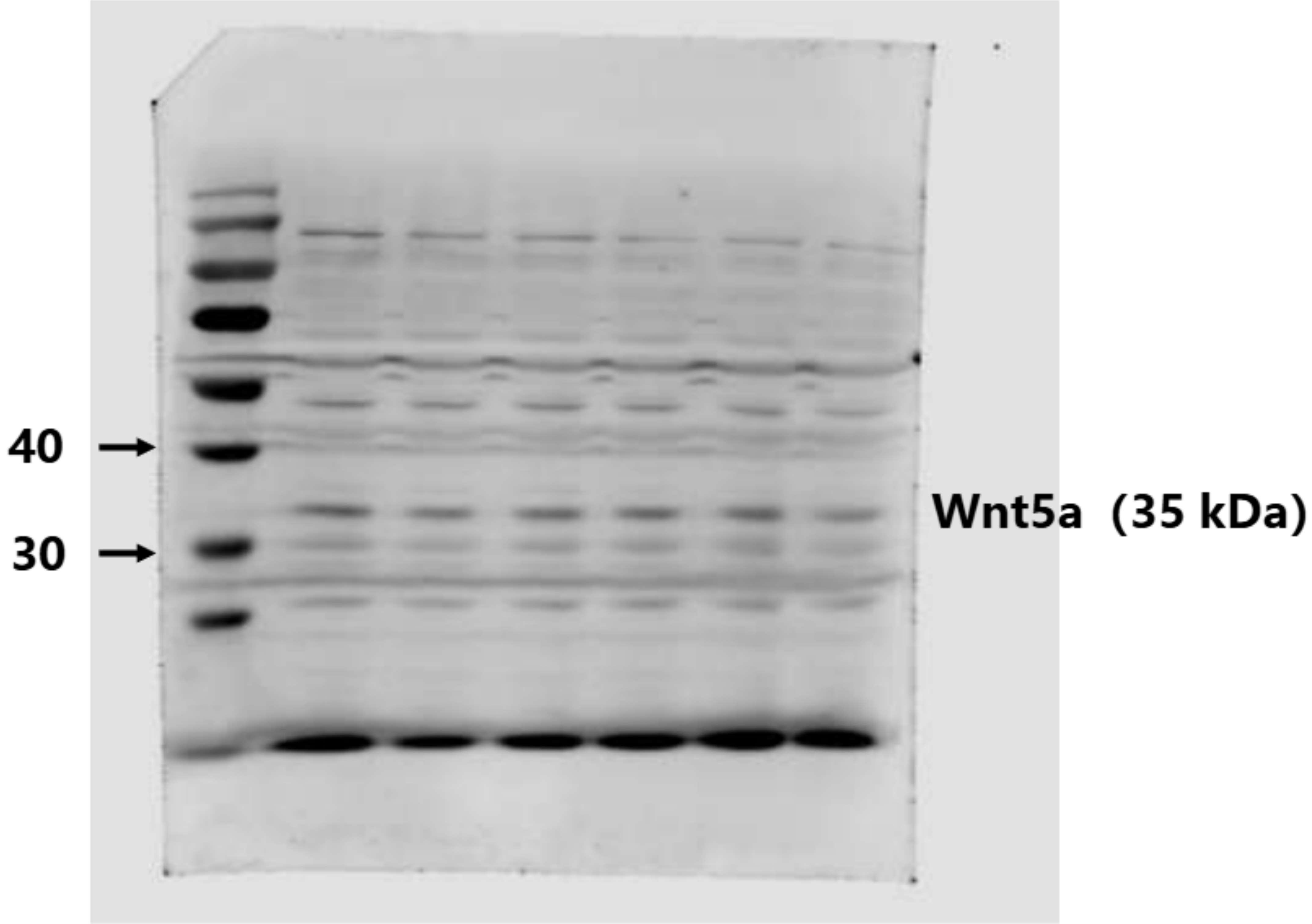

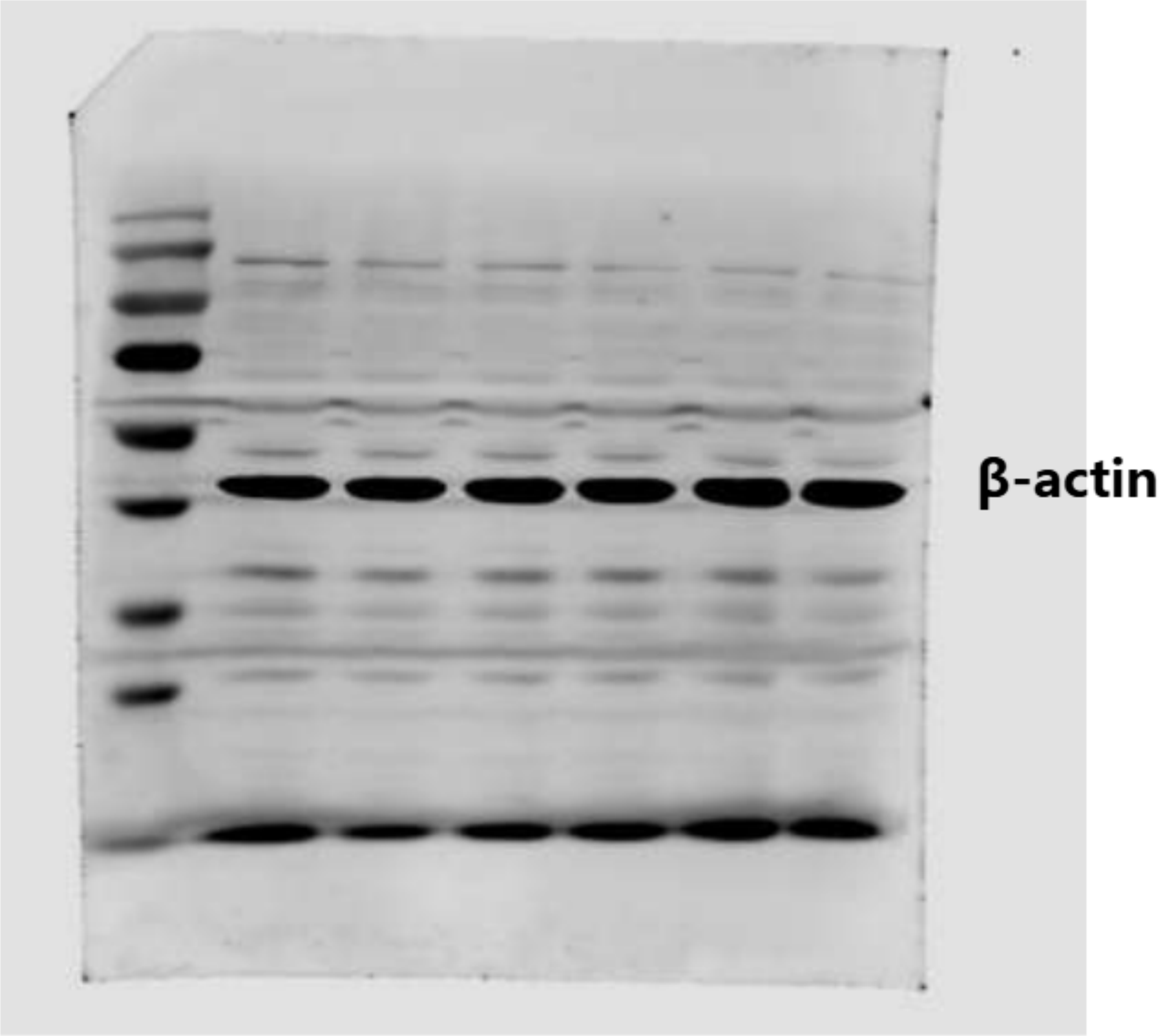

Lanes 4-5 of the unedited blot correspond to those shown in the cropped images within the supplementary materials.

Full unedited blot for Figure S2C

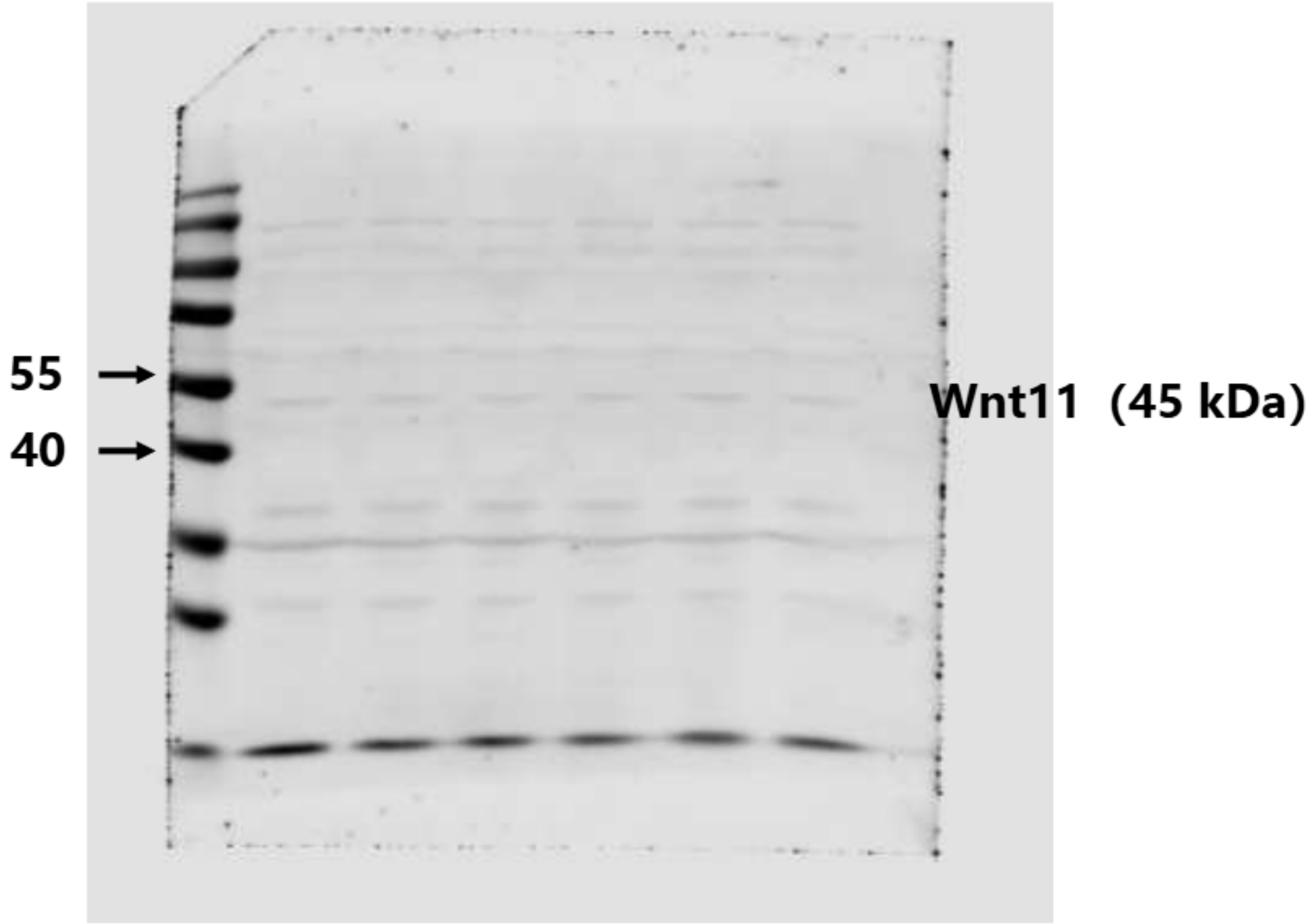

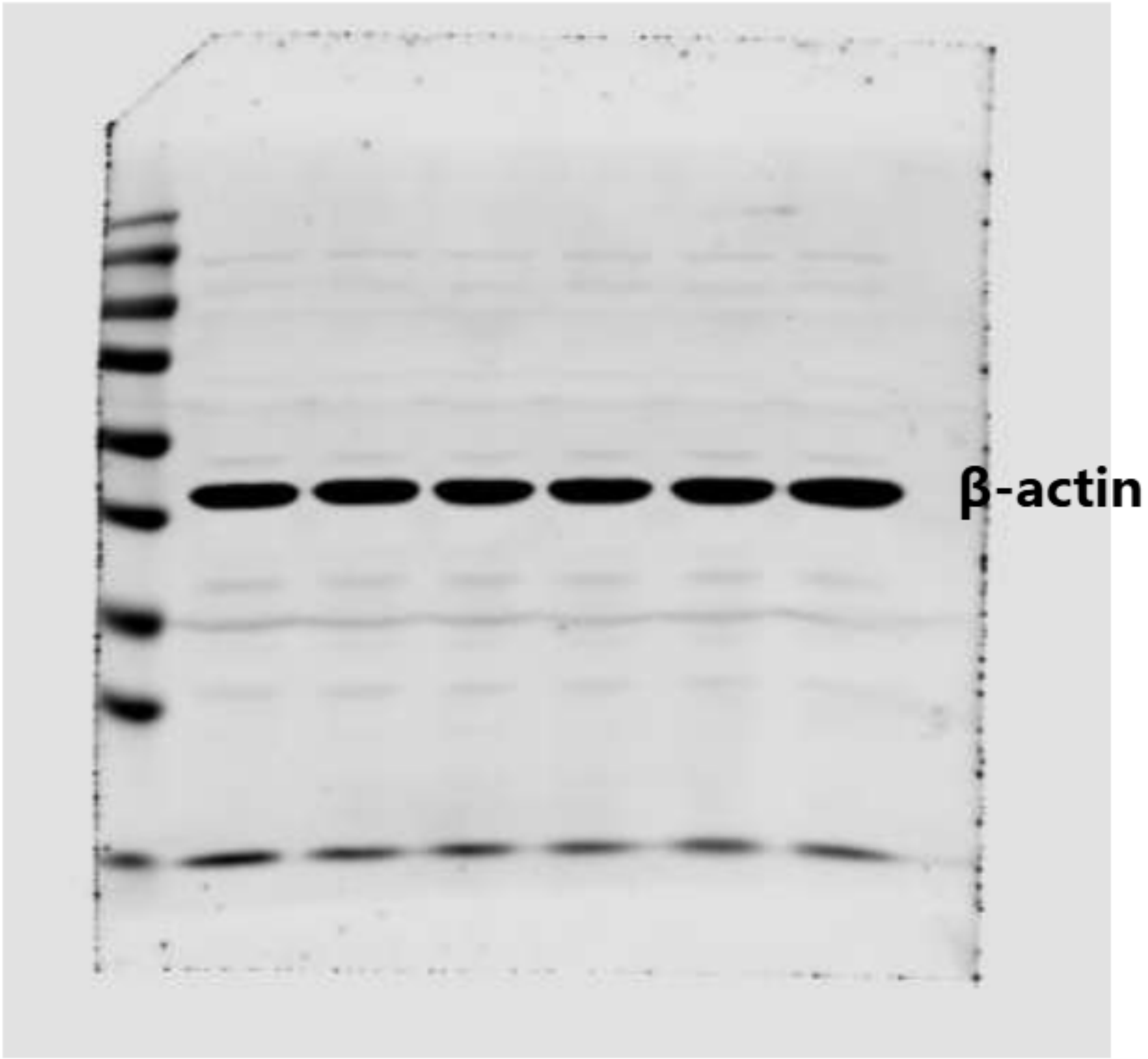

Lanes 4-5 of the unedited blot correspond to those shown in the cropped images within the supplementary materials.

Full unedited blot for Figure S2D

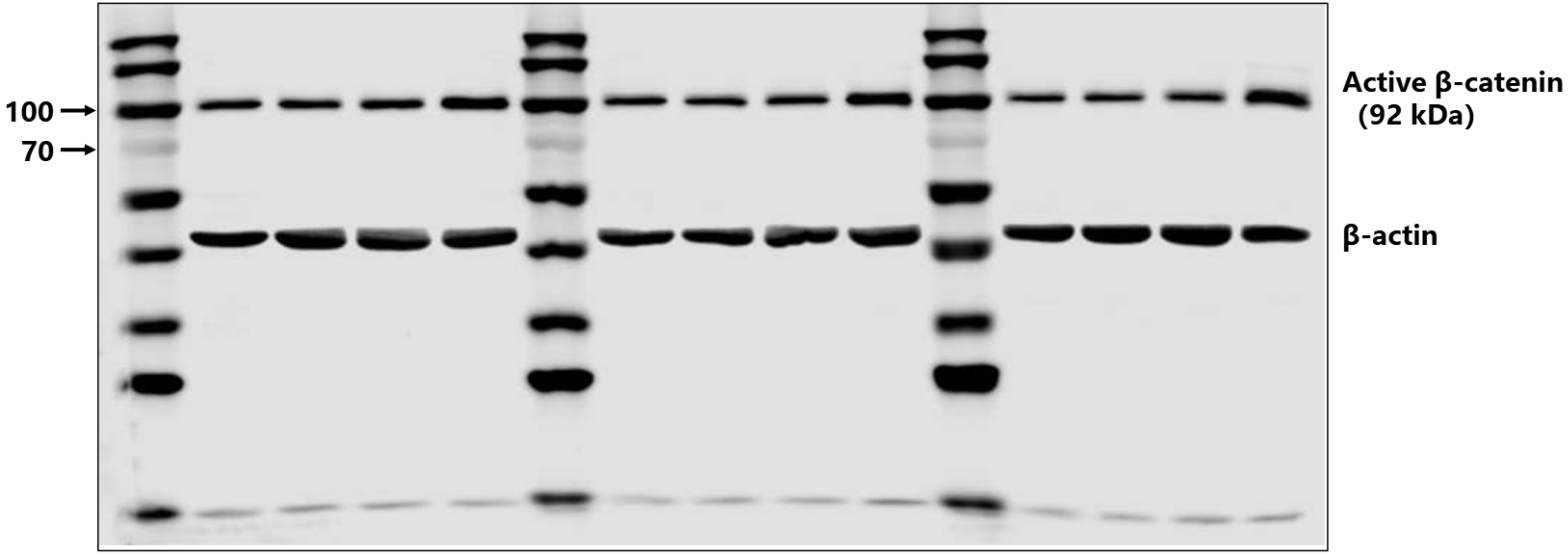

Lanes 12-15 of the unedited blot correspond to those shown in the cropped images within the supplementary materials.

Full unedited blot for Figure S3B

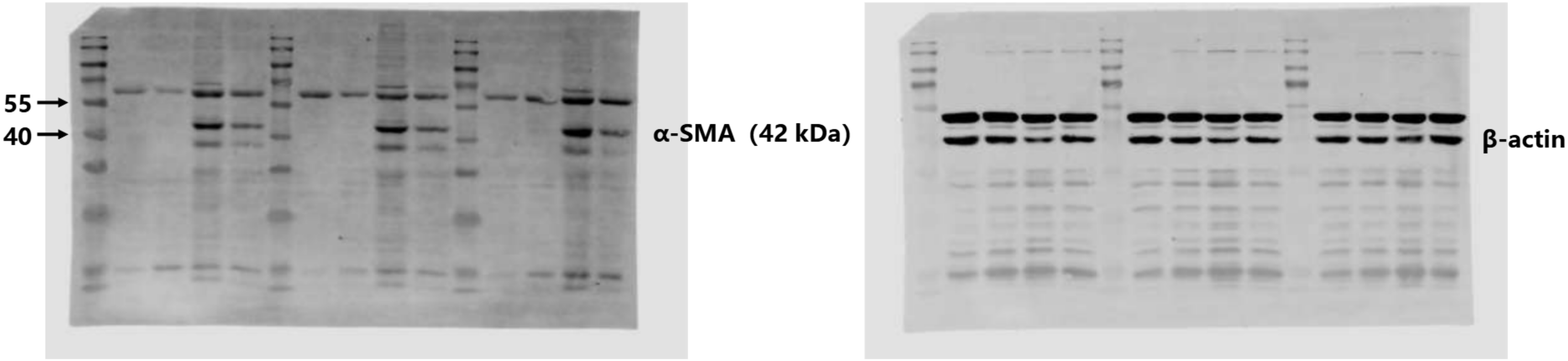

Lanes 2-5 of the unedited blot correspond to those shown in the cropped images within the supplementary materials.

Full unedited blot for Figure S3B

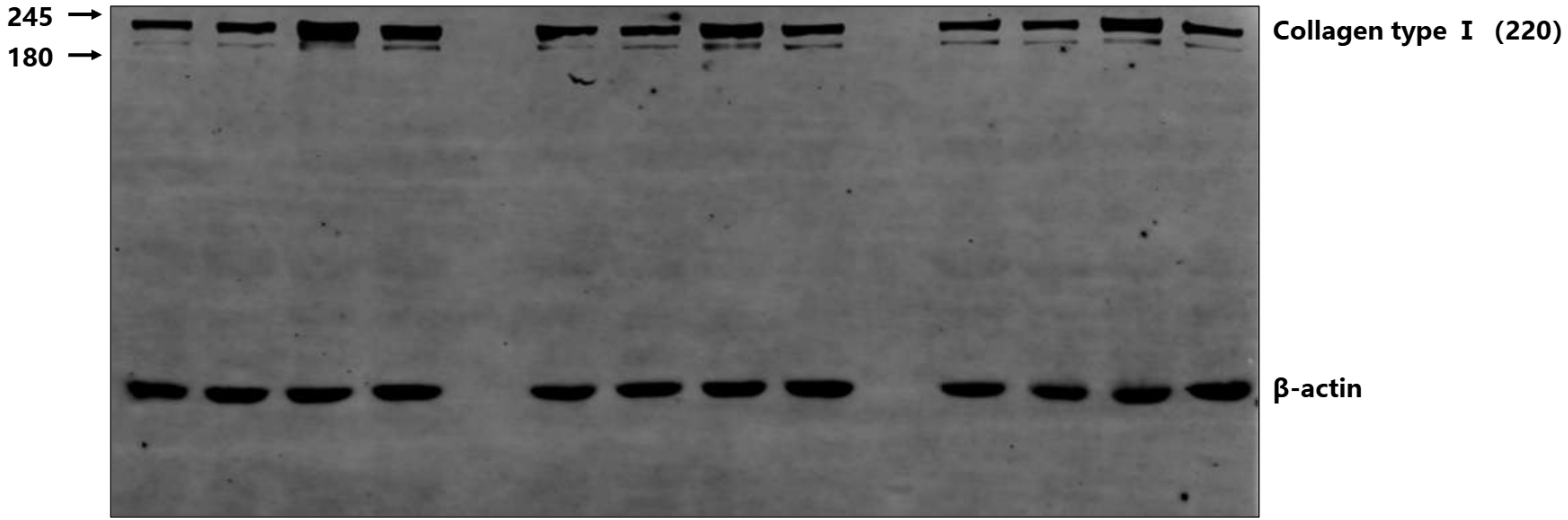

Lanes 1-4 of the unedited blot correspond to those shown in the cropped images within the supplementary materials.

Full unedited blot for Figure S3B

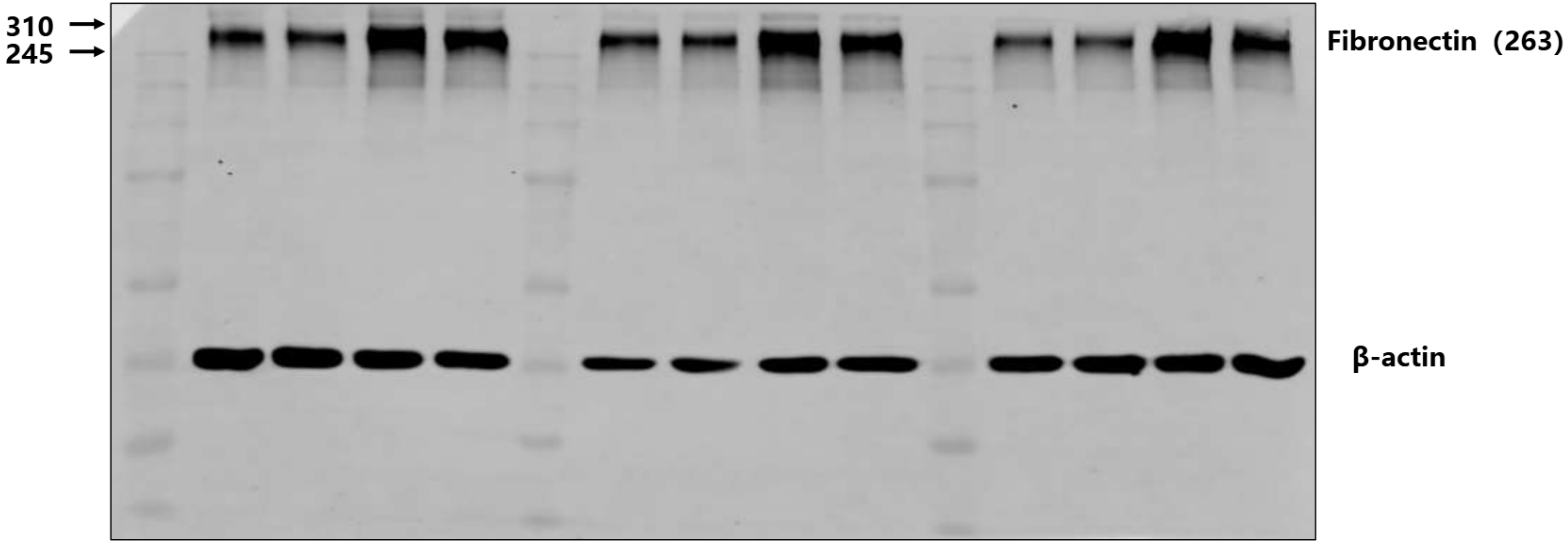

Lanes 7-10 of the unedited blot correspond to those shown in the cropped images within the supplementary materials.

Full unedited blot for Figure S3D

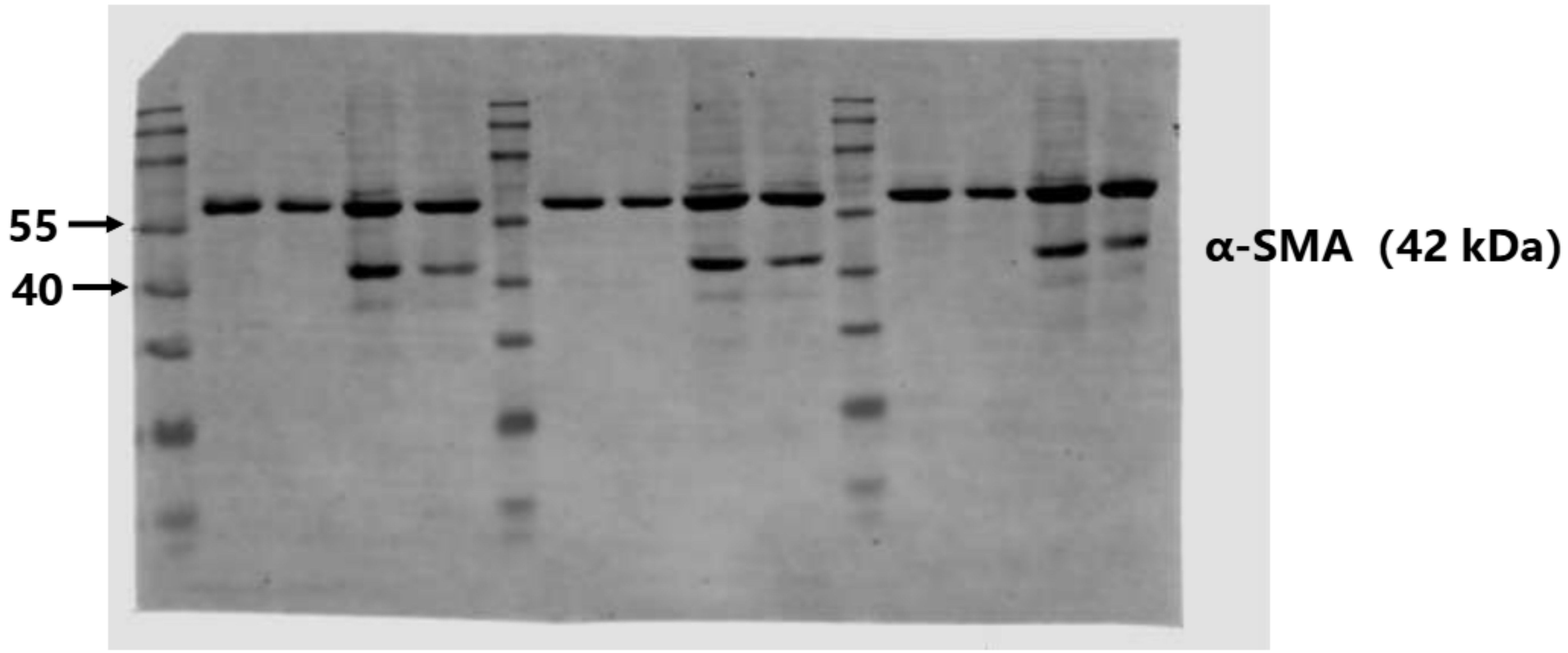

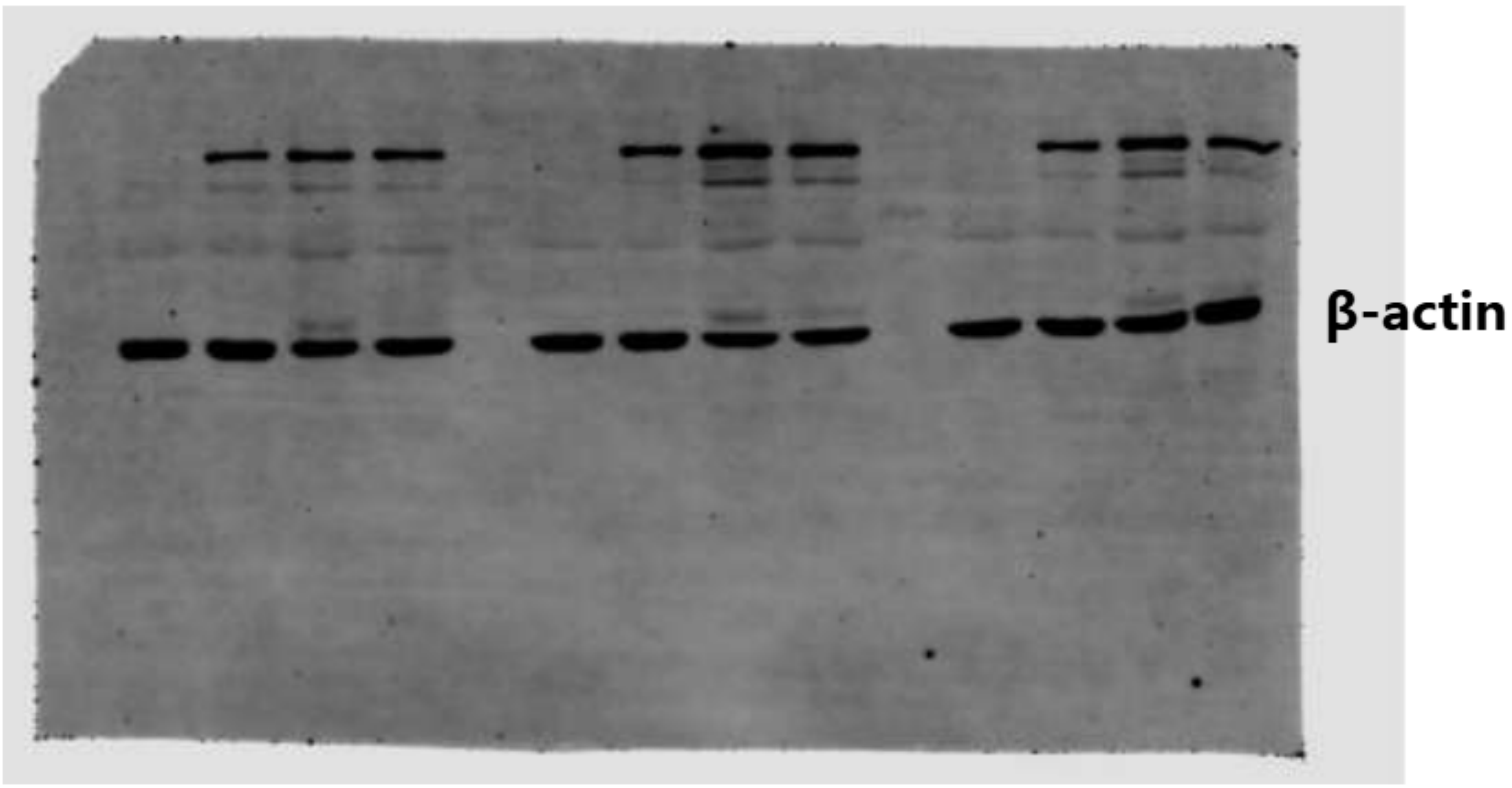

Lanes 7-10 of the unedited blot correspond to those shown in the cropped images within the supplementary materials.

Full unedited blot for Figure S3D

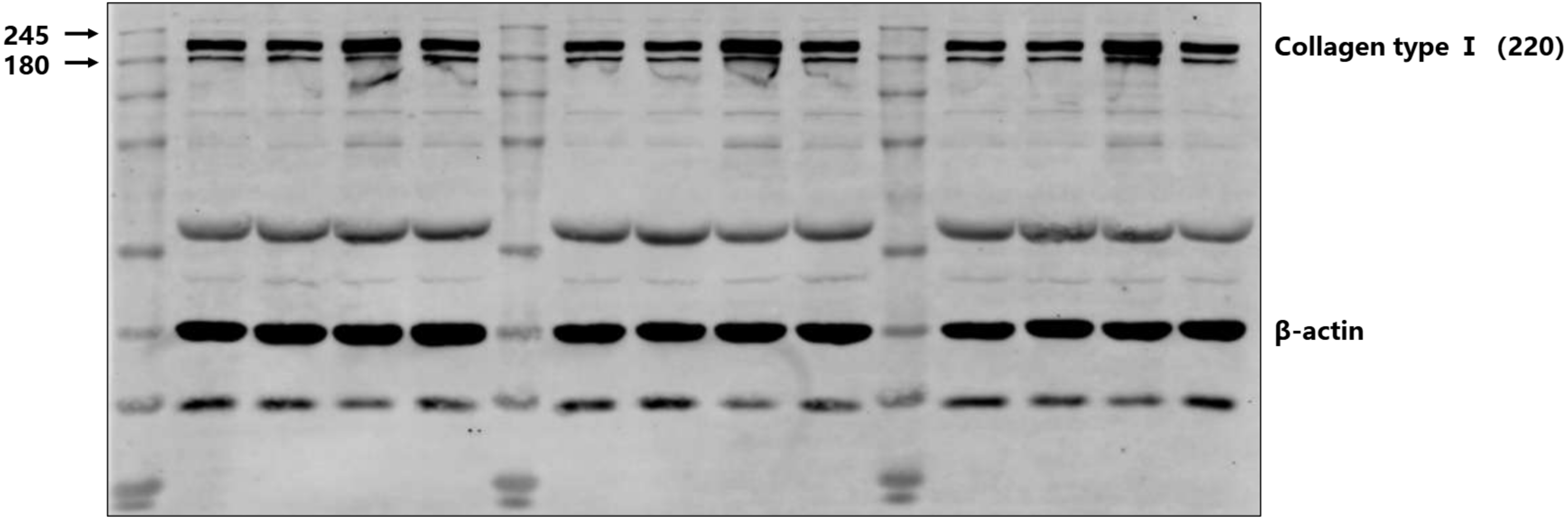

Lanes 12-15 of the unedited blot correspond to those shown in the cropped images within the supplementary materials.

Full unedited blot for Figure S3D

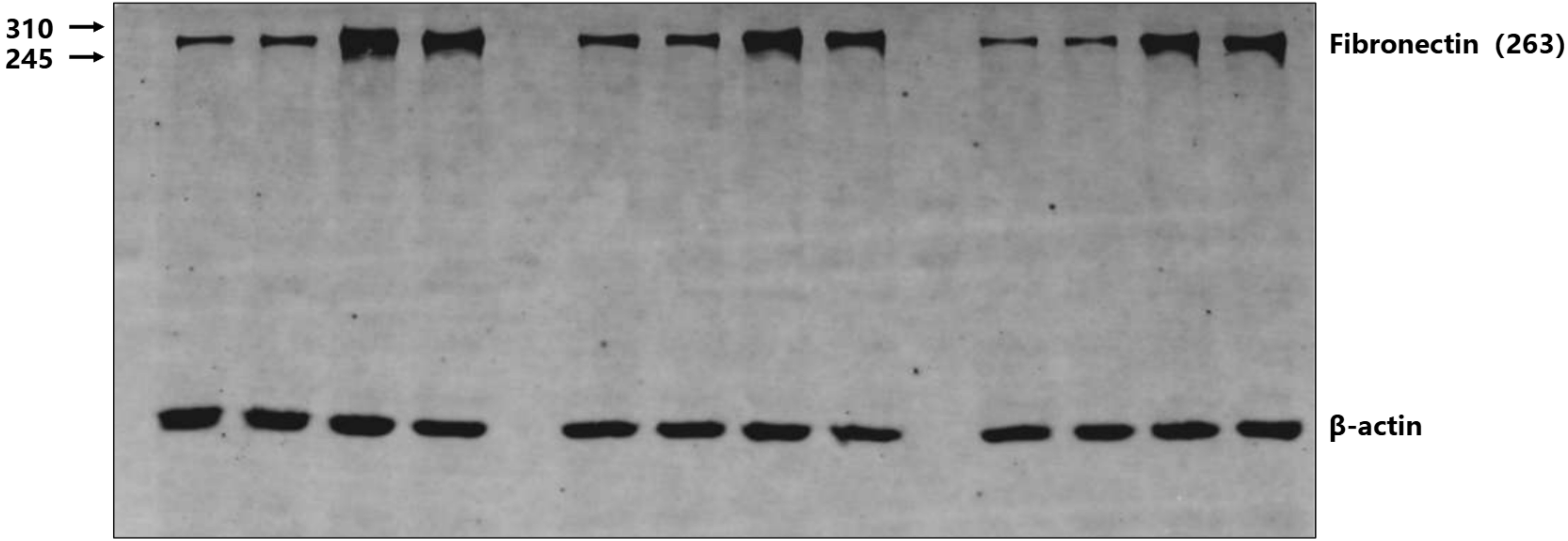

Lanes 7-10 of the unedited blot correspond to those shown in the cropped images within the supplementary materials.

Full unedited blot for Figure S3E

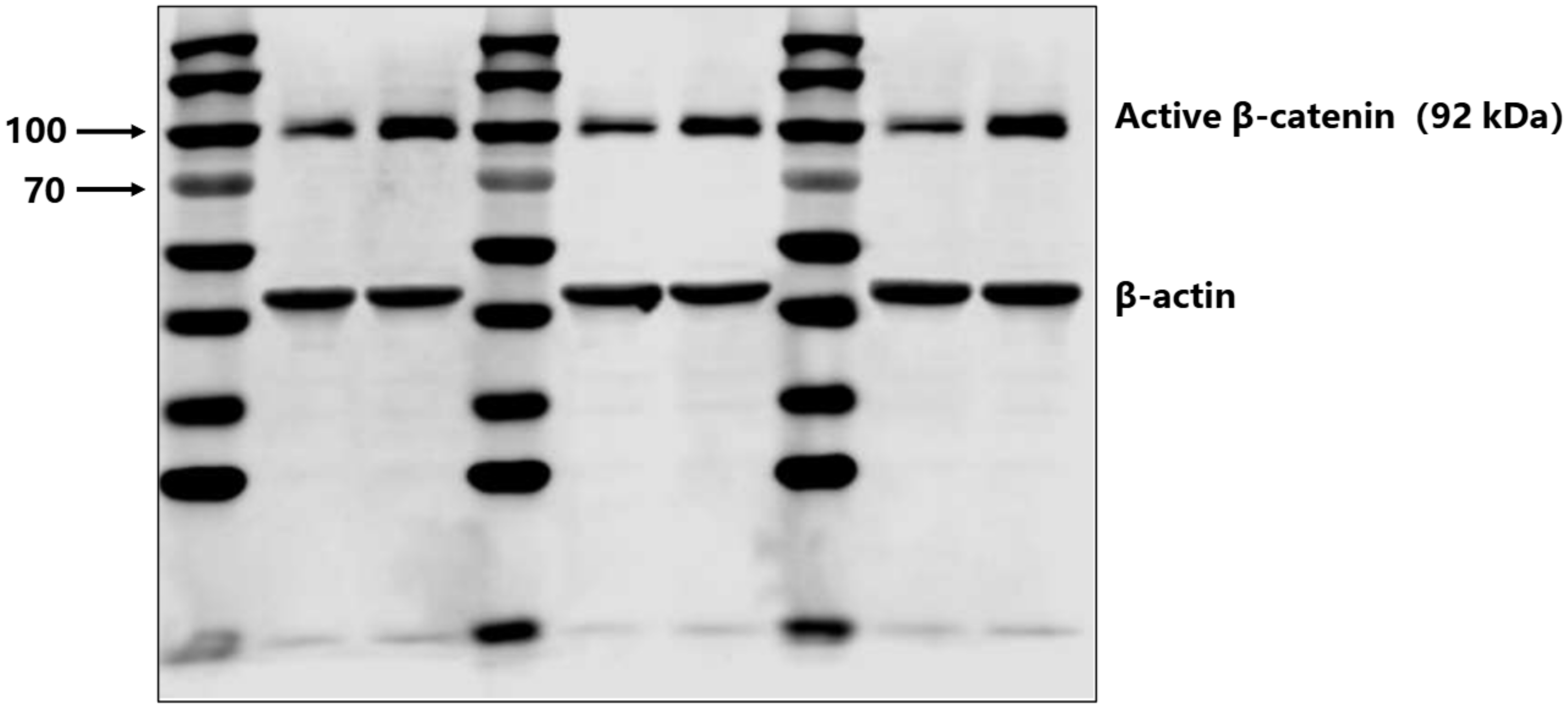

Lanes 8-9 of the unedited blot correspond to those shown in the cropped images within the supplementary materials.

Full unedited blot for Figure S4D

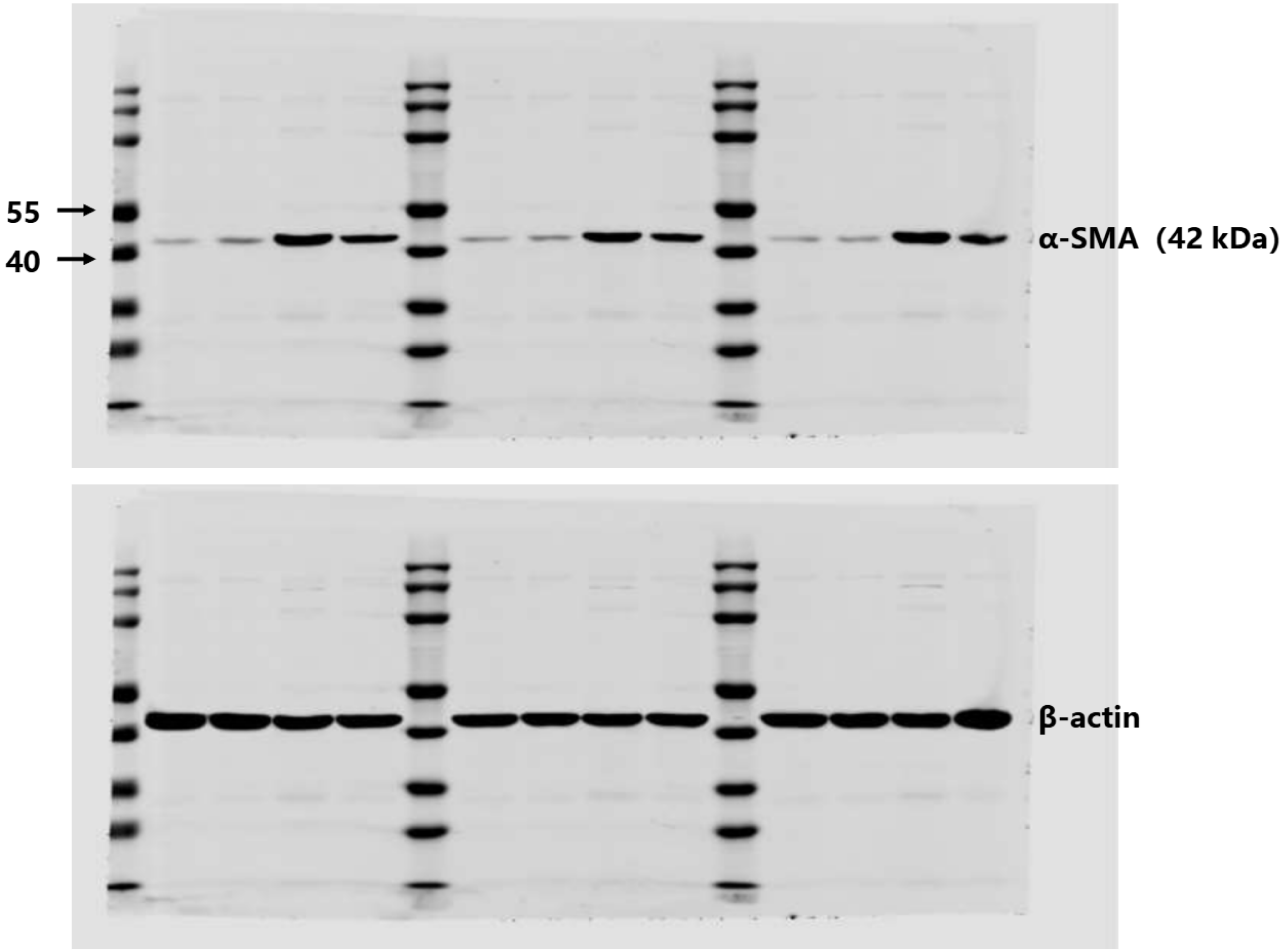

Lanes 7-10 of the unedited blot correspond to those shown in the cropped images within the supplementary materials.

Full unedited blot for Figure S4D

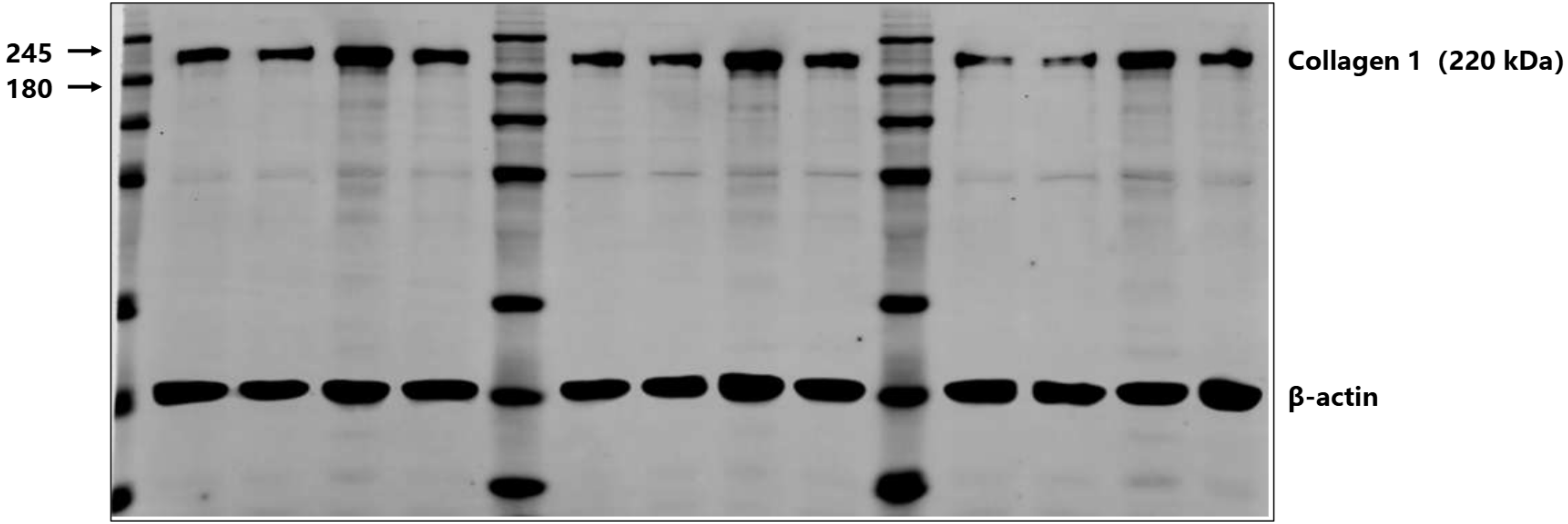

Lanes 2-5 of the unedited blot correspond to those shown in the cropped images within the supplementary materials.

Full unedited blot for Figure S4D

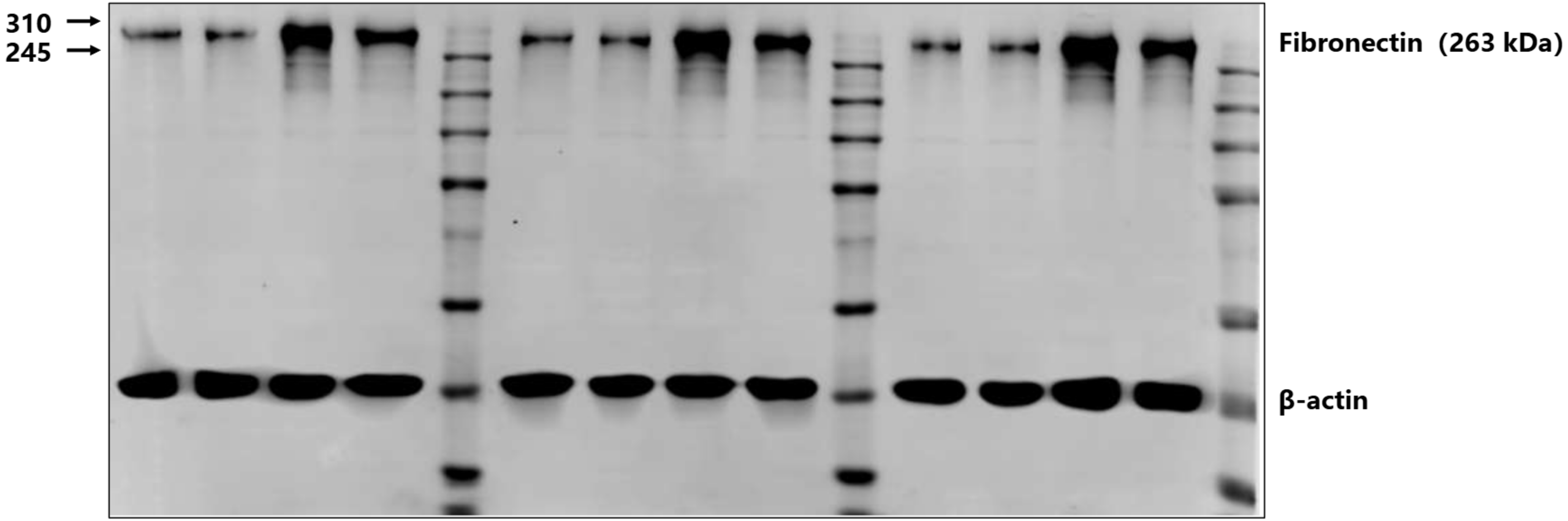

Lanes 6-9 of the unedited blot correspond to those shown in the cropped images within the supplementary materials.

Full unedited blot for Figure S4H

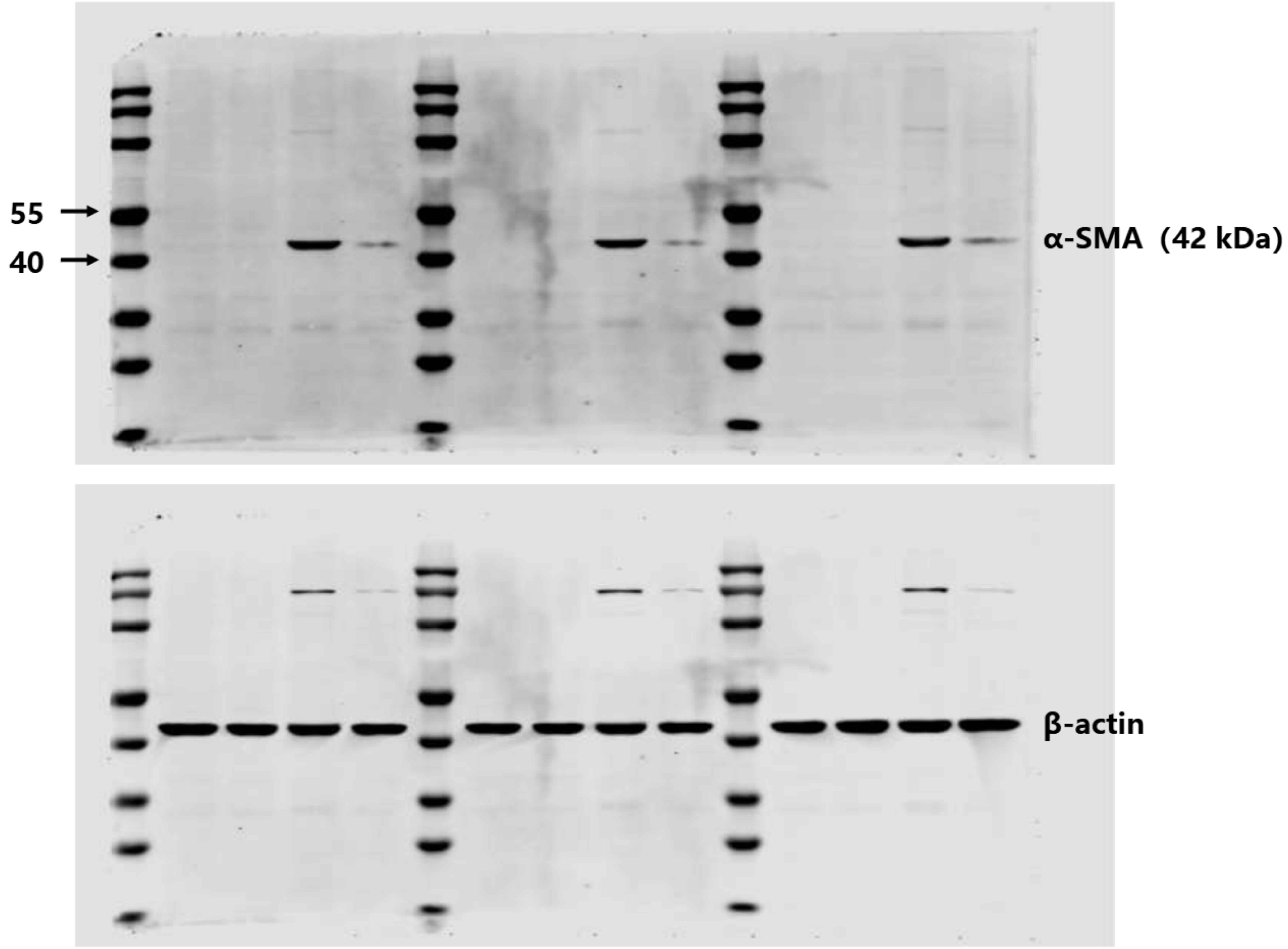

Lanes 12-15 of the unedited blot correspond to those shown in the cropped images within the supplementary materials.

Full unedited blot for Figure S4H

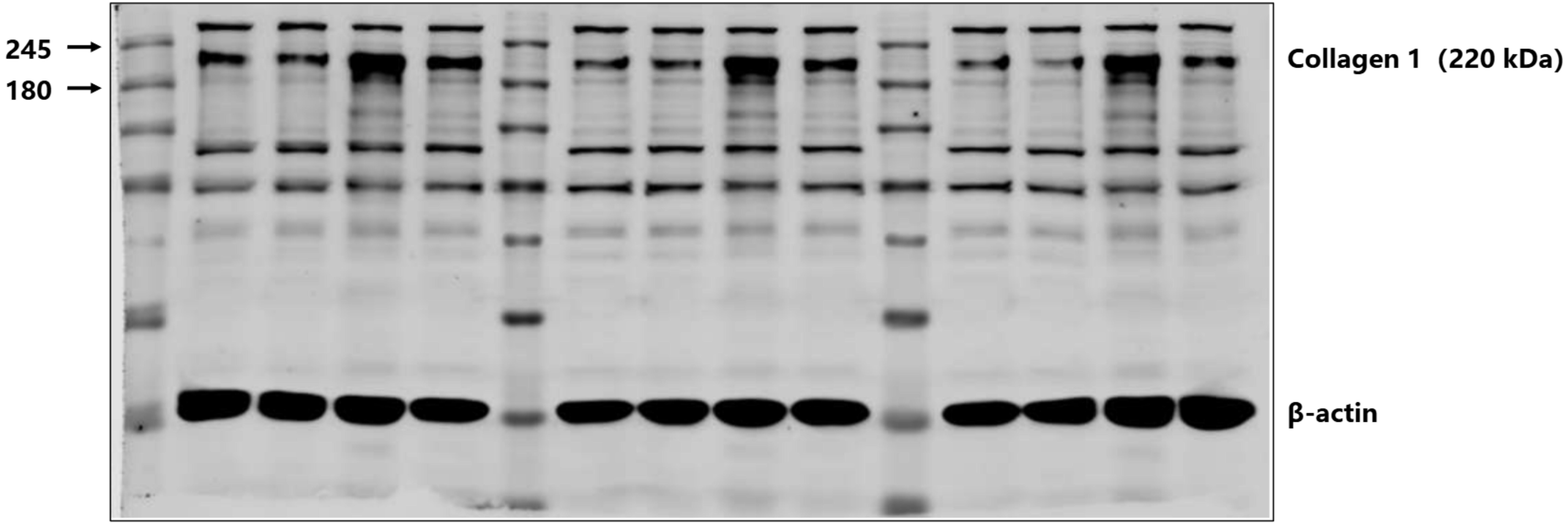

Lanes 2-5 of the unedited blot correspond to those shown in the cropped images within the supplementary materials.

Full unedited blot for Figure S4H

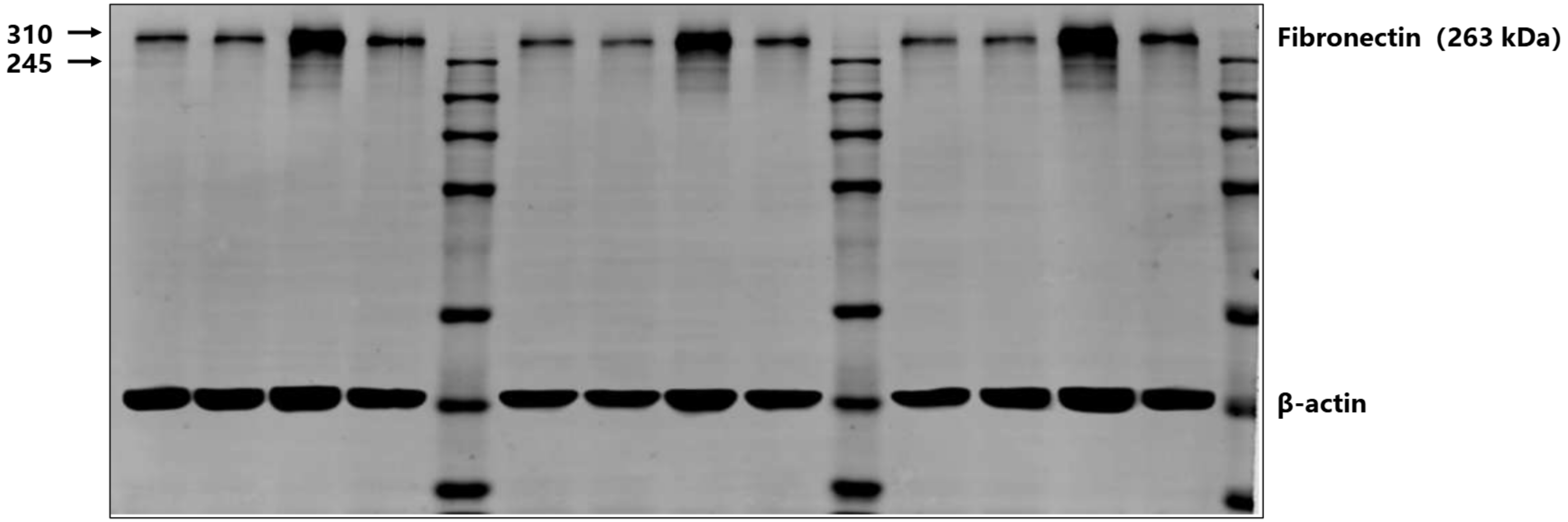

Lanes 11-14 of the unedited blot correspond to those shown in the cropped images within the supplementary materials.

Supplemental Methods S1. Primary antibodies used for Western blot in this study.

The primary antibodies used were as follows: anti-HIF-1α (ab179483; Abcam, UK), anti-Glut1 (12939S; Cell Signaling Technology, USA), anti-E-cadherin (14472S; Cell Signaling Technology, USA), anti-ZO-1 (21773-1-AP; Proteintech, China), anti-α-SMA (19245S; Cell Signaling Technology, USA), anti-Vimentin (10366-1-AP; Proteintech, China), anti-Collagen type I (ab270993; Abcam, UK), anti-Fibronectin (ab45688; Abcam, UK), anti-Alix (67715-1-Ig; Proteintech, China), anti-TSG101 (67381-1-Ig; Proteintech, China), anti-CD9 (98327S; Cell Signaling Technology, USA), anti-WLS (17950-1-AP; Proteintech, China), anti-Wnt5b (sc-376249; Santa Cruz, USA), anti-Wnt5b (bs-8010R; Bioss, China), anti-Wnt3a (bs-1700R; Bioss, China), anti-Wnt4 (bs-6134R; Bioss, China), anti-Wnt5a (bs-1948R; Bioss, China), anti-Wnt11 (bs-42347R; Bioss, China), anti-β-Catenin (8480S; Cell Signaling Technology, USA), anti-active β-Catenin (13537S; Cell Signaling Technology, USA), anti-FZD1 (DF4882; Affinity Biosciences, USA), anti-FZD1 (sc-398082; Santa Cruz, USA), anti-FZD2 (AF5282; Affinity Biosciences, USA), anti-LRP6 (DF2995; Affinity Biosciences, USA), anti-c-Jun (AF6090; Affinity Biosciences, USA), anti-p-c-Jun (Ser63) (AF3089; Affinity Biosciences, USA), anti-p-c-Jun (Ser73) (AF3095; Affinity Biosciences, USA), anti-NFAT1 (DF7189; Affinity Biosciences, USA), anti-NFAT2 (DF6446; Affinity Biosciences, USA), anti-β-Actin (66009-1-Ig; Proteintech, China), anti-GAPDH (60004-1-Ig; Proteintech, China), anti-Lamin B1 (12987-1-AP; Proteintech, China).
